# “Marriage” of Inorganic to Organic Chemistry as Motivation for a Theoretical Study of Chloroform Hydrolysis Mechanisms

**DOI:** 10.1021/acs.joc.4c00942

**Published:** 2024-09-20

**Authors:** Christina Stamou, Spyros P. Perlepes, Michail M. Sigalas, Dionissios Papaioannou, Athanassios C. Tsipis, Evangelos G. Bakalbassis

**Affiliations:** † Department of Chemistry, University of Patras, Patras 26504, Greece; ‡ Department of Materials Science, University of Patras, Patras 26504, Greece; § Laboratory of Inorganic Chemistry, Chemistry Department, University of Ioannina, Ioannina 45110, Greece; ∥ Aristotle University of Thessaloniki, University Campus, Thessaloniki 54124, Greece

## Abstract

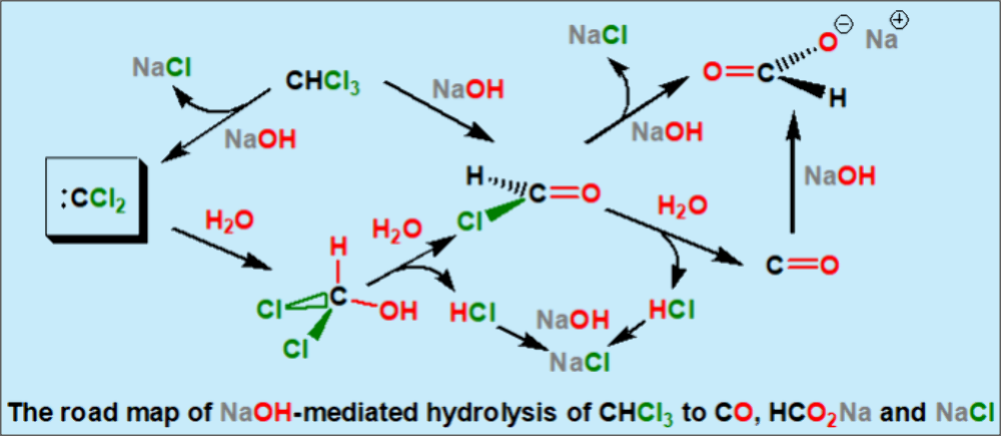

Incorporation of chlorides in coordination complexes, prepared by reactions in CHCl_3_, stimulated MP2 and DFT studies of its complete hydrolysis mechanisms. In excellent agreement with previous experimental results, the most important mechanism for CHCl_3_ basic hydrolysis at room temperature is the radical one producing :CCl_2_. The latter inserts into the H–O bond of H_2_O yielding dichloromethanol (**1**). The hydrolysis mechanism of α-H-lacking PhCCl_3_ to the corresponding dichloro(phenyl)methanol (**3**) was also studied. **1** decomposes by H_2_O to formyl chloride (**2**) and HCl. **2**, following a variety of pathways, leads to known CHCl_3_ hydrolysis products, such as CO (**4**) and formic acid (**6**), via the intermediates chloromethanediol (**5**), *s-cis*, *s-trans*-dihydroxycarbene (**ct**-**7**), and *s*-*trans*, *s*-*trans*-dihydroxycarbene (**tt-7**). Interestingly, both **ct**-**7** and **tt-7** intermediates have recently been implicated in the reduction of CO_2_ with H_2_ to **6**. The conversion of CO to HCOOH was studied. Most of the reactions studied are asynchronous concerted processes, the radical mechanism being a multistep one. The synthetic utility of this mechanism is briefly mentioned. To avoid chloride ions when performing reactions in CHCl_3_, we should use the solvent at room temperature even in the presence of water. This has been verified further by coordination chemistry reactions in progress.

## Introduction

The majority of chemical reactions and many measurements of properties are carried out in a solvent or a mixture of solvents. The properties of the solvent are of paramount importance to the success of the study. For the inorganic chemist, water has been the most important solvent, but many other solvents (including inorganic ones, e.g., HF, NH_3_, BrF_3_, IF_5_, Cl_3_PO, etc.) have been tried and found useful. For the organic chemist, organic solvents have dominated synthetic and characterization work. Solvents are also important in other branches of chemistry, such as medicine, pharmacy, and material science. Properties that chiefly determine the utility of a solvent are (i) the temperature range over which it is liquid; (ii) its dielectric constant; (iii) its acceptor and donor (Lewis acid–base) properties; (iv) its protonic acidity or basicity; and (v) the nature and extent of autoionization.^1^ Concerning point (iii), the ability of a solvent to keep a given solute in solution depends on its success in solvating the dissolved particles, i.e., to interact with them in a quasichemical way. For ionic solutes, the solvation of the cations is crucial; this is essentially the process of forming coordination complexes, in which the ligands are solvent molecules. The order of coordinating ability toward metal cations for some common organic solvents is DMSO > DMF ≈ Η_2_Ο > Me_2_CO > MeCN > MeNO_2_ > C_6_H_5_NO_2_ ≫ CH_2_Cl_2,_ CHCl_3_. When solvents act as acceptors, the positive ends of the solvent molecule dipoles will orient themselves toward the anions. The dielectric constant (point (ii)) and the ability to solvate ions (point (iii)) are related properties, which tend to increase or decrease simultaneously. Concerning points (iv) and (v), the solvents can be divided into protic (they contain ionizable protons), e.g., H_2_O, liquid HF, and liquid NH_3_, which characteristically undergo autodissociation, and aprotic solvents. There are three broad classes of the latter: (a) nonpolar (or very weakly polar) nondissociated liquids, which do not solvate strongly, e.g., hydrocarbons and CCl_4_; (b) nonionized but strongly solvating (generally polar) solvents, e.g., MeCN, DMF, DMSO, THF, and SO_2_; and (c) highly polar, autoionizing solvents, e.g., BrF_3_, IF_5_, and Cl_3_PO. CHCl_3_, which is the subject of the present study, is a nonpolar (its dielectric constant is 4.8), nondonor, and weakly protic solvent (although some organic chemistry textbooks describe it as aprotic) with almost negligible autodissociation.

Several coordination chemistry results gave the stimulus to this work.^2,3^ These will be briefly illustrated in the first part of the “Results and Discussion” section. Trying to ensure coordinative unsaturation at the metal center, we have been using nondonor solvents. Despite the poor solubility of many inorganic starting materials in CHCl_3_, this solvent proved to be successful in several cases.^2^ However, in most reactions, the products were found to contain chloro ligands (i.e., coordinated chlorides). Since the inorganic reactants did not have chlorides, the only possible source of chlorides is CHCl_3_. It is important to mention at this point that the reactions were performed with either the addition or the absence of external bases, usually at a refluxing temperature or even sometimes at room temperature.

Based on the above-mentioned, we thought it was of interest to investigate how chlorides are produced from chloroform using ab initio and density functional theory (DFT) calculations. It is well experimentally established that CHCl_3_ hydrolyzes under basic conditions (e.g., NaOH/H_2_O), producing CO, HCO_2_Na, and NaCl (the source of chlorides). Since in many of our reaction systems, no strong base was added, we thus decided to search for potential pathways of CHCl_3_ decomposition, not only under basic but also in neutral conditions (namely, by H_2_O alone). To the best of our knowledge, no such theoretical calculations have been conducted for the hydrolysis of CHCl_3_, although some studies have been reported on the interaction of dichlorocarbene (:CCl_2_)—the key radical intermediate in the basic hydrolysis of chloroform^4,5^—with one^6^ or two^7^ water molecules. We were also interested in identifying potential mechanisms through which CHCl_3_ ends up in CO and HCO_2_Na under basic hydrolysis, another unexplored area.

**Scheme 1:**
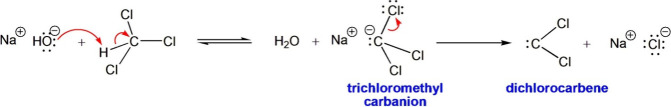
Formation of the Trichloromethyl Carbanion through the Radical Mechanism and Its Decomposition to the Active Intermediate Dichlorocarbene and Chloride Anion

CHCl_3_ is a special type of alkyl chloride for the following reasons: (i) it includes not only one but three very good leaving groups (Cl) on the same atom. The two leaving groups with their strong electronegative effect remove electron density from the C atom, thus reducing considerably the leaving group ability of the third Cl. Thus, the relative reactivity series in the S_N_2 reaction of CH_3_Cl, CH_2_Cl_2_, and CHCl_3_ is CH_3_Cl ≫ CH_2_Cl_2_ > CHCl_3_, and actually, the reaction of CHCl_3_, even with the strong PhS^–^ nucleophile, is negligibly slow; and (ii) on the other hand, because of the presence of three strong electron-withdrawing groups (Cl) on the same carbon, CHCl_3_ is weakly acidic, e.g., its p*K*_α_ value is almost identical to that of water. As a result, when chloroform reacts with strong nucleophiles, which at the same time are also strong bases (e.g., HO^–^), the H atom of this molecule is removed by the strong base in an almost balanced equilibrium giving rise to the trichloromethyl carbanion (Cl_3_C^–^). Thus, CHCl_3_ would be expected to react with H_2_O or other stronger nucleophiles, e.g., HO^–^ ions, through either S_N_1 or S_N_2 mechanism, as, for example, is the case for the classical alkyl halides methyl chloride and *tert*-butyl chloride proposed to react with H_2_O through S_N_2 and S_N_1 mechanisms, respectively. We should, however, note that recent DFT calculations have shown that the hydrolysis of both methyl chloride^8^ and *tert*-butyl chloride^8,9^ takes place mainly by the classical S_N_2 mechanism (backside attack by H_2_O); the only difference is that in the former case, the frontside attack (S_N_1-type mechanism) by H_2_O is disfavored by ca. 23 kcal mol^–1^ with regard to the backside attack, whereas in the latter case, this difference is only ca. 2 kcal mol^–1^.^8^ Of course, in the case of CHCl_3_, a third possibility, namely, a radical mechanism, is open with strong bases due to its relatively acidic H atom. The latter can be readily abstracted by the base creating the trichloromethyl carbanion which decomposes slowly to the neutral, high-energy, active intermediate dichlorocarbene and chloride ion (Scheme 1).

Another useful synthetic aspect in which the CHCl_3_/ HO^–^ system finds utility is the Reimer–Tiemann reaction, where chloroform and hydroxide ions formylate aromatic rings. The method is useful for phenols and certain electron-rich heterocyclic compounds, such as pyrroles and indoles.^10,11^ In particular, in the case of phenols (specifically the phenolate ions), the incoming group is directed *ortho*, unless both *ortho* positions are occupied, in which case the attack is *para*. With pyrroles or indoles, dichlorocarbene, in addition to the expected 2- or 3-formyl derivatives, respectively, also gives 3-chloropyridine and 3-chloroquinoline, respectively, through the so-called Ciamician–Dennstedt rearrangement.^12−14^ Also, dichlorocarbene adds to alkenes through a concerted [2 + 1] cycloaddition reaction^15,16^ and reacts with primary amines to give isonitriles (carbylamine reaction or Hofmann isonitrile synthesis).^17−19^

We therefore thought it was of interest to perform, using ab initio and DFT calculations, a complete study of the hydrolysis of CHCl_3_ in neutral and basic environments aiming to identify all possible pathways leading to its known hydrolysis products.

## Results and Discussion

### Previous Coordination Chemistry Results That Stimulated the Present Study

**Scheme 2:**
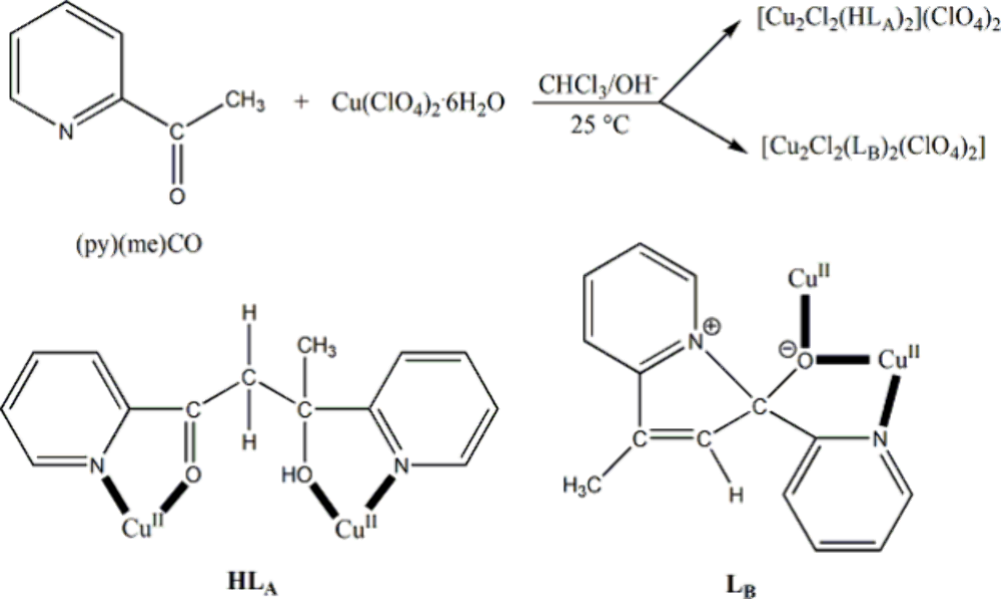
(Up) Reaction of (py)(me)CO and Cu(ClO_4_)_2_·6H_2_O in CHCl_3_/OH^–^ at Room Temperature That Gives Two Dinuclear Cu(II) Complexes Containing Terminal Chloro Ligands; (Bottom) Coordination Modes of the Ligands HL_A_ and L_B_ Derived from Metal Ion-Assisted Transformations of (py)(me)CO^,^ The H atom in the abbreviation of the coordinated ligand HL_A_ denotes the presence of an acidic hydrogen. The coordination bonds are drawn with bold lines.

**Scheme 3:**
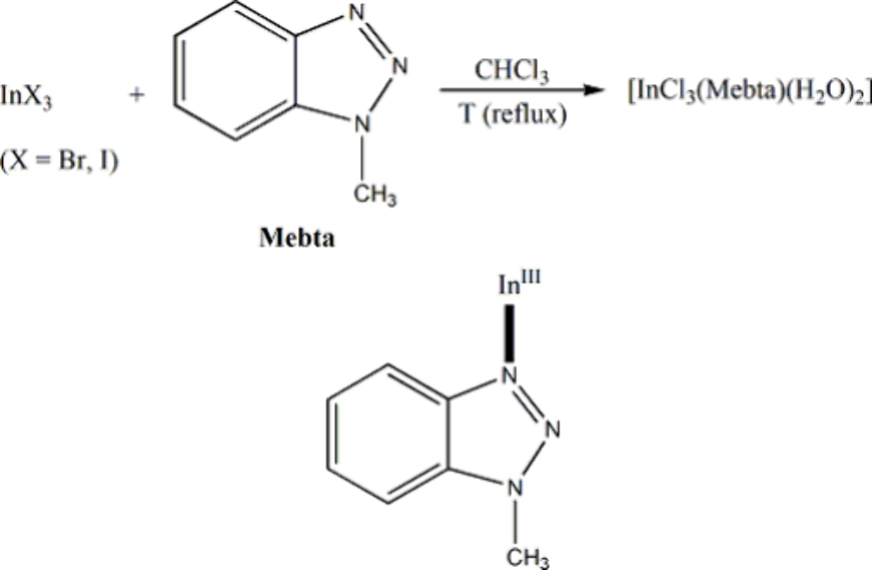
(Up) Reactions of Mebta and InX_3_ (X = Br, I) in CHCl_3_ under Refluxing Conditions in the Absence of an External Base That Give the Chloro-Containing Octahedral Complex [InCl_3_(Mebta)(H_2_O)_2_]; (Bottom) Monodentate Ligation Mode of the Coordinated Mebta Molecule in the Complex The coordination bond is drawn with a bold line.

As mentioned in the “Introduction” section, the stimulus of the present work was a variety of reactions in CHCl_3_, where the products (their structures were confirmed by single-crystal X-ray crystallography) contained a chloro ligand, not present in the starting materials; the only possible source of chlorides is the solvent used in the reactions. Two such reaction systems are illustrated in Schemes 2 and 3. In the former, the reaction of Cu(ClO_4_)_2_·6H_2_O and 2-acetylpyridine, (py)(me)CO, in CHCl_3_, under ambient conditions in a basic environment, gave the dinuclear complexes [Cu_2_Cl_2_(HL_A_)_2_](ClO_4_)_2_ and [Cu_2_Cl_2_(L_B_)_2_(ClO_4_)_2_], where HL_A_ is the transformed ligand 3-hydroxy-1,3-di(pyridin-2-yl)-butane-1-one and L_B_ is the zwitterionic-type, transformed ligand 3-hydroxy-1-methyl-3-(pyridin-2-yl)-3*H*-indolizin-4-ium. Both products contain one coordinated chloro group at each Cu^II^ center.^2^ In the latter, reactions of InX_3_ (X = Br, I) and 1-methylbenzotriazole (Mebta) in refluxing CHCl_3_, in the absence of an external base, gave the mononuclear complex [InCl_3_(Mebta)(H_2_O)_2_],^3^ bearing three chloro ligands at *fac* positions and two aqua ligands derived from the water molecule contained in the solvent and/or the inorganic starting material.^3^

### Theoretical Study

Considering the above discussion, we decided to look for all possible pathways for chloroform hydrolysis under both basic (NaOH/H_2_O and/or NaOH) and neutral conditions, i.e., with H_2_O only. The reaction steps involved in the entire CHCl_3_ hydrolysis mechanisms of all 20 reaction profiles studied have been scrutinized, and the transition states have been fully identified by monitoring the corresponding geometric and energetic reaction profiles using the MP2/cc-pVDZ, M06-2X/cc-pVTZ, and ωB97XD/cc-pVTZ calculation protocols in the gas phase. First, we will present the results of our study on the reaction of CHCl_3_ with water or NaOH alone or a combination of NaOH and water through one of the classical substitution nucleophilic (S_N_) reaction mechanisms, namely, S_N_1 or S_N_2, or the less common S_N_i and the alternative α-elimination reaction mechanism to give dichlorocarbene (coined as the radical mechanism^4^). The latter mechanism is available to CHCl_3_ because it incorporates a relatively acidic α-H. We should however bear in mind that the S_N_1 and the S_N_2 mechanisms are the two extremes of the S_N_1/S_N_2 mechanistic continuum, with S_N_i lying in between the two.^20^

#### Study of the S_N_ Mechanisms

**Figure 1:**
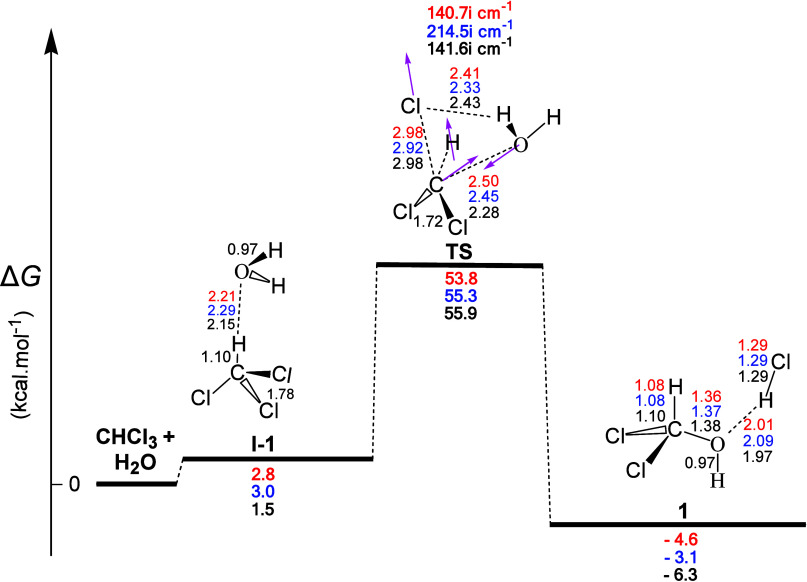
Gas-phase energy and geometry profile of intermediate **1** formation, derived from the CHCl_3_ + H_2_O interaction, calculated at the MP2/cc-pVDZ level (numbers in black), at the M06-2X/cc-pVTZ level (numbers in blue), and at the ωB97XD/cc-pVTZ level (numbers in red), along with selected structural data. Relative Δ*G* values (in bold) are given in kcal mol^–1^, imaginary frequencies (in bold) are given in cm^–1^, and calculated bond lengths (in plain) are given in Å.

The gas-phase energy (Δ*G*) and geometry profile of intermediate **1** formation, derived from the CHCl_3_+H_2_O interaction, calculated at the MP2/cc-pVDZ, M06-2X/cc-pVTZ, and ωB97xd/cc-pVTZ levels is shown in Figure 1. Moreover, selected structural data, corresponding mostly to hydrogen-bond (**H-b**) lengths, are also shown. It is obvious that all three theoretical levels used showed nearly the same bond lengths for each distinct bond type presented, and this was the case with all reaction profiles studied.

In the reaction profile of Figure 1 the CHCl_3_ molecule interacted with one water molecule resulting in the first intermediate **1** via both the pre-TS intermediate **I-1** and the transition state **TS.** Intermediate **I-1** involved one **H-b** formation between the O atom of the water molecule and the relatively acidic H atom of HCCl_3_. Moreover, the normal coordinate vectors of **TS** showed that the dominant motions involved the formation of a four-membered ring containing unequal C...Cl and C...O linkages (partial bonds). The former, followed by the concomitant H-abstraction, corresponded to the formation of the H–Cl bond, and the latter, followed by the concomitant water molecule OH-addition, corresponded to the formation of the C–OH bond. The intrinsic reaction coordinate (IRC) plots of the **TS**, given in the SI, show that the molecular mechanism was a concerted process,^21^ and this was the case with all the other reactions studied except the multistep reactions of the radical mechanism.

Taking into consideration the C–Cl (1.78 Å) and C–O (1.38 Å) bond lengths, the difference (Δ*R*) between these bonds in the ground state and the TS was calculated, at the MP2/cc-pVDZ level, and found to be Δ*R*(C–Cl) = (2.98–1.78) = 1.20 Å and Δ*R*(C–O) = (2.28–1.38) = 0.90 Å, respectively, indicating that C–O bond formation was delayed compared to C–Cl bond breaking. This points out to an asynchronous concerted mechanism which from the organic chemistry point of view would be classified as an S_N_2-type mechanism with hydrogen bond-assisted frontside attack of the weak incoming nucleophile (H_2_O). Perusal of Figure 1 revealed that the energy profiles calculated by either the MP2 or DFT methods were qualitatively the same. Notice that the results obtained with the higher quality cc-pVTZ basis set differed marginally and converged to the same result. On the other hand, the effect of basis set on the energetic results was exemplified by the lower quality basis set cc-pVDZ, where significant changes could be observed. Accordingly, the reaction was predicted to be weakly exergonic with calculated Δ*G* values equal to −4.6, −3.1, and −6.3 kcal/mol at the ωB97XD/cc-pVTZ, M06-2X/cc-pVTZ, and MP2/cc-pVDZ levels, respectively. Thereafter, the values given for the relative stability, the exer-/endergonicity, and the activation energy will correspond to the previous series of calculation protocols. Also, all three calculation protocols we employed gave us activation energy Δ*G^≠^* values higher than ca. 54.0 kcal mol^–1^, rendering this reaction pathway kinetically unfavorable. Finally, the O atom of **1** formed one **H-b** with the H atom of the HCl molecule.

Hoping that a second water molecule might facilitate the interaction of CHCl_3_ and H_2_O and thus lower the activation energy of the reaction, we next attempted the calculation of the CHCl_3_ + 2H_2_O reaction to form **1**. The results are depicted in Figure S1. In this case, the Δ*R* values were calculated as 0.94 Å for the C–Cl bond and 0.85 Å for the C–O bond, i.e., the two values were comparable indicating a slightly asynchronous concerted mechanism, which would also be classified as an S_N_2-type mechanism^21,22^ with hydrogen bond-assisted backside attack of the weak incoming nucleophile (H_2_O).

In the reaction profile of Figure S1 the CHCl_3_ molecule interacted with two H_2_O molecules resulting again in the first intermediate **1** via both the pre-TS intermediate **I-2** and the transition state **TS.** Intermediate **I-2** involved two **H-bs** formation between the O atom of the one water molecule and the relatively acidic H atom of HCCl_3_; the second one was formed between the two water molecules. Moreover, the normal coordinate vectors of **TS** showed that the dominant motions involved the formation of a six-membered ring containing unequal C...Cl and C...O linkages (partial bonds). The former, followed by the concomitant H-abstraction, corresponded to the formation of the H–Cl bond, and the latter, followed by the concomitant water molecule OH-addition, corresponded to the formation of the C–OH bond. The second water molecule did not participate in the formation of **1**.

Perusal of Figure S1 revealed that the energetic profile was basically the same for all three methods we employed, i.e., they converged to the same result. Accordingly, the reaction was predicted to be weakly exergonic with calculated Δ*G* values equal to −2.8, −3.1, and −6.7 kcal/mol. Also, all three computational protocols we employed gave us activation energy Δ*G^≠^* values higher than ca. 53.0 kcal mol^–1^, rendering this reaction pathway kinetically unfavorable. Nevertheless, as will be seen from the following hydrolysis mechanism study, the reaction profiles of Figures 1 and S1 are related to the safe use of CHCl_3_ in inorganic reactions (absence of chloride ions in the reaction mixture). Finally, dichloromethanol (**1**) formed three **H-b**s with H_2_O and HCl molecules.

**Figure 2:**
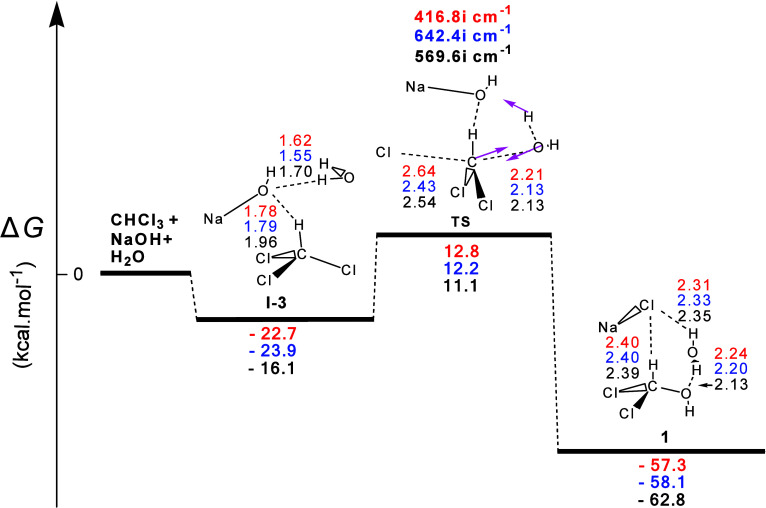
Gas-phase energy and geometry profile of intermediate **1** formation, derived from the CHCl_3_ + NaOH + Η_2_Ο interaction, along with selected structural data. For more details, see Figure 1 caption.

Unlike the previous two cases, the gas-phase reaction profile to form **1** derived from the CHCl_3_ + NaOH + H_2_O interaction, shown in Figure 2, was completely different. The first step corresponded to the formation of intermediate **I-3**, which involved two **H-b**s, because the O atom of NaOH formed a bond with the H atom of CHCl_3_ and a second one with the neighboring water molecule. Moreover, the normal coordinate vectors of the **TS** showed that the dominant motions involved the formation of a five-membered ring and unequal C...Cl and C...O linkages, corresponding to the C–Cl bond breaking and C–OH bond forming. Interestingly, the incoming OH group was that derived from the water molecule under NaOH activation. The almost identical Δ*R* values, 0.76 Å for the C–Cl bond and 0.75 Å for the C–O bond, indicated a synchronous concerted mechanism, which would also be classified as an S_N_2-type mechanism with backside attack of the nucleophile.^23^

Perusal of Figure 2 revealed that the reaction was strongly exergonic with calculated Δ*G* values equal to ca. −57.3, −58.1, and −62.8 kcal/mol and calculated activation Δ*G*^≠^ values equal to ca. 35.5, 36.1, and 27.2 kcal mol^–1^. Hence, the latter were much lower than those of the previous two figures. This could be attributed to its unhindered passage through the transition state. Indeed, the position of the NaOH molecule facilitated both the detachment of the H atom from the H_2_O molecule, thus the approach of HO^–^ to C, and the extrusion of Cl from the latter. Therefore, this hydrolysis mechanism was important because **1** could be indeed attained by gentle heating. This mechanism might offer an alternative explanation for the fact that the thiophenolate, a stronger nucleophile than the hydroxide ion, did not displace chloride from chloroform, a result which was related by Hine^4^ to the inability of CHCl_3_ to undergo an S_N_2 reaction with NaOH. We propose instead that thiophenolate, being a much weaker base than hydroxide, could not activate H_2_O to attack CHCl_3_. Finally, the Cl atom of NaCl formed two **H-b**s, one with the H atom of **1** and the other with the neighboring water molecule. The latter formed a second **H-b** with the O atom of **1**. The presence of three **H-b**s could well explain the strong stabilization of the products in this case.

On the other hand, in the reaction profile depicted in Figure S2, the CHCl_3_ molecule interacted with one NaOH molecule resulting again in **1**, via both the pre-TS intermediate **I-4** and the transition state **TS**. Intermediate **I-4** involved one **H-b** formation between the O atom of the NaOH molecule and the relatively acidic H atom of HCCl_3_. The most important characteristic of this profile was the different shape of the transition state, in which Cl, C, and OH of NaOH now adopted an almost linear arrangement. The normal coordinate vectors of the **TS** showed that the dominant motions involved the formation of a five-membered ring and unequal C...Cl and C...O linkages, corresponding to C–Cl bond breaking and C–OH bond forming. The Δ*R* values for the C–Cl (0.47 Å) and C–O (0.53 Å) bonds were comparable indicating a slightly asynchronous concerted mechanism, which would also be classified as an S_N_2-type mechanism with a backside attack of the incoming nucleophile HO^–^, with HO^–^ however coming now from NaOH.

Perusal of Figure S2 revealed that the reaction was also strongly exergonic with calculated Δ*G* values equal to −59.2, −59.1, and −61.3 kcal/mol and corresponding calculated activation energy Δ*G^≠^* values equal to ca. 38.3, 36.2, and 40.1 kcal mol^–1^. Hence, the latter are higher than those in Figure 2. Dichloromethanol (**1**) formed one **H-b** with the Cl atom, with the latter also exhibiting an electrostatic attraction to the Na atom.

**Figure 3:**
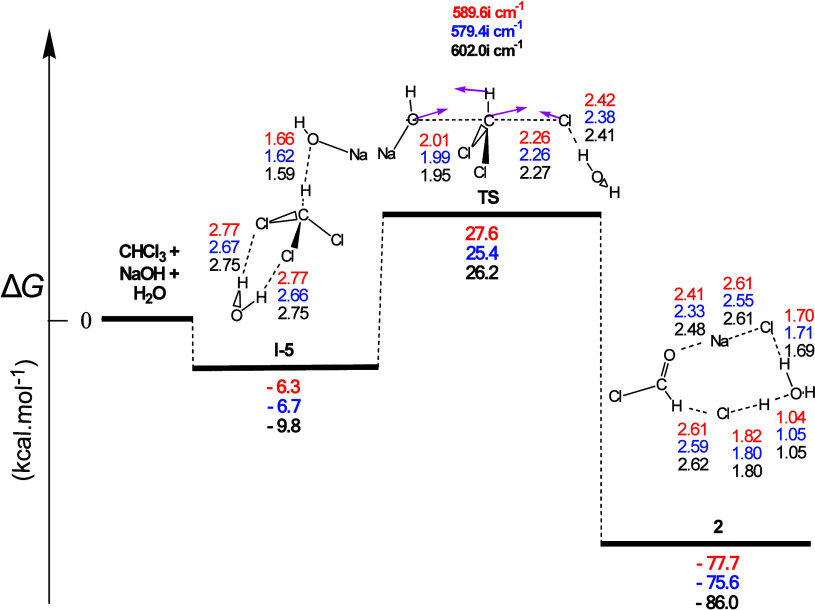
Gas-phase energy and geometry profile of intermediate **2** formation, derived from the CHCl_3_ + NaOH + Η_2_Ο interaction, along with selected structural data. For more details, see Figure 1 caption.

It is worth mentioning here that the CHCl_3_ + NaOH + Η_2_Ο interaction could also lead to a different reaction profile (see Figure 3), yielding however formyl chloride (**2**) instead of dichloromethanol (**1**).

In the reaction profile of Figure 3 the CHCl_3_ molecule interacted with one H_2_O molecule and one NaOH molecule resulting in **2** via both the pre-TS intermediate **I-5** and the transition state **TS**. Intermediate **I-5** involved three **H-bs** formation, one between the O atom of the NaOH molecule and the H atom of HCCl_3_ and two between the two Cl atoms of HCCl_3_ and the two H atoms of the H_2_O molecule. The most important characteristics of this profile were (a) the shape of the transition state, in which Cl, C, and OH of NaOH adopted an almost linear arrangement, and (b) the leaving group (Cl) in this case, formed a **H-b** with the water molecule. The latter could facilitate extrusion of Cl, which (c) could further reduce the activation energy value against that of an analogous reaction profile (see Figure S3), in which the strong nucleophile (HO^–^) formed a **H-b** with the water molecule, reducing its nucleophilicity. This could delay its approach to the C atom, thus increasing its activation energy value. Notably, the Δ*R* values for the C–Cl (0.49 Å) and C–O (0.57 Å) bonds calculated from Figure 3 and the corresponding ones 0.55 Å for the C–Cl bond and 0.50 Å for the C–O bond from Figure S3 are comparable, indicating slightly asynchronous concerted processes, which would also be classified as S_N_2-type mechanisms.

It is worth noting that Figures 2 and 3 based on the same CHCl_3_ + NaOH + Η_2_Ο interaction led to the formation of the two different intermediates **1** and **2,** respectively. Also, both reaction profiles presented very close Δ*G* values. Actually, in the former case, the presence of NaOH facilitated both the attack of the OH group of water on C due to the formation of a **H-b** of its second H with the O atom of NaOH and the detachment of Cl from C due to Na’s proximity to Cl. In the latter case, the detachment of Cl from C was facilitated by the **H-b** formed with the H atom of a water molecule; still, the OH group of NaOH was not prevented from attacking the C atom. In contrast, **2** presented greater exergonicity than **1**, accounting for its greater thermodynamic stability.

Perusal of Figures 3 and S3 revealed that both reactions were also strongly exergonic with calculated Δ*G* values equal to −77.7, −75.6, and −86.0 kcal/mol for the former and −69.8, −67.6, and −78.4 kcal/mol for the latter, exceeding the values of all others. The presence of the product pair **2** + HCl—more stable than **1** (vide infra)—in combination with 4 **H**-**b**s and 2 electrostatic interactions, namely, O...Na and Na...Cl, in the reaction products within the nine-membered ring formed, could justify the very strong exergonicity obtained for the hydrolysis shown in Figure 3. The one **H**-**b**, along with the one O...Na and the two Na...Cl electrostatic interactions in the hydrolysis products of Figure S3, could account for its lower exergonicity. The corresponding calculated activation energy Δ*G^≠^* values were ca. 33.9, 32.1, and 36.0 kcal mol^–1^, for Figure 3, and 40.6, 39.7, and 43.0 kcal mol^–1^, for Figure S3, the latter being higher than those in Figure 3.

Obviously, both **TSs** in Figures 3 and S3 led to dichloromethanol (**1**) as the substitution product, which then entered into a barrierless, possibly water-assisted, decomposition step to produce formyl chloride (**2**) and HCl.

#### Study of the Radical Mechanism

**Figure 4:**
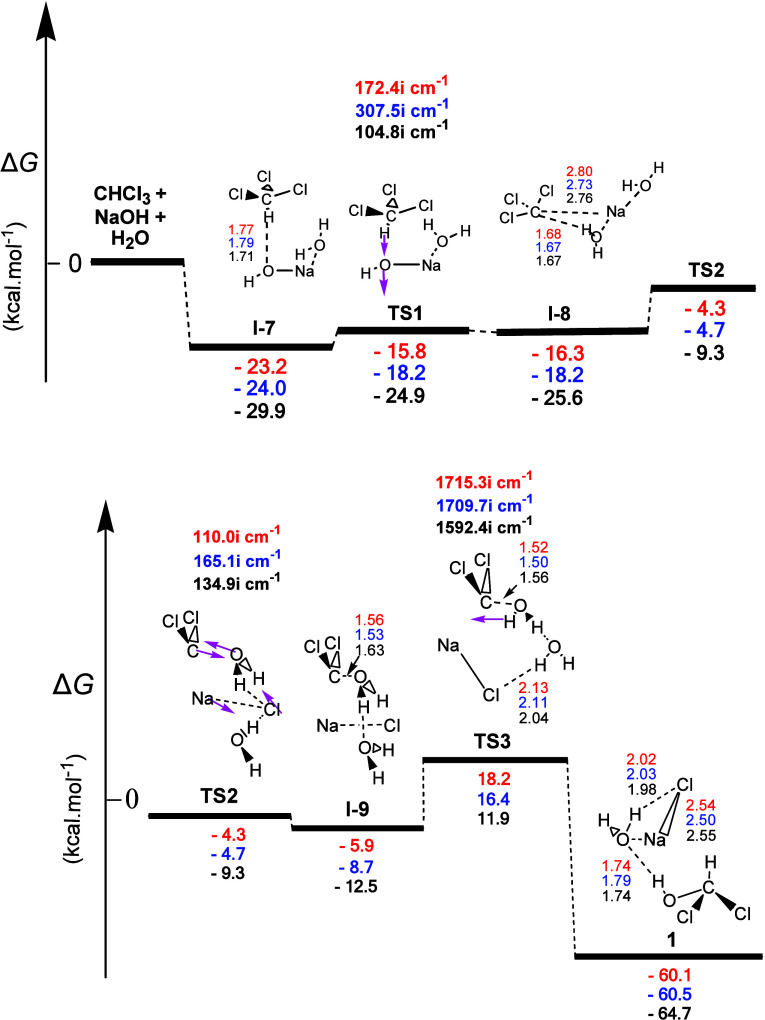
Gas-phase energy and geometry profile of intermediate **1** formation, derived from the CHCl_3_ + NaOH + Η_2_Ο interaction, via the radical mechanism, along with selected structural data. For more details, see Figure 1 caption.

It is worth emphasizing that the CHCl_3_ + NaOH + Η_2_Ο interaction could also lead to **1** (see Figure 4) via a radical mechanism. Unlike the former concerted mechanisms, the latter included three transition states. First, intermediate **I-7** was derived, in which the reactant triad formed one **H-b** (the one between the O atom of NaOH and the H atom of HCCl_3_) and one electrostatic interaction (that between Na and the O atom of the neighboring H_2_O molecule). The creation of these hydrogen bonding and electrostatic interactions could cause significant stabilization of the reactants in relation to their initial energy. Via the transition state **TS1**, surmounting an activation energy of ca. 7.4, 5.8, and 5.0 kcal mol^–1^, the triad of the reactants resulted in the second intermediate **I-8**, being only ca. 0.5, 0.0, and 0.7 kcal mol^–1^ more stable than **TS1**. The normal coordinate vectors (arrows) of the vibrational modes, corresponding to the imaginary frequency of **TS1**, showed that the dominant motion corresponded to the abstraction of the H atom of CHCl_3_ by the O atom of NaOH to form a second H_2_O molecule. Via the transition state **TS2**, surmounting an activation energy of ca. 12.0, 13.5, and 16.3 kcal mol^–1^, **I-8** resulted in the third intermediate **I-9**, being only 1.6, 4.0, and 3.2 kcal mol^–1^ more stable than **TS2**. The normal coordinate vectors (arrows) of the vibrational modes, corresponding to the imaginary frequency of **TS2**, showed that the dominant motions corresponded to (a) the abstraction of the Cl atom from CCl_3_; (b) the breaking of the Na...OH_2_ electrostatic attraction due to the formation of the second H_2_O molecule; and (c) the approach of the latter to dichlorocarbene (CCl_2_). It is worth mentioning here that **I-9** was a mixture of compounds comprising two H_2_O molecules, NaCl and the dichlorocarbene radical. Therefore, at room temperature, via the radical mechanism, the dichlorocarbene radical can be generated, which can give a series of useful reactions. If this does not happen, then with gentle heating, dichloromethanol (**1**) can be formed. Finally, via the transition state **TS3,** surmounting an activation energy of ca. 24.1, 25.1, and 24.4 kcal mol^–1^, being the rate-determining step of the reaction, **I-9** was transformed to dichloromethanol (**1**). The normal coordinate vectors of the vibrational modes, corresponding to the imaginary frequency of **TS3**, showed that the dominant motions corresponded to the abstraction of the H atom of the H_2_O molecule close to the CCl_2_ by its C atom and the weak C...OH (1.56 Å) linkage, resulting in the formation of the C–OH bond of **1**. The two O...H and H...Cl **H**-**bs**, along with the electrostatic interactions of O...Na and Na...Cl in the hydrolysis products of Figure 4, could account for its strong exergonicity (ca. −60.1, −60.5, and −64.7 kcal mol^–1^). It should be stressed here that the present radical mechanism, involving the dichlorocarbene radical as its active intermediate, is the main hydrolysis mechanism of CHCl_3_, in excellent agreement with the experimental data of Hine.^4^

We were also interested to see whether the radical mechanism can operate through the interaction of the molecular triad CHCl_3_ + 2Η_2_Ο. The results of the calculation are depicted in Figure S5, showing that it was a two-**TS** reaction profile process with main characteristics: (a) its rate-determining step was **TS2** with an activation energy of ca. 77.1, 76.3, and 71.6 kcal mol^–1^, implying that this particular reaction is not likely to occur. Despite that, (b) intermediate **I-11** was a mixture of compounds comprising two water molecules, HCl, and dichlorocarbene; and (c) the two H...Cl along with the O...H 3**H**-**bs** of the products could account well for its weak exergonicity with calculated Δ*G* values equal to ca. −3.1, −2.8, and −5.0 kcal mol^–1^.

The results of our calculations on the possible interactions of CHCl_3_ with water alone, through either the concerted or the radical mechanisms, are in agreement with classical organic chemistry considerations which would predict that water alone cannot react with CHCl_3_ for the following reasons: (a) the dichloromethyl carbocation is a species of high energy to be formed under an S_N_1 mechanism because the destabilizing effect of the two strong electron-withdrawing chlorine atoms (−I effect) more than exceeds their weak electron-donating mesomeric effect (+M); (b) H_2_O is a weak nucleophile to participate in an S_N_2 reaction with CHCl_3_ because it has to overcome the electronic cloud of the other two Cl atoms on approaching the electropositive carbon atom from the opposite direction from which the leaving group is outgoing; and (c) H_2_O is such a weak base (p*K*_a_ of H_3_O^+^ = −1.74) that cannot abstract the weakly acidic proton of CHCl_3_ (p*K*_a_ = 15.5). Indeed, CHCl_3_ does not react with water even upon heating at 90 °C for 12 h.^4^

#### Study of the PhCCl_3_ Hydrolysis Mechanism

Before proceeding to the last part of the hydrolysis of CHCl_3_, we thought it was of interest to also briefly refer to the study of the hydrolysis of PhCCl_3_, a compound where the H atom of CHCl_3_ has been replaced by Ph, thus eliminating the possibility of the radical mechanism. To the best of our knowledge, this reaction has been only studied experimentally under a variety of environments (neutral, acidic, basic, and nucleophilic) and temperatures (5–30 °C),^24,25^ and the S_N_1 mechanism has been proposed to account for the results obtained.^25^

**Figure 5:**
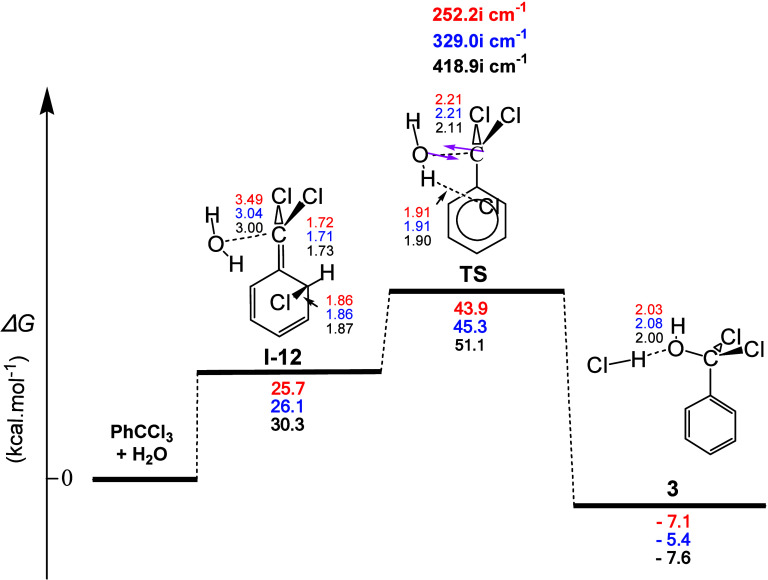
Gas-phase energy and geometry profile of intermediate **3** formation, derived from the PhCCl_3_ + Η_2_Ο interaction, along with selected structural data. For more details, see Figure 1 caption.

The reaction profile of the interaction of PhCCl_3_ with one molecule of H_2_O is given in Figure 5. As can be seen from this figure, the specific mechanism presented some characteristics similar to those of CHCl_3_. Indeed, the reaction profile showed a concerted mechanism, high calculated activation energy Δ*G*^≠^ values equal to ca. 43.9, 45.3, and 51.1 kcal mol^–1^, low exergonicity with calculated Δ*G* values equal to ca. −7.1, −6.4, and −7.6 kcal mol^–1^, and the same type of final intermediate PhC(OH)Cl_2_ (**3**). It is worth emphasizing the particularity that arises for the structure of intermediate **I-12**, which also gives it its high energy value. Its energetic instability arose from the fact that branched Cl binds to an *o*-C atom of the phenolic ring, thereby removing its aromatic stabilization. Nevertheless, the unstable structure of intermediate **I-12** easily changed to the transition state, where Cl was removed from the aromatic ring and H-bonded with the H_2_O molecule to finally give the HCl molecule in the products. The high value of the activation energy in this case is in excellent agreement with the fact that the direct hydrolysis of benzotrichloride with water alone to produce benzoic acid and HCl is effective at high temperatures (e.g., around 140–190 °C) under pressure, or temperatures in the neighborhood of 100–110 °C, when anhydrous ZnCl_2_ is used as a catalyst.^26^ In terms of classical organic chemistry considerations, the present mechanism involves migration of the α-Cl atom to the *o*-position of the aromatic ring with loss of resonance energy of the aromatic system, followed by an S_N_1′-like (allylic rearrangement) reaction which restores aromaticity.

**Figure 6:**
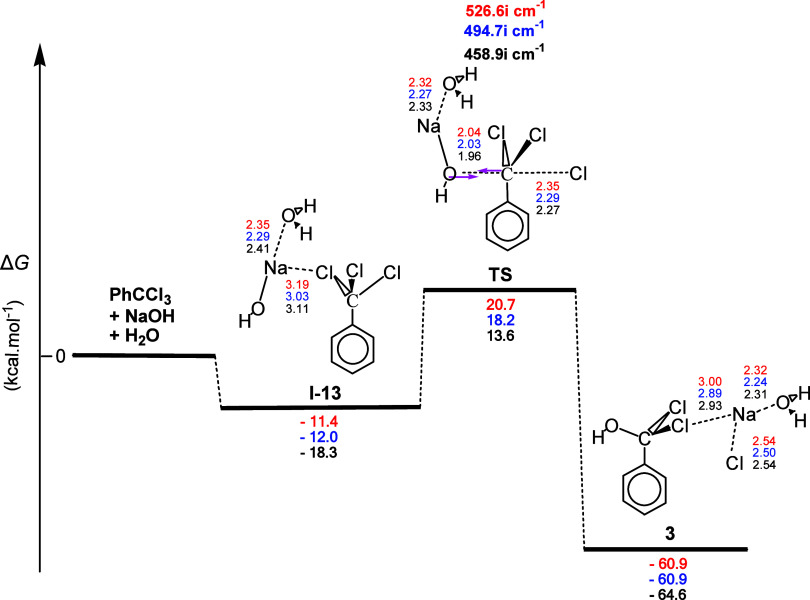
Gas-phase energy and geometry profile of intermediate **3** formation, derived from the PhCCl_3_ + NaOH + Η_2_Ο interaction, along with selected structural data. For more details, see Figure 1 caption.

In contrast to the previous one, the reaction profile of the PhCCl_3_ + NaOH + H_2_O interaction, shown in Figure 6, could be characterized as an energetically allowed concerted one. Indeed, with mild heating, the reaction could easily overcome the relatively low calculated activation energy Δ*G*^≠^ values of ca. 32.1, 30.2, and 31.9 kcal mol^–1^ and give **3**.

The Δ*R* values for the C–Cl and the C–O bonds were comparable, namely, 0.49 and 0.58 Å, respectively, indicating a slightly asynchronous concerted reaction, which could be classified as an S_N_2-type mechanism. Moreover, it was also a strongly exergonic reaction with calculated Δ*G* values equal to ca. −60.9, −60.9, and −64.6 kcal mol^–1^, possibly because its product involves two Na...Cl and one Na...O electrostatic interactions. The milder temperature required for the hydrolysis of benzotrichloride in the presence of a base can be seen in industrial processes involving an aqueous solution of Ca(OH)_2_ with (iron powder or iron salts) or without the catalyst^26,27^ or an aqueous solution of CaCO_3_ kept at 80–90 °C.^27^ It should be noted that the study of the alternative interactions of PhCCl_3_ with two H_2_O molecules or NaOH alone failed to produce acceptable mechanistic pathways and was not examined further.

#### Study of the Last Steps Completing CHCl_3_ Alkaline Hydrolysis

##### Conversion of Dichlorocarbene to Dichloromethanol (**1**)

Based on experimental observations, Hine^4^ proposed that dichlorocarbene can be converted to the final alkaline (NaOH) hydrolysis products, namely, CO and HCO_2_Na, by a fast NaOH-mediated hydrolysis of :CCl_2_. Alternatively, an initial slow attack of the dichlorocarbene C atom by the weak nucleophile, but present in huge excess being the reaction solvent, H_2_O produces ylid H_2_O(+)—(−)CCl_2_, which finally decomposes also by fast NaOH-mediated hydrolysis, with the former mechanism considered as much more probable than the latter.^4^ Robinson, also based on experimental evidence, concluded that H_2_O, and not the much stronger nucleophile HO^–^, reacts exclusively, or almost exclusively, with dichlorocarbene to give the aforementioned ylide, which decomposes to CO and HCl by an initial proton abstraction by the hydroxide ion, followed by chloride ion expulsion to give the new carbene C(OH)Cl. The latter further decomposes by chloride ion loss, followed by proton abstraction.^28^ On the other hand, Pliego and De Almeida showed through ab initio studies that the ylid H_2_O(+)—(−)CCl_2_, which anyhow had not been detected experimentally, cannot exist and that the CCl_2_ singlet carbene directly inserts into the H–O bond of a water molecule resulting in dichloromethanol (**1**).^29^ Later on, the same authors came up with an improved mechanism involving one molecule of dichlorocarbene and two molecules of water creating a five-membered ring transition state of lower activation energy than the one in the interaction CCl_2_ + H_2_O.^7^ According to this model, the H atom and the HO group connected to the C atom of CCl_2_ are from two different water molecules.

A DFT study published later on also showed a direct insertion mechanism for the reaction CCl_2_ + H_2_O with activation energies ca. 14.7 and 13.4 kcal mol^–1^, depending on whether the B3LYP/6-311G or the MP2/DZP/SCF/DZP level of theory was employed.^30^ This barrier is ca. 3.3 kcal mol^–1^ higher than the one calculated using MP2/DZP for the reaction CCl_2_ + 2H_2_O.^7^ Also, a detailed theoretical study for the interactions CCl_2_ + H_2_O and CCl_2_ + 2H_2_O, using the B3LYP/6-311++G(d,p) level, has been reported verifying that indeed the CCl_2_ + 2H_2_O interaction involves a smaller activation energy than the former one and thus results in a larger rate constant.^31^

Concerning the results of the present study in the context of the conversion of dichlorocarbene to dichloromethanol (**1**) (see Figure S4 for a detailed picture of the TS3 of Figure 4), the following comments could be made. The formation of the two new bonds C–H and C–O and the breaking of the H–O bond of water proceed at almost the same extent (Δ*R* (C–H) = 0.21 Å, Δ*R*(C–O) = 0.18 Å, and Δ*R* (H–O) = 0.21 Å), thus indicating a highly concerted process for the insertion step, in excellent agreement with our theoretical results. There are also two strong **H-b**s, one with the noninteracting carbene second water molecule and the other of the latter water molecule with the chloride ion. This second water molecule obviously helps to increase the nucleophilicity of the hydroxyl group attacking the carbene while at the same time somehow retarding the cleavage of the H–O bond. These findings are in accord with the above-mentioned ab initio studies indicating a direct insertion mechanism for the reaction of dichlorocarbene with one molecule of water,^29,30^ which involves a higher activation energy (lower reaction rate indicating a slow step as suggested by Pliego and De Almeida).^29^ Τhis can be however surmounted by heating the reaction mixture at a temperature slightly higher than that of the ambient, e.g., 36 °C used by Hine in his experiments.^4^ On the other hand, it is different from the alternative interaction CCl_2_ + 2H_2_O proposed by Pliego and De Almeida,^7^ in that the second water molecule does not activate the first one through a five-membered TS but through activating the hydroxyl O atom for nucleophilic attack on the carbene C atom. It is also interesting to note that despite its known volatility, dichloromethanol (**1**) was the product of all but two of the reaction profiles studied. Indeed, calculations have shown that **1** was 7.2 kcal mol^–1^ less stable than its next more stable counterpart formyl chloride (**2**) + HCl. Furthermore, **1** could also be obtained either (a) on gentle heating from the reactants CHCl_3_ + NaOH + H_2_O (Figure 2) and/or (b) from the reaction of CHCl_3_ with the strong base, NaOH, also upon heating (Figure S2).

##### Conversion of Dichloromethanol (**1**) to Formyl Chloride (**2**)

Based on ab initio calculations, Pliego and De Almeida proposed that dichloromethanol (**1**) decomposes in a fast step to formyl chloride and hydrogen chloride ion by a hydroxide-catalyzed mechanism, through abstraction of the acidic hydroxyl proton first, followed by chloride ion elimination, and that this process has no energy barrier.^29^ We noticed above that the triad of reactants CHCl_3_ + NaOH + H_2_O at higher temperatures could lead directly to the formation of **2**, instead of **1** (Figures 3 and S3). Hence, we thought at this point that it would be interesting to shed light on the mechanistic aspects of the transformation of dichloromethanol (**1**) to formyl chloride (**2**).

We first attempted to find the reaction profile for the interaction of **1** with NaOH but had no success. Obviously, NaOH alone cannot be involved in the transformation of **1** to **2**. The same results were also obtained with triad **1** + NaOH + H_2_O. We then focused on the possibility of H_2_O-alone-assisted decomposition of **1** to **2** + HCl.

**Figure 7:**
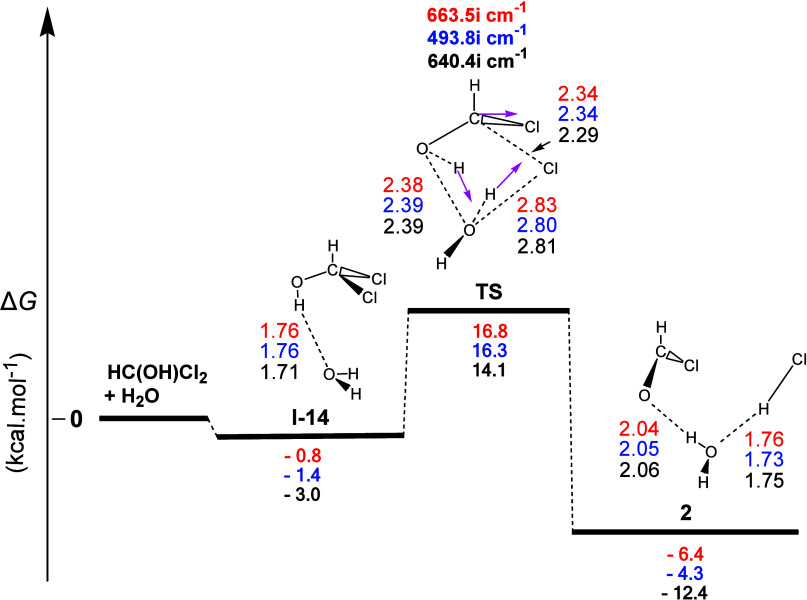
Gas-phase energy and geometry profile of intermediate **2** formation, derived from the **1** + Η_2_Ο interaction, along with selected structural data. For more details, see Figure 1 caption.

The gas-phase reaction profile to form **2**, derived from the **1** + H_2_O interaction, is shown in Figure 7. The first step (**I-14**) corresponded to the formation of **H-b** between the O atom of the H_2_O molecule and the H atom of the hydroxyl group of **1**. The **TS** that followed showed that the molecular mechanism was a concerted process. The normal coordinate vectors showed that the dominant motions involve the formation of a six-membered ring containing the C...Cl (2.29 Å), Cl...H (1.83 Å), H...O (1.04 Å), O...H (1.12 Å), and H...O (1.31 Å) linkages, corresponding to (a) the detachment of a Cl atom from **1**, (b) the H–Cl bond forming occurring simultaneously by the abstraction of the H atom of the H_2_O molecule, and (c) the abstraction of the H atom of the OH group of **1** by the O atom of the H_2_O molecule, with the breaking of the H–O bond of **1** slightly delaying [Δ*R*(O–H) = 0.34 Å], compared to the C–Cl bond cleavage [Δ*R*(C–Cl) = 0.51 Å]. At the same time, the C–O bond has been considerably shortened (from 1.38 to 1.25 Å), being a partial double bond. Calculations also showed that this process (i) was weakly exergonic with calculated Δ*G* values equal to ca. −6.4, −4.3, and −12.4 kcal mol^–1^ and (ii) had calculated activation energy Δ*G^≠^* values of ca. 17.6, 17.7, and 17.1 kcal mol^–1^. Hence, this transformation can occur at room temperature. Finally, the three products (**2**, H_2_O, and HCl) of the reaction formed two **H-b**s.

**Figure 8:**
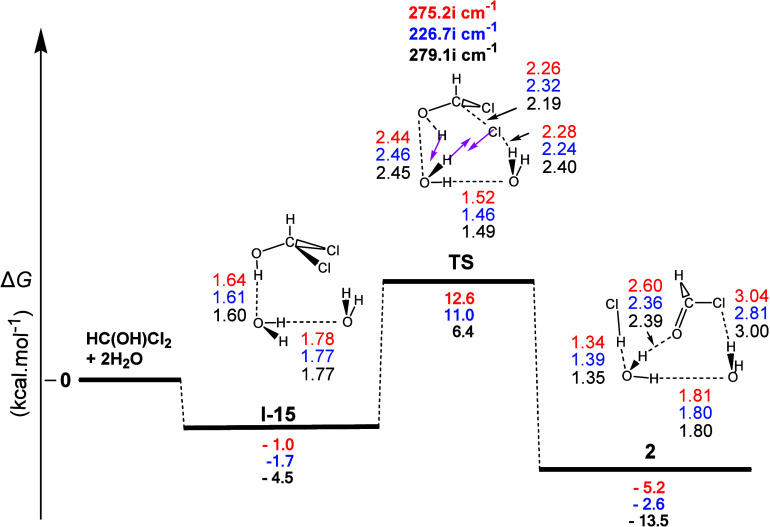
Gas-phase energy and geometry profile of intermediate **2** formation, derived from the **1** + 2Η_2_Ο interaction, along with selected structural data. For more details, see Figure 1 caption.

The gas-phase reaction profile to form **2**, derived from the **1** + 2H_2_O interaction, is shown in Figure 8. The first step (**I-15**) corresponded to the formation of two **H-b**s between the O atom of one H_2_O molecule and the H atom of the hydroxyl group of **1** and that between the O atom of the second water molecule and the H atom of the first one. Moreover, the normal coordinate vectors showed that the dominant motions in the **TS** involved the formation of an eight-membered ring, including both water molecules now, containing a series of linkages, corresponding to (a) the detachment of a Cl atom from **1**, (b) the H–Cl bond formation occurring simultaneously with the abstraction of the H atom of the water molecule, and (c) the abstraction of the H atom of the OH group of **1** by the O atom of the second water molecule, reforming thus the latter molecule. For the most interesting C...Cl, H...O, and O...C bonds, the distances were found to be 2.19, 1.42, and 1.24 Å, respectively, with the breaking of the H–O bond of **1** to take place in a synchronous manner [Δ*R*(O–H) = 0.45 Å] with the C–Cl bond cleavage [Δ*R*(C–Cl) = 0.41 Å]. At the same time, the C–O bond has been considerably shortened (from 1.38 to 1.24 Å), also being a partial double bond. Calculations showed that this process (i) was weakly exergonic with calculated Δ*G* values equal to ca. −5.2, −2.6, and −13.5 kcal mol^–1^ and (ii) had calculated activation energy Δ*G*^≠^ values equal to ca. 13.6, 12.7, and 10.9 kcal mol^–1^, accounting well for a faster transformation than the previous one at room temperature. Finally, the four products (**2**, two H_2_O and HCl) of the reaction formed four **H-b**s. Calculations showed that the decomposition of dichloromethanol (**1**) to formyl chloride (**2**) and HCl did not require the presence of NaOH to occur but was a water-assisted concerted HCl elimination process.

##### Conversion of Formyl Chloride (**2**) to the Final Products of Hydrolysis, Carbon Monoxide (**4**) and Sodium Formate (**6**)

We have seen above that dichloromethanol (**1**) decomposes to formyl chloride (**2**) and HCl. Formyl chloride, although more stable than dichloromethanol, is itself unstable and decomposes above −60 °C to carbon monoxide (**4**) and hydrogen chloride.^32^ Indeed, our calculations have shown that the couple CO + HCl is more stable than ΗCOCl by 9.8 kcal mol^–1^. Also, formyl chloride can be hydrolyzed to formic acid and hydrogen chloride, with the couple HCO_2_H + HCl being more stable than the couple HCOCl + H_2_O by 14.9 kcal mol^–1^. It has been reported^28,29^ that the decomposition of formyl chloride to CO is also catalyzed by the hydroxide ion present in the basic hydrolysis of chloroform and that the sodium formate also produced is the result of the addition of NaOH to carbon monoxide. On the other hand, it has been also shown experimentally that in alkaline solutions the hydroxide ion-induced hydrolysis of formyl chloride competes with its decomposition to CO and HCl, and the two reaction rates actually become equal in strongly basic solutions, thus implying that sodium formate might also be produced by the alkaline hydrolysis of formyl chloride. In acidic or neutral pH solutions, however, formyl chloride mainly decomposes to CO and HCl.^33^

We therefore thought it was of interest to draw light on the possible mechanism for these transformations. Accordingly, we first attempted to find a potential transition state for the reaction of HCOCl with NaOH to produce CO, NaCl, and H_2_O but with no success. Therefore, NaOH is not involved in the decomposition of HCOCl to CO and HCl. We then examined the possibility of water-alone-assisted decomposition of HCOCl to CO and HCl and of the hydrolysis of HCOCl to HCO_2_H and HCl.

**Figure 9:**
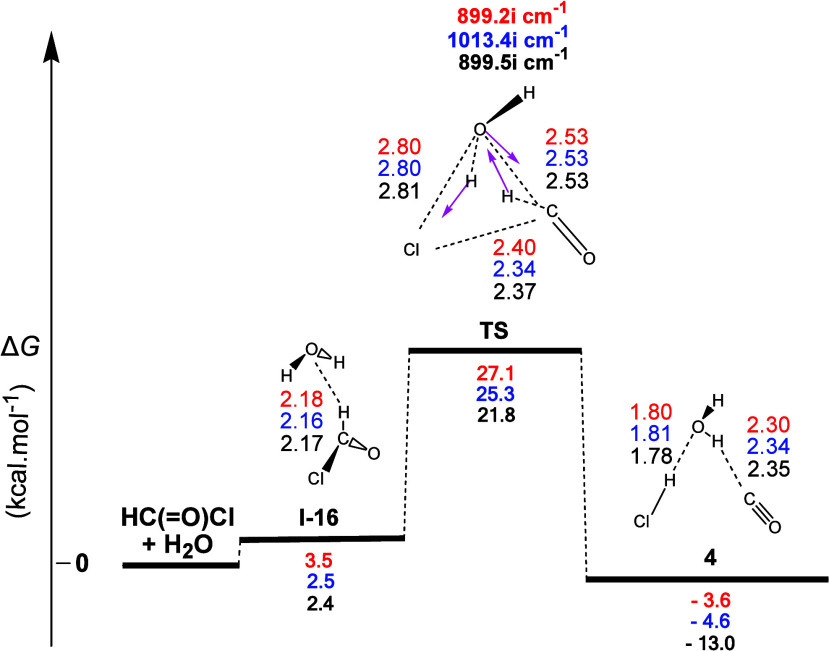
Gas-phase energy and geometry profile of product **4** formation, derived from the **2** + Η_2_Ο interaction, along with selected structural data. For more details, see Figure 1 caption.

The gas-phase reaction profile to form carbon monoxide (**4**), derived from the **2** + H_2_O interaction, is shown in Figure 9. The first step (**I-16**) corresponded to the formation of one **H-b** between the O atom of the water molecule and the H atom of the formyl chloride (**2**). The **TS** that followed showed that the molecular mechanism was a concerted process. The normal coordinate vectors showed that the dominant motions involved the formation of one five-membered ring corresponding to (a) the detachment of the Cl atom from **2**, (b) the H–Cl bond forming occurring simultaneously by the abstraction of the H atom of the water molecule by the Cl atom, and (c) the abstraction of the H atom of **2**, by the hydroxyl group leading to the reformation of the water molecule. The most interesting linkages in the **TS**, namely, the C...Cl and H...C ones are 2.37 and 1.32 Å, respectively, with the corresponding Δ*R* values calculated as 0.59 Å for the C–Cl bond and 0.22 Å for the C–H bond, thus indicating that the C–H bond cleavage delays compared to the C–Cl bond cleavage. Interestingly, the length of the C=O bond in **TS** was 1.15 Å, indicating the formation of a carbon monoxide molecule already in the **TS**. Calculations also showed that this process (i) was weakly exergonic with calculated Δ*G* values equal to ca. −3.6, −4.6, and −13.0 kcal mol^–1^ and (ii) had calculated activation energy Δ*G^≠^* values equal to ca. 27.1, 25.3, and 21.8 kcal mol^–1^. Therefore, this transformation can occur at a slightly higher temperature than the ambient. Finally, the three products (**3**, H_2_O, and HCl) of the reaction formed two **H-bs**, namely, H...O and H...C.^34^

This was also the case with the reaction profile for the **2** + 2H_2_O interaction (see Figure S6) to form **4.** Its **TS** showed that the molecular mechanism was also a concerted process. Moreover, this interaction had a similar energy profile to the **2** + H_2_O one, namely being a weakly exergonic reaction with calculated Δ*G* values equal to ca. −2.6, −4.2, and −13.4 kcal mol^–1^ with calculated activation energy Δ*G*^≠^ values equal to ca. 29.2, 26.1, and 22.9 kcal mol^–1^. The lengths of the C...Cl and C...H linkages are 2.40 and 1.26 Å, respectively. The Δ*R*(C–Cl) and Δ*R*(C–H) values are 0.72 and 0.16 Å, respectively, indicating a delay in the cleavage of the C–H compared to the cleavage of the C–Cl bond. The four products of the reaction formed two **H-b**s.

**Figure 10:**
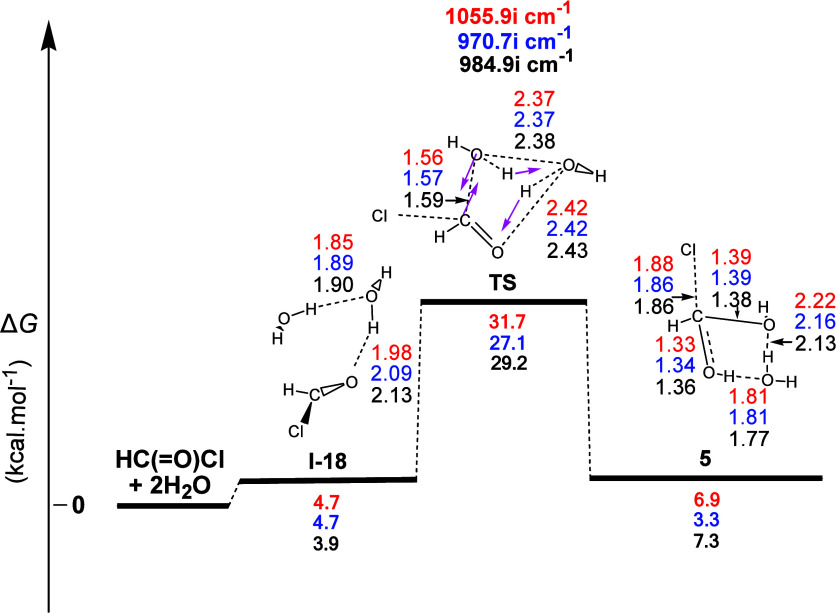
Gas-phase energy and geometry profile of diol (**5**) formation, derived from the **2** + 2Η_2_Ο interaction, along with selected structural data. For more details, see Figure 1 caption.

We then examined the possibility of the hydrolysis of **2** leading to formic acid and HCl. The corresponding gas-phase reaction profile (Figure 10) showed that the first product formed from the **2** + 2H_2_O interaction was chloromethanediol (**5**) with a slightly shorter C–O bond in one of the two hydroxyl groups and, correspondingly, a slight elongation of the C–Cl bond. The first step (**I-18**) corresponded to the formation of two **H-b**s, one between the two water molecules and the second between one of the water molecules and **2**. It actually involved activation of the carbonyl function through hydrogen bonding with one water molecule, with the latter activating the other water molecule also through hydrogen bonding to attack the carbonyl carbon. Accordingly, one water molecule offered a proton to the carbonyl oxygen and the other a hydroxy group to the carbonyl carbon. The normal coordinate vectors showed that the dominant motions involved the formation of a six-membered ring corresponding to (a) the attachment of an HO group of one water molecule on the C atom of **2**, leading to the simultaneous formation of the C–O bond, (b) the abstraction of the H atom of the latter water molecule by the other one, and (c) the abstraction of the H atom of the second water molecule by the O atom of **2**. Calculations also showed that this was (i) also a concerted process, (ii) a weakly endergonic reaction with calculated Δ*G* values equal to ca. 6.9, 3.3, and 7.3 kcal mol^–1^, and (iii) had calculated activation energy Δ*G*^≠^ values equal to ca. 31.7, 27.1, and 29.2 kcal mol^–1^. Therefore, this transformation could occur with mild heating. The calculated lengths of the C = O, O...H, H...O, O...H, H...O, and O...C partial bonds in the TS were 1.25, 1.45, 1.06, 1.19, 1.23, and 1.59 Å, respectively, with the corresponding Δ*R* values for the most important C=O (broken) and O–H and O–C (formed) bonds being 0.06, 0.48, and 0.21 Å, respectively, showing a rather asynchronous concerted process in which the formation of the new C–O bond advances compared to the C=O bond cleavage and the new O–H bond formation. Chloromethanediol (**5**) formed, incorporating two electron-donating hydroxyl groups and one very good leaving group (Cl) on the same C atom, should be expected to be unstable. In this case, however, stabilization is probably secured via the two **H-b**s with the remaining water molecule, forming a six-membered ring. This reaction can be considered as a water-assisted addition of water to the carbonyl group of formyl chloride.

**Figure 11:**
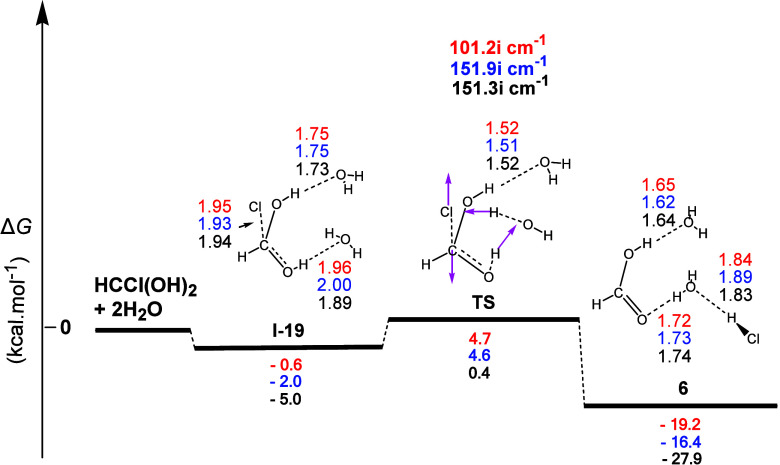
Gas-phase energy and geometry profile of formic acid (**6**) formation, derived from the **5** + 2Η_2_Ο interaction, along with selected structural data. For more details, see Figure 1 caption.

Chloromethanediol (**5**) formed can then proceed to formic acid (**6**), reacting with two H_2_O molecules. This reaction is shown in Figure 11. The first step (**I-19**) corresponded to the formation of two **H-b**s between **5** and two H_2_O molecules. Moreover, the normal coordinate vectors of the **TS** showed that the dominant motions involved the formation of a six-membered ring corresponding to (a) the detachment of the Cl atom from **5**, (b) the abstraction of the H atom of the water molecule H-bonded to one of the two HO functions of **5** by the departing Cl atom, leading to the simultaneous formation of the H–Cl bond, and (c) the abstraction of the H atom of the same OH group of **5** by the HO group of the same water molecule (regeneration of H_2_O). The other water molecule simply bonded to the second HO group of **5** through a **H**-**b**. The partial bond lengths of interest in the transition state were C...Cl (2.65 Å), Cl...H (1.80 Å), H...O (1.05 Å), O...H (1.51 Å), H...O (1.03 Å), and O...C (1.29 Å). The corresponding Δ*R* values for the most interesting C–Cl and H–O (broken) and O=C (formed) bonds were 0.87, 0.06, and 0.10 Å, respectively, thus showing an asynchronous concerted mechanism, with the O–H bond cleavage delaying in comparison to the C–Cl bond cleavage and the C–O formed being halfway to C=O double bond formation. Calculations also showed that this process was (i) an exergonic reaction with calculated Δ*G* values equal to ca. −19.2, −16.4, and −27.9 kcal mol^–1^ and (ii) had calculated activation energy Δ*G*^≠^ values equal to ca. 5.3, 6.6, and 5.4 kcal mol^–1^, indicating that this transformation can occur at room temperature. Finally, the formic acid formed joined the two water molecules through two **H-b**s, namely, with one of the water molecules additionally forming another **H-b** with the H atom of HCl.

**Figure 12:**
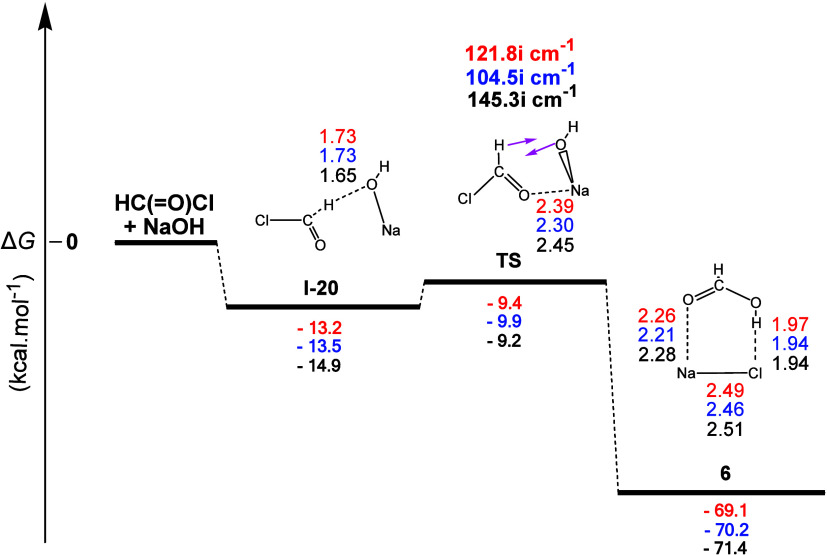
Gas-phase energy and geometry profile of formic acid (**6**) formation, derived from the **2** + NaΟΗ interaction, along with selected structural data. For more details, see Figure 1 caption.

The gas-phase reaction profile to form formic acid (**6**), derived from the **2** + NaOH interaction, is shown in Figure 12. The first step (**I-20**) corresponded to the formation of one strong **H-b** between the H atom of **2** and the O atom of NaOH, with simultaneous slight elongation of the C–H bond to 1.15 Å (from 1.11 Å). In the **TS** that followed, the length of C–Cl remained constant in relation to **I-20**, showing no participation of C–Cl in the dominant motions, and changed slightly (from 1.19 to 1.21 Å), of C=O, which is understood taking into consideration the main rather weak electrostatic interaction of the O atom of the hydroxide ion with the electropositive C atom of the carbonyl group, Δ*R*(C–O) = 1.13 Å. There is also a rather weak electrostatic interaction between the carbonyl O atom with Na^+^ with the length of this interaction being 2.39 Å. Τhe above, combined with the almost barrierless activation energy of the reaction with calculated Δ*G^≠^* values equal to ca. 3.8, 3.6, and 5.7 kcal mol^–1^, could account well for the conversion of **TS** to products through two very fast (barrierless) steps, namely, addition of the strong nucleophile hydroxide ion to the highly electron-deficient C atom of the carbonyl group, followed by elimination of the very good leaving group chloride ion from the thus formed tetrahedral electron-rich alkoxide intermediate. Calculations also showed that this process was a strong exergonic reaction with calculated Δ*G* values equal to ca. −69.1, −70.2, and −71.4 kcal mol^–1^. Finally, the formic acid formed joined the NaCl molecule through one electrostatic Na...O interaction and one H...Cl **H-b**.

**Figure 13:**
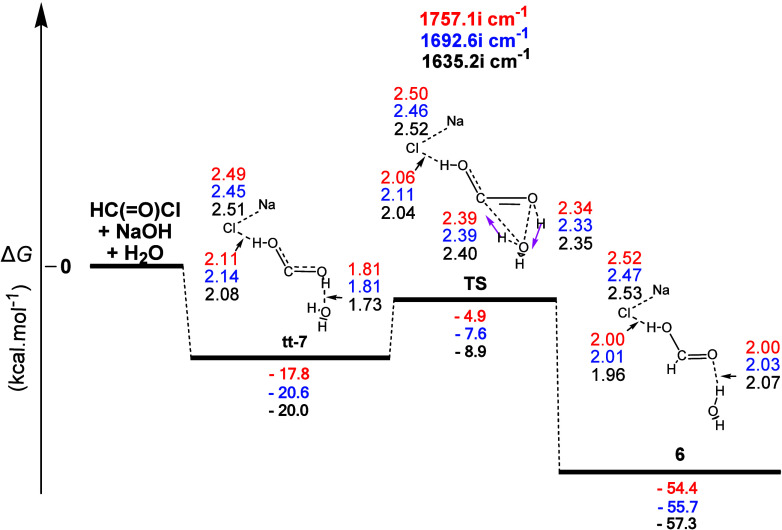
Gas-phase energy and geometry profile of formic acid (**6**) formation, derived from the **2** + NaΟΗ + H_2_O interaction, along with selected structural data. For more details, see Figure 1 caption.

The gas-phase reaction profile to form formic acid (**6**) derived from the **2** + NaOH + H_2_O interaction is shown in Figure 13. This was a quite different mechanistic scheme than the previous one. The first step (**tt-7**) led to the formation of two **H-b**s, of which the first was formed between an OH group of *s-trans, s-trans-*dihydroxycarbene (**tt**-**7**)^35^ and the O atom of the water molecule and the other between the second OH group of **tt**-**7** and the Cl atom of NaCl. Dihydroxycarbene, stabilized by the strong +M mesomeric effect of the two hydroxyl groups, could be envisaged to be formed by the nucleophilic attack of the hydroxide ion on the carbonyl carbon, followed by an internal proton abstraction by the intermediate tetrahedral alkoxide ion and chloride ion elimination. No energy barrier exists for this process. Τhe two C–O bonds of **tt-7** had almost identical lengths (1.34 and 1.32 Å), somewhat shorter than a C–O single bond, due to the aforementioned resonance effect. It should be noted here that a new conformer of dihydroxycarbene was recently isolated and studied experimentally and theoretically by Schreiner and co-workers.^35^ In particular, they isolated and studied the *s-cis, s-cis* (cc) conformer of dihydroxycarbene as the possible missing link in the reduction of CO_2_ with H_2_ to form *s-cis, s-trans* (ct)-dihydroxycarbene and subsequently formic acid. The same research group had also succeeded in synthesizing the ct- and the *s-trans, s-trans* (tt)-conformers via pyrolysis of oxalic acid.^36^ As can be seen from Figure 13, *s-trans, s-trans* (tt)-dihydroxycarbene (**tt-7**) was the intermediate leading to formic acid, except that here it was the product of the hydrolysis of formyl chloride in an alkaline (NaOH + H_2_O) environment. The **TS** that followed showed that the molecular mechanism was a concerted process. The normal coordinate vectors showed that the dominant motions involved the formation of a five-membered ring containing one C–O linkage (1.27 Å), with a high character of double bonds, and four other linkages with the following lengths: C...H (1.49 Å), H...O (1.16 Å), O...H (1.22 Å), and H...O (1.23 Å). The first one corresponded to the detachment of the H atom of the water molecule by the C atom of the diol and the second to the detachment of the H atom of the diol by the hydroxy group, thus reforming the water molecule. The Δ*R* values for the newly formed C–H bond and the broken H–O one of the HO group of the diol were 0.38 and 0.26 Å, respectively, thus representing an asynchronous concerted mechanism, with C–H bond formation delaying compared to the H–O bond cleavage. The lengths of the two C–O bonds now differ significantly, with the one outside of the ring retaining its length, with a dominant single bond character and becoming the C–OH bond of the product (formic acid). Calculations also showed that this process was (i) a concerted one, (ii) a strong exergonic reaction with calculated Δ*G* values equal to ca. −54.4, −55.7, and −57.3 kcal mol^–1^, and (iii) had calculated activation energy Δ*G^≠^* values equal to ca. 12.9, 13.0, and 11.1 kcal mol^–1^, indicating that this transformation could readily occur at room temperature. Finally, the formic acid formed joined the NaCl and H_2_O molecules through two **H-b**s. These calculations show that formyl chloride can be readily hydrolyzed to formic acid by the action of NaOH alone or the combination NaOH + H_2_O. Of course, formic acid thus produced further reacted with additional NaOH to form one of the two final products of the alkaline hydrolysis of chloroform, namely, sodium formate and of course ΝaCl. On the other hand, hydrolysis of formyl chloride to formic acid can also take place by water alone but with gentle heating.

**Figure 14:**
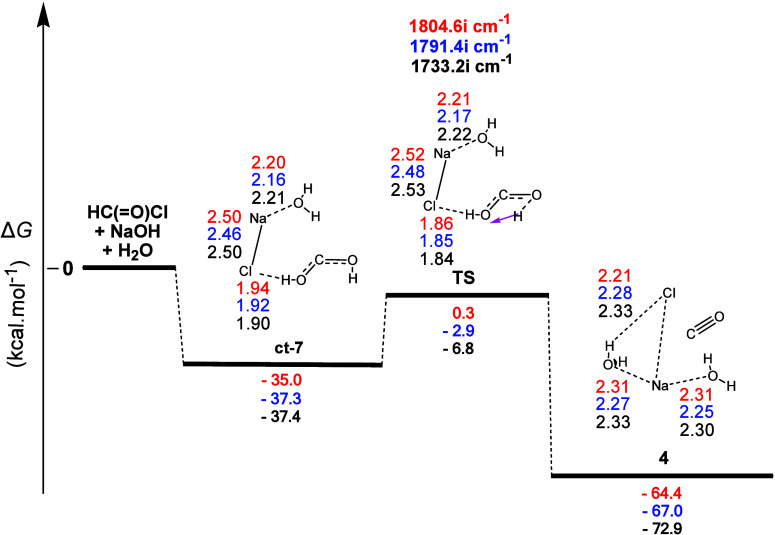
Gas-phase energy and geometry profile of carbon monoxide (**4**) formation, derived from the **2** + NaΟΗ + H_2_O interaction, along with selected structural data. For more details, see Figure 1 caption.

Figure 14 shows the result of our next attempt to further expand this very interesting **2** + NaOH + H_2_O interaction. Comparison with the reaction profile of Figure 13 revealed five very important differences, being (a) the much larger stabilization of **ct-7** of ca. 35.0, 37.3, and 37.4 kcal mol^–1^ almost twice as much as the previous **tt-7** one. This was probably because, unlike the previous intermediate which was stabilized by two **H-b**s, in the second one, we have one **H-b** and one electrostatic O...Na interaction, (b) the presence of the second **ct-7** conformer instead of the **tt-7** conformer in the counterpart of the previous one, (c) the much higher value of the activation energy in the second with calculated Δ*G^≠^* values equal to ca. 35.3, 34.4, and 30.6 kcal mol^–1^ which, however, could lead to a product, with heating, (d) the much higher exergonicity of the second with calculated Δ*G* values equal to ca. −64.4, −67.0, and −72.9 kcal mol^–1^, due to the presence of two Na...O and one Na...Cl electrostatic interactions and one H...Cl **H-b**, and (e) the different product resulted, CO, compared to the HCOOH in the first case, which, nevertheless, constituted one of the final products of CHCl_3_ hydrolysis. Nevertheless, both processes were concerted ones.

##### Conversion of Carbon Monoxide (**4**) to Formic Acid (**6**)

**Figure 15:**
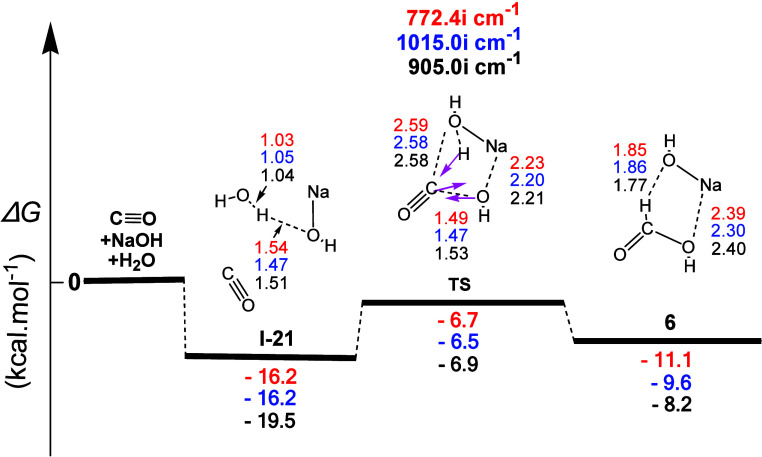
Gas-phase energy and geometry profile of formic acid (**6**) formation, derived from the **4** + NaΟΗ + H_2_O interaction, along with selected structural data. For more details, see Figure 1 caption.

The conversion of carbon monoxide (**4**) to formic acid (**6**) was the last part of the study. In particular, we were interested in investigating whether formic acid could be formed by the action of NaOH + H_2_O or only water on carbon monoxide. The gas-phase reaction profile to give formic acid (**6**), derived from the **4** + NaOH + H_2_O interaction, is shown in Figure 15. The first step (**I-21**) corresponded to the formation of one **H-b** between the H atom of H_2_O and the O atom of NaOH. The **TS** that followed showed that the molecular mechanism was a concerted process. The normal coordinate vectors revealed that the dominant motions involved the formation of a five-membered ring incorporating the C...OH (1.53 Å), C...H (1.40 Å), and H...O (1.22 Å) linkages, which corresponded to the attachment of the HO^–^ group of NaOH to the C atom of **4**, leading to the simultaneous formation of the C–O bond, whereas the C atom attached a proton from the water molecule, thus regenerating NaOH. The Δ*R* values for the new C–O and the C–H bonds were 0.15 and 0.29 Å, respectively. This reaction could be coined as NaOH-catalyzed water addition on carbon monoxide, and it was asynchronous with the C–O formation preceding the C–H formation. Calculations also showed that this process (i) was a weakly exergonic reaction with calculated Δ*G* values equal to ca. −11.1, −9.6, and −8.2 kcal mol^–1^ and (ii) had calculated activation energy Δ*G^≠^* values equal to ca. 9.5, 9.7, and 12.6 kcal mol^–1^, indicating that this transformation could occur at room temperature. The formic acid formed joined the NaOH molecule through one electrostatic Na...O interaction and one H...O **H-b**.

**Figure 16:**
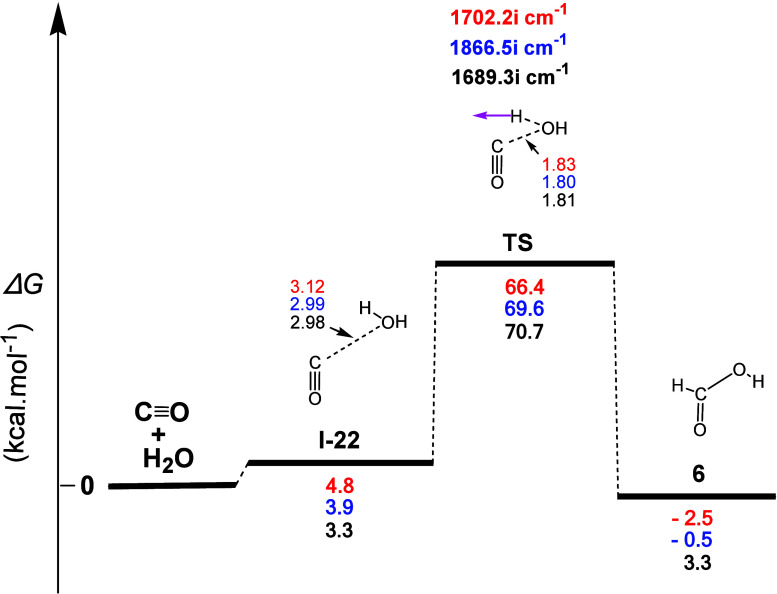
Gas-phase energy and geometry profile of formic acid (**6**) formation, derived from the **4** + H_2_O interaction, along with selected structural data. For more details, see Figure 1 caption.

The gas-phase reaction profile to form formic acid (**6**), derived from the **4** + H_2_O interaction, is shown in Figure 16. The first step (**I-22**) corresponded to the formation of a weak C...O electrostatic interaction between the C atom of CO and the O atom of H_2_O. The normal coordinate vectors of the **TS** showed that the dominant motions corresponded to the abstraction of the H atom of H_2_O by the C atom of **4**, followed by the nucleophilic attack of the OH group of H_2_O on the C atom of **4**, leading to the formation of the C–O bond of **6**. The calculated C...H, H...O, and C–O bond lengths were 1.17, 1.40, and 1.81 Å, respectively, and the corresponding Δ*R*(C–H), Δ*R*(H–O), and Δ*R*(C–O) values were 0.07, 0.43, and 0.43 Å, respectively, representing an asynchronous three-membered cyclic concerted mechanism, in which the formation of the C–O bond is significantly delayed compared to the formation of the C–H bond. Calculations also showed that this process was (i) a concerted one, (ii) a rather very weak exergonic reaction with calculated Δ*G* values equal to ca. −2.5, −0.5, and 3.3 kcal mol^–1^, and (iii) had calculated activation energy Δ*G*^≠^ values equal to ca. 66.4, 69.6, and 70.7 kcal mol^–1^, implying that this reaction was not likely to occur.

The gas-phase reaction profile to form formic acid (**6**), derived from the **4** + 2H_2_O interaction shown in Figure S7, was quite analogous to the previous one. Their most characteristic difference concerned the shape of the transition states, with the present one being a five-membered one. The normal coordinate vectors of the TS of Figure S7 corresponded to (a) the abstraction of a proton from the first water molecule by the C atom of CO, followed (b) by the abstraction of a proton from the second water molecule by the OH group of the first one, regenerating thus the first water molecule, and (c) the attack of the HO group of the second water molecule on the C atom of CO, thus resulting in the formation of the C–O bond. The lengths of the linkages in the TS structure were as follows: C...H (1.37 Å), H...OH (1.24 Å), HO...H (1.14 Å), H...OH (1.29 Å), and HO...C (1.80 Å). The Δ*R* values for the most significant C–H and C–O bonds were 0.26 and 0.42 Å, respectively, showing that also in this case, formation of the C–O was delayed compared to the formation of the C–H bond. The Δ*R* values for the other H–O broken (0.27 and 0.32 Å) or formed (0.17 Å) bonds in the TS pointed to an asynchronous five-membered cyclic concerted mechanism. Regarding the rest of their characteristics, the former was slightly exergonic and the present weakly endergonic. Furthermore, both had high activation energy values, which precluded the possibility of either occurring. Interestingly, the activation energy of TS for the present interaction was lower, with calculated Δ*G^≠^* values equal to ca. 53.7, 53.6, and 54.4 kcal mol^–1^ than the former but not lower enough to make it take place at ambient temperature. Of course, these results are in accord with classical considerations of organic chemistry excluding the ability of the weak nucleophile water to attack the electronegative carbon atom of carbon monoxide.

It is worth mentioning that coordination chemistry reactions in progress verified that syntheses at room temperature did not release chlorides in the reaction systems, in accordance with the theoretical predictions.

## Conclusions

**Scheme 4:**
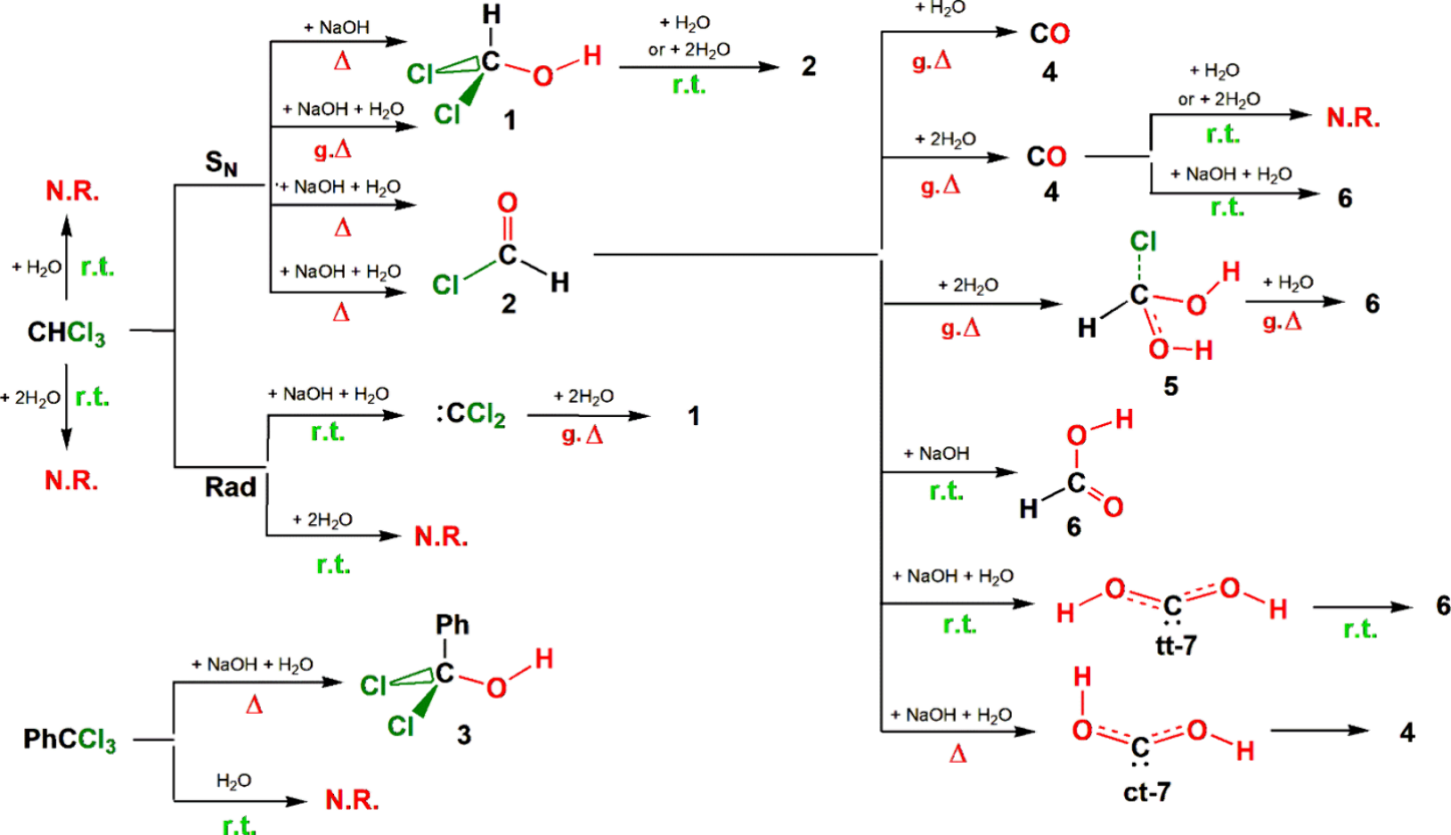
Outline of the Reactions Theoretically Studied in the Present Work Apart from the N.R. cases, all others may also produce NaCl or HCl and/or H_2_O, not shown for clarity. N.R. = no reaction (Δ*G^≠^* > 50 kcal mol^–1^); Δ = heating; g.Δ = gentle heating (Δ*G*^≠^ up to ca. 25 kcal mol^–1^); r.t. = room temperature (Δ*G*^≠^ up to ca. 19 kcal mol^–1^).

Coordination chemistry-related experiments have shown that when chloroform is used as the solvent, chloride ions are often incorporated in the isolated complexes, under basic or neutral conditions at room temperature (rt) or heating, respectively, with the only potential source of them being the solvent. Using ab initio and DFT calculations, we performed a complete study of the hydrolysis of chloroform aiming to identify the pathways leading to its known hydrolysis products, namely, CO, formic acid or sodium formate, and HCl or NaCl. Accordingly, 20 reaction profiles were studied of the interaction of CHCl_3_ with NaOH or NaOH + H_2_O and/or one or two H_2_O molecules, usually present in the experiments in the solvent or the inorganic starting materials. From these studies, the following conclusions could be drawn:

1. All three theoretical levels used showed nearly the same bond lengths for each distinct bond type presented, and this was the case with all reaction profiles studied. Moreover, the energy profile was basically the same for all three methods we employed, i.e., the energy data calculated either with the MP2 or with the DFT methods differed marginally and they converged to the same result. Hence, all three theoretical methods yielded, at least qualitatively, an almost identical energy profile for each reaction studied.
2. Hydrolysis of CHCl_3_ at room temperature can be only initiated by a strong base, e.g., NaOH, following the radical mechanism, an E1cB-type α-elimination, with generation of the active intermediate dichlorocarbene. This then undergoes a direct insertion reaction in one of the H–O bonds of water leading to the first intermediate, namely, dichloromethanol (**1**). The latter intermediate undergoes a water (one or two molecules)-assisted decomposition to formyl chloride (**2**) and HCl, resembling E2-type *syn* elimination reactions with the proton removed from O instead of a C atom. Formyl chloride (**2**) in turn also undergoes a water-assisted decomposition to CO (**4**) and HCl and hydrolyses with water, through the chloromethanediol (**5**) intermediate, providing formic acid (**6**) and HCl. This reaction and the following ones are summarized in Scheme 4. All reactions with water are concerted processes with cyclic transition states, are characterized by rather low activation energies (Δ*G^≠^* of ca. 12.7–27.0 kcal mol^–1^), and thus can be performed at ambient temperature or with gentle heating. NaOH, in addition to initiating hydrolysis, serves in the reaction to neutralize the acids (HCl and HCO_2_H) produced to the corresponding chloride and formate sodium salts.
3. Water alone does not react at room temperature with CHCl_3_ through any of the available mechanisms, substitution nucleophilic (S_N_) and radical, as they are all characterized by high energy barriers (Δ*G^≠^* > 50 kcal mol^–1^). With heating, however, water (one or two molecules) can react with CHCl_3_, through a hydrogen bond-assisted S_N_2-type mechanism, with either front- or backside attack of the nucleophile (H_2_O), or a two-step radical mechanism leading also to dichloromethanol (**1**).
4. CHCl_3_ undergoes a classical S_N_2-type reaction with NaOH (Δ*G^≠^* of ca. 36–40 kcal mol^–1^) on heating, but the corresponding interaction also involving one H_2_O molecule (Δ*G^≠^* of ca. 27–36 kcal mol^–1^) can take place with only gentle heating. Interestingly, in this case, the attacking nucleophile is the water molecule activated by the hydroxide ion.
5. On the other hand, the triad CHCl_3_ + NaOH + H_2_O can lead directly to formyl chloride (**2**) upon heating (Δ*G^≠^* of ca. 34–41 kcal mol^–1^), through S_N_2 mechanisms, leading obviously to intermediate **1,** which in a second barrierless step ends up in **2**.
6. Benzotrichloride presents a behavior similar to CHCl_3_, lacking the α–Η of chloroform and therefore not amenable to the radical mechanism. It cannot react with water to form dichloro(phenyl)methanol (**3**) at room temperature presenting a high energy barrier (Δ*G^≠^* of ca. 45 kcal mol^–1^), but it does so on heating, through an unexpected two-step procedure involving migration of a Cl atom to the *o*-position of the ring, followed by an S_N_1′-like reaction (allylic rearrangement). On the contrary, with NaOH and H_2_O, benzotrichloride undergoes an S_N_2-type reaction with mild heating (Δ*G*^≠^ of ca. 32 kcal mol^–1^) producing alcohol **3.**
7. Formyl chloride (**2**) readily reacts with NaOH at room temperature providing formic acid (**6**), obviously through a two-step barrierless process, involving hydroxide ion addition on the carbonyl function followed by chloride ion elimination. On the other hand, the interaction of **2** + NaOH + H_2_O, depending on whether the reaction takes place at room temperature or with mild heating (Δ*G^≠^* of ca. 35 kcal mol^–1^), leads to either formic acid (**6**) or carbon monoxide (**4**).
8. Interestingly, in the former case, the intermediate is *s-trans, s-trans*-dihydroxycarbene (**tt-7**) and in the latter case *s-cis, s-trans*-dihydroxycarbene (**ct-7**), which both have been identified also as key intermediates in other types of reactions.
9. Finally, although water alone cannot add to carbon monoxide (**4**) at room temperature to give formic acid (**6**) due to the high energy barriers (Δ*G^≠^* > 50 kcal mol^–1^) involved, it does so in the presence of NaOH. Therefore, the final product of the basic hydrolysis of CHCl_3_ sodium formate can be formed from either formyl chloride directly or carbon monoxide or both.

A final chemical message of the above conclusions is that the mechanistic ideas developed in this work can be useful for the synthetic inorganic chemist. For example, a safe way to avoid chloride ions (and hence chloro ligands) when performing reactions in CHCl_3_ is to use it at room temperature, even in the presence of water. On the other hand, addition of NaOH with limited amount of water under gentle heating could release Cl^–^ ions from CHCl_3_, thus avoiding the use of other chloride sources (e.g., metal chlorides) and different solvents, which might direct a given reaction to undesirable pathways.

## Computational Details

All stationary points, located on the potential energy surfaces, were fully optimized using the ab initio MP2 level of theory^37^ and the Dunning’s cc-pVDZ basis set^38,39^ for all atoms. Higher level DFT calculations, using the MP2-optimized geometries, were performed to obtain more accurate stabilization and activation energies. Two different DFT functionals were used, namely, the long-range corrected ωB97XD,^40−43^ which includes empirical dispersion, and the Minnesota global hybrid M06-2X^44^ both with the Dunning’s cc-pVTZ basis set for all atoms, to account for the long-range interactions of the systems under study. All ab initio and DFT calculations were performed using the Gaussian 09 suite of programs (version B.01).^45^ Analytical frequencies were calculated at the same level of theory, and the nature of the stationary points was determined in each case according to the number of negative eigenvalues of the Hessian matrix (i.e., no imaginary frequency for local and global minima and one imaginary frequency for transition states). Gibbs free energies were used to construct the energetic reaction profiles and the sum of electronic and ZPE for the relative stability of intermediate structures, where needed. To confirm that the located TSs on the reaction profiles connect to the desired minima on both sides, IRC^46^ calculations were carried out. All energies reported throughout the text are given in kcal mol^–1^, and the bond lengths are given in angstroms (Å).

## Data Availability

The data underlying this study are available in the published article and its Supporting Information.

## Supporting Information Available

Additional reaction profiles, structure of the transition state TS3 in the interaction CHCl_3_ + NaOH + H_2_O (related to Figure 4), gas-phase Cartesian coordinates of all reactants and products along with all corresponding calculated energy values, and gas-phase Cartesian coordination of all stationary points along with all corresponding calculated energy values (PDF)
