# Supplementary material for: “Marriage” of Inorganic to Organic Chemistry as Motivation for a Theoretical Study of Chloroform Hydrolysis Mechanisms
Source: J Org Chem. 2024 Sep 20;89(19):13894–912. doi: 10.1021/acs.joc.4c00942 (PMC11459432; doi:10.1021/acs.joc.4c00942)

Supporting Information

"Marriage" of Inorganic to Organic Chemistry as Motivation for a Theoretical Study of Chloroform Hydrolysis Mechanisms

Christina Stamou[a], Spyros P. Perlepes[a], Michail M. Sigalas,[b] Dionissios Papaioannou[a*], Athanassios C. Tsipis [c] and Evangelos G. Bakalbassis[d*]

[a] Department of Chemistry, University of Patras, Patras 26504, Greece

[b] Department of Materials Science, University of Patras, Patras 26504, Greece

[c] Lab. of Inorganic Chemistry, Chemistry Department, University of Ioannina, Greece

[d] Aristotle University of Thessaloniki, University Campus, Thessaloniki 54124, Greece

Table of Contents

Contents	Page
Figures S1-S7	S2-S8
Calculations at the MP2/cc-pVDZ level	S9
**Table S1**. Gas-phase Cartesian coordinates of all Reactants and Products along with all corresponding calculated Energy values at the MP2/cc-pVDZ level	S10-S13
**Table S2.** Gas-phase Cartesian coordinates of all stationary points along with all corresponding calculated Energy values and the IRC plots found in the Potential Energy Surfaces shown in the Figures.	S14-S64
Calculations at the M06-2X/cc-pVTZ level	S65
**Table S3**. Gas-phase Cartesian coordinates of all Reactants and Products along with all corresponding calculated Energy values at the M06-2X/c-pVTZ level.	S66-S69
**Table S4.** Gas-phase Cartesian coordinates of all stationary points along with all corresponding calculated Energy values and the IRC plots found in the Potential Energy Surfaces shown in the Figures.	S70-S122
Calculations at the ωB97XD/cc-pVTZ level	S123
**Table S5**. Gas-phase Cartesian coordinates of all Reactants and Products along with all corresponding calculated Energy values at the ωB97XD/cc-pVTZ level.	S124-S127
**Table S6.** Gas-phase Cartesian coordinates of all stationary points along with all corresponding calculated Energy values and the IRC plots found in the Potential Energy Surfaces shown in the Figures.	S128-S179

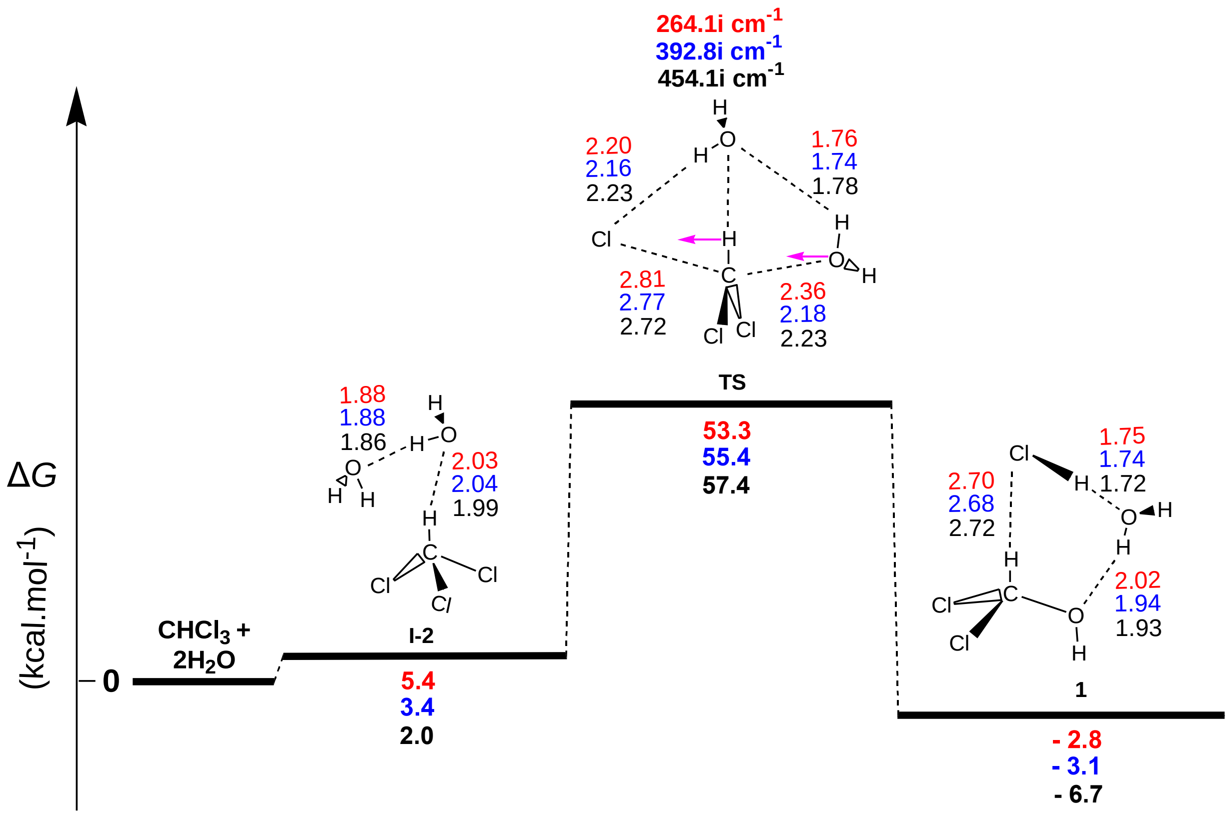

Figure S1. Gas-phase energy and geometry profile of intermediate **1** formation, derived from the $CHCl_3 + 2H_2O$ interaction, along with selected structural data. For more details see Figure 1 caption.

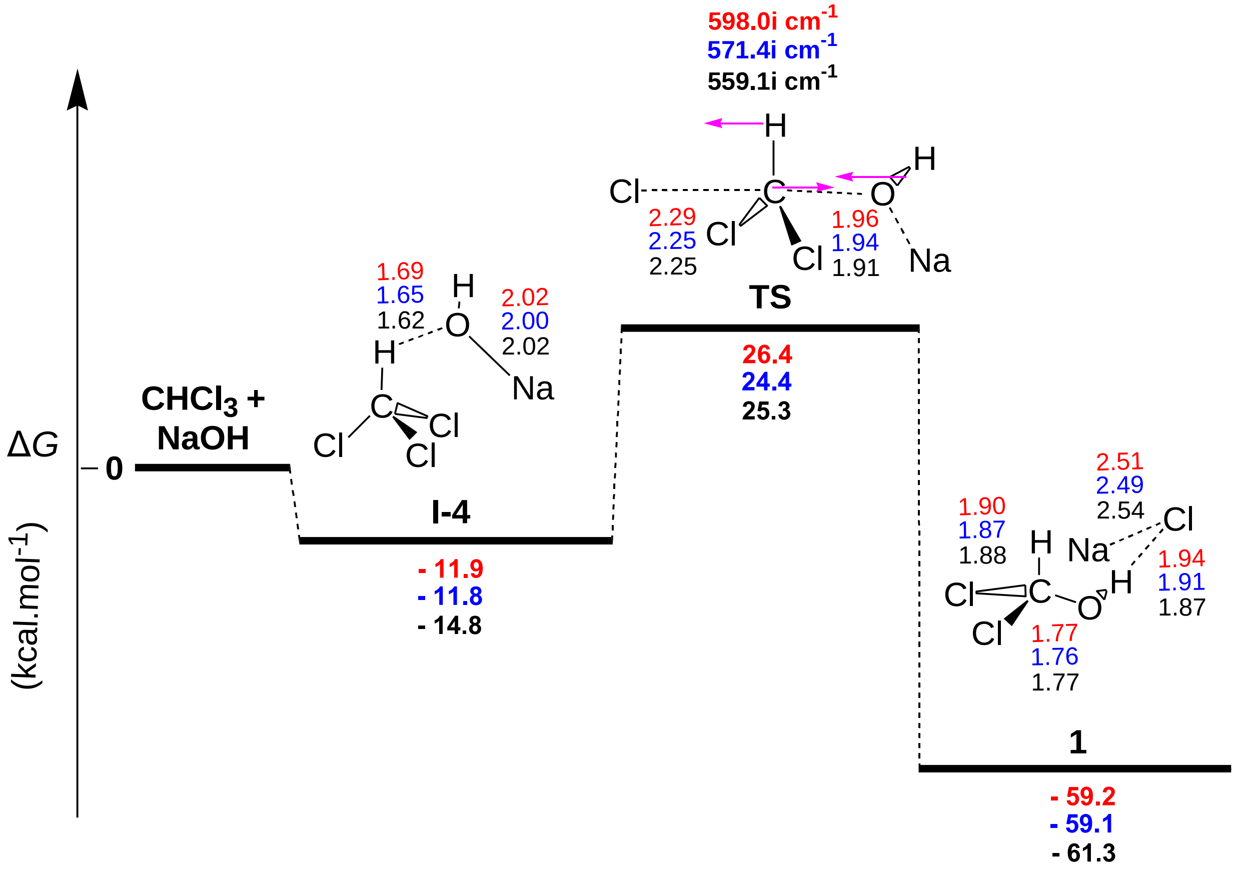

Figure S2. Gas-phase energy and geometry profile of intermediate **1** formation, derived from the $CHCl_3$ + NaOH interaction, along with selected structural data. For more details see Figure 1 caption.

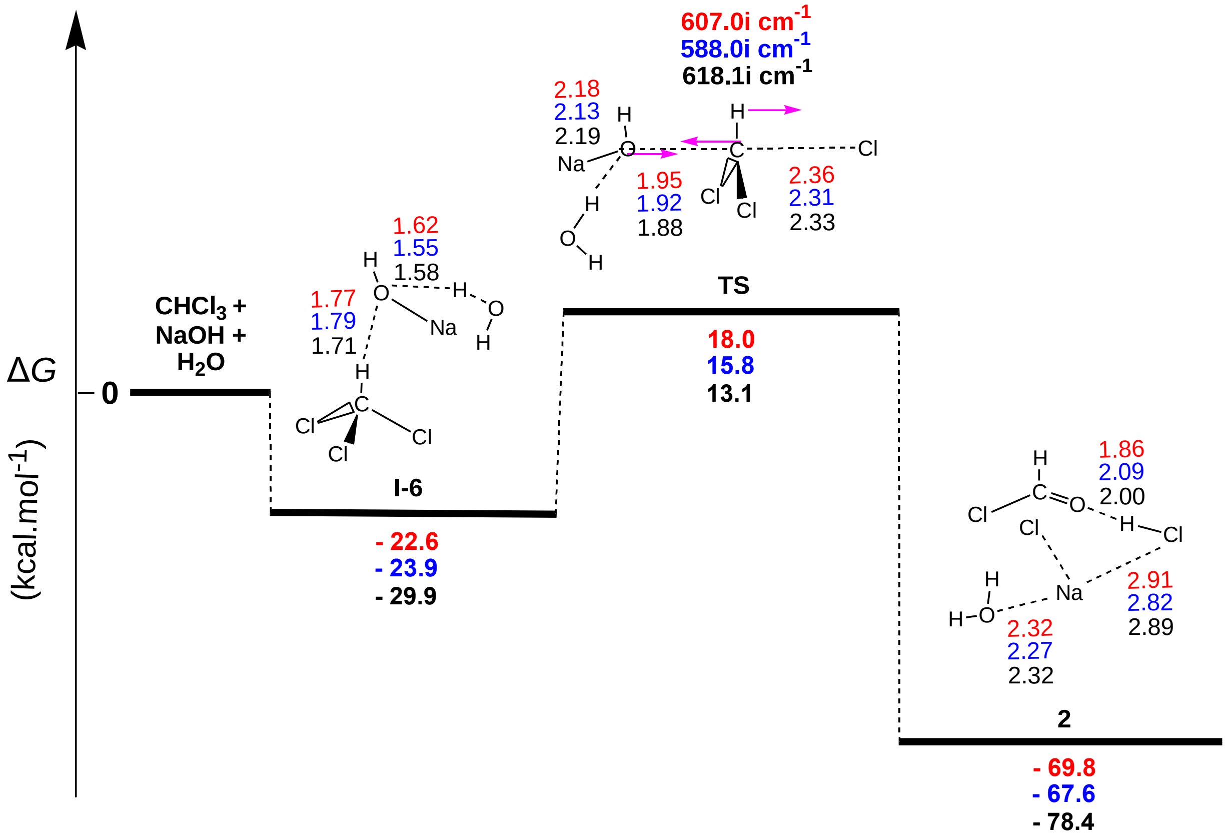

Figure S3. Gas-phase energy and geometry profile of intermediate **2** formation, derived from the $CHCl_3$ + NaOH + H_2O interaction, along with selected structural data. For more details see Figure 1 caption.

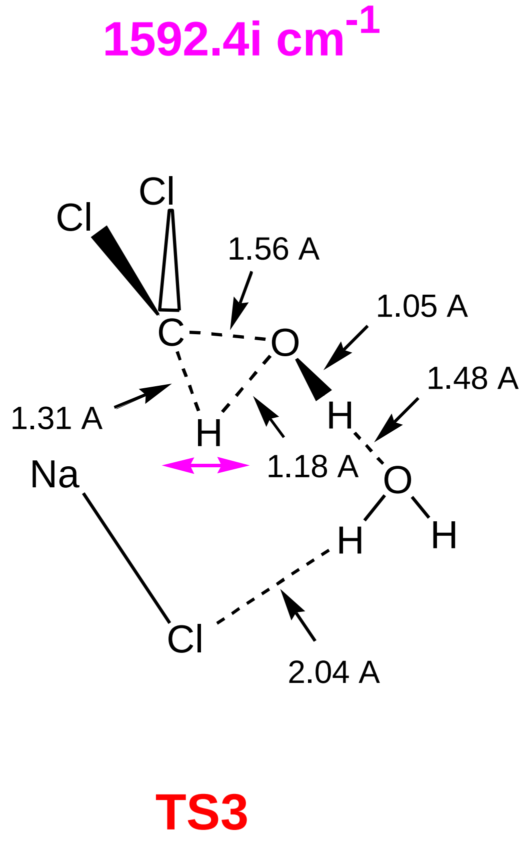

Figure S4. Detailed structure of TS3 in the interaction of $CHCl_3$ + NaOH + H_2O of Figure 4, calculated at the MP2/cc-pVDZ level.

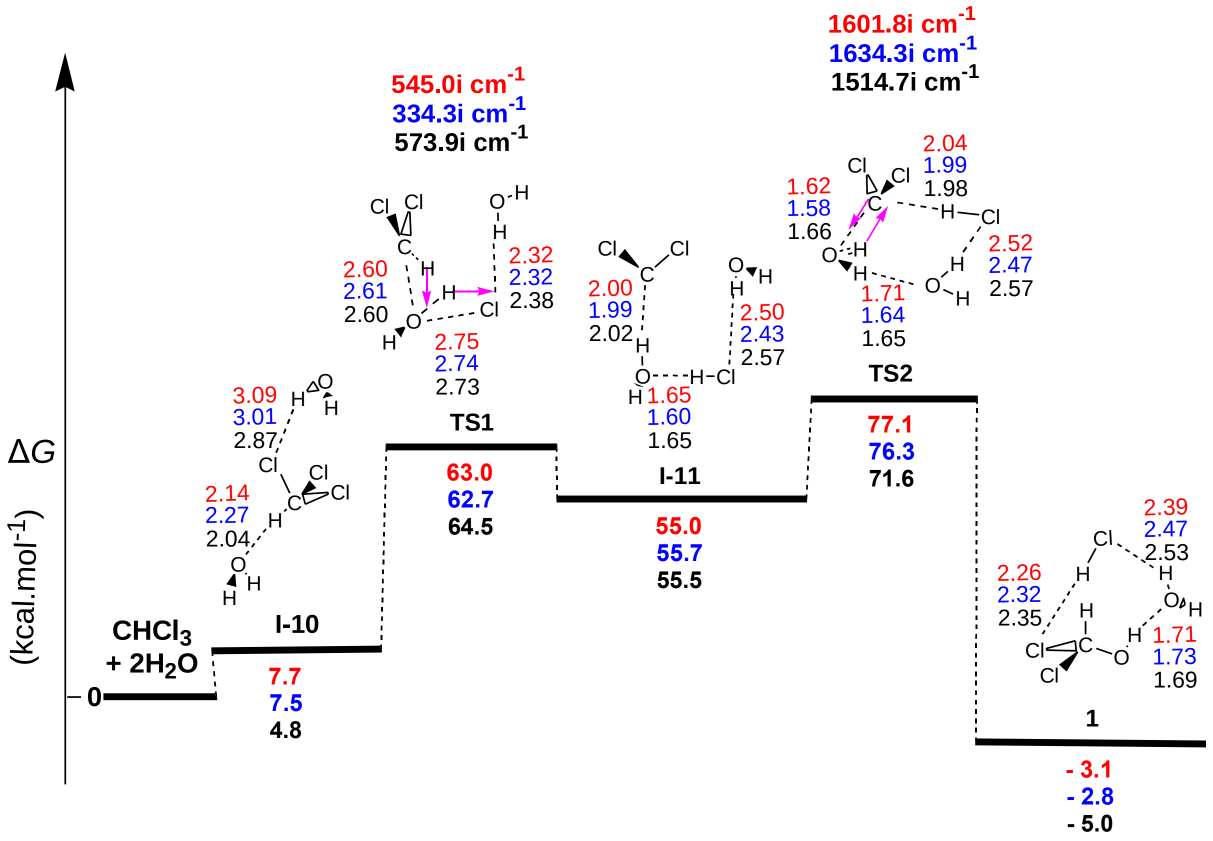

Figure S5. Gas-phase energy and geometry profile of intermediate **1** formation, derived from the $CHCl_3$ + $2H_2O$ interaction, *via* the radical mechanism, along with selected structural data. For more details see Figure 1 caption.

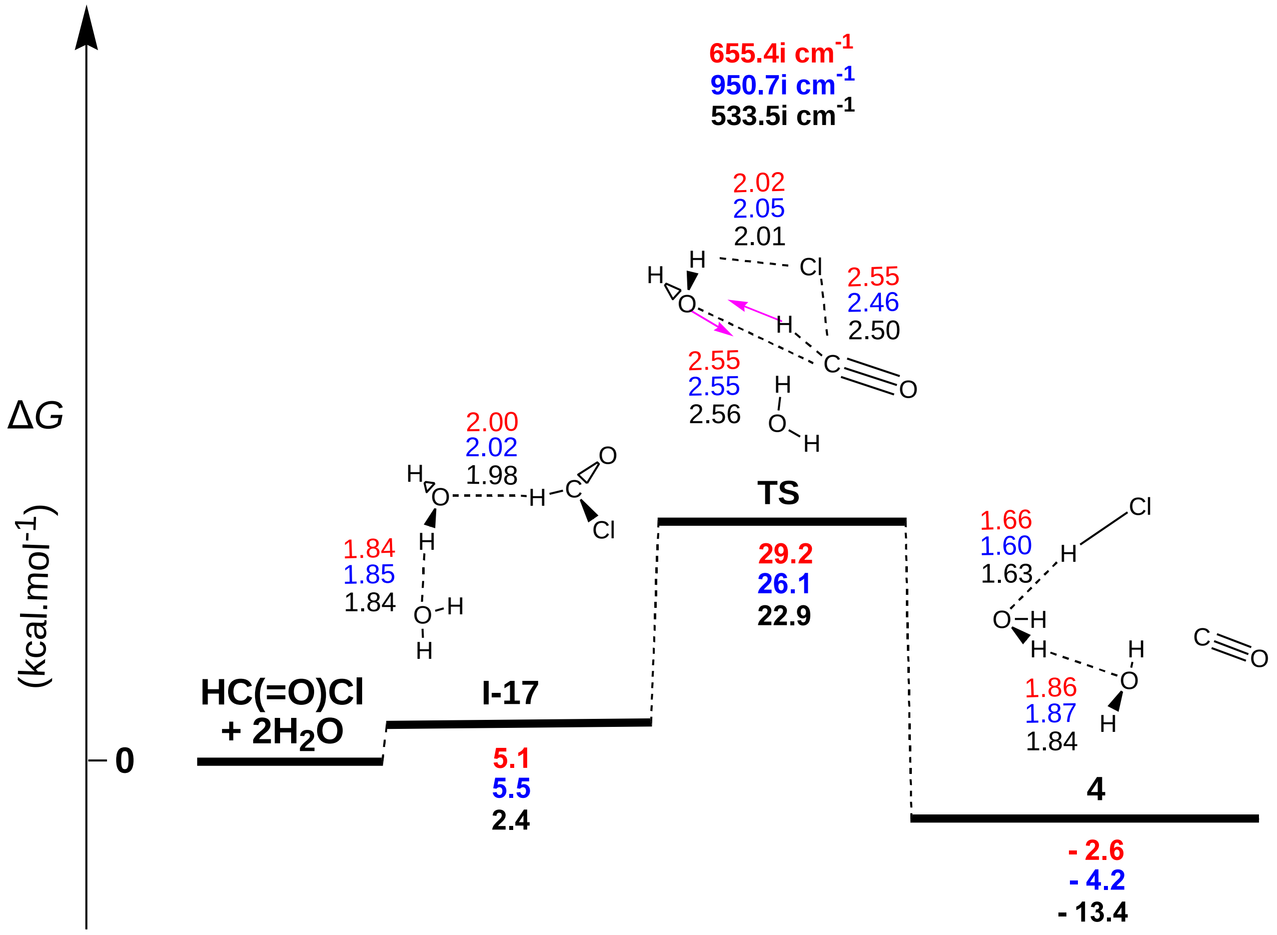

Figure S6. Gas-phase energy and geometry profile of the carbon monoxide, **4** formation, derived from the **2** + $2H_2O$ interaction, along with selected structural data. For more details see Figure 1 caption.

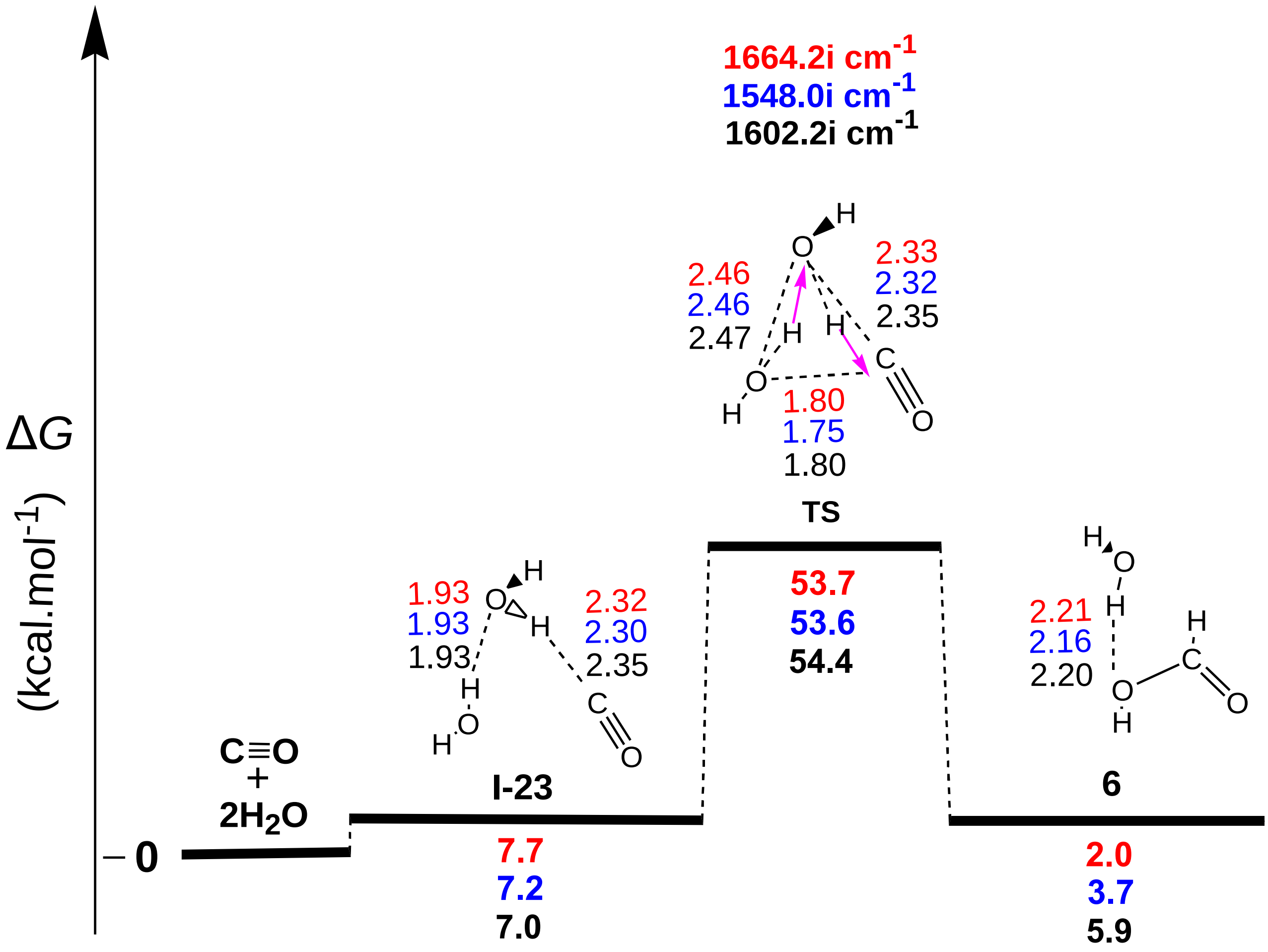

Figure S7. Gas-phase energy and geometry profile of the formic acid, **6** formation, derived from the **4** + $2H_2O$ interaction, along with selected structural data. For more details see Figure 1 caption.

Calculations at the MP2/cc-pVDZ level

Table S1. Gas-phase Cartesian coordinates of all Reactants and Products along with all corresponding calculated Energy values at the MP2/cc-pVDZ level.

A-Reactants

$CHCl_3$

C	-2.59860800	-2.63827200	0.02598700
H	-2.23326000	-2.12136400	-0.86911200
Cl	-1.98207000	-1.76623400	1.44540700
Cl	-4.37409200	-2.61199600	-0.01945800
Cl	-1.98211500	-4.30359800	-0.01944100

Zero-point correction= 0.020324 (Hartree/Particle)
Thermal correction to Energy= 0.024709
Thermal correction to Enthalpy= 0.025653
Thermal correction to Gibbs Free Energy= -0.008822
Sum of electronic and zero-point Energies= -1417.468099
Sum of electronic and thermal Energies= -1417.463714
Sum of electronic and thermal Enthalpies= -1417.462769
Sum of electronic and thermal Free Energies= -1417.497245

NaOH

H	-1.97668200	-2.24763900	-0.65057300
Na	-4.71499600	-2.71481900	0.15866600
O	-2.88573400	-2.40283500	-0.38174500

Zero-point correction= 0.010960 (Hartree/Particle)
Thermal correction to Energy= 0.014452
Thermal correction to Enthalpy= 0.015396
Thermal correction to Gibbs Free Energy= -0.004498
Sum of electronic and zero-point Energies= -237.500107
Sum of electronic and thermal Energies= -237.496616
Sum of electronic and thermal Enthalpies= -237.495671
Sum of electronic and thermal Free Energies= -237.515565

H_2O

H	-2.30597600	-2.15128400	-0.81745700
O	-2.54868300	-2.61813600	-0.00884100
H	-3.51275300	-2.59588800	-0.04735400

Zero-point correction= 0.021641 (Hartree/Particle)
Thermal correction to Energy= 0.024476
Thermal correction to Enthalpy= 0.025420
Thermal correction to Gibbs Free Energy= 0.003325
Sum of electronic and zero-point Energies= -76.207025

Sum of electronic and thermal Energies= -76.204190
Sum of electronic and thermal Enthalpies= -76.203246
Sum of electronic and thermal Free Energies= -76.225341

$PhCCl_3$

C	-1.84100900	-0.52555200	-0.11690300
Cl	-0.87142900	-2.02454600	-0.22973600
Cl	-3.13845700	-0.60827700	-1.36205800
C	-1.02424300	0.73881200	-0.32001400
C	0.35831300	0.72105400	-0.57241200
C	1.05340900	1.93032300	-0.75106300
C	0.37937500	3.15819900	-0.67982400
C	-1.00444300	3.17396400	-0.42710500
C	-1.70401200	1.97411800	-0.24804500
H	0.89702300	-0.22645800	-0.63076800
H	2.13022900	1.90464700	-0.94714900
H	0.92538500	4.09667200	-0.81968800
H	-1.54276900	4.12555700	-0.36895500
H	-2.78009300	1.99290600	-0.05180100
Cl	-2.61482400	-0.48565500	1.50795300

Zero-point correction= 0.100190 (Hartree/Particle)
Thermal correction to Energy= 0.109358
Thermal correction to Enthalpy= 0.110302
Thermal correction to Gibbs Free Energy= 0.063917
Sum of electronic and zero-point Energies= -1647.728034
Sum of electronic and thermal Energies= -1647.718866
Sum of electronic and thermal Enthalpies= -1647.717922
Sum of electronic and thermal Free Energies= -1647.764307

B-Products

$CH(OH)Cl_2$ (1)

C	0.18053500	0.11712900	0.67440800
H	-0.30082500	-0.48635200	-0.10316500
Cl	1.50129200	-0.88146500	1.38796000
Cl	-1.05562200	0.43753800	1.94717400
O	0.64685100	1.26356200	0.10251600
H	1.06902600	1.78366500	0.80670500

Zero-point correction= 0.034597 (Hartree/Particle)
Thermal correction to Energy= 0.038997
Thermal correction to Enthalpy= 0.039941
Thermal correction to Gibbs Free Energy= 0.006269
Sum of electronic and zero-point Energies= -1033.462432
Sum of electronic and thermal Energies= -1033.458033
Sum of electronic and thermal Enthalpies= -1033.457088

Sum of electronic and thermal Free Energies= -1033.490761

HCOCl (2)

C	-0.85664900	-1.21293100	-0.25743800
H	-0.70913900	-0.26618400	-0.81239800
Cl	0.46692000	-1.41697300	0.92108700
O	-1.74748900	-1.99525300	-0.39962600

Zero-point correction= 0.019236 (Hartree/Particle)
Thermal correction to Energy= 0.022473
Thermal correction to Enthalpy= 0.023417
Thermal correction to Gibbs Free Energy= -0.005987
Sum of electronic and zero-point Energies= -573.245147
Sum of electronic and thermal Energies= -573.241910
Sum of electronic and thermal Enthalpies= -573.240966
Sum of electronic and thermal Free Energies= -573.270370

CO (4)

C	-0.81070100	-1.15792500	-0.26047900
O	-1.65067500	-1.93168800	-0.36898500

Zero-point correction= 0.004816 (Hartree/Particle)
Thermal correction to Energy= 0.007176
Thermal correction to Enthalpy= 0.008121
Thermal correction to Gibbs Free Energy= -0.014338
Sum of electronic and zero-point Energies= -113.031991
Sum of electronic and thermal Energies= -113.029630
Sum of electronic and thermal Enthalpies= -113.028686
Sum of electronic and thermal Free Energies= -113.051145

$HCCl(OH)_2$ (5)

C	-0.89110500	-0.83949100	0.28326500
H	-1.43561900	-1.72121300	0.63814200
Cl	0.77595300	-0.89904400	1.05928400
O	-0.76400400	-0.95161300	-1.08411800
O	-1.56595700	0.27456100	0.73305600
H	-0.14864900	-0.26191400	-1.37847400
H	-1.00483200	1.04646900	0.55921600

Zero-point correction= 0.048308 (Hartree/Particle)
Thermal correction to Energy= 0.053020
Thermal correction to Enthalpy= 0.053964
Thermal correction to Gibbs Free Energy= 0.020460
Sum of electronic and zero-point Energies= -649.452755
Sum of electronic and thermal Energies= -649.448043

Sum of electronic and thermal Enthalpies= -649.447099
Sum of electronic and thermal Free Energies= -649.480602

HCOOH (6)

C	0.26065400	0.10806800	0.74313300
H	-0.30700000	-0.55387000	0.05994700
O	0.57881400	1.24671600	0.09058900
H	1.07222100	1.78128000	0.74056300
O	0.55101500	-0.14455500	1.88951300

Zero-point correction= 0.034235 (Hartree/Particle)
Thermal correction to Energy= 0.037389
Thermal correction to Enthalpy= 0.038333
Thermal correction to Gibbs Free Energy= 0.010147
Sum of electronic and zero-point Energies= -189.247080
Sum of electronic and thermal Energies= -189.243927
Sum of electronic and thermal Enthalpies= -189.242983
Sum of electronic and thermal Free Energies= -189.271169

Table S2. Gas-phase Cartesian coordinates of all stationary points along with corresponding calculated Energy values and the IRC plots found in the PES shown in the Figures.

Figure-1

Intermediate (I-1)

C	0.20911400	0.05177300	0.13767100
H	0.20449800	-1.04393000	0.13406300
Cl	1.67177000	0.64625100	0.94151600
Cl	0.15205300	0.58012100	-1.56397600
Cl	-1.25789100	0.57753800	1.00340000
O	-0.99864300	-2.70061400	-0.52843000
H	-1.11128600	-2.42337400	-1.44809600
H	-1.83555200	-2.42491600	-0.12979100

Zero-point correction= 0.043963 (Hartree/Particle)

Thermal correction to Energy= 0.052431

Thermal correction to Enthalpy= 0.053375

Thermal correction to Gibbs Free Energy= 0.007720

Sum of electronic and zero-point Energies= -1493.683891

Sum of electronic and thermal Energies= -1493.675422

Sum of electronic and thermal Enthalpies= -1493.674478

Sum of electronic and thermal Free Energies= -1493.720133

Transition State

C	-0.71728000	0.84997900	0.42334000
H	-1.10952500	1.03356200	-1.11127000
Cl	0.86938300	0.43678100	0.89780200
Cl	-1.46897900	0.89091500	-2.46129200
Cl	-1.74748100	1.14836900	1.76777700
O	-1.41932700	-1.25276600	-0.11389900
H	-1.33887400	-1.09271400	-1.07227400
H	-2.38232500	-1.23859300	0.00221400

Zero-point correction= 0.038262 (Hartree/Particle)

Thermal correction to Energy= 0.046007

Thermal correction to Enthalpy= 0.046951

Thermal correction to Gibbs Free Energy= 0.003926

Sum of electronic and zero-point Energies= -1493.599155

Sum of electronic and thermal Energies= -1493.591410

Sum of electronic and thermal Enthalpies= -1493.590466

Sum of electronic and thermal Free Energies= -1493.633491

Product (1)

C	0.17386200	-0.05546200	0.48352100
H	0.57176400	0.65373700	-0.25141200
Cl	1.48756800	-0.45105500	1.63687800
Cl	-1.32242900	-0.08063000	-3.05973200
Cl	-1.16974300	0.75184500	1.35389200
O	-0.25512800	-1.17168600	-0.20132700
H	-1.01209700	-0.67896600	-1.95584100
H	-0.64304300	-1.78460300	0.44731400

Zero-point correction= 0.043626 (Hartree/Particle)
Thermal correction to Energy= 0.051089
Thermal correction to Enthalpy= 0.052033
Thermal correction to Gibbs Free Energy= 0.007747
Sum of electronic and zero-point Energies= -1493.696919
Sum of electronic and thermal Energies= -1493.689456
Sum of electronic and thermal Enthalpies= -1493.688512
Sum of electronic and thermal Free Energies= -1493.732798

IRC

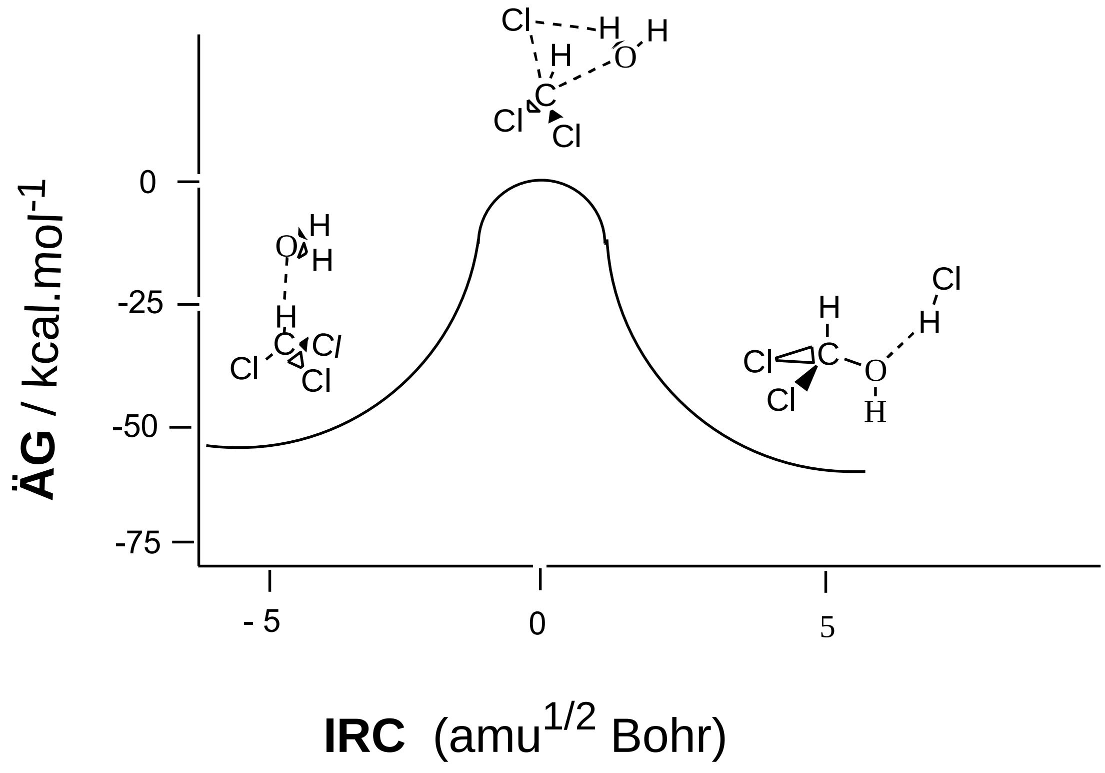

Figure-2

Intermediate (I-3)

C	0.47041800	0.29647300	0.22829400
H	0.36714900	-0.74800400	-0.12875400
Cl	1.78000500	0.50879800	1.37533900
Cl	0.70565400	1.33710500	-1.22239300
Cl	-1.11849000	0.72713300	0.97353300
O	-0.10505700	-2.60196700	1.24557500
H	-0.40361300	-2.48837200	0.30445200
H	-0.88957900	-2.31749800	1.73020200
O	-0.72406900	-1.91641300	-1.25887900
H	-0.23872800	-2.57691400	-1.76931300
Na	-1.86758600	-0.27724300	-1.73894600

Zero-point correction= 0.058121 (Hartree/Particle)

Thermal correction to Energy= 0.070039

Thermal correction to Enthalpy= 0.070983

Thermal correction to Gibbs Free Energy= 0.017883

Sum of electronic and zero-point Energies= -1731.223553

Sum of electronic and thermal Energies= -1731.211635

Sum of electronic and thermal Enthalpies= -1731.210691

Sum of electronic and thermal Free Energies= -1731.263791

Transition State

C	-1.01188600	0.15461600	-0.54836800
H	-1.22248400	-0.42891200	-1.45978400
Cl	0.56839400	0.37288300	0.00420500
Cl	-1.09297200	1.86903400	-2.41792900
Cl	-2.27425700	0.85434000	0.37007700
O	-1.32624200	-1.86534000	0.05471100
H	-1.74102900	-1.97808000	-0.90450900
H	-2.09469100	-1.88055200	0.64599300
O	-2.09179900	-1.71758100	-2.31901600
H	-1.56110200	-2.39964200	-2.75730200
Na	-2.57190600	0.04279400	-3.49388900

Zero-point correction= 0.057220 (Hartree/Particle)

Thermal correction to Energy= 0.067782

Thermal correction to Enthalpy= 0.068726

Thermal correction to Gibbs Free Energy= 0.019590

Sum of electronic and zero-point Energies= -1731.182855

Sum of electronic and thermal Energies= -1731.172292

Sum of electronic and thermal Enthalpies= -1731.171348

Sum of electronic and thermal Free Energies= -1731.220484

Product (1)

C	0.41873500	-0.36834600	0.96514500
H	0.71398400	-0.09609900	-0.06154300
Cl	1.56550700	0.30647500	2.14527500
Cl	0.45162300	0.25958700	-2.40541600
Cl	-1.22019600	0.43694300	1.23443800
O	0.30444500	-1.73011100	1.06507600
H	-0.81167600	-2.45033500	-0.60214100
H	0.13803100	-1.94645800	1.99894800
O	-1.21289300	-2.23841700	-1.46022600
H	-0.44493000	-1.85681400	-1.93938800
Na	-1.92164200	-0.00650300	-1.56433800

Zero-point correction= 0.062033 (Hartree/Particle)
Thermal correction to Energy= 0.073130
Thermal correction to Enthalpy= 0.074075
Thermal correction to Gibbs Free Energy= 0.023116
Sum of electronic and zero-point Energies= -1731.299333
Sum of electronic and thermal Energies= -1731.288236
Sum of electronic and thermal Enthalpies= -1731.287292
Sum of electronic and thermal Free Energies= -1731.338250

IRC

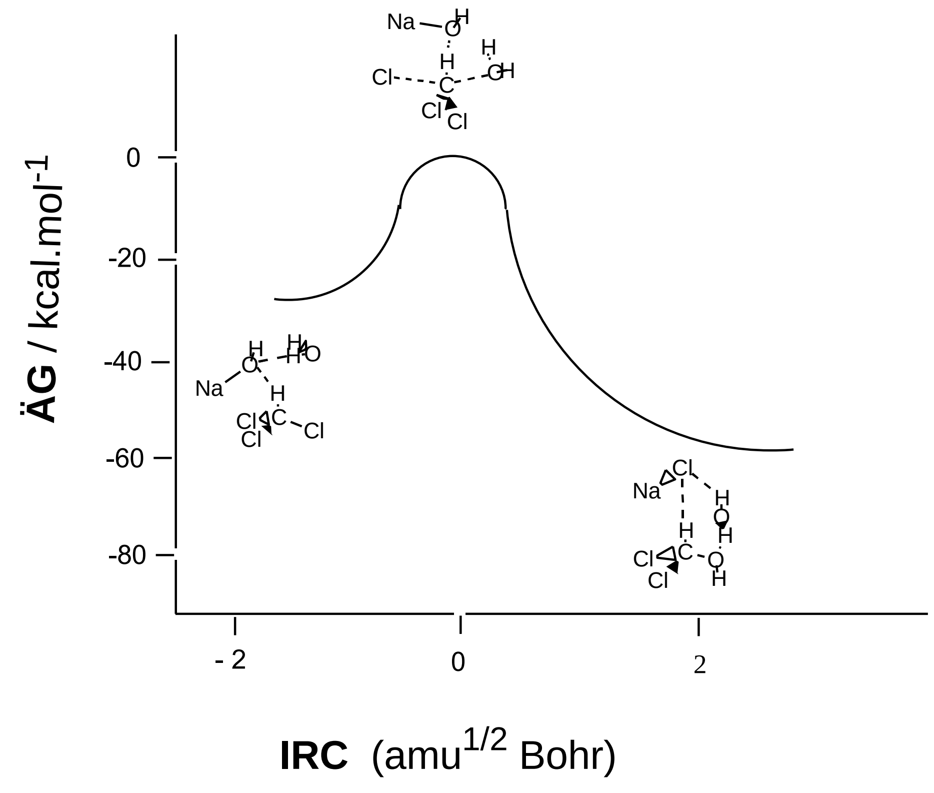

Figure-3

Intermediate (I-5)

C	-0.38766400	0.71629200	-0.18404800
H	0.30683300	0.74771700	-1.10549500
Cl	-0.03310200	-0.88149900	0.62407000
Cl	-0.01637000	2.03355100	0.95130700
Cl	-2.09985000	0.75508300	-0.66249000
O	1.39431600	0.35118600	-2.19447700
H	1.51591200	1.01691700	-2.88053200
Na	1.90196300	-1.45020700	-1.42225100
O	-2.45238500	0.25402200	2.84879700
H	-2.79175200	0.24159600	1.94510200
H	-1.73264700	0.89200100	2.76550100

Zero-point correction= 0.055594 (Hartree/Particle)
Thermal correction to Energy= 0.068629
Thermal correction to Enthalpy= 0.069573
Thermal correction to Gibbs Free Energy= 0.013645
Sum of electronic and zero-point Energies= -1731.211777
Sum of electronic and thermal Energies= -1731.198742
Sum of electronic and thermal Enthalpies= -1731.197798
Sum of electronic and thermal Free Energies= -1731.253726

Transition State

C	-0.66978500	-0.11527400	1.76232600
H	-0.08480300	0.79062700	1.83729900
Cl	0.11007400	-1.67996600	2.08504000
Cl	-1.02138200	0.32814400	3.95667400
Cl	-2.34127000	-0.13155700	1.29704000
O	-0.00442500	-0.10238000	-0.07488600
H	-0.46744600	0.66853500	-0.43523000
Na	0.80817500	-1.93774300	-0.59436600
O	-4.31665600	0.79843700	3.83746300
H	-4.36503500	-0.08714500	4.21811400
H	-3.35629700	0.93546000	3.85759200

Zero-point correction= 0.056728 (Hartree/Particle)
Thermal correction to Energy= 0.068734
Thermal correction to Enthalpy= 0.069678
Thermal correction to Gibbs Free Energy= 0.015314
Sum of electronic and zero-point Energies= -1731.154997
Sum of electronic and thermal Energies= -1731.142991
Sum of electronic and thermal Enthalpies= -1731.142046
Sum of electronic and thermal Free Energies= -1731.196411

Product (2)

C	-0.75719600	-1.40364900	-2.23565000
H	-1.11833400	-1.65855800	-1.22226900
Cl	-0.42697200	-0.59678400	1.06593400
Cl	0.55239100	3.33132300	0.57954700
Cl	-1.34200300	-2.49830400	-3.46872800
O	-0.02773100	-0.46672700	-2.49259000
H	-0.31860600	2.48906100	1.75744300
Na	0.71231300	1.04205700	-0.66390900
O	-0.81230400	1.83051200	2.48759000
H	-0.76456300	0.89697900	2.00571300
H	-0.16303700	1.75012900	3.20707900

Zero-point correction= 0.057063 (Hartree/Particle)
Thermal correction to Energy= 0.068666
Thermal correction to Enthalpy= 0.069611
Thermal correction to Gibbs Free Energy= 0.015217
Sum of electronic and zero-point Energies= -1731.333324
Sum of electronic and thermal Energies= -1731.321721
Sum of electronic and thermal Enthalpies= -1731.320777
Sum of electronic and thermal Free Energies= -1731.375171

IRC

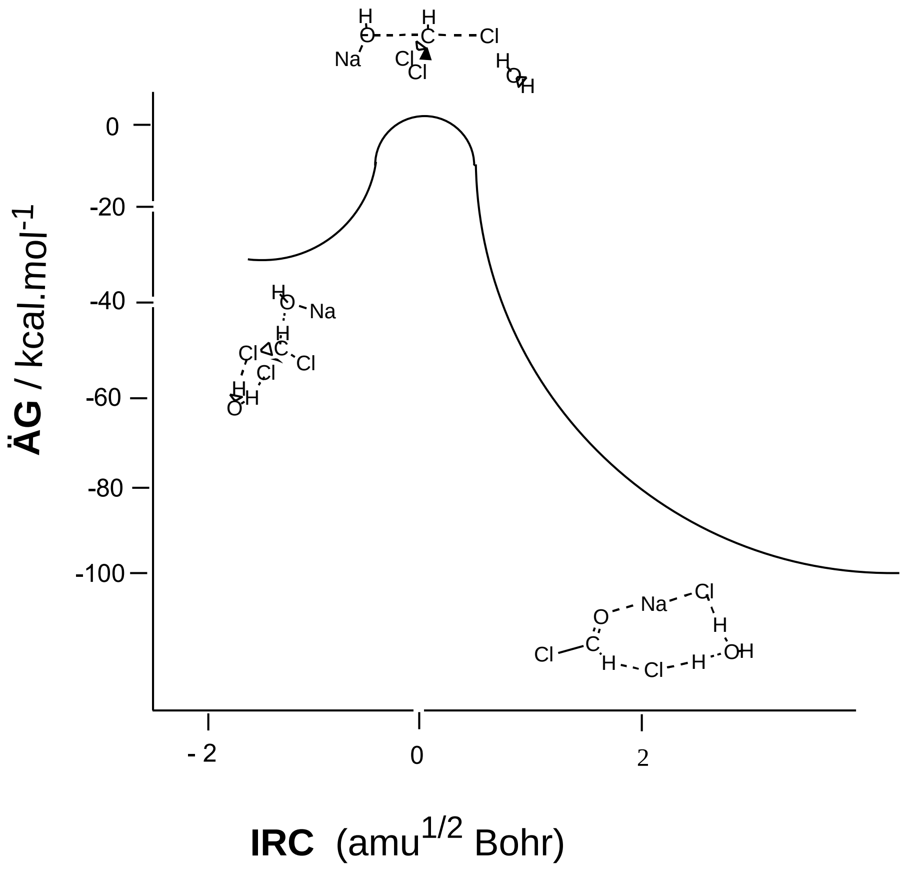

Figure-4

Intermediate I-7

C	-0.45034200	0.16220100	0.69298100
H	-0.84913300	-0.36928100	-0.22070900
Cl	1.23729500	-0.47239200	0.93292900
Cl	-0.37637200	1.91137200	0.37702600
Cl	-1.42525200	-0.19055400	2.12693400
O	-0.90909000	-1.17708600	-1.72140100
H	-1.78071000	-1.51072500	-1.96663100
Na	1.19570100	-1.50762600	-1.79338400
O	0.63157600	0.41370100	-2.93720400
H	0.59232200	1.28935100	-2.53151400
H	-0.20387300	-0.05304900	-2.57766600

Zero-point correction= 0.058723 (Hartree/Particle)
Thermal correction to Energy= 0.069815
Thermal correction to Enthalpy= 0.070759
Thermal correction to Gibbs Free Energy= 0.019866
Sum of electronic and zero-point Energies= -1731.246981
Sum of electronic and thermal Energies= -1731.235889
Sum of electronic and thermal Enthalpies= -1731.234945
Sum of electronic and thermal Free Energies= -1731.285838

Transition State 1

C	-0.21192400	0.09396600	0.58798800
H	-0.70129900	-0.68954700	-0.66619900
Cl	1.46612500	-0.59035100	1.12919900
Cl	0.07947200	1.86941500	0.26716000
Cl	-1.28501800	-0.03613600	2.02129600
O	-0.81849000	-1.24135500	-1.61095100
H	-1.61653500	-1.77334400	-1.49778400
Na	1.41918000	-1.27112000	-1.51869800
O	0.83784500	0.59112100	-2.80278300
H	0.79223800	1.37806900	-2.23638300
H	-0.06287100	0.22155600	-2.69954300

Zero-point correction= 0.055894 (Hartree/Particle)
Thermal correction to Energy= 0.066833
Thermal correction to Enthalpy= 0.067777
Thermal correction to Gibbs Free Energy= 0.017001
Sum of electronic and zero-point Energies= -1731.238875
Sum of electronic and thermal Energies= -1731.227936
Sum of electronic and thermal Enthalpies= -1731.226992
Sum of electronic and thermal Free Energies= -1731.277767

IRC

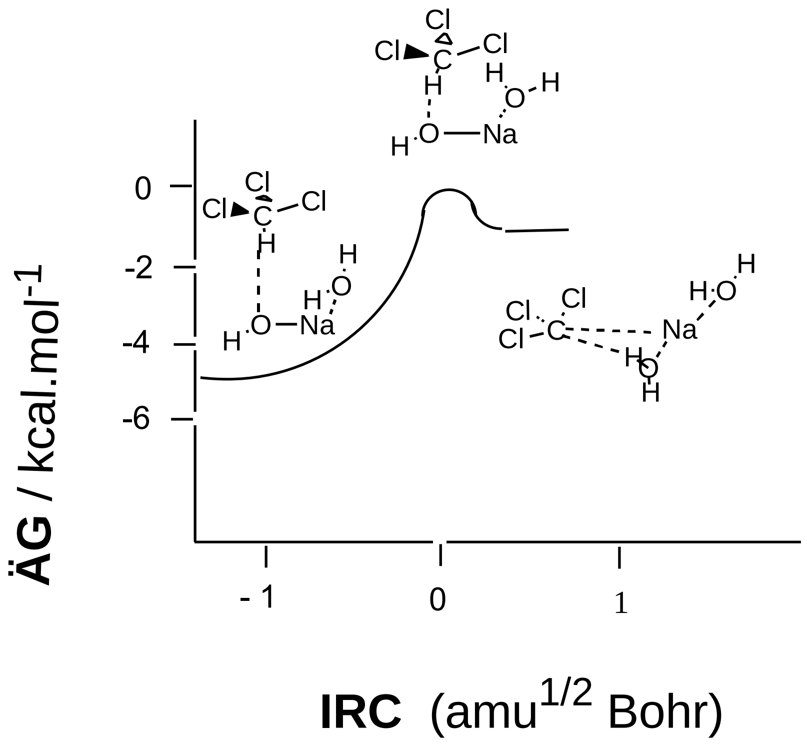

Intermediate I-8

C	0.33408700	0.06177700	0.46087700
H	-0.17424900	-1.49345000	-1.84914000
Cl	1.07110700	-0.66551600	1.95906200
Cl	1.54764200	-0.29591600	-0.91820800
Cl	0.44505700	1.91497300	0.69735600
O	-1.10361600	-1.76072600	-2.00340800
H	-1.17676800	-1.77415300	-2.96622000
Na	-1.95147900	-0.58138500	-0.21896900
O	-2.73024800	1.49768300	0.38488700
H	-1.84748600	1.85906200	0.60644500
H	-3.18616300	2.24832800	-0.01663300

Zero-point correction= 0.056782 (Hartree/Particle)
Thermal correction to Energy= 0.069693
Thermal correction to Enthalpy= 0.070637
Thermal correction to Gibbs Free Energy= 0.014628
Sum of electronic and zero-point Energies= -1731.235854
Sum of electronic and thermal Energies= -1731.222942
Sum of electronic and thermal Enthalpies= -1731.221998
Sum of electronic and thermal Free Energies= -1731.278007

Transition State 2

C	0.78350500	-0.35328200	1.09288900
H	-0.24502600	-0.93621500	-1.34242400
Cl	2.27843600	-1.12205700	0.69335700
Cl	-0.35250100	0.73675100	-2.77662400
Cl	0.02968600	-1.15732200	2.43692300
O	-0.42070300	-1.35838600	-0.46446400
H	-1.34408700	-1.08585700	-0.31203000
Na	-0.19454400	1.49828700	-0.30350500
O	-2.43792600	0.77498200	-0.61798400
H	-3.33520300	1.12538900	-0.69017100
H	-2.10500600	0.74910000	-1.55103800

Zero-point correction= 0.057487 (Hartree/Particle)
Thermal correction to Energy= 0.068985
Thermal correction to Enthalpy= 0.069929
Thermal correction to Gibbs Free Energy= 0.018041
Sum of electronic and zero-point Energies= -1731.213467
Sum of electronic and thermal Energies= -1731.201968
Sum of electronic and thermal Enthalpies= -1731.201024
Sum of electronic and thermal Free Energies= -1731.252913

IRC

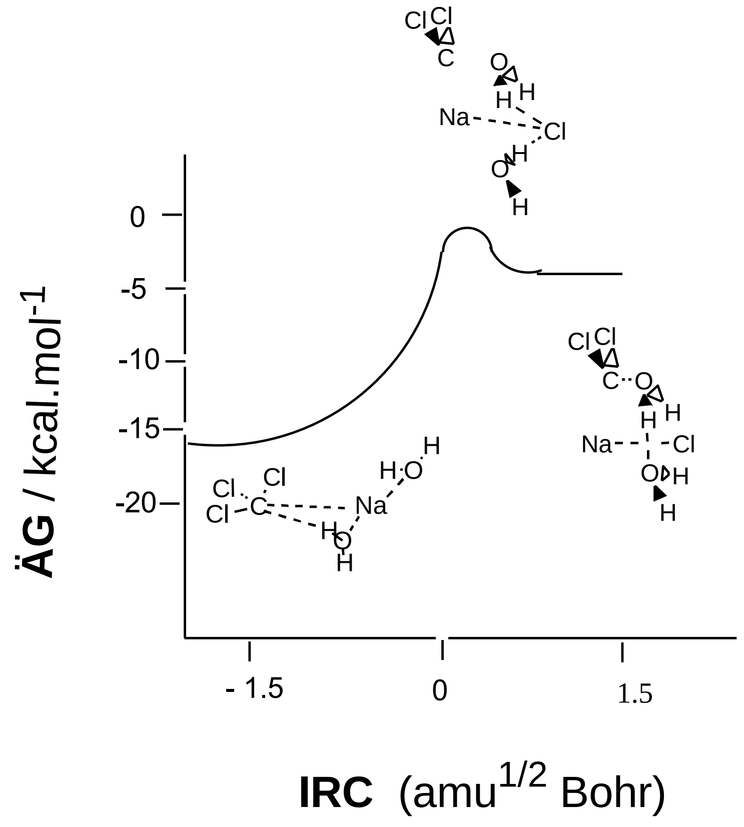

Intermediate I-9

C	0.39978600	-0.22505100	0.74845300
H	-0.24930300	-0.58789600	-1.23386600
Cl	2.01779900	-0.97196400	0.54856400
Cl	-0.32805700	0.72809100	-2.60925500
Cl	-0.33643000	-0.85757800	2.27793200
O	-0.45686000	-1.07835000	-0.34347400
H	-1.36696700	-0.72619200	-0.17790100
Na	-0.19668900	1.96267800	-0.25258900
O	-2.41840400	0.85627200	-0.49379400
H	-3.37637800	0.98678600	-0.48262200
H	-2.16377300	0.84600100	-1.44678600

Zero-point correction= 0.059165 (Hartree/Particle)
Thermal correction to Energy= 0.070202
Thermal correction to Enthalpy= 0.071146
Thermal correction to Gibbs Free Energy= 0.021013
Sum of electronic and zero-point Energies= -1731.219917
Sum of electronic and thermal Energies= -1731.208880
Sum of electronic and thermal Enthalpies= -1731.207936
Sum of electronic and thermal Free Energies= -1731.258070

Transition State 3

C	-0.24276200	-0.56727000	0.34869600
H	-0.70542600	-0.49696400	-0.87985000
Cl	0.87822700	-1.84219900	0.82100700
Cl	-1.63733900	2.01987700	-2.00494700
Cl	-0.88950300	0.27586500	1.81223300
O	-1.42093500	-1.29217800	-0.37541700
H	-2.27438100	-0.67947300	-0.29260100
Na	0.21140300	1.91554400	-0.31288500
O	-3.42895500	0.23531400	-0.38004000
H	-4.08084300	-0.10429600	-1.01143200
H	-2.98915200	0.96640200	-0.89312100

Zero-point correction= 0.054296 (Hartree/Particle)
Thermal correction to Energy= 0.065111
Thermal correction to Enthalpy= 0.066055
Thermal correction to Gibbs Free Energy= 0.015513
Sum of electronic and zero-point Energies= -1731.180482
Sum of electronic and thermal Energies= -1731.169667
Sum of electronic and thermal Enthalpies= -1731.168723
Sum of electronic and thermal Free Energies= -1731.219265

IRC

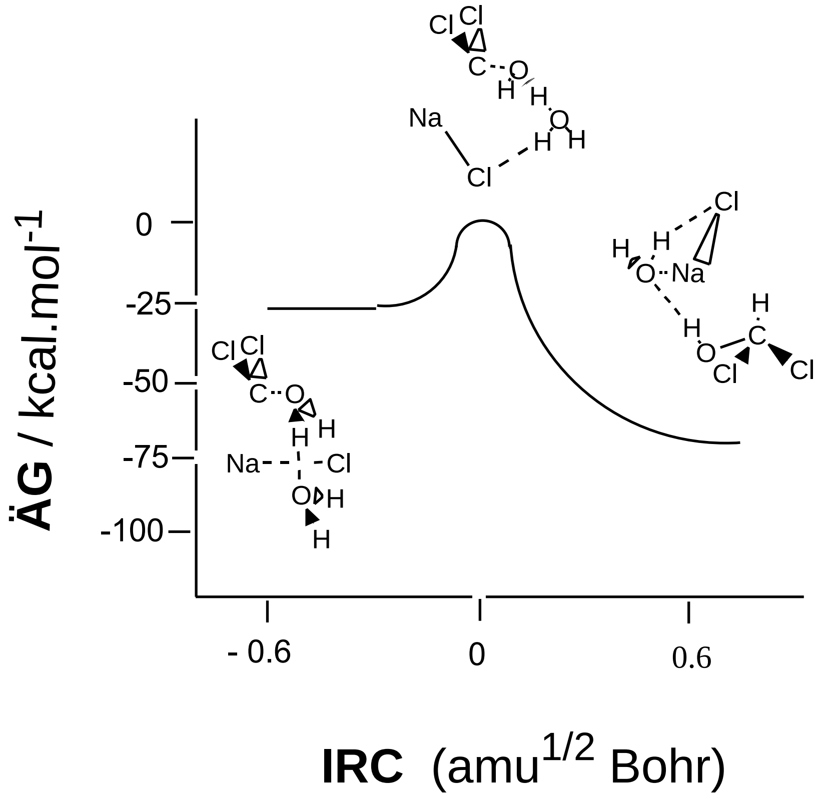

Product (1)

C	0.42360000	-1.10640500	0.16840800
H	0.53244900	-0.54067800	-0.77031000
Cl	1.71601700	-2.29410400	0.31801900
Cl	-0.37275600	1.47787500	-2.33971800
Cl	0.74869800	0.25002800	1.47642800
O	-0.76915200	-1.68465400	0.37765900
H	-1.44862400	-1.05285800	0.03317800
Na	-0.78914600	2.10629800	0.10526200
O	-2.34025300	0.33257500	-0.53104100
H	-3.26878100	0.25077700	-0.79144900
H	-1.85192800	0.57933000	-1.37788500

Zero-point correction= 0.061950 (Hartree/Particle)
Thermal correction to Energy= 0.072598
Thermal correction to Enthalpy= 0.073543
Thermal correction to Gibbs Free Energy= 0.023658
Sum of electronic and zero-point Energies= -1731.302985
Sum of electronic and thermal Energies= -1731.292337
Sum of electronic and thermal Enthalpies= -1731.291392
Sum of electronic and thermal Free Energies= -1731.341277

Figure-5

Intermediate (I-12)

C	-0.09564000	-0.94523400	-0.78686400
Cl	0.93542500	-2.26732100	-1.18068900
Cl	-1.09545200	1.33364400	1.63173100
Cl	-1.78552000	-1.31513800	-0.85410600
O	0.04876800	-1.88499600	2.05882500
H	-0.88406700	-2.13116800	2.01719600
H	-0.01679000	-0.92982200	2.19352000
C	0.37254900	0.30117700	-0.46035500
C	1.79875200	0.57333500	-0.34715300
C	2.25041900	1.86429000	-0.33327200
C	1.32085600	2.98113800	-0.44761100
C	-0.02386000	2.77811200	-0.39840800
C	-0.59069000	1.41600600	-0.16218300
H	2.49746200	-0.26549900	-0.29550000
H	3.32380300	2.06402700	-0.25480600
H	1.71458800	3.99472500	-0.57702700
H	-0.72769400	3.61499200	-0.45203100
H	-1.54399100	1.27507200	-0.68910000

Zero-point correction= 0.123471 (Hartree/Particle)
Thermal correction to Energy= 0.136929
Thermal correction to Enthalpy= 0.137873
Thermal correction to Gibbs Free Energy= 0.081365
Sum of electronic and zero-point Energies= -1723.899227
Sum of electronic and thermal Energies= -1723.885769
Sum of electronic and thermal Enthalpies= -1723.884825
Sum of electronic and thermal Free Energies= -1723.941333

Transition State

C	-1.49391700	-0.87648900	-0.73709800
Cl	-0.45502500	-2.18026700	-1.17567100
Cl	-2.57027100	1.34375000	1.83901500
Cl	-3.16623600	-1.19819400	-1.03556800
O	-1.46217400	-1.28297600	1.33725500
H	-2.23550300	-1.83575800	1.54410400
H	-1.78152800	-0.36854200	1.64461700
C	-1.01382300	0.45486700	-0.70731200
C	0.39430600	0.71135300	-0.61682400
C	0.84729300	2.01839200	-0.57338100
C	-0.07407500	3.10474100	-0.60318100
C	-1.44046900	2.86597300	-0.61918000
C	-1.94177700	1.53778000	-0.56582600
H	1.10059300	-0.12149600	-0.60349100
H	1.92221800	2.21687200	-0.52144200
H	0.30091100	4.13366900	-0.61471100
H	-2.15084400	3.69720600	-0.61021700
H	-3.00083200	1.36396200	-0.75015900

Zero-point correction= 0.124333 (Hartree/Particle)
Thermal correction to Energy= 0.135589
Thermal correction to Enthalpy= 0.136533
Thermal correction to Gibbs Free Energy= 0.086322
Sum of electronic and zero-point Energies= -1723.870185
Sum of electronic and thermal Energies= -1723.858928
Sum of electronic and thermal Enthalpies= -1723.857984
Sum of electronic and thermal Free Energies= -1723.908195

Product (3)

C	-0.36631600	-0.90741400	-0.21566500
Cl	-0.11109000	-1.85689700	-1.74327400
Cl	-0.00685100	-0.28006500	3.85908600
Cl	-2.15917400	-0.80241700	0.05645400
O	0.22303500	-1.57238600	0.84494800
H	-0.13991200	-2.47548200	0.85215100
H	-0.00510100	-0.71130800	2.63953200
C	0.23543000	0.46977000	-0.36053400
C	1.39304800	0.81101000	0.36260600
C	1.96112500	2.08617800	0.20165600
C	1.38829700	3.01328200	-0.68293800
C	0.23744200	2.66180300	-1.41155100
C	-0.33975400	1.39478700	-1.25318500
H	1.84769900	0.09126000	1.04638600
H	2.85641700	2.35149700	0.77303900
H	1.83447500	4.00551100	-0.80492400
H	-0.21448800	3.37868200	-2.10460800
H	-1.23598800	1.12288500	-1.81871100

Zero-point correction= 0.123464 (Hartree/Particle)
Thermal correction to Energy= 0.135607
Thermal correction to Enthalpy= 0.136551
Thermal correction to Gibbs Free Energy= 0.081696
Sum of electronic and zero-point Energies= -1723.959913
Sum of electronic and thermal Energies= -1723.947770
Sum of electronic and thermal Enthalpies= -1723.946826
Sum of electronic and thermal Free Energies= -1724.001681

IRC

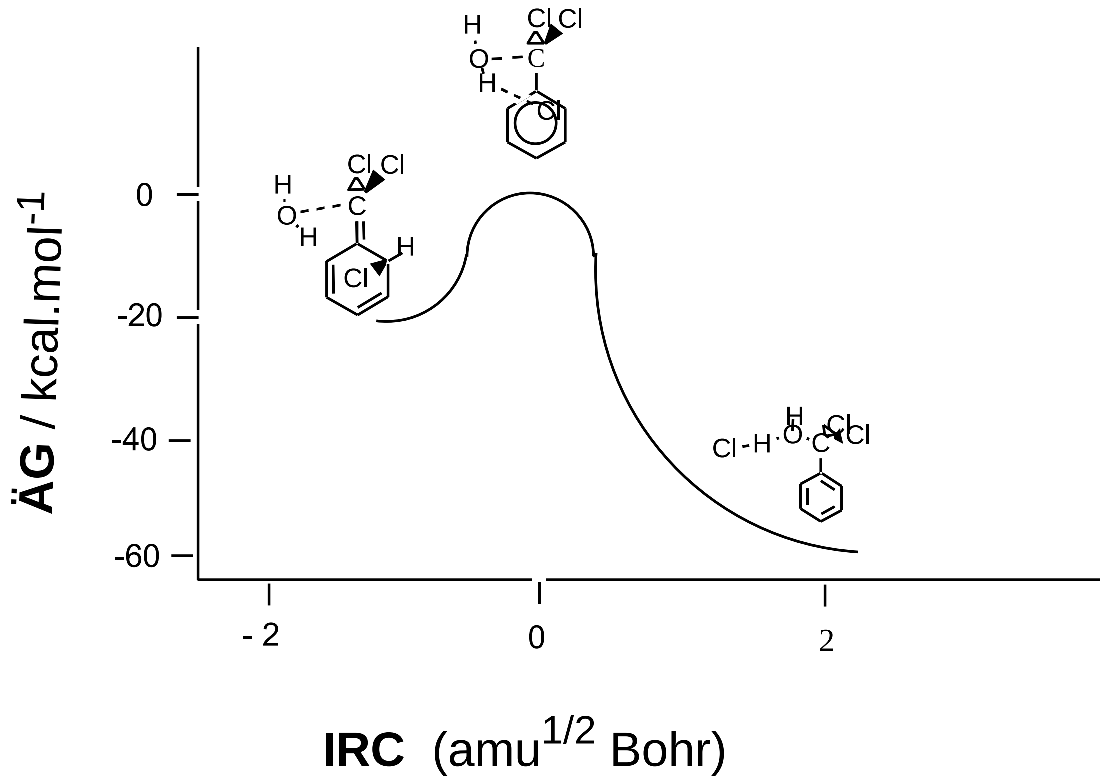

Figure-6

Intermediate (I-13)

C	0.51505700	-0.31429700	-0.79113200
Cl	0.72298700	-1.63931800	0.44849300
Cl	1.28577000	-0.89172700	-2.29956600
Cl	-1.24781500	-0.13775000	-1.12199200
O	-1.92573000	0.49314400	2.27163300
H	-2.09803800	1.09443000	3.00521400
C	1.09281700	0.97470800	-0.24896800
C	2.28074600	1.51937500	-0.77532900
C	2.80727800	2.69899900	-0.22409400
C	2.16088200	3.32737600	0.85127400
C	0.98216900	2.76455500	1.37213900
C	0.43045200	1.58835300	0.83995900
H	2.79519600	1.03994900	-1.61058200
H	3.72685100	3.12242800	-0.64147900
H	2.57291800	4.24759900	1.27936600
H	0.47304200	3.24402200	2.21552100
H	-0.50127800	1.17746400	1.30558000
Na	-2.12635500	-1.39890800	1.57741200
O	-2.24171900	-3.42614300	0.27592700
H	-2.92043600	-3.58914600	-0.39758400
H	-1.41587000	-3.42087700	-0.23747300

Zero-point correction= 0.136437 (Hartree/Particle)

Thermal correction to Energy= 0.154081

Thermal correction to Enthalpy= 0.155025

Thermal correction to Gibbs Free Energy= 0.087865

Sum of electronic and zero-point Energies= -1961.485850

Sum of electronic and thermal Energies= -1961.468207

Sum of electronic and thermal Enthalpies= -1961.467263

Sum of electronic and thermal Free Energies= -1961.534423

Transition State

C	-0.18848500	0.16544700	0.06797200
Cl	-1.12917800	0.72413700	-1.36253300
Cl	0.21537900	2.29799300	0.72261800
Cl	-1.13462000	-0.16127600	1.55473400
O	-0.48248600	-1.70260600	-0.43327500
H	0.16792600	-1.80455200	-1.14786900
C	1.28004900	-0.09017500	-0.01675400
C	2.02497700	0.41250300	-1.10214500
C	3.39339900	0.12009000	-1.20447200
C	4.03444700	-0.65836500	-0.22584900
C	3.28624100	-1.15128700	0.85740200
C	1.91150300	-0.89289900	0.95389400
H	1.54260600	1.05972500	-1.83896200
H	3.96385500	0.51993700	-2.04973900
H	5.10554600	-0.87164400	-0.30209000

H	3.77126200	-1.76122000	1.62726500
H	1.32923900	-1.29907200	1.78376200
Na	-2.62649700	-1.70658100	-0.34768400
O	-4.27471700	-0.05604800	-0.29241300
H	-4.83044200	0.19480800	0.45913300
H	-3.73054300	0.73724700	-0.43368500

Zero-point correction= 0.137010 (Hartree/Particle)
Thermal correction to Energy= 0.153363
Thermal correction to Enthalpy= 0.154307
Thermal correction to Gibbs Free Energy= 0.091943
Sum of electronic and zero-point Energies= -1961.438466
Sum of electronic and thermal Energies= -1961.422114
Sum of electronic and thermal Enthalpies= -1961.421170
Sum of electronic and thermal Free Energies= -1961.483533

Product (3)

C	-0.78151300	0.59803500	1.69617900
Cl	-0.07274300	-1.05007300	2.13149800
Cl	1.19892000	-2.15921100	-1.69318400
Cl	-2.12034900	0.30786100	0.49975400
O	-1.40843400	1.12118300	2.79944100
H	-0.69566800	1.36721400	3.41575400
C	0.28425300	1.47140100	1.07681000
C	1.22554800	0.95916900	0.16149800
C	2.15851900	1.82954900	-0.42144700
C	2.16357100	3.19830800	-0.09901500
C	1.21395900	3.70457100	0.80344700
C	0.26654100	2.84732000	1.38594800
H	1.24289600	-0.09819000	-0.12674400
H	2.88131900	1.42300500	-1.13578900
H	2.89900200	3.86879900	-0.55553400
H	1.20128200	4.77103700	1.05100300
H	-0.49400200	3.25158000	2.06014000
Na	-0.96323700	-2.23932700	-0.35499900
O	-1.06173100	-4.23196800	-1.52540800
H	-1.51086300	-4.47305200	-2.34563400
H	-0.19358200	-3.87811300	-1.83762200

Zero-point correction= 0.140882 (Hartree/Particle)
Thermal correction to Energy= 0.157215
Thermal correction to Enthalpy= 0.158160
Thermal correction to Gibbs Free Energy= 0.092494
Sum of electronic and zero-point Energies= -1961.559768
Sum of electronic and thermal Energies= -1961.543435
Sum of electronic and thermal Enthalpies= -1961.542491
Sum of electronic and thermal Free Energies= -1961.608157

IRC

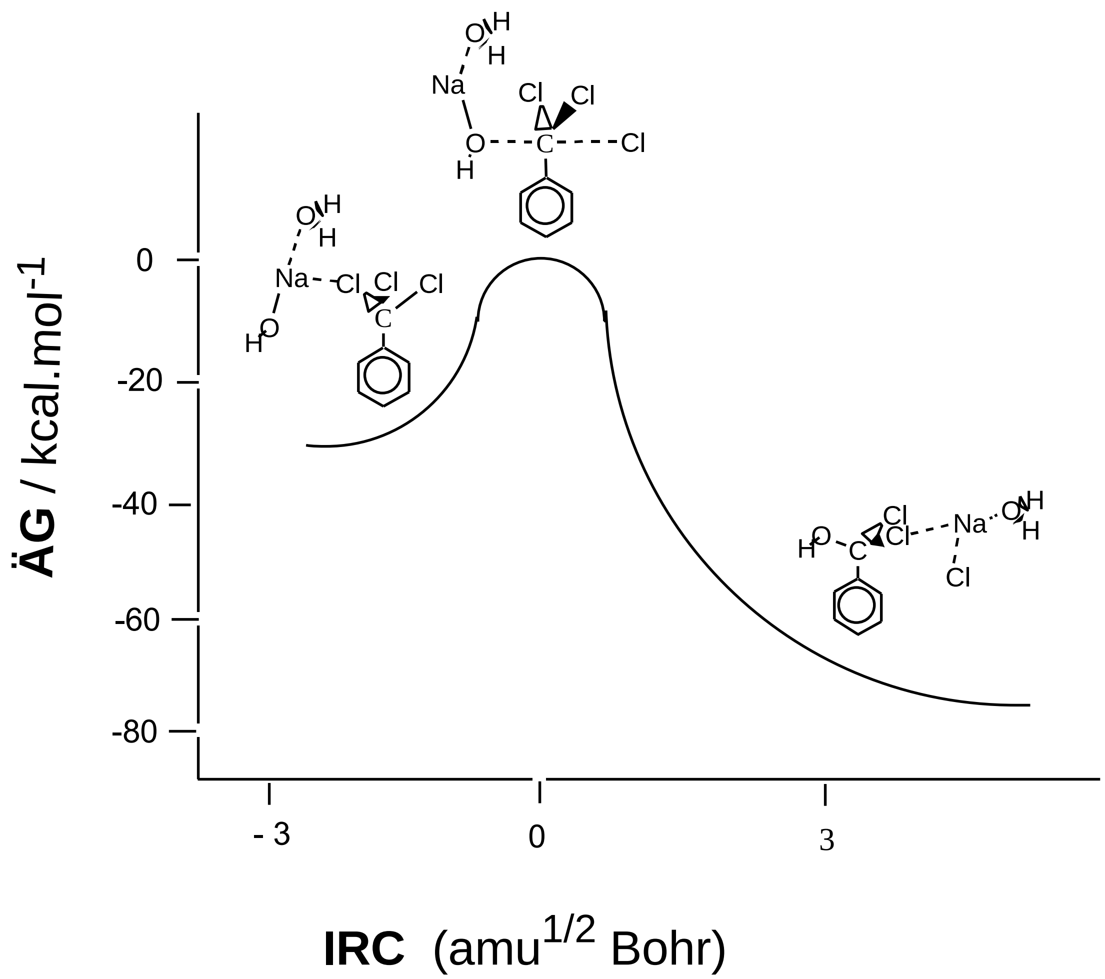

Figure-7

Intermediate (I-14)

C	-0.49254800	0.77962000	0.23226200
H	-0.74552600	1.84119600	0.13243700
Cl	0.78534400	0.68389400	1.53336700
Cl	0.29755900	0.29711600	-1.34170000
O	-0.40438400	-2.34699300	0.63968300
H	0.30786400	-2.13918500	1.26350700
H	0.05899600	-2.34049600	-0.21170800
O	-1.58529400	0.05670100	0.51494600
H	-1.29434900	-0.89039500	0.59316300

Zero-point correction= 0.059740 (Hartree/Particle)
Thermal correction to Energy= 0.067174
Thermal correction to Enthalpy= 0.068118
Thermal correction to Gibbs Free Energy= 0.027302
Sum of electronic and zero-point Energies= -1109.688374
Sum of electronic and thermal Energies= -1109.680940
Sum of electronic and thermal Enthalpies= -1109.679996
Sum of electronic and thermal Free Energies= -1109.720813

Transition State

C	0.70661900	-0.34512900	-0.66479900
H	0.72453600	0.68475600	-0.28519500
Cl	2.66785500	-0.88935200	0.38028300
Cl	1.23735100	-0.40302200	-2.36420200
O	1.16431900	-3.18906100	-0.20649500
H	0.95104200	-3.57091500	0.66196400
H	1.94694100	-2.53195100	0.00639400
O	-0.13169900	-1.17997600	-0.26413200
H	0.41358300	-2.36823800	-0.34580200

Zero-point correction= 0.055523 (Hartree/Particle)
Thermal correction to Energy= 0.061613
Thermal correction to Enthalpy= 0.062557
Thermal correction to Gibbs Free Energy= 0.024569
Sum of electronic and zero-point Energies= -1109.662590
Sum of electronic and thermal Energies= -1109.656500
Sum of electronic and thermal Enthalpies= -1109.655556
Sum of electronic and thermal Free Energies= -1109.693543

Product (2)

C	-1.05410600	1.05001200	-0.14778200
H	-0.87026300	2.08133500	0.20510200

Cl	1.81566400	-0.21892400	1.39088800
Cl	-0.35813000	0.80520200	-1.75022800
O	-0.24060400	-2.33361900	0.56426000
H	-0.61049300	-2.71760200	1.37216300
H	1.00909400	-1.19357300	1.02608700
O	-1.65181600	0.19875000	0.45274200
H	-0.94169900	-1.72312800	0.28230700

Zero-point correction= 0.053702 (Hartree/Particle)
Thermal correction to Energy= 0.062822
Thermal correction to Enthalpy= 0.063767
Thermal correction to Gibbs Free Energy= 0.016865
Sum of electronic and zero-point Energies= -1109.699083
Sum of electronic and thermal Energies= -1109.689963
Sum of electronic and thermal Enthalpies= -1109.689019
Sum of electronic and thermal Free Energies= -1109.735921

IRC

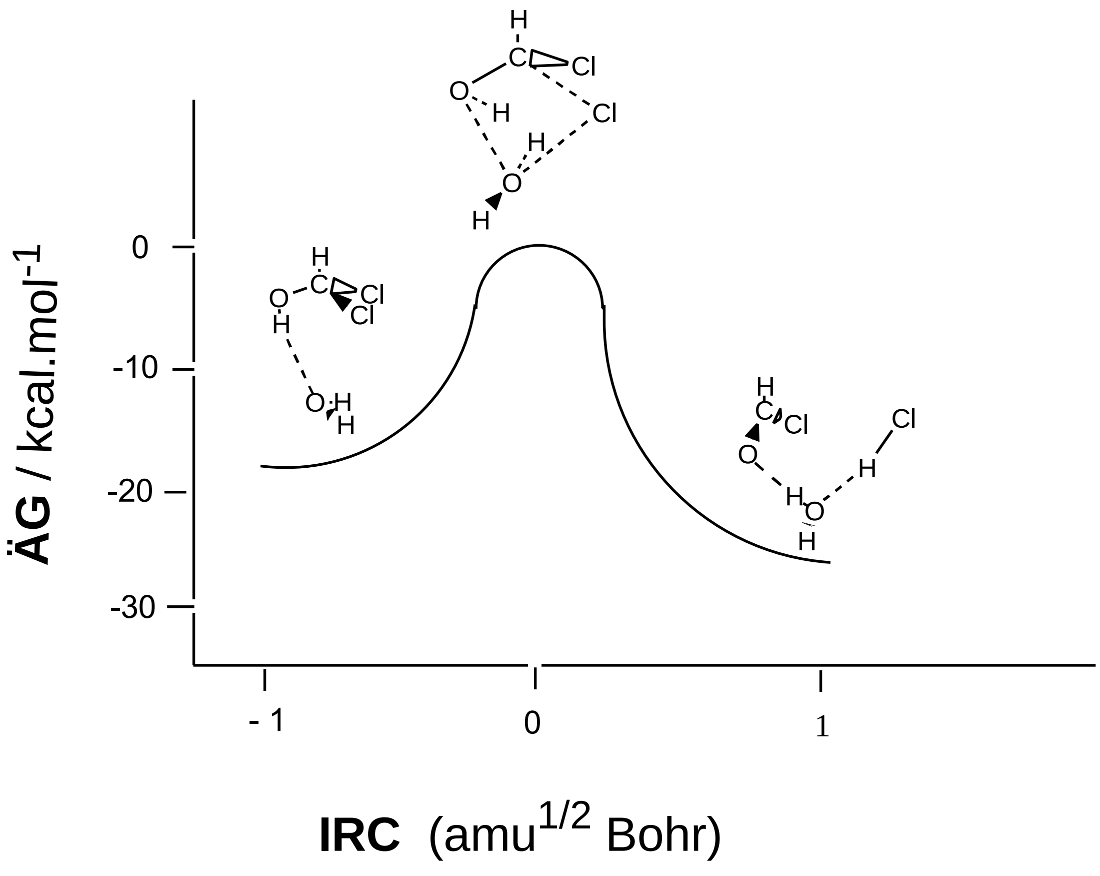

Figure-8

Intermediate (I-15)

C	-0.22158700	0.99164100	-0.86971100
H	-0.19391000	1.96990900	-1.36367100
Cl	-0.57566500	1.35795400	0.90126100
Cl	1.49201700	0.35016300	-0.94751800
O	0.80971300	-1.64647400	1.72422100
H	0.61465200	-0.70354100	1.84332400
H	1.62700000	-1.60925600	1.20620200
O	-1.11862900	0.18988300	-1.43175900
H	-1.13039900	-0.69533100	-0.94883300
O	-1.16516600	-2.07165400	-0.13028700
H	-1.97349000	-2.15659700	0.39370100
H	-0.45648500	-2.04992300	0.55364400

Zero-point correction= 0.085113 (Hartree/Particle)

Thermal correction to Energy= 0.095238

Thermal correction to Enthalpy= 0.096182

Thermal correction to Gibbs Free Energy= 0.048895

Sum of electronic and zero-point Energies= -1185.912346

Sum of electronic and thermal Energies= -1185.902221

Sum of electronic and thermal Enthalpies= -1185.901277

Sum of electronic and thermal Free Energies= -1185.948563

Transition State

C	-0.43359700	0.50148700	0.32589900
H	-0.58965800	1.56800400	0.11211100
Cl	-1.20197300	0.64050400	2.37707800
Cl	1.38695900	0.29379500	0.64160000
O	0.53964000	-1.99091000	2.76969800
H	0.29469900	-1.10013800	3.09346700
H	1.29165700	-1.77602800	2.19229300
O	-1.01763000	-0.40986000	-0.27338900
H	-1.26894100	-1.54255400	0.54644700
O	-1.54280100	-2.15580100	1.36524600
H	-2.01030200	-1.46251500	1.88925400
H	-0.65668900	-2.21586800	1.91438800

Zero-point correction= 0.083143 (Hartree/Particle)

Thermal correction to Energy= 0.091962

Thermal correction to Enthalpy= 0.092906

Thermal correction to Gibbs Free Energy= 0.048637

Sum of electronic and zero-point Energies= -1185.896673

Sum of electronic and thermal Energies= -1185.887854

Sum of electronic and thermal Enthalpies= -1185.886910

Sum of electronic and thermal Free Energies= -1185.931179

Product (2)

C	0.31839400	0.80940300	-1.23764400
H	-0.39266700	1.62951700	-1.43838800
Cl	-1.58530200	0.81417900	1.38161900
Cl	1.81026600	1.44492700	-0.51665500
O	0.97803200	-1.76020700	1.09339700
H	0.90268800	-0.97265100	1.65352800
H	1.41181200	-1.40567400	0.30346900
O	0.15710100	-0.35683600	-1.46764700
H	-1.56377100	-1.78890300	-0.64248500
O	-1.65560300	-1.89880000	0.31594000
H	-1.73766300	-0.43954800	0.89742600
H	-0.72462800	-2.06173900	0.59373900

Zero-point correction= 0.079316 (Hartree/Particle)
Thermal correction to Energy= 0.090895
Thermal correction to Enthalpy= 0.091839
Thermal correction to Gibbs Free Energy= 0.039389
Sum of electronic and zero-point Energies= -1185.923039
Sum of electronic and thermal Energies= -1185.911460
Sum of electronic and thermal Enthalpies= -1185.910516
Sum of electronic and thermal Free Energies= -1185.962967

IRC

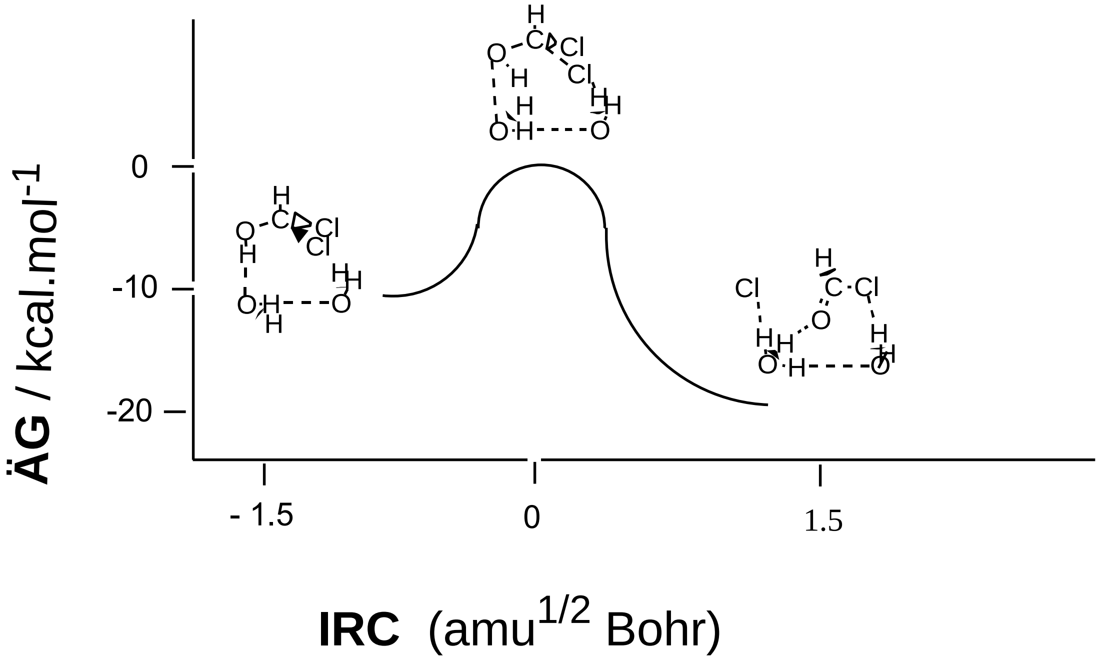

Figure-9

Intermediate (I-16)

C	0.70778700	-0.62372800	-0.51872500
H	0.16031000	0.08173400	-1.17174300
Cl	0.11213600	-0.36417900	1.17931500
O	1.54208800	-1.42813300	-0.79255700
O	-1.34300000	1.64230700	-1.22395000
H	-0.95294400	2.52293700	-1.14222800
H	-1.61859100	1.45708900	-0.31502400

Zero-point correction= 0.043189 (Hartree/Particle)

Thermal correction to Energy= 0.050183

Thermal correction to Enthalpy= 0.051128

Thermal correction to Gibbs Free Energy= 0.011529

Sum of electronic and zero-point Energies= -649.460252

Sum of electronic and thermal Energies= -649.453258

Sum of electronic and thermal Enthalpies= -649.452314

Sum of electronic and thermal Free Energies= -649.491912

Transition State

C	0.74866900	-0.29033300	1.25913300
H	-0.10278100	0.64944700	0.88777600
Cl	-0.22768600	0.20605800	3.36445800
O	1.56603600	-1.10225100	1.24038700
O	-1.03275600	1.49173100	1.00425100
H	-0.62226400	2.37150900	1.04433400
H	-1.08191300	1.21804000	1.99044900

Zero-point correction= 0.039687 (Hartree/Particle)

Thermal correction to Energy= 0.044818

Thermal correction to Enthalpy= 0.045762

Thermal correction to Gibbs Free Energy= 0.011031

Sum of electronic and zero-point Energies= -649.432245

Sum of electronic and thermal Energies= -649.427113

Sum of electronic and thermal Enthalpies= -649.426169

Sum of electronic and thermal Free Energies= -649.460900

Product (4)

C	1.10418400	-0.78544100	-1.30782000
H	-0.60922300	0.79991100	-1.57294400
Cl	-0.86982300	0.35937300	1.72606900
O	1.84877500	-1.58928500	-0.97560000
O	-1.17422100	1.45884100	-1.14018300
H	-0.64308800	2.26555300	-1.20353100

H -1.09625800 0.86942500 0.53923500

Zero-point correction= 0.038875 (Hartree/Particle)
Thermal correction to Energy= 0.046911
Thermal correction to Enthalpy= 0.047855
Thermal correction to Gibbs Free Energy= 0.004754
Sum of electronic and zero-point Energies= -649.482237
Sum of electronic and thermal Energies= -649.474201
Sum of electronic and thermal Enthalpies= -649.473256
Sum of electronic and thermal Free Energies= -649.516357

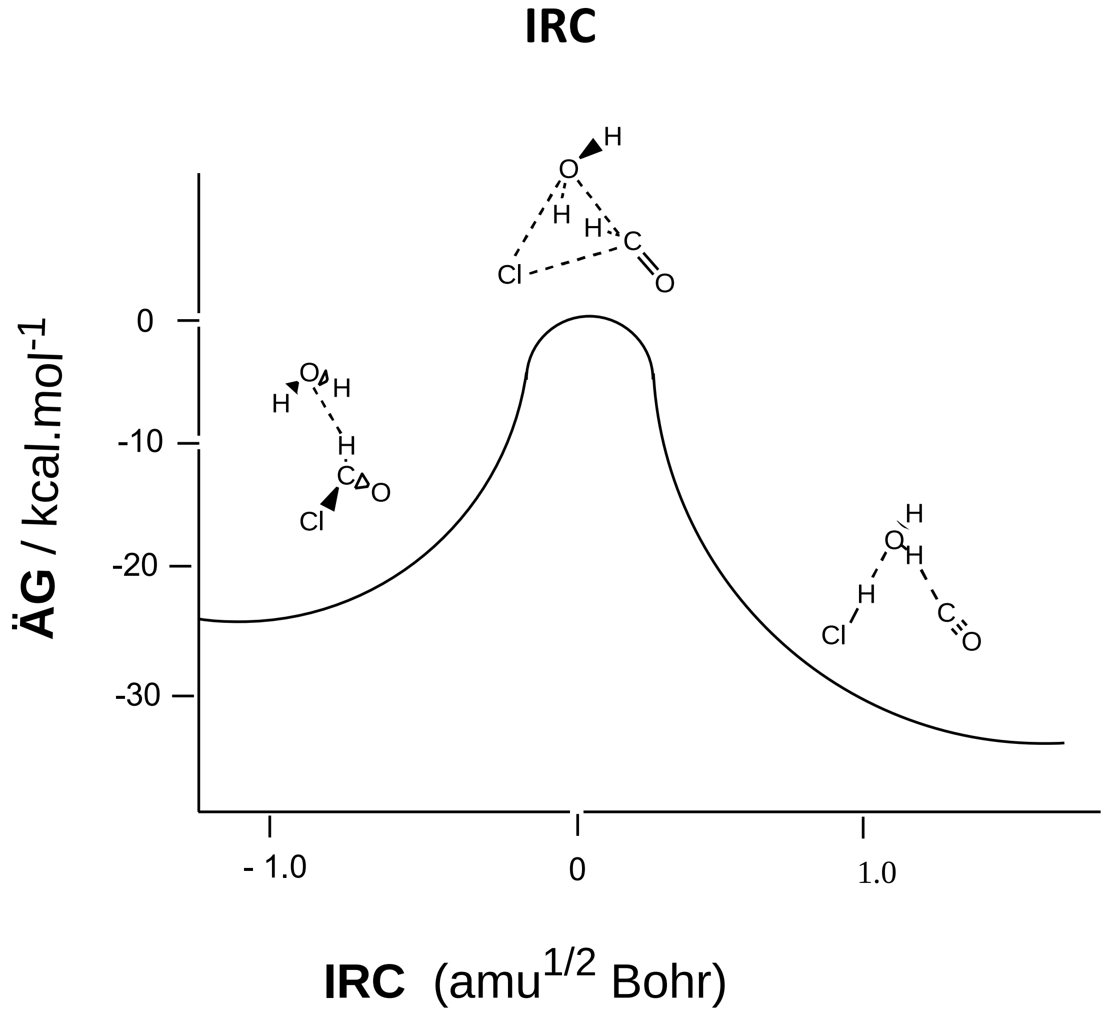

Figure-10

Intermediate (I-18)

C	-0.72151100	-0.91911400	-0.45179500
H	-1.65774200	-0.63194500	-0.95620100
Cl	-0.94155000	-0.86947100	1.31939800
O	0.30132500	-1.26789500	-0.96805200
O	-0.46023900	1.73163700	-0.70483700
H	0.50675400	1.60353900	-0.73839300
O	2.26093500	0.88322200	-0.71968600
H	2.56367200	0.83725700	-1.63702400
H	1.99084100	-0.03281300	-0.55290100
H	-0.57986800	2.11382000	0.17405800

Zero-point correction= 0.069200 (Hartree/Particle)

Thermal correction to Energy= 0.078735

Thermal correction to Enthalpy= 0.079679

Thermal correction to Gibbs Free Energy= 0.033887

Sum of electronic and zero-point Energies= -725.679489

Sum of electronic and thermal Energies= -725.669955

Sum of electronic and thermal Enthalpies= -725.669011

Sum of electronic and thermal Free Energies= -725.714803

Transition State

C	0.76714100	0.30329000	1.06760900
H	-0.25242600	0.13974300	0.68233300
Cl	0.47746100	0.22481000	3.00156400
O	1.74454600	-0.33807300	0.62480900
O	0.98810000	1.86861100	0.92325300
H	2.21928300	1.88952600	0.98603600
O	3.33199800	1.47012000	0.98524900
H	3.69768700	1.66279100	0.10758700
H	2.89436400	0.51168900	0.87312700
H	0.61899200	2.23717800	1.74732800

Zero-point correction= 0.069033 (Hartree/Particle)

Thermal correction to Energy= 0.074969

Thermal correction to Enthalpy= 0.075913

Thermal correction to Gibbs Free Energy= 0.038894

Sum of electronic and zero-point Energies= -725.644437

Sum of electronic and thermal Energies= -725.638501

Sum of electronic and thermal Enthalpies= -725.637557

Sum of electronic and thermal Free Energies= -725.674576

Product (5)

C	-0.67629200	-0.24233400	-0.47425100
H	-1.66582500	-0.62789800	-0.76044900
Cl	-0.63179500	-0.49076500	1.37549400
O	0.28266500	-0.93357200	-1.11333300
O	-0.53340400	1.13097400	-0.71427700
H	1.56327800	1.39087600	-0.45233000
O	2.31891600	0.80677000	-0.63016000
H	2.62491100	1.12261500	-1.49211800
H	1.13853200	-0.47906300	-0.91512800
H	-1.16959500	1.58172700	-0.13765400

Zero-point correction= 0.074106 (Hartree/Particle)
Thermal correction to Energy= 0.081471
Thermal correction to Enthalpy= 0.082415
Thermal correction to Gibbs Free Energy= 0.042351
Sum of electronic and zero-point Energies= -725.677691
Sum of electronic and thermal Energies= -725.670327
Sum of electronic and thermal Enthalpies= -725.669382
Sum of electronic and thermal Free Energies= -725.709447

IRC

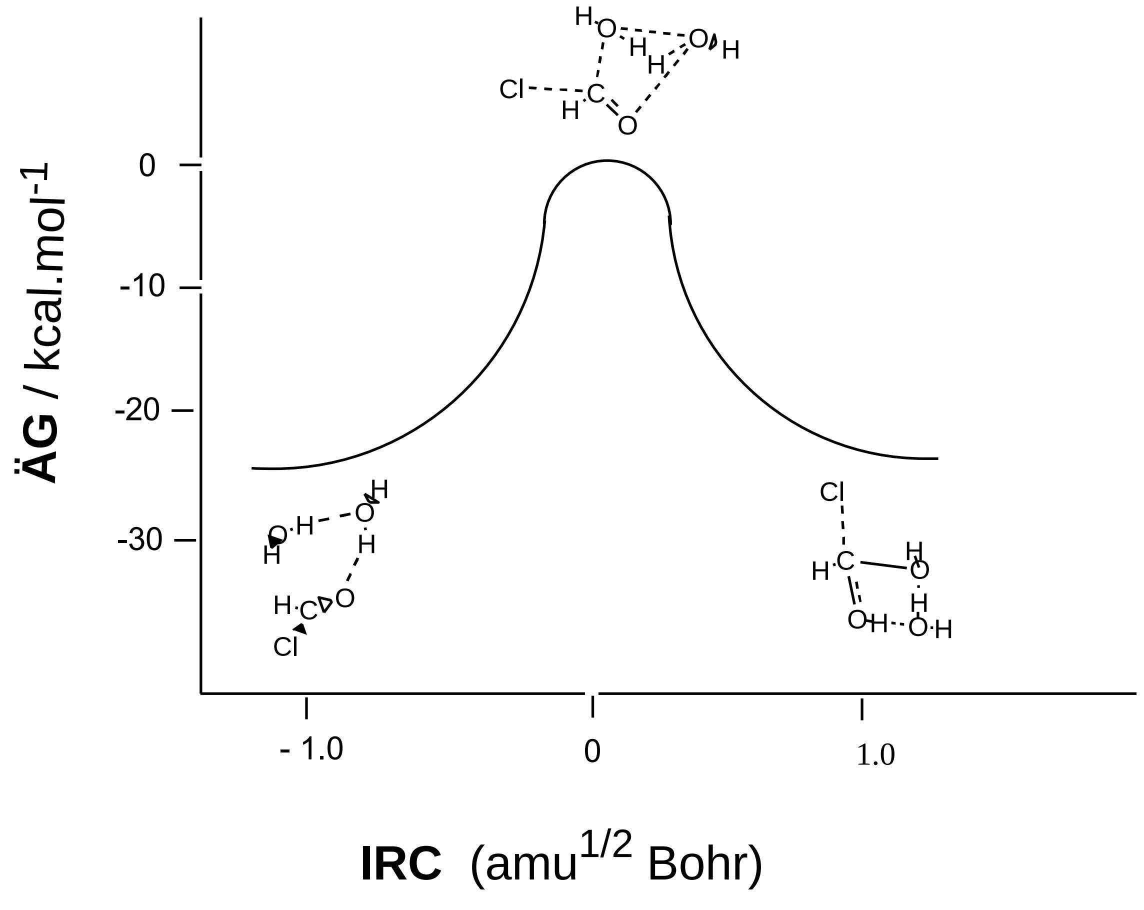

Figure-11

Intermediate (i-19)

C	-0.91760500	-0.82642200	0.29479500
H	-1.42301500	-1.72911300	0.65423800
Cl	0.84610000	-1.03702300	1.07595400
O	-0.77587000	-0.88992100	-1.06310000
O	-0.55514200	2.44769100	-0.51182700
H	-1.11739800	2.60898000	-1.28191500
H	0.28984300	2.17159200	-0.91012300
O	-1.50107800	0.26897800	0.81450800
H	-0.12442900	-0.21363500	-1.33441700
H	-1.13831700	1.07488900	0.36839200
O	1.47786800	0.78986500	-1.40528200
H	1.73525400	0.30092900	-0.59984300
H	2.15711800	0.55528200	-2.05190300

Zero-point correction= 0.100021 (Hartree/Particle)
Thermal correction to Energy= 0.109931
Thermal correction to Enthalpy= 0.110876
Thermal correction to Gibbs Free Energy= 0.065181
Sum of electronic and zero-point Energies= -801.904337
Sum of electronic and thermal Energies= -801.894427
Sum of electronic and thermal Enthalpies= -801.893482
Sum of electronic and thermal Free Energies= -801.939177

Transition State

C	-0.83827900	-0.69993100	0.06477900
H	-1.20928700	-1.60734200	0.54838600
Cl	1.58626900	-0.98401300	1.10535000
O	-0.41580000	-0.82268700	-1.14620900
O	-0.16151200	2.47244900	-0.46305100
H	-0.58587100	2.80417500	-1.26687400
H	0.70133000	2.12358200	-0.77672800
O	-1.13153400	0.39626500	0.65333100
H	0.33760700	-0.14356800	-1.34549100
H	-0.77180400	1.23020200	0.16940800
O	1.62158500	0.63869900	-1.20365800
H	1.80505800	0.10072200	-0.31776300
H	2.35098300	0.38723900	-1.79010400

Zero-point correction= 0.097399 (Hartree/Particle)
Thermal correction to Energy= 0.105763
Thermal correction to Enthalpy= 0.106707
Thermal correction to Gibbs Free Energy= 0.064317
Sum of electronic and zero-point Energies= -801.897601
Sum of electronic and thermal Energies= -801.889237

Sum of electronic and thermal Enthalpies= -801.888293

Sum of electronic and thermal Free Energies= -801.930683

Product (6)

C	-2.16903200	-0.67283200	-0.34739300
H	-3.00608200	-1.38993700	-0.24608000
Cl	3.66151500	-0.48485900	0.87798800
O	-1.06093200	-1.02508000	-0.73754900
O	-0.57650700	2.28453600	-0.28134900
H	-0.70114400	2.90736100	-1.00981000
H	0.23561900	1.79469100	-0.52960200
O	-2.54667700	0.54142900	-0.00128100
H	0.48217800	-0.22774600	-0.88560000
H	-1.77807900	1.18294600	-0.10482700
O	1.26967900	0.36298800	-0.96712800
H	2.63958100	-0.13132500	0.14783800
H	1.56213200	0.22357800	-1.87964300

Zero-point correction= 0.095857 (Hartree/Particle)

Thermal correction to Energy= 0.106585

Thermal correction to Enthalpy= 0.107529

Thermal correction to Gibbs Free Energy= 0.056994

Sum of electronic and zero-point Energies= -801.936887

Sum of electronic and thermal Energies= -801.926159

Sum of electronic and thermal Enthalpies= -801.925215

Sum of electronic and thermal Free Energies= -801.975750

IRC

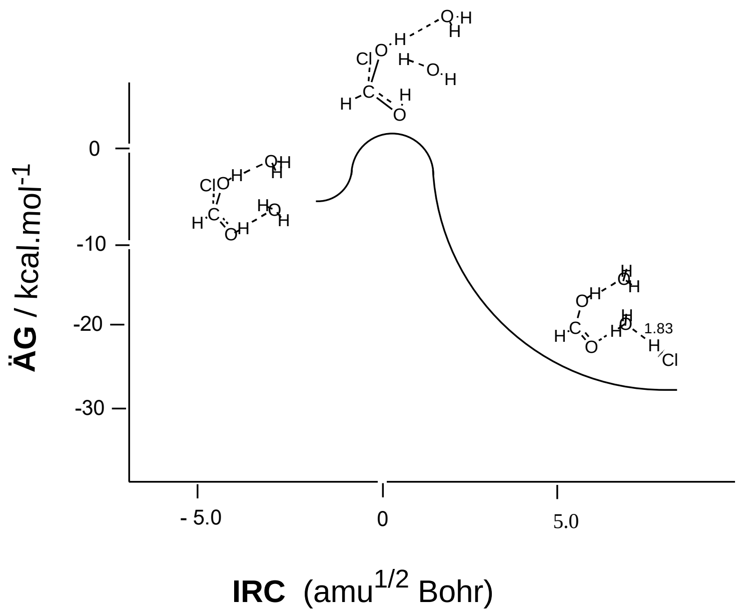

Figure-12

Intermediate (I-20)

C	-0.46314600	-0.17729200	0.18296700
O	0.15669900	0.10465400	1.19141600
Na	2.40892900	0.26734700	0.15509600
O	1.51001700	-0.19792200	-1.61356400
H	0.05017500	-0.28455100	-0.84040000
H	1.59568800	-0.35795900	-2.55990000
Cl	-2.23088700	-0.43913800	0.26531700

Zero-point correction= 0.031983 (Hartree/Particle)

Thermal correction to Energy= 0.039227

Thermal correction to Enthalpy= 0.040171

Thermal correction to Gibbs Free Energy= -0.000029

Sum of electronic and zero-point Energies= -810.777724

Sum of electronic and thermal Energies= -810.770480

Sum of electronic and thermal Enthalpies= -810.769536

Sum of electronic and thermal Free Energies= -810.809736

Transition State

C	-1.05971200	0.53528900	1.49799000
O	-0.40255300	1.01033800	2.39942400
Na	1.64595800	1.69633500	1.24924700
O	0.60858600	0.95771100	-0.32312100
H	-0.74332200	-0.24161400	0.78984900
H	0.54962700	0.92393400	-1.28484400
Cl	-2.72407100	1.07153300	1.19208600

Zero-point correction= 0.032538 (Hartree/Particle)

Thermal correction to Energy= 0.039144

Thermal correction to Enthalpy= 0.040088

Thermal correction to Gibbs Free Energy= 0.001234

Sum of electronic and zero-point Energies= -810.769298

Sum of electronic and thermal Energies= -810.762692

Sum of electronic and thermal Enthalpies= -810.761748

Sum of electronic and thermal Free Energies= -810.800602

Product (6)

C	0.44891100	-1.40000800	-0.07454300
O	0.93524300	-0.64822200	0.77977300
Na	0.81005600	1.62990300	0.84244200
O	-0.24421400	-1.06327000	-1.12988000
H	0.57343500	-2.49747500	-0.00452700
H	-0.35299300	-0.04626200	-1.18273500

```
Cl                 -0.54054000    1.87849300   -1.25479100
```

Zero-point correction= 0.036620 (Hartree/Particle)
Thermal correction to Energy= 0.042942
Thermal correction to Enthalpy= 0.043886
Thermal correction to Gibbs Free Energy= 0.005405
Sum of electronic and zero-point Energies= -810.868531
Sum of electronic and thermal Energies= -810.862209
Sum of electronic and thermal Enthalpies= -810.861265
Sum of electronic and thermal Free Energies= -810.899746

IRC

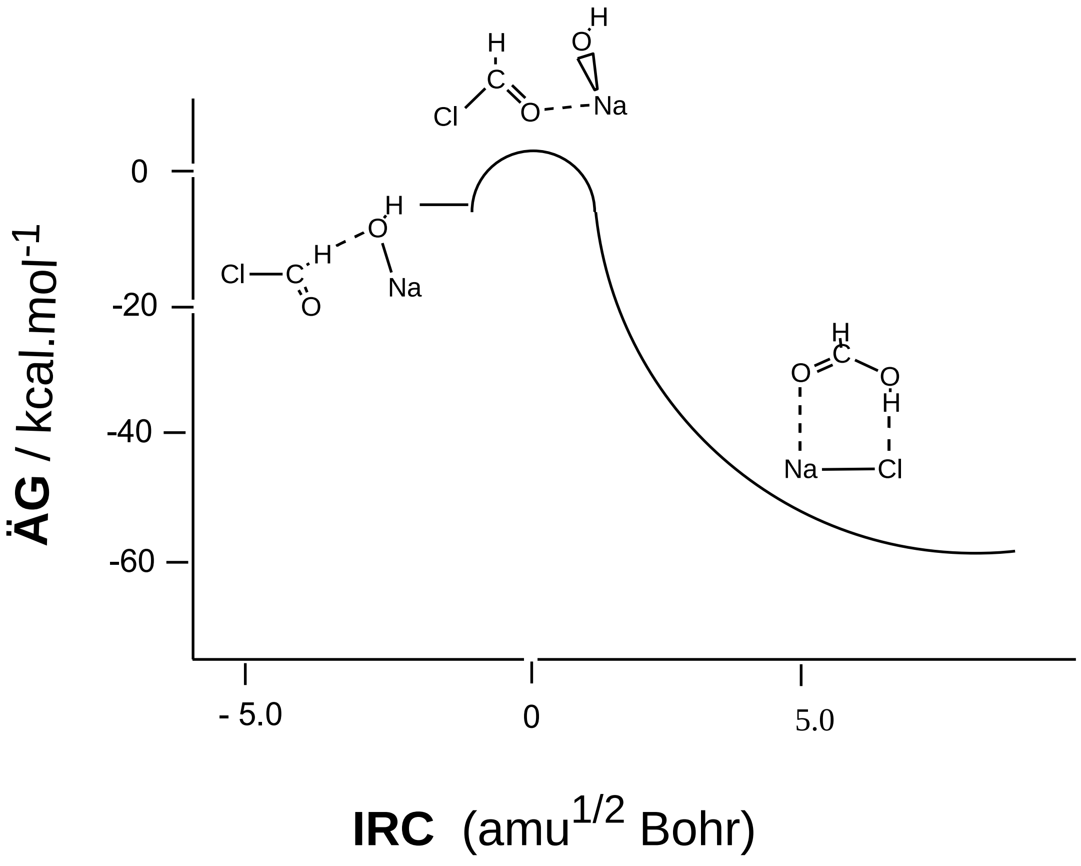

Figure-13

Intermediate (tt-7)

C	0.65174300	-1.03962900	0.43050000
O	0.31125400	0.13811100	-0.10084700
O	1.85952500	-0.88161600	0.93334000
H	-0.61547600	0.09535500	-0.48478000
Na	-0.01693400	2.42291600	-0.44168200
O	1.70667700	-3.42894400	1.69783000
H	2.01808600	-4.01974000	0.99731300
H	0.80167400	-3.22152100	1.40282900
H	2.06057400	-1.77559800	1.31223500
Cl	-2.11210300	1.31222700	-1.26401100

Zero-point correction= 0.061105 (Hartree/Particle)
Thermal correction to Energy= 0.070936
Thermal correction to Enthalpy= 0.071880
Thermal correction to Gibbs Free Energy= 0.023487
Sum of electronic and zero-point Energies= -887.005520
Sum of electronic and thermal Energies= -886.995689
Sum of electronic and thermal Enthalpies= -886.994745
Sum of electronic and thermal Free Energies= -887.043138

Transition State

C	0.33452100	0.25256300	0.35628500
O	-0.10794200	1.36682500	-0.23652700
O	1.50125700	0.36161200	0.85588600
H	-1.04558800	1.24625900	-0.58580600
Na	-0.68009900	3.60474700	-0.56255900
O	0.88290100	-1.84552000	1.38649200
H	1.23768000	-2.44720900	0.71363100
H	0.16024200	-1.15876100	0.79583200
H	1.49647900	-0.79484400	1.26682500
Cl	-2.67028700	2.25586200	-1.30570700

Zero-point correction= 0.056078 (Hartree/Particle)
Thermal correction to Energy= 0.064302
Thermal correction to Enthalpy= 0.065246
Thermal correction to Gibbs Free Energy= 0.020381
Sum of electronic and zero-point Energies= -886.989738
Sum of electronic and thermal Energies= -886.981514
Sum of electronic and thermal Enthalpies= -886.980570
Sum of electronic and thermal Free Energies= -887.025434

Product (6)

C	0.94709800	-0.82939700	0.53343800
O	0.40874800	0.25983300	-0.05146500
O	2.06850900	-0.82626900	0.99526100
H	-0.54326700	0.10308900	-0.37337700
Na	-0.24027600	2.41953400	-0.66247600
O	1.53280800	-3.60686400	1.66492100
H	1.95570700	-4.03660000	0.91083900
H	0.28104500	-1.71446200	0.55199800
H	2.01779000	-2.76783300	1.70737300
Cl	-2.18535100	0.87062400	-1.13122800

Zero-point correction= 0.060225 (Hartree/Particle)
Thermal correction to Energy= 0.070422
Thermal correction to Enthalpy= 0.071366
Thermal correction to Gibbs Free Energy= 0.021798
Sum of electronic and zero-point Energies= -887.064234
Sum of electronic and thermal Energies= -887.054038
Sum of electronic and thermal Enthalpies= -887.053093
Sum of electronic and thermal Free Energies= -887.102662

IRC

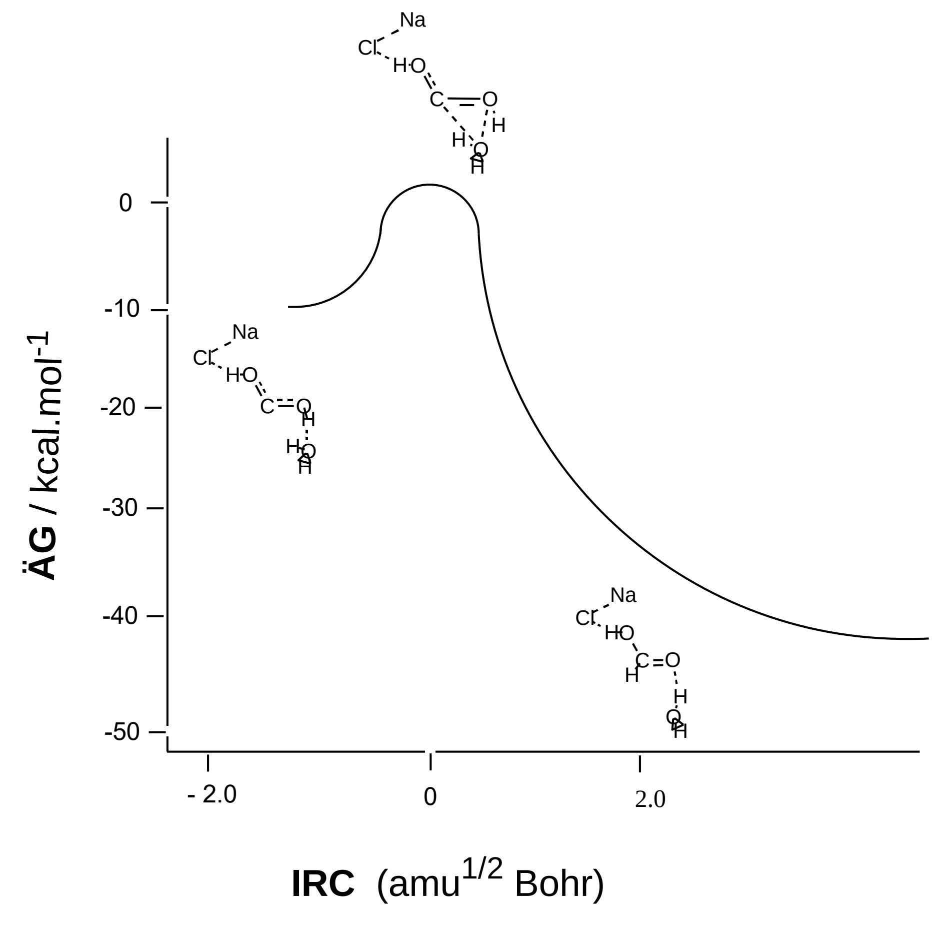

Figure-14

Intermediate (ct-7)

C	1.27133000	0.30305300	0.93862200
O	2.29727500	0.63915300	1.70996700
Na	-0.64222000	-0.25150300	-2.08873000
O	1.40888700	0.53319100	-1.87650300
H	1.45454600	0.47907400	-0.86379200
H	1.95486600	1.29703300	-2.09843500
Cl	-1.97502000	-0.84258000	-0.04753900
O	0.28119700	-0.11782300	1.66813900
H	-0.50516200	-0.37195600	1.06750500
H	2.03090000	0.47622200	2.64247000

Zero-point correction= 0.060224 (Hartree/Particle)

Thermal correction to Energy= 0.069605

Thermal correction to Enthalpy= 0.070550

Thermal correction to Gibbs Free Energy= 0.024106

Sum of electronic and zero-point Energies= -887.034703

Sum of electronic and thermal Energies= -887.025322

Sum of electronic and thermal Enthalpies= -887.024377

Sum of electronic and thermal Free Energies= -887.070821

Transition State

C	0.62416300	-0.60913200	0.38066800
O	1.12966600	-0.06738800	1.39888300
Na	-1.59173500	-1.02694900	-2.35525600
O	0.55564800	-0.47456800	-2.46293600
H	0.75057100	-0.49130800	-1.47737000
H	1.14359700	0.20594400	-2.81264600
Cl	-2.87991500	-1.48796100	-0.23216200
O	-0.29881600	-1.51367000	1.04388300
H	-1.26771400	-1.52193600	0.64959000
H	0.22246600	-0.83240200	1.93126600

Zero-point correction= 0.053402 (Hartree/Particle)

Thermal correction to Energy= 0.062851

Thermal correction to Enthalpy= 0.063795

Thermal correction to Gibbs Free Energy= 0.016739

Sum of electronic and zero-point Energies= -886.985524

Sum of electronic and thermal Energies= -886.976074

Sum of electronic and thermal Enthalpies= -886.975130

Sum of electronic and thermal Free Energies= -887.022186

Product (4)

C	2.31036300	1.43070100	0.96750700
O	2.07387500	1.74742600	2.03829500
Na	0.06722400	-1.24215300	-1.00631700
O	1.41439800	0.39550900	-1.95742400
H	2.13168400	0.71398100	-1.38894200
H	0.67069100	0.96965800	-1.66744000
Cl	-1.27807100	0.89452200	-0.39991300
O	0.10157600	-1.22510400	1.29751900
H	-0.43804500	-0.41286700	1.12972000
H	-0.35708900	-1.66030600	2.02728800

Zero-point correction= 0.062991 (Hartree/Particle)
Thermal correction to Energy= 0.074757
Thermal correction to Enthalpy= 0.075701
Thermal correction to Gibbs Free Energy= 0.022346
Sum of electronic and zero-point Energies= -887.086778
Sum of electronic and thermal Energies= -887.075012
Sum of electronic and thermal Enthalpies= -887.074068
Sum of electronic and thermal Free Energies= -887.127423

IRC

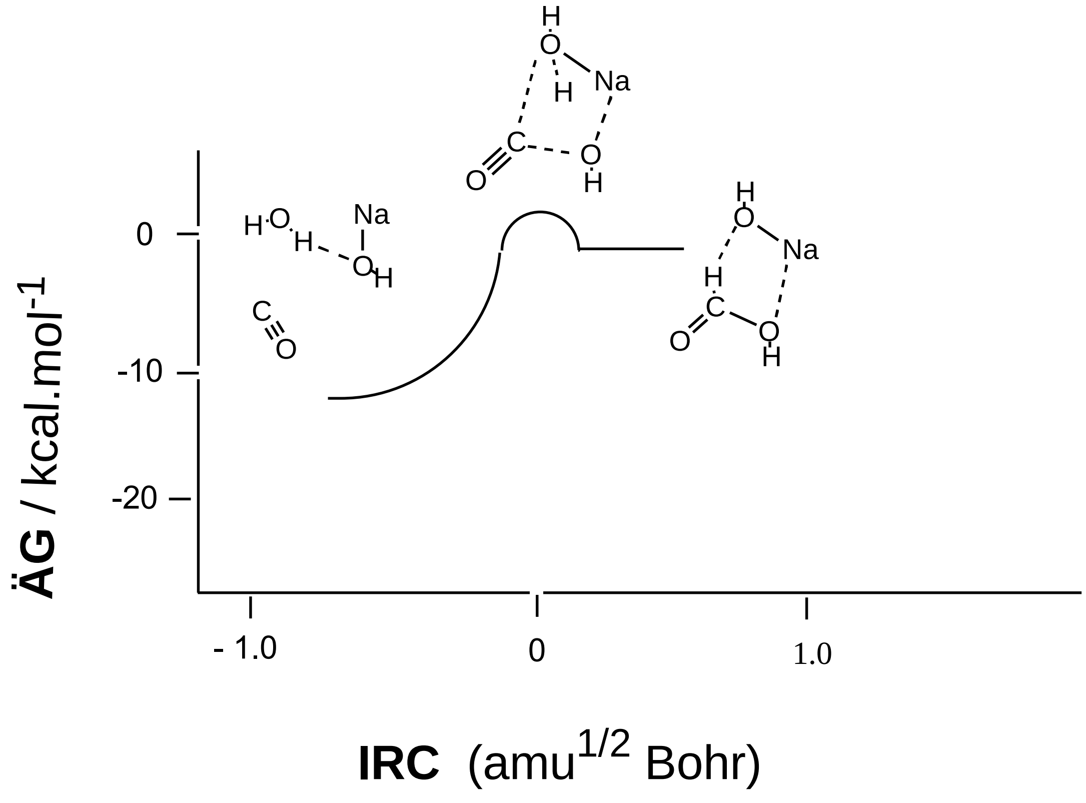

Figure-15

Intermediate (I-21)

C	-1.40206000	-1.10479400	1.35907300
O	0.54849200	-0.42584500	-0.64805000
O	-1.25649700	-2.24262300	1.32244600
H	1.11700600	-1.18607800	-0.48604200
Na	-0.16086200	1.22429900	-1.66153800
O	0.18608100	1.71817800	0.55122000
H	-0.49059000	1.57380500	1.22511000
H	0.46101300	0.74498000	0.29984200

Zero-point correction= 0.042913 (Hartree/Particle)

Thermal correction to Energy= 0.051802

Thermal correction to Enthalpy= 0.052746

Thermal correction to Gibbs Free Energy= 0.008193

Sum of electronic and zero-point Energies= -426.788413

Sum of electronic and thermal Energies= -426.779524

Sum of electronic and thermal Enthalpies= -426.778580

Sum of electronic and thermal Free Energies= -426.823133

Transition State

C	-0.44429800	0.45299500	0.35016000
O	0.25085700	0.32999100	-1.00921500
O	-0.74671500	-0.59357100	0.85606300
H	0.29480900	-0.63141400	-1.16920300
Na	0.60790300	2.44419000	-1.55526500
O	-0.29123700	3.02155400	0.24582800
H	-0.64839400	3.55219700	0.96654100
H	-0.46485700	1.84041400	0.49458800

Zero-point correction= 0.041092 (Hartree/Particle)

Thermal correction to Energy= 0.048124

Thermal correction to Enthalpy= 0.049068

Thermal correction to Gibbs Free Energy= 0.010003

Sum of electronic and zero-point Energies= -426.771963

Sum of electronic and thermal Energies= -426.764931

Sum of electronic and thermal Enthalpies= -426.763987

Sum of electronic and thermal Free Energies= -426.803052

Product (6)

C	-0.37680200	-0.96268200	0.66295500
O	0.27108800	-0.97442900	-0.59863900
O	-0.68829700	-1.99963100	1.19358600
H	0.35347300	-1.92015000	-0.82836500
Na	0.65452100	1.31837900	-1.17729300

O	-0.22551100	1.83509900	0.57246300
H	-0.50394100	2.54108100	1.16587400
H	-0.48777700	0.12429800	0.95791200

Zero-point correction= 0.046975 (Hartree/Particle)
Thermal correction to Energy= 0.054106
Thermal correction to Enthalpy= 0.055050
Thermal correction to Gibbs Free Energy= 0.015892
Sum of electronic and zero-point Energies= -426.774021
Sum of electronic and thermal Energies= -426.766890
Sum of electronic and thermal Enthalpies= -426.765945
Sum of electronic and thermal Free Energies= -426.805104

IRC

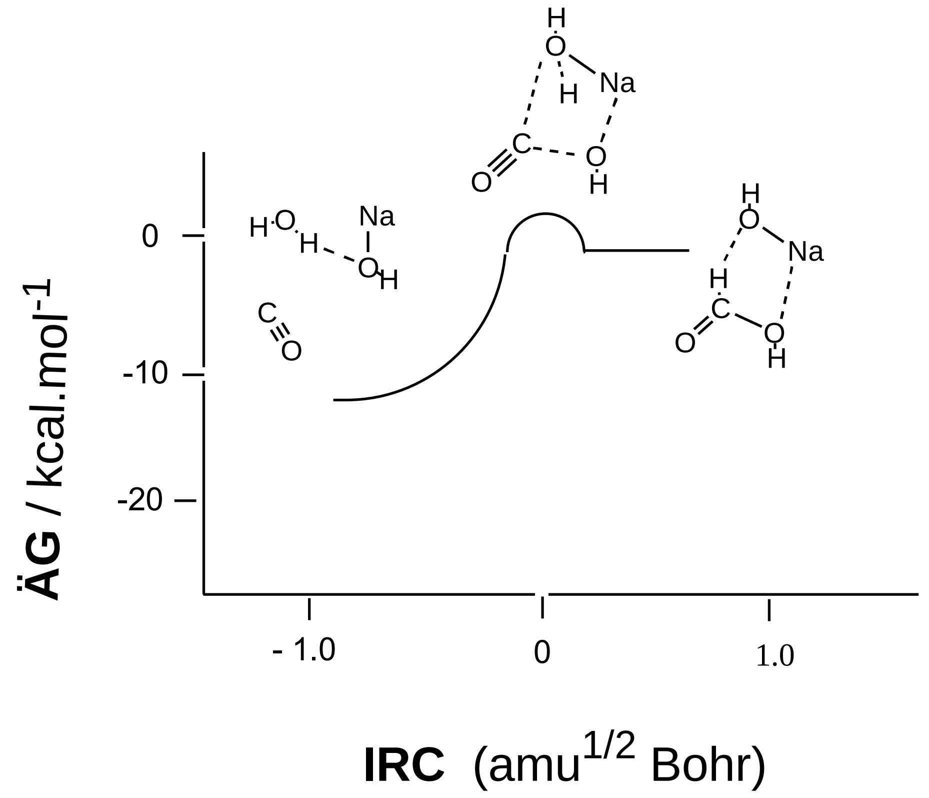

Figure-16

Intermediate (I-22)

C	-0.95406500	-0.17866700	0.65749300
O	-1.07045000	-1.28746400	0.38977000
H	0.66215700	1.55788700	-0.77072400
O	1.42828300	0.97203600	-0.72441900
H	1.66198300	1.04245800	0.20983700

Zero-point correction= 0.028039 (Hartree/Particle)
Thermal correction to Energy= 0.034247
Thermal correction to Enthalpy= 0.035191
Thermal correction to Gibbs Free Energy= -0.001697
Sum of electronic and zero-point Energies= -189.241449
Sum of electronic and thermal Energies= -189.235242
Sum of electronic and thermal Enthalpies= -189.234298
Sum of electronic and thermal Free Energies= -189.271186

Transition State

C	-0.20359800	0.95030900	0.04543100
O	-0.27288300	-0.09575300	0.55874800
H	0.08147400	2.03725700	-0.26615600
O	1.35565600	1.58777700	-0.61828100
H	1.99799100	1.48976600	0.11269200

Zero-point correction= 0.026285 (Hartree/Particle)
Thermal correction to Energy= 0.030071
Thermal correction to Enthalpy= 0.031016
Thermal correction to Gibbs Free Energy= 0.001577
Sum of electronic and zero-point Energies= -189.139172
Sum of electronic and thermal Energies= -189.135386
Sum of electronic and thermal Enthalpies= -189.134441
Sum of electronic and thermal Free Energies= -189.163880

Product (6)

C	-0.33591000	0.25927900	0.01132900
O	-0.46426700	-0.80553500	0.56961700
H	-1.16077500	0.93514400	-0.28861900
O	0.84205500	0.81572700	-0.34643600
H	1.52144100	0.17863700	-0.05641600

Zero-point correction= 0.034228 (Hartree/Particle)
Thermal correction to Energy= 0.037382
Thermal correction to Enthalpy= 0.038326

Thermal correction to Gibbs Free Energy= 0.010139
Sum of electronic and zero-point Energies= -189.247087
Sum of electronic and thermal Energies= -189.243934
Sum of electronic and thermal Enthalpies= -189.242989
Sum of electronic and thermal Free Energies= -189.271176

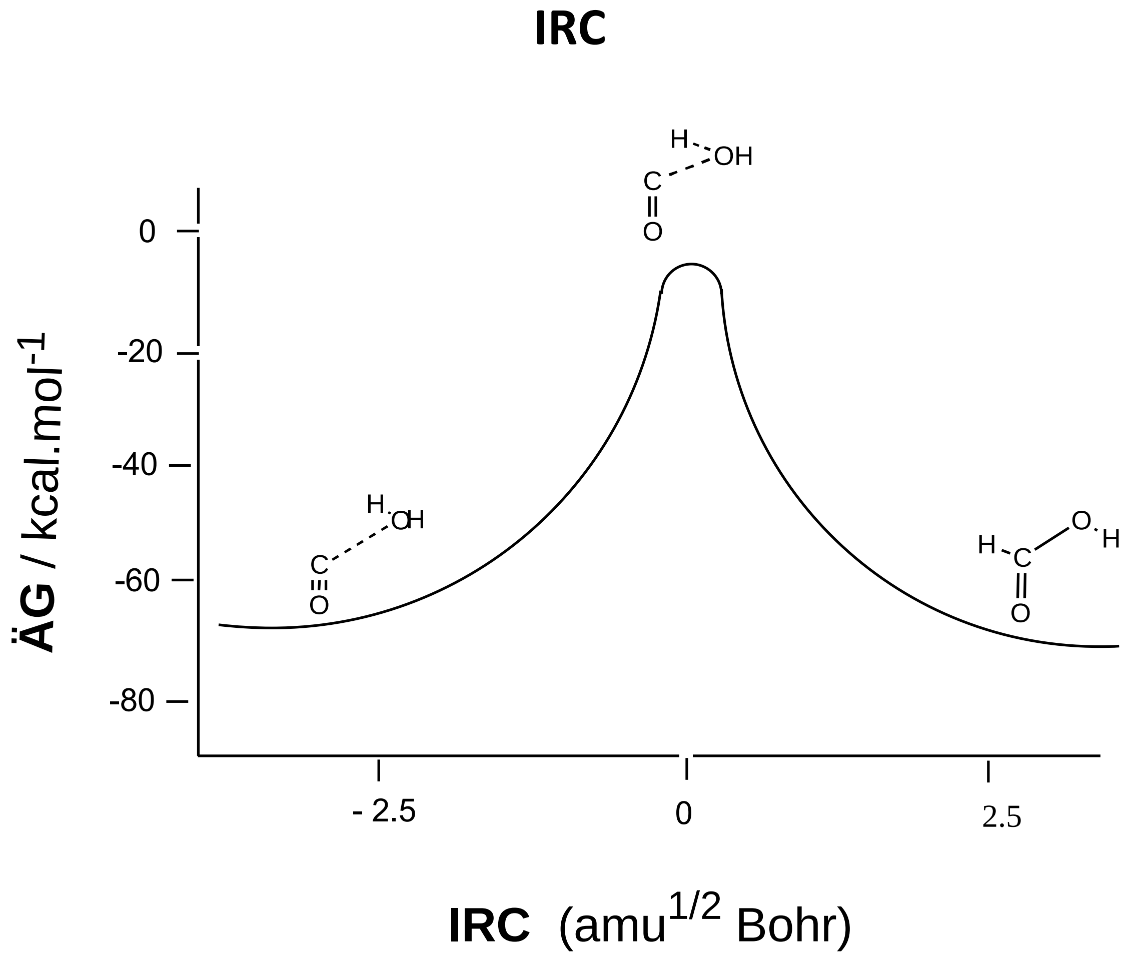

Figure-S1

Intermediate (I-2)

C	0.30845000	0.46631100	0.43178200
H	0.02222700	-0.59155300	0.32823200
Cl	1.97493900	0.55953300	1.02260000
Cl	0.17588900	1.22394600	-1.19123200
Cl	-0.82269200	1.23946700	1.56365900
O	-0.49591500	-2.50588100	0.14105700
H	-0.81802300	-2.38118500	-0.77298700
H	-1.31493200	-2.64825500	0.63376400
O	-1.25624000	-1.70683400	-2.45198700
H	-0.51415500	-1.87664200	-3.04887800
H	-1.20704400	-0.74684900	-2.33306900

Zero-point correction= 0.069651 (Hartree/Particle)

Thermal correction to Energy= 0.080906

Thermal correction to Enthalpy= 0.081850

Thermal correction to Gibbs Free Energy= 0.029061

Sum of electronic and zero-point Energies= -1569.904222

Sum of electronic and thermal Energies= -1569.892967

Sum of electronic and thermal Enthalpies= -1569.892023

Sum of electronic and thermal Free Energies= -1569.944813

Transition State

C	-0.81668600	0.46269700	-0.08717000
H	-1.01949300	0.39420200	-1.16262500
Cl	0.76489500	0.38788400	0.44684900
Cl	-1.05374700	2.01869700	-2.29962600
Cl	-1.98125300	1.10527100	0.93809500
O	-1.39117800	-1.68969200	-0.15061300
H	-1.66640500	-1.58219100	-1.09569500
H	-2.23399600	-1.84051300	0.30437000
O	-2.01755200	-0.92540000	-2.71713200
H	-1.32674500	-1.15179900	-3.35735300
H	-1.99903600	0.05939800	-2.77067300

Zero-point correction= 0.068300 (Hartree/Particle)

Thermal correction to Energy= 0.078297

Thermal correction to Enthalpy= 0.079241

Thermal correction to Gibbs Free Energy= 0.031017

Sum of electronic and zero-point Energies= -1569.819238

Sum of electronic and thermal Energies= -1569.809240

Sum of electronic and thermal Enthalpies= -1569.808296

Sum of electronic and thermal Free Energies= -1569.856521

Product (1)

C	-0.33591000	0.25927900	0.01132900
C	0.09636800	-0.37906400	0.80738700
H	0.22100700	0.26301800	-0.07188700
Cl	1.64002300	-0.39021500	1.71796500
Cl	-0.31209400	1.25549900	-2.54409100
Cl	-1.20009400	0.32273900	1.82488100
O	-0.23591400	-1.64720600	0.36649100
H	-1.04893900	-1.78730800	-1.38122500
H	-0.38341200	-2.19936900	1.15354900
O	-1.23180800	-1.62485700	-2.32369000
H	-0.57497600	-2.18056700	-2.76698200
H	-0.77857800	0.02024900	-2.53759500

Zero-point correction= 0.069189 (Hartree/Particle)
Thermal correction to Energy= 0.079429
Thermal correction to Enthalpy= 0.080373
Thermal correction to Gibbs Free Energy= 0.029199
Sum of electronic and zero-point Energies= -1569.918592
Sum of electronic and thermal Energies= -1569.908352
Sum of electronic and thermal Enthalpies= -1569.907408
Sum of electronic and thermal Free Energies= -1569.958582

IRC

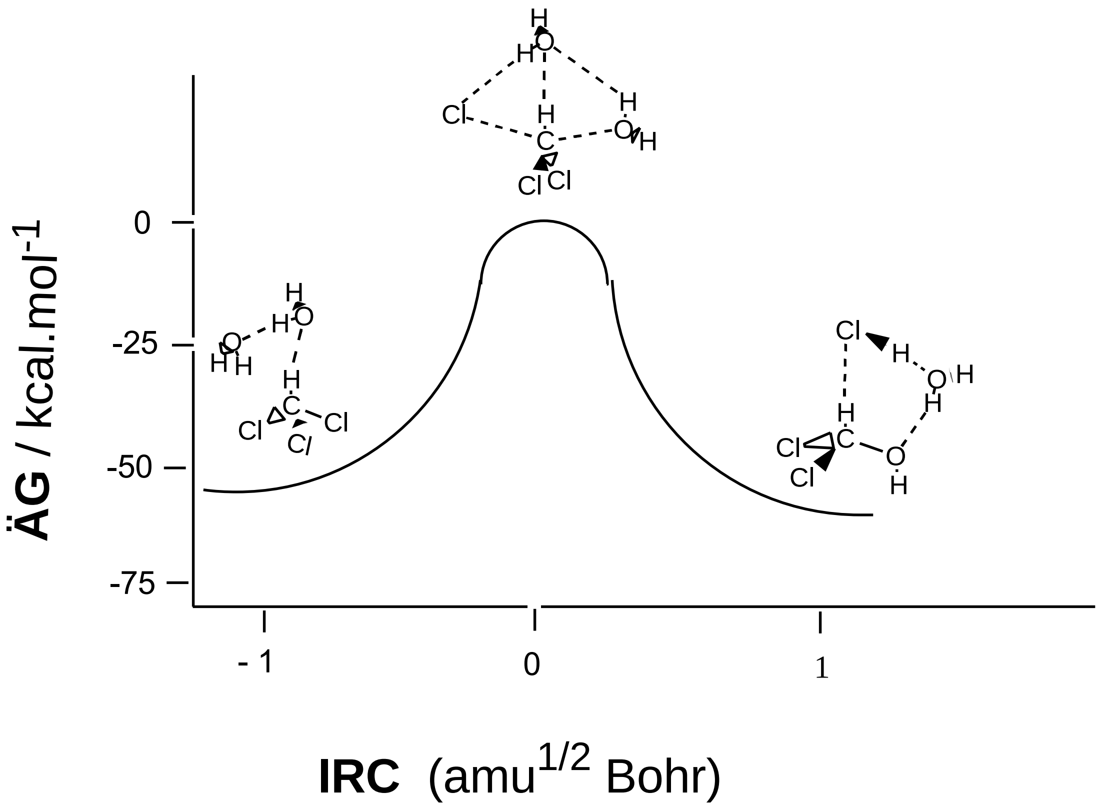

Figure-S2

Intermediate (I-4)

C	-0.52962500	0.20200900	0.11202600
H	0.32208300	0.37674500	0.85999300
Cl	0.13759900	-1.03335500	-1.06968800
Cl	-1.96248700	-0.47920600	0.90311700
Cl	-0.93056000	1.68380400	-0.77504300
O	1.75095000	0.29296100	1.62575300
H	1.80091200	0.87023000	2.39548200
Na	2.59605400	-1.06146400	0.39116500

Zero-point correction= 0.032303 (Hartree/Particle)
Thermal correction to Energy= 0.041246
Thermal correction to Enthalpy= 0.042190
Thermal correction to Gibbs Free Energy= -0.003854
Sum of electronic and zero-point Energies= -1655.000201
Sum of electronic and thermal Energies= -1654.991258
Sum of electronic and thermal Enthalpies= -1654.990314
Sum of electronic and thermal Free Energies= -1655.036357

Transition State

C	-0.22937000	0.03903200	0.51213400
H	-0.61733800	0.06233000	1.52220200
Cl	0.16854800	-1.49654200	-0.28121400
Cl	-2.43206900	-0.09921000	0.05214900
Cl	0.01632700	1.51158100	-0.44819900
O	1.49717000	0.11209500	1.31496000
H	1.39536000	0.88765900	1.89043300
Na	2.57486200	-0.06031600	-0.51149000

Zero-point correction= 0.032795 (Hartree/Particle)
Thermal correction to Energy= 0.040818
Thermal correction to Enthalpy= 0.041763
Thermal correction to Gibbs Free Energy= -0.001248
Sum of electronic and zero-point Energies= -1654.938433
Sum of electronic and thermal Energies= -1654.930409
Sum of electronic and thermal Enthalpies= -1654.929465
Sum of electronic and thermal Free Energies= -1654.972475

Product (1)

C	0.50469100	-0.10910100	0.09129600
H	0.41546600	0.83048200	-0.47259500
Cl	2.14404300	-0.24614700	0.74968000
Cl	-2.08522100	-0.53875200	-2.22729200

Cl	-0.66358500	0.15289100	1.53919600
O	0.16400200	-1.21590400	-0.60090700
H	-0.48344400	-0.92059100	-1.34900700
Na	-2.27753200	-1.62901500	0.06618300

Zero-point correction= 0.035454 (Hartree/Particle)
Thermal correction to Energy= 0.043581
Thermal correction to Enthalpy= 0.044526
Thermal correction to Gibbs Free Energy= 0.000015
Sum of electronic and zero-point Energies= -1655.075062
Sum of electronic and thermal Energies= -1655.066934
Sum of electronic and thermal Enthalpies= -1655.065990
Sum of electronic and thermal Free Energies= -1655.110500

IRC

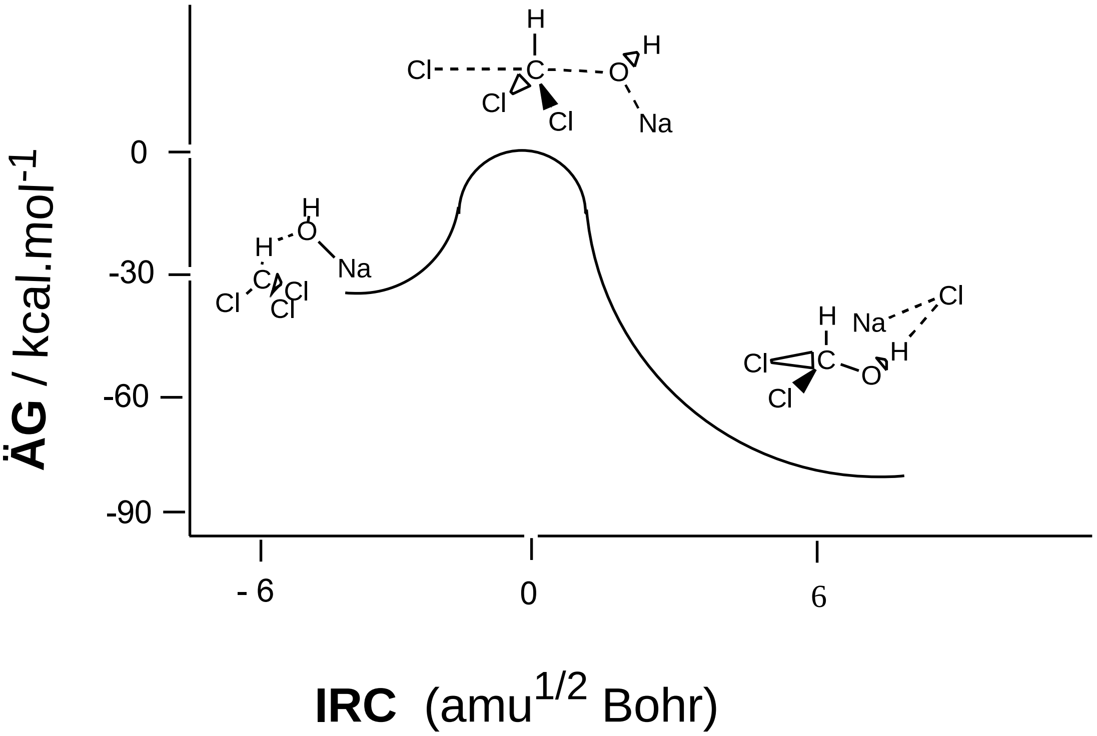

Figure-S3

Intermediate (I-6)

C	-0.48144000	0.42778200	0.58504100
H	0.42518700	0.71127800	-0.02652300
Cl	-0.17165600	-1.26782700	1.16574900
Cl	-0.68859000	1.50169900	1.97613100
Cl	-1.92445300	0.43360300	-0.45533300
O	1.79969700	0.58859100	-1.02923500
H	2.28684800	1.41275300	-1.15010500
Na	1.92941100	-1.53060700	-0.83905600
O	0.74531700	-0.95239800	-2.73139400
H	-0.21171000	-0.82321100	-2.74850600
H	1.08954000	-0.11873600	-2.25038400

Zero-point correction= 0.058725 (Hartree/Particle)

Thermal correction to Energy= 0.069817

Thermal correction to Enthalpy= 0.070761

Thermal correction to Gibbs Free Energy= 0.019873

Sum of electronic and zero-point Energies= -1731.246979

Sum of electronic and thermal Energies= -1731.235887

Sum of electronic and thermal Enthalpies= -1731.234943

Sum of electronic and thermal Free Energies= -1731.285831

Transition State

C	-0.83166800	-0.00804300	1.15134800
H	-0.21197900	0.67764300	1.71325000
Cl	-0.78557200	-1.74206500	1.52574600
Cl	-2.08218000	0.47051100	3.05470800
Cl	-2.05057000	0.53495900	0.02982600
O	0.53629200	-0.14290200	-0.12886400
H	0.73287400	0.79548900	-0.28019400
Na	1.29615500	-2.18801700	-0.27912100
O	0.02999000	-1.80453000	-2.15827600
H	-0.88555100	-2.04099400	-2.35604000
H	-0.04979400	-0.97172000	-1.62925500

Zero-point correction= 0.058554 (Hartree/Particle)

Thermal correction to Energy= 0.069247

Thermal correction to Enthalpy= 0.070192

Thermal correction to Gibbs Free Energy= 0.020624

Sum of electronic and zero-point Energies= -1731.179338

Sum of electronic and thermal Energies= -1731.168646

Sum of electronic and thermal Enthalpies= -1731.167701

Sum of electronic and thermal Free Energies= -1731.217269

Product (2)

C	-1.04210100	0.51289100	-0.09729100
H	-1.41015800	-0.05865300	0.76795100
Cl	0.39247400	-2.05112200	-0.03044300
Cl	2.67647800	1.74060900	1.32080800
Cl	-2.06271000	0.27655100	-1.51050100
O	-0.10308100	1.27806600	-0.11160300
H	1.39092100	1.74505500	1.13650500
Na	2.08408800	-0.23798500	-0.69894600
O	1.25905100	-0.89513600	-2.76682400
H	0.76101300	-1.52291800	-2.19355300
H	0.55842600	-0.45761900	-3.26877600

Zero-point correction= 0.055857 (Hartree/Particle)
Thermal correction to Energy= 0.068711
Thermal correction to Enthalpy= 0.069655
Thermal correction to Gibbs Free Energy= 0.013088
Sum of electronic and zero-point Energies= -1731.319749
Sum of electronic and thermal Energies= -1731.306895
Sum of electronic and thermal Enthalpies= -1731.305951
Sum of electronic and thermal Free Energies= -1731.362517

IRC

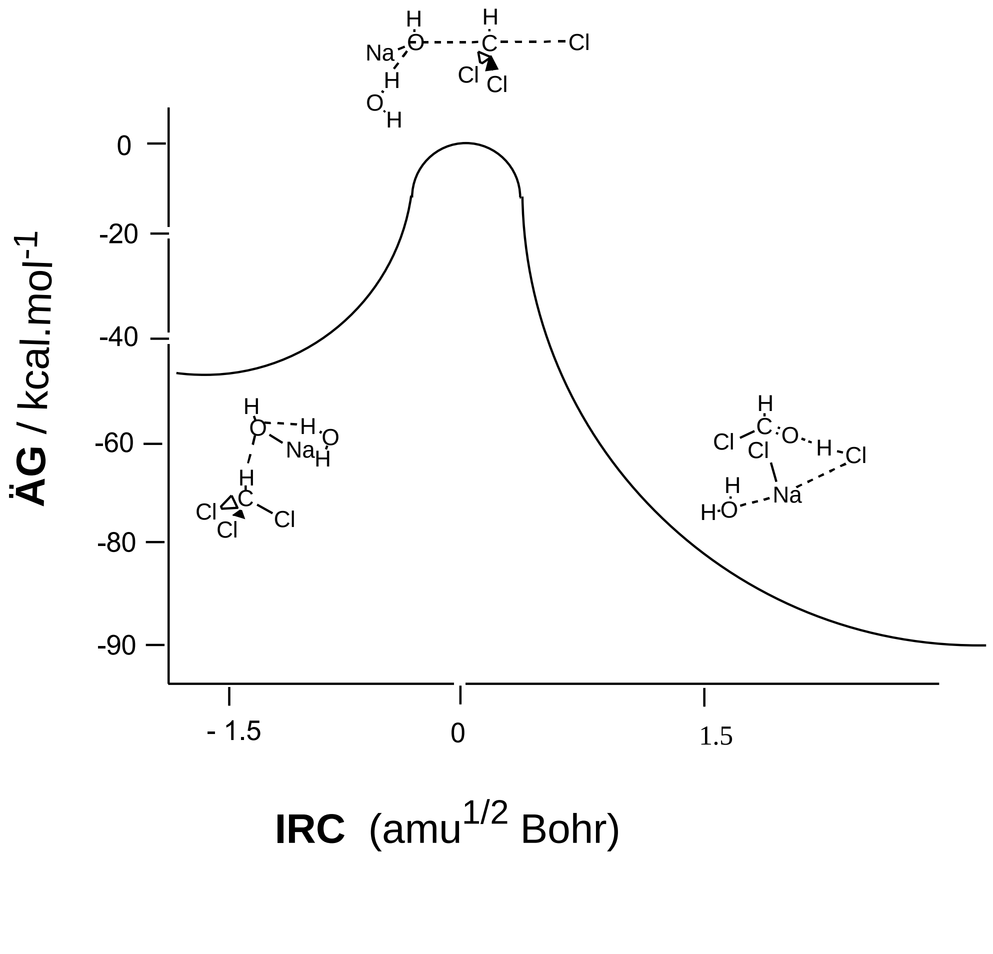

Figure-S5

Intermediate-(I-10)

C	-0.13880900	-0.13221400	0.29530800
H	-0.88019400	-0.89475700	0.02252000
Cl	1.45259800	-0.70322700	-0.27047000
Cl	-0.57509800	1.39861100	-0.50139100
Cl	-0.15434500	0.04772600	2.06351700
O	-2.05603500	-2.51797100	-0.36340100
H	-2.40028400	-2.92782500	0.44156600
O	2.36882800	1.82680700	-2.54498600
H	1.45483900	1.51885400	-2.52176100
H	2.61263300	1.74102400	-1.61537100
H	-1.55297300	-3.23827700	-0.76672300

Zero-point correction= 0.066544 (Hartree/Particle)

Thermal correction to Energy= 0.079916

Thermal correction to Enthalpy= 0.080861

Thermal correction to Gibbs Free Energy= 0.020035

Sum of electronic and zero-point Energies= -1569.893777

Sum of electronic and thermal Energies= -1569.880405

Sum of electronic and thermal Enthalpies= -1569.879460

Sum of electronic and thermal Free Energies= -1569.940286

Transition State-1

C	-1.12382600	0.06458900	0.42571500
H	-1.69535500	-1.21283500	-0.20011700
Cl	0.90441600	-1.58349100	-0.81878100
Cl	-0.92594400	1.47827000	-0.45126900
Cl	-0.55930900	0.15300900	2.02998800
O	-1.76257900	-2.15683200	-0.77351100
H	-1.83890100	-2.85704700	-0.10407500
O	1.35388700	1.27552300	-2.43261300
H	1.23305500	0.34656700	-2.17517700
H	2.27484000	1.41901900	-2.18123200
H	-0.65910500	-2.09878600	-0.95616800

Zero-point correction= 0.062197 (Hartree/Particle)

Thermal correction to Energy= 0.072744

Thermal correction to Enthalpy= 0.073688

Thermal correction to Gibbs Free Energy= 0.023625

Sum of electronic and zero-point Energies= -1569.806617

Sum of electronic and thermal Energies= -1569.796071

Sum of electronic and thermal Enthalpies= -1569.795127

Sum of electronic and thermal Free Energies= -1569.845190

Intermediate-(I-11)

C	-1.14146400	0.39989400	1.12918400
H	-1.43945100	-1.42309000	0.30434100
Cl	0.80086800	-1.84478700	-2.30567700
Cl	0.02117100	1.19836900	0.18609800
Cl	-1.74692500	1.40126500	2.38817000
O	-1.32162100	-2.23300900	-0.24087800
H	-0.99987100	-2.88070200	0.40253500
O	1.88581100	1.47100100	-2.07689900
H	1.49284300	0.63001400	-2.35387800
H	2.80226700	1.21052000	-1.91908800
H	-0.16004600	-1.99665900	-1.39470400

Zero-point correction= 0.063244 (Hartree/Particle)
Thermal correction to Energy= 0.075794
Thermal correction to Enthalpy= 0.076738
Thermal correction to Gibbs Free Energy= 0.020048
Sum of electronic and zero-point Energies= -1569.816314
Sum of electronic and thermal Energies= -1569.803764
Sum of electronic and thermal Enthalpies= -1569.802820
Sum of electronic and thermal Free Energies= -1569.859510

IRC

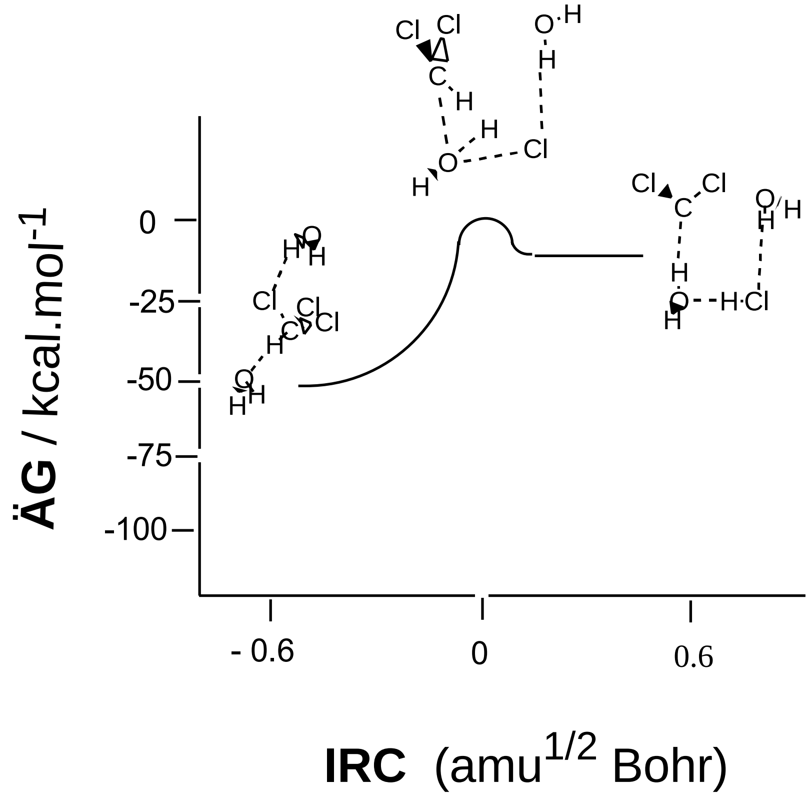

Transition State-2

C	-0.32254800	-0.14611600	0.14301200
H	-0.82776800	-0.53550200	-1.00480400
Cl	1.13074800	-0.99786700	0.61692400
Cl	-1.19705300	2.17959400	-1.96293500
Cl	-1.07420100	0.67143700	1.52183900
O	-1.34706800	-1.34829200	-0.35967000
H	-2.24359000	-0.87634600	-0.34309000
O	-3.53401500	0.12279100	-0.58151500
H	-3.24417900	0.82298400	-1.19116700
H	-3.70728000	0.61648900	0.23389000
H	-0.58169000	1.29815200	-1.18179700

Zero-point correction= 0.062237 (Hartree/Particle)
Thermal correction to Energy= 0.072245
Thermal correction to Enthalpy= 0.073190
Thermal correction to Gibbs Free Energy= 0.025326
Sum of electronic and zero-point Energies= -1569.796959
Sum of electronic and thermal Energies= -1569.786951
Sum of electronic and thermal Enthalpies= -1569.786007
Sum of electronic and thermal Free Energies= -1569.833871

Product (1)

C	0.40471500	-0.99630200	0.18157900
H	0.47739000	-0.71545300	-0.88099500
Cl	1.79285600	-2.02310600	0.57554600
Cl	-0.54584600	2.15332300	-2.00144700
Cl	0.61926100	0.64168200	1.05615800
O	-0.73411900	-1.61077000	0.54515900
H	-1.47332300	-1.11572200	0.10560800
O	-2.63820700	-0.12726500	-0.63178900
H	-2.30193200	0.46336700	-1.32385100
H	-3.01815100	0.49110300	0.00864800
H	-0.00892400	1.85972000	-0.85686500

Zero-point correction= 0.068316 (Hartree/Particle)
Thermal correction to Energy= 0.078889
Thermal correction to Enthalpy= 0.079833
Thermal correction to Gibbs Free Energy= 0.029420
Sum of electronic and zero-point Energies= -1569.916934
Sum of electronic and thermal Energies= -1569.906362
Sum of electronic and thermal Enthalpies= -1569.905417
Sum of electronic and thermal Free Energies= -1569.955830

IRC

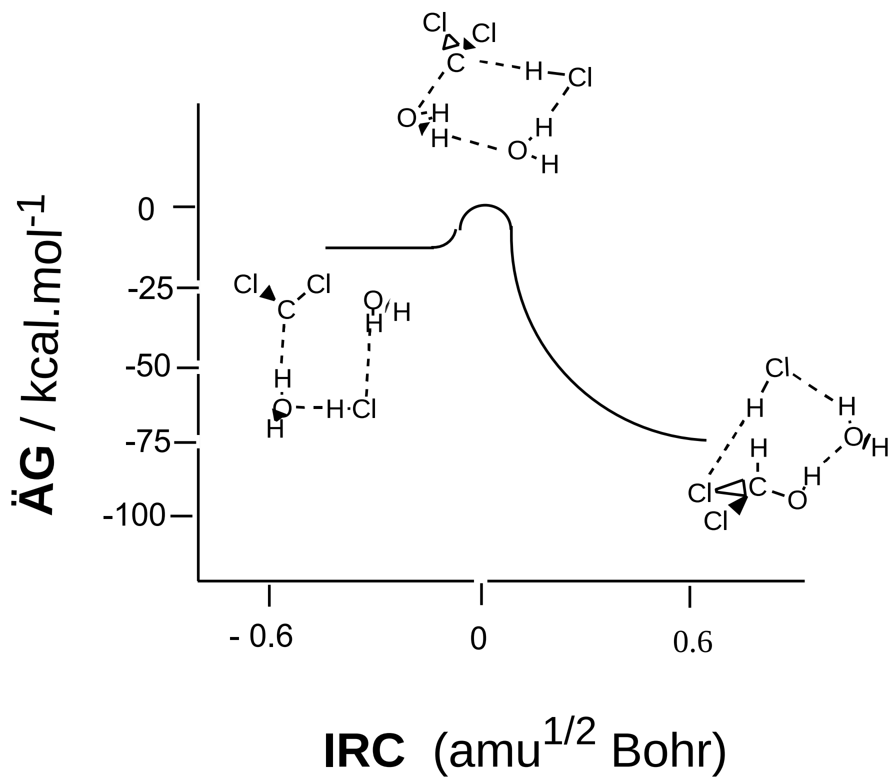

Figure-S6

Intermediate (I-17)

C	1.32648600	0.52629900	-0.93855900
H	0.24453000	0.41669700	-1.16705200
Cl	1.64711700	-0.31419600	0.67813300
O	2.20127400	1.05864700	-1.53981800
O	-1.68805100	-0.01705100	-1.24912300
H	-2.27993300	0.73297800	-1.10681100
H	-1.79515700	-0.53036000	-0.42303000
O	-1.57097000	-1.34028300	1.21012100
H	-1.47896100	-2.29955800	1.12935100
H	-0.65966000	-1.05873600	1.38642400

Zero-point correction= 0.069036 (Hartree/Particle)
Thermal correction to Energy= 0.078630
Thermal correction to Enthalpy= 0.079574
Thermal correction to Gibbs Free Energy= 0.033278
Sum of electronic and zero-point Energies= -725.681423
Sum of electronic and thermal Energies= -725.671829
Sum of electronic and thermal Enthalpies= -725.670885
Sum of electronic and thermal Free Energies= -725.717182

Transition State

C	1.22994100	0.00369800	1.27658700
H	0.01298300	0.28294000	1.13957600
Cl	0.78812700	1.31128300	3.35725300
O	2.32125200	-0.29942000	1.09500000
O	-1.21391800	0.76065800	1.36847400
H	-1.31503300	1.59310900	0.87876700
H	-0.88089500	1.07503000	2.26413000
O	0.15998800	-1.70448500	2.82159100
H	0.82935500	-2.30928800	3.16942900
H	0.25270500	-0.93377400	3.41225900

Zero-point correction= 0.065010 (Hartree/Particle)
Thermal correction to Energy= 0.073346
Thermal correction to Enthalpy= 0.074291
Thermal correction to Gibbs Free Energy= 0.032251
Sum of electronic and zero-point Energies= -725.651859
Sum of electronic and thermal Energies= -725.643523
Sum of electronic and thermal Enthalpies= -725.642579
Sum of electronic and thermal Free Energies= -725.684619

Product (4)

```
C                 0.95543700   -0.34238100   -2.00586500
H                -1.55188400   -0.49064100   -0.35725500
Cl               -0.01743000    1.62954700    1.23947600
O                 2.02804600   -0.73366700   -1.91131500
O                -1.85908300    0.37787000   -0.69439300
H                -1.40649500    0.42538500   -1.54991700
H                -0.91509300    1.20603000    0.33736600
O                -0.43469000   -1.63057100    0.55798500
H                 0.00720400   -0.95473800    1.09751800
H                -0.79144100   -2.23886000    1.21911800
```

Zero-point correction= 0.064544 (Hartree/Particle)
Thermal correction to Energy= 0.075142
Thermal correction to Enthalpy= 0.076086
Thermal correction to Gibbs Free Energy= 0.026652
Sum of electronic and zero-point Energies= -725.704485
Sum of electronic and thermal Energies= -725.693887
Sum of electronic and thermal Enthalpies= -725.692943
Sum of electronic and thermal Free Energies= -725.742377

IRC

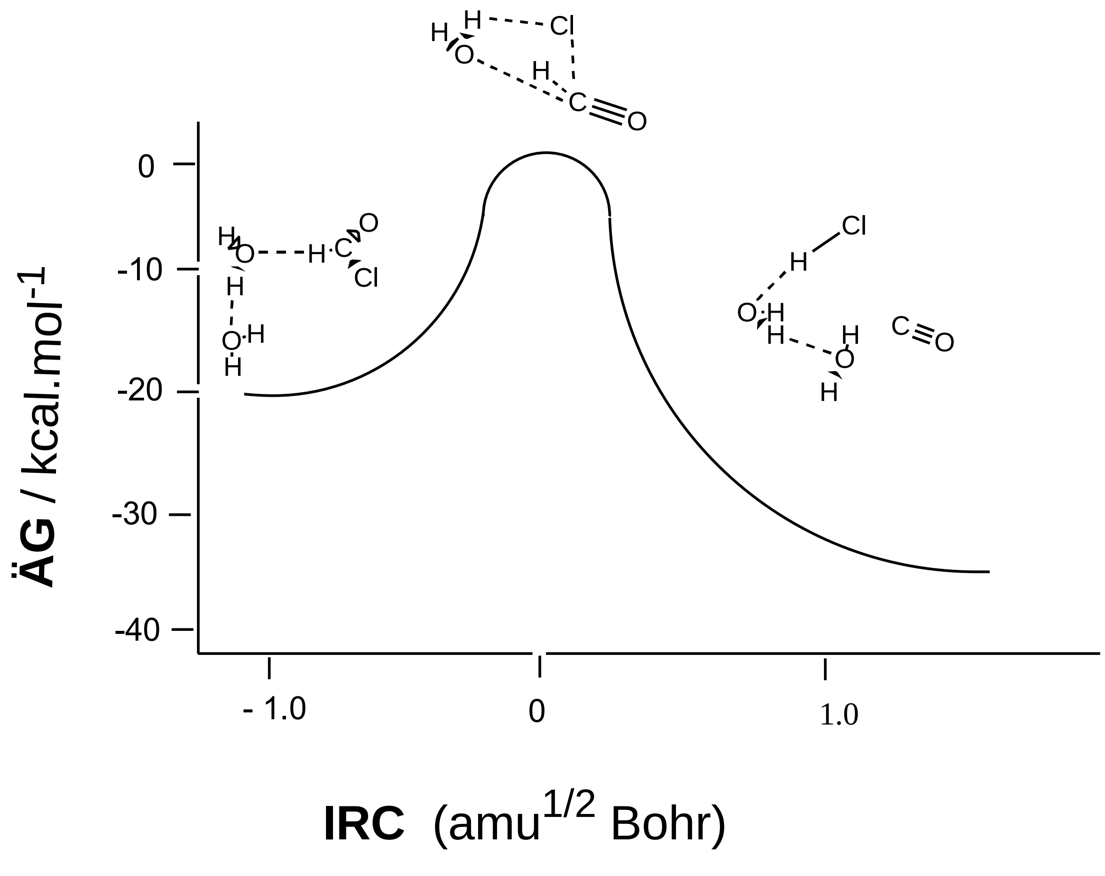

Figure-S7

Intermediate (I-23)

C	-0.51898800	-0.84239700	1.07896100
O	0.38069100	-0.09348900	-1.65421600
O	-0.79399000	-1.94574800	0.94127300
H	1.30293600	-0.33379100	-1.80578600
O	0.43816900	2.08637700	0.20738500
H	-0.41914400	2.52604800	0.12465400
H	0.23774400	1.36807100	0.82632900
H	0.46866700	0.75816600	-1.19246200

Zero-point correction= 0.053964 (Hartree/Particle)

Thermal correction to Energy= 0.062687

Thermal correction to Enthalpy= 0.063631

Thermal correction to Gibbs Free Energy= 0.021200

Sum of electronic and zero-point Energies= -265.459680

Sum of electronic and thermal Energies= -265.450956

Sum of electronic and thermal Enthalpies= -265.450012

Sum of electronic and thermal Free Energies= -265.492444

Transition State

C	-0.63929600	0.41428700	0.66248800
O	-0.14553300	0.88858400	-1.00425200
O	-0.82085700	-0.72377500	0.85344800
H	0.54932900	0.28252400	-1.31100100
O	0.06784600	2.76204800	0.39100700
H	-0.67631800	3.33579300	0.15075600
H	-0.42246300	1.74424400	0.89035700
H	0.15839100	2.02534900	-0.47784300

Zero-point correction= 0.051205 (Hartree/Particle)

Thermal correction to Energy= 0.056246

Thermal correction to Enthalpy= 0.057190

Thermal correction to Gibbs Free Energy= 0.023743

Sum of electronic and zero-point Energies= -265.387722

Sum of electronic and thermal Energies= -265.382681

Sum of electronic and thermal Enthalpies= -265.381737

Sum of electronic and thermal Free Energies= -265.415184

Product (6)

C	-0.20638100	-0.71363100	0.27493400
O	0.33002400	-0.30299500	-0.90821800
O	-0.53018000	-1.85176800	0.51765700
H	0.38948900	-1.10734500	-1.45662900

O	0.45532500	2.40927300	0.23149500
H	-0.35293200	2.73340300	-0.18647400
H	-0.28464400	0.16190100	0.94425600
H	0.78847900	1.79732000	-0.43960900

Zero-point correction= 0.058516 (Hartree/Particle)
Thermal correction to Energy= 0.065214
Thermal correction to Enthalpy= 0.066158
Thermal correction to Gibbs Free Energy= 0.028377
Sum of electronic and zero-point Energies= -265.460580
Sum of electronic and thermal Energies= -265.453883
Sum of electronic and thermal Enthalpies= -265.452938
Sum of electronic and thermal Free Energies= -265.490720

IRC

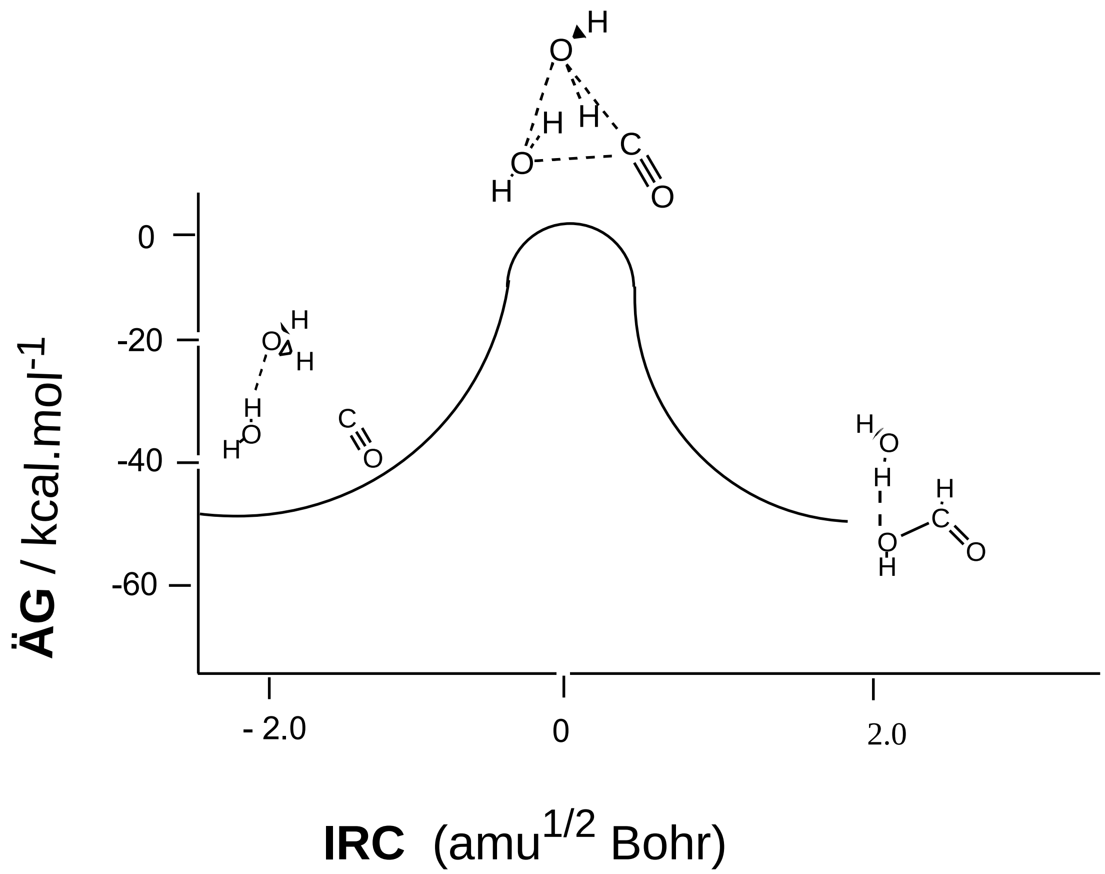

Calculations at the M06-2X/cc-pVTZ level

Table S3. Gas-phase Cartesian coordinates of all Reactants and Products along with all corresponding calculated Energy values at the M06-2X/c-pVTZ level.

A-Reactants

$CHCl_3$

C	-2.59767100	-2.63668600	0.02320600
H	-2.23743100	-2.12718900	-0.85873400
Cl	-1.98630800	-1.77285200	1.43599200
Cl	-4.36215800	-2.61258600	-0.01873500
Cl	-1.98657600	-4.29214900	-0.01834500

Zero-point correction= 0.020175 (Hartree/Particle)
Thermal correction to Energy= 0.024596
Thermal correction to Enthalpy= 0.025540
Thermal correction to Gibbs Free Energy= -0.008984
Sum of electronic and zero-point Energies= -1419.288771
Sum of electronic and thermal Energies= -1419.284351
Sum of electronic and thermal Enthalpies= -1419.283406
Sum of electronic and thermal Free Energies= -1419.317931

NaOH

H	-1.98204000	-2.24885400	-0.64846700
Na	-4.71362800	-2.71304900	0.15560100
O	-2.88174400	-2.40339000	-0.38078500

Zero-point correction= 0.010944 (Hartree/Particle)
Thermal correction to Energy= 0.014495
Thermal correction to Enthalpy= 0.015440
Thermal correction to Gibbs Free Energy= -0.007197
Sum of electronic and zero-point Energies= -238.096719
Sum of electronic and thermal Energies= -238.093167
Sum of electronic and thermal Enthalpies= -238.092223
Sum of electronic and thermal Free Energies= -238.114860

H_2O

H	-2.29326100	-2.15097400	-0.81799400
O	-2.55794500	-2.61179600	-0.01982100
H	-3.51620600	-2.60253800	-0.03583600

Zero-point correction= 0.021602 (Hartree/Particle)
Thermal correction to Energy= 0.024438
Thermal correction to Enthalpy= 0.025382
Thermal correction to Gibbs Free Energy= 0.003315

Sum of electronic and zero-point Energies= -76.403672
Sum of electronic and thermal Energies= -76.400837
Sum of electronic and thermal Enthalpies= -76.399892
Sum of electronic and thermal Free Energies= -76.421960

$PhCCl_3$

C	-1.83075700	-0.50962000	-0.12000900
Cl	-0.87786700	-1.99989300	-0.27459500
Cl	-3.15735900	-0.58588500	-1.31785800
C	-1.01845600	0.75268000	-0.32838400
C	0.34722000	0.73385900	-0.56298800
C	1.03714800	1.92865700	-0.73603000
C	0.36949800	3.13833400	-0.67525900
C	-1.00073400	3.15698800	-0.43874000
C	-1.69078600	1.97299300	-0.26590500
H	0.88006300	-0.20259400	-0.61244100
H	2.10232700	1.90315400	-0.91893600
H	0.90940700	4.06527300	-0.81056900
H	-1.53161800	4.09739600	-0.38896600
H	-2.75639600	1.98739600	-0.08118100
Cl	-2.55923500	-0.49297400	1.51429500

Zero-point correction= 0.100973 (Hartree/Particle)
Thermal correction to Energy= 0.110007
Thermal correction to Enthalpy= 0.110951
Thermal correction to Gibbs Free Energy= 0.064854
Sum of electronic and zero-point Energies= -1650.240275
Sum of electronic and thermal Energies= -1650.231241
Sum of electronic and thermal Enthalpies= -1650.230297
Sum of electronic and thermal Free Energies= -1650.276394

B-Products

$CH(OH)Cl_2$ (1)

C	0.17835500	0.11352100	0.67264500
H	-0.29686700	-0.48219000	-0.09455900
Cl	1.49156300	-0.87917700	1.38383200
Cl	-1.05029500	0.43183800	1.93978200
O	0.64639300	1.25630600	0.11742500
H	1.07210900	1.79377900	0.79647400

Zero-point correction= 0.034395 (Hartree/Particle)
Thermal correction to Energy= 0.038864
Thermal correction to Enthalpy= 0.039808
Thermal correction to Gibbs Free Energy= 0.006024
Sum of electronic and zero-point Energies= -1034.906483

Sum of electronic and thermal Energies= -1034.902014
Sum of electronic and thermal Enthalpies= -1034.901070
Sum of electronic and thermal Free Energies= -1034.934854

HCOCl (2)

C	-0.85384200	-1.21064500	-0.25844100
H	-0.71698300	-0.27633100	-0.81075300
Cl	0.45364100	-1.42212100	0.91470700
O	-1.72917300	-1.98224400	-0.39388800

Zero-point correction= 0.019439 (Hartree/Particle)
Thermal correction to Energy= 0.022663
Thermal correction to Enthalpy= 0.023607
Thermal correction to Gibbs Free Energy= -0.005747
Sum of electronic and zero-point Energies= -574.106566
Sum of electronic and thermal Energies= -574.103342
Sum of electronic and thermal Enthalpies= -574.102397
Sum of electronic and thermal Free Energies= -574.131752

CO (4)

C	-0.81998200	-1.16647400	-0.26167800
O	-1.64139400	-1.92313900	-0.36778600

Zero-point correction= 0.005183 (Hartree/Particle)
Thermal correction to Energy= 0.007543
Thermal correction to Enthalpy= 0.008487
Thermal correction to Gibbs Free Energy= -0.013929
Sum of electronic and zero-point Energies= -113.313682
Sum of electronic and thermal Energies= -113.311321
Sum of electronic and thermal Enthalpies= -113.310377
Sum of electronic and thermal Free Energies= -113.332793

$HCCl(OH)_2$ (5)

C	-0.89674900	-0.83911300	0.28145100
H	-1.42685700	-1.71393100	0.63657700
Cl	0.76606000	-0.89055100	1.05072800
O	-0.76638600	-0.94111300	-1.07761500
O	-1.56339500	0.27152500	0.72389700
H	-0.12675800	-0.29516800	-1.39652500
H	-1.02012800	1.05610400	0.59185700

Zero-point correction= 0.048070 (Hartree/Particle)
Thermal correction to Energy= 0.052896

Thermal correction to Enthalpy= 0.053840
Thermal correction to Gibbs Free Energy= 0.020014
Sum of electronic and zero-point Energies= -650.520251
Sum of electronic and thermal Energies= -650.515424
Sum of electronic and thermal Enthalpies= -650.514480
Sum of electronic and thermal Free Energies= -650.548306

HCOOH (6)

```
C     0.25912600    0.10604700    0.74152100
H    -0.30258400   -0.55239300    0.07043700
O     0.58139000    1.23939200    0.10817100
H     1.07395400    1.79201800    0.73193300
O     0.54381900   -0.14742700    1.87168300
```

Zero-point correction= 0.034343 (Hartree/Particle)
Thermal correction to Energy= 0.037501
Thermal correction to Enthalpy= 0.038445
Thermal correction to Gibbs Free Energy= 0.010280
Sum of electronic and zero-point Energies= -189.731439
Sum of electronic and thermal Energies= -189.728281
Sum of electronic and thermal Enthalpies= -189.727336
Sum of electronic and thermal Free Energies= -189.755501

Table S4. Gas-phase Cartesian coordinates of all stationary points along with all corresponding calculated Energy values and the IRC plots found in the Potential Energy Surfaces shown in the Figures.

Figure-1

Intermediate (I-1)

C	0.25348400	-0.00934800	0.16271900
H	0.40661700	-1.07556100	0.24615200
Cl	1.60351100	0.84520900	0.90287900
Cl	0.13185900	0.37398700	-1.56177300
Cl	-1.26576000	0.37243200	0.98851600
O	-1.07703000	-2.62353900	-0.56991700
H	-1.14162300	-2.31610800	-1.47776300
H	-1.87699300	-2.30422300	-0.14445700

Zero-point correction= 0.043710 (Hartree/Particle)
Thermal correction to Energy= 0.052158
Thermal correction to Enthalpy= 0.053102
Thermal correction to Gibbs Free Energy= 0.008510
Sum of electronic and zero-point Energies= -1495.699983
Sum of electronic and thermal Energies= -1495.691535
Sum of electronic and thermal Enthalpies= -1495.690591
Sum of electronic and thermal Free Energies= -1495.735183

Transition State

C	-0.68364800	0.93923500	0.41793200
H	-1.04202300	1.04685800	-0.97406400
Cl	0.85354600	0.46306300	0.87637500
Cl	-1.41303800	0.89880700	-2.41285700
Cl	-1.72071800	1.27711500	1.69912800
O	-1.46064700	-1.30453300	-0.18256500
H	-1.47180700	-1.03184500	-1.11391100
H	-2.37607300	-1.51316800	0.02235900

Zero-point correction= 0.036940 (Hartree/Particle)
Thermal correction to Energy= 0.044860
Thermal correction to Enthalpy= 0.045804
Thermal correction to Gibbs Free Energy= 0.002272
Sum of electronic and zero-point Energies= -1495.617077
Sum of electronic and thermal Energies= -1495.609157
Sum of electronic and thermal Enthalpies= -1495.608213
Sum of electronic and thermal Free Energies= -1495.651746

Product (1)

C	0.14805000	-0.10050700	0.41026700
H	0.51229100	0.52764900	-0.39243200
Cl	1.47289100	-0.31726000	1.58669300
Cl	-1.18137500	0.19706000	-2.84727200
Cl	-1.20777400	0.75712200	1.19450400
O	-0.25216300	-1.29368100	-0.12127200
H	-1.06927900	-0.72375600	-1.95738200
H	-0.59188600	-1.86344600	0.58018700

Zero-point correction= 0.043344 (Hartree/Particle)
Thermal correction to Energy= 0.050840
Thermal correction to Enthalpy= 0.051784
Thermal correction to Gibbs Free Energy= 0.008331
Sum of electronic and zero-point Energies= -1495.709878
Sum of electronic and thermal Energies= -1495.702382
Sum of electronic and thermal Enthalpies= -1495.701438
Sum of electronic and thermal Free Energies= -1495.744891

IRC

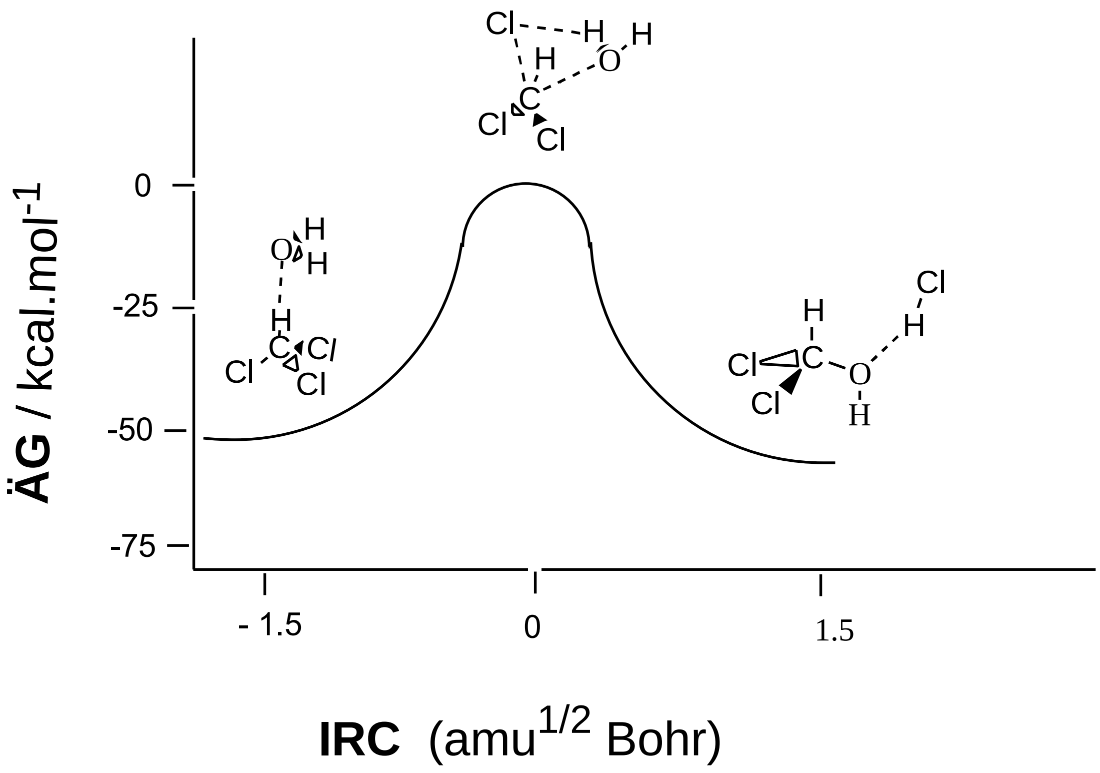

Figure-2

Intermediate (I-3)

C	0.63423800	0.39864200	0.36567300
H	0.36740500	-0.60133500	0.74824100
Cl	1.55501800	1.31622300	1.54841100
Cl	1.52965600	0.22350500	-1.14587700
Cl	-0.93222800	1.22832600	0.04250700
O	-0.60775900	-2.09458600	0.91872500
H	-0.61340500	-2.36405400	-0.61135100
H	-0.28675300	-2.75529600	1.52888000
O	-1.02545800	-2.23512600	-1.53949400
H	-0.33288500	-1.92279100	-2.12262000
Na	-2.31172300	-1.25041100	0.00601600

Zero-point correction= 0.057764 (Hartree/Particle)
Thermal correction to Energy= 0.069107
Thermal correction to Enthalpy= 0.070052
Thermal correction to Gibbs Free Energy= 0.018463
Sum of electronic and zero-point Energies= -1733.853458
Sum of electronic and thermal Energies= -1733.842115
Sum of electronic and thermal Enthalpies= -1733.841171
Sum of electronic and thermal Free Energies= -1733.892759

Transition State

C	-0.96605300	0.21447600	-0.60046900
H	-0.84584900	-0.36760200	-1.48874900
Cl	0.36893500	0.62551900	0.33499100
Cl	-0.87855700	1.88212000	-2.37241900
Cl	-2.51159400	0.66851800	-0.02820600
O	-1.18206400	-1.83906100	-0.06425600
H	-1.70847200	-1.94357800	-1.02582400
H	-1.81946800	-1.95305100	0.64544600
O	-2.20664500	-1.76472100	-2.26941400
H	-2.14050800	-2.60733300	-2.71884400
Na	-2.52969700	0.10827500	-3.23806800

Zero-point correction= 0.056179 (Hartree/Particle)
Thermal correction to Energy= 0.066756
Thermal correction to Enthalpy= 0.067700
Thermal correction to Gibbs Free Energy= 0.018657
Sum of electronic and zero-point Energies= -1733.797847
Sum of electronic and thermal Energies= -1733.787270
Sum of electronic and thermal Enthalpies= -1733.786326
Sum of electronic and thermal Free Energies= -1733.835369

Product (1)

C	0.41908300	-0.39981300	0.94437200
H	0.72889300	-0.15585300	-0.07056300
Cl	1.52066000	0.33872500	2.11752800
Cl	0.42757400	0.29110100	-2.41315900
Cl	-1.22743500	0.38381200	1.15007200
O	0.32542400	-1.74603500	1.10422000
H	-0.86452200	-2.48962500	-0.59438700
H	0.18094700	-1.95699800	2.03532300
O	-1.20978100	-2.15760800	-1.42928500
H	-0.42763400	-1.80940100	-1.89703500
Na	-1.89222100	0.01161700	-1.57125600

Zero-point correction= 0.061457 (Hartree/Particle)
Thermal correction to Energy= 0.072702
Thermal correction to Enthalpy= 0.073646
Thermal correction to Gibbs Free Energy= 0.022323
Sum of electronic and zero-point Energies= -1733.908275
Sum of electronic and thermal Energies= -1733.897030
Sum of electronic and thermal Enthalpies= -1733.896086
Sum of electronic and thermal Free Energies= -1733.947409

IRC

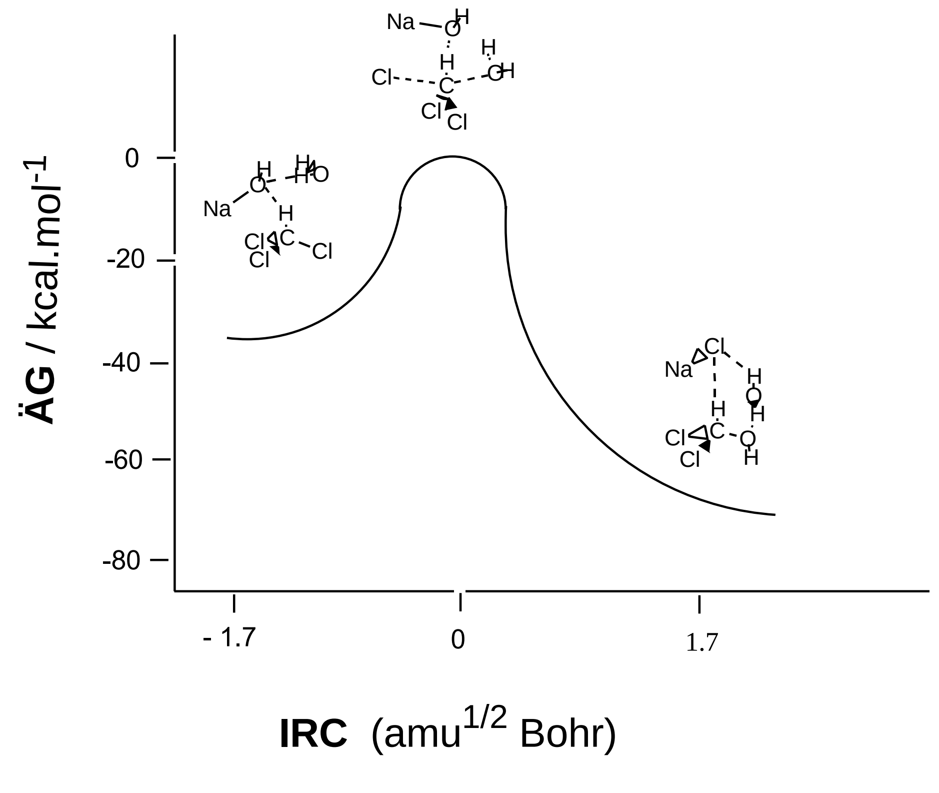

Figure-3

Intermediate (I-5)

C	-0.40094100	0.71543100	-0.16540400
H	0.27053700	0.76747200	-1.07668100
Cl	-0.02265700	-0.88008600	0.60091500
Cl	-0.02671100	2.01027600	0.97542900
Cl	-2.10529100	0.74919700	-0.62556900
O	1.37899600	0.35703900	-2.17946700
H	1.52352300	0.99117500	-2.87757900
Na	1.85143600	-1.42486000	-1.36455700
O	-2.37897400	0.23424000	2.77432000
H	-2.78103300	0.24143100	1.90204300
H	-1.70363200	0.91534500	2.72203400

Zero-point correction= 0.055325 (Hartree/Particle)
Thermal correction to Energy= 0.068386
Thermal correction to Enthalpy= 0.069330
Thermal correction to Gibbs Free Energy= 0.013242
Sum of electronic and zero-point Energies= -1733.823415
Sum of electronic and thermal Energies= -1733.810353
Sum of electronic and thermal Enthalpies= -1733.809409
Sum of electronic and thermal Free Energies= -1733.865497

Transition State

C	-0.67557600	-0.09717800	1.76759700
H	-0.08114500	0.78579000	1.82747100
Cl	0.08148900	-1.65755700	2.07265000
Cl	-1.02271300	0.33074100	3.96388800
Cl	-2.32661600	-0.08494500	1.29813400
O	0.01010500	-0.08962500	-0.10361500
H	-0.43164000	0.65694800	-0.51096400
Na	0.77702700	-1.94656700	-0.52006600
O	-4.29444500	0.77224900	3.84653000
H	-4.40829100	-0.10024500	4.22815800
H	-3.33704500	0.89752700	3.87728400

Zero-point correction= 0.056462 (Hartree/Particle)
Thermal correction to Energy= 0.068664
Thermal correction to Enthalpy= 0.069608
Thermal correction to Gibbs Free Energy= 0.014128
Sum of electronic and zero-point Energies= -1733.772012
Sum of electronic and thermal Energies= -1733.759809
Sum of electronic and thermal Enthalpies= -1733.758865
Sum of electronic and thermal Free Energies= -1733.814346

Product (2)

C	-0.71258000	-1.45278200	-2.24024800
H	-1.10041000	-1.77648900	-1.27015700
Cl	-0.78943800	-0.46818300	0.94723400
Cl	0.66084500	3.28705500	0.55747300
Cl	-1.03127900	-2.59263600	-3.51819000
O	-0.13003400	-0.43437300	-2.43412800
H	-0.15931500	2.49788200	1.83357600
Na	0.29956200	1.13420100	-0.76803700
O	-0.66149000	1.86136300	2.56422200
H	-0.78708000	0.95474800	2.04953300
H	-0.05482300	1.70525200	3.29888100

Zero-point correction= 0.056307 (Hartree/Particle)
Thermal correction to Energy= 0.067988
Thermal correction to Enthalpy= 0.068932
Thermal correction to Gibbs Free Energy= 0.014462
Sum of electronic and zero-point Energies= -1733.933369
Sum of electronic and thermal Energies= -1733.921688
Sum of electronic and thermal Enthalpies= -1733.920744
Sum of electronic and thermal Free Energies= -1733.975214

IRC

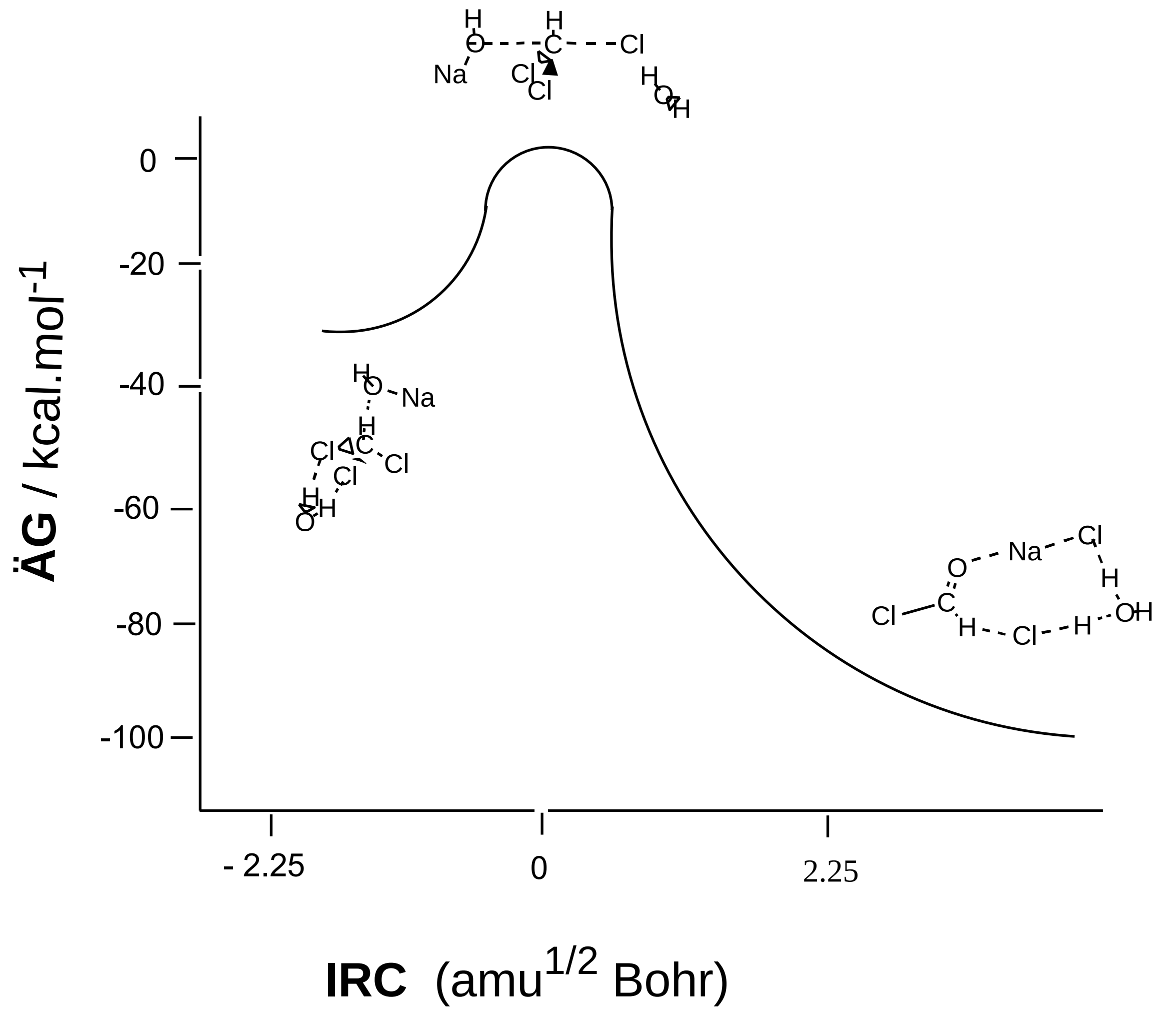

ÄG / kcal.mol-1
0
-20
-40
-60
-80
-100
- 2.25
0
2.25
IRC (amu1/2 Bohr)

Figure-4

Intermediate I-7

C	-0.45139000	0.16441700	0.67138900
H	-0.89349100	-0.38044800	-0.18052600
Cl	1.22507400	-0.47373700	0.83981100
Cl	-0.38913200	1.88717500	0.28843300
Cl	-1.34513800	-0.12467400	2.15710600
O	-0.92135800	-1.22284700	-1.75918600
H	-1.76950100	-1.54004900	-2.06235100
Na	1.16642600	-1.51527500	-1.80073000
O	0.64787200	0.42316800	-2.79377900
H	0.59925500	1.32960800	-2.48841000
H	-0.20649400	-0.05142700	-2.49039700

Zero-point correction= 0.057728 (Hartree/Particle)
Thermal correction to Energy= 0.069104
Thermal correction to Enthalpy= 0.070049
Thermal correction to Gibbs Free Energy= 0.018229
Sum of electronic and zero-point Energies= -1733.853476
Sum of electronic and thermal Energies= -1733.842100
Sum of electronic and thermal Enthalpies= -1733.841155
Sum of electronic and thermal Free Energies= -1733.892975

Transition State 1

C	-0.24278800	0.13142700	0.52805300
H	-0.69778700	-0.65109300	-0.60858800
Cl	1.49526200	-0.42386700	0.89430800
Cl	-0.13923400	1.89951300	0.20317800
Cl	-1.17787600	-0.11018000	2.01783200
O	-0.82154400	-1.28041000	-1.57208500
H	-1.66618100	-1.73036300	-1.55198200
Na	1.34657000	-1.41386400	-1.60011500
O	0.89287800	0.54109300	-2.65146700
H	0.93970900	1.34244300	-2.11899300
H	-0.03028800	0.24757600	-2.56684000

Zero-point correction= 0.054256 (Hartree/Particle)
Thermal correction to Energy= 0.065212
Thermal correction to Enthalpy= 0.066156
Thermal correction to Gibbs Free Energy= 0.016219
Sum of electronic and zero-point Energies= -1733.845642
Sum of electronic and thermal Energies= -1733.834687
Sum of electronic and thermal Enthalpies= -1733.833742
Sum of electronic and thermal Free Energies= -1733.883680

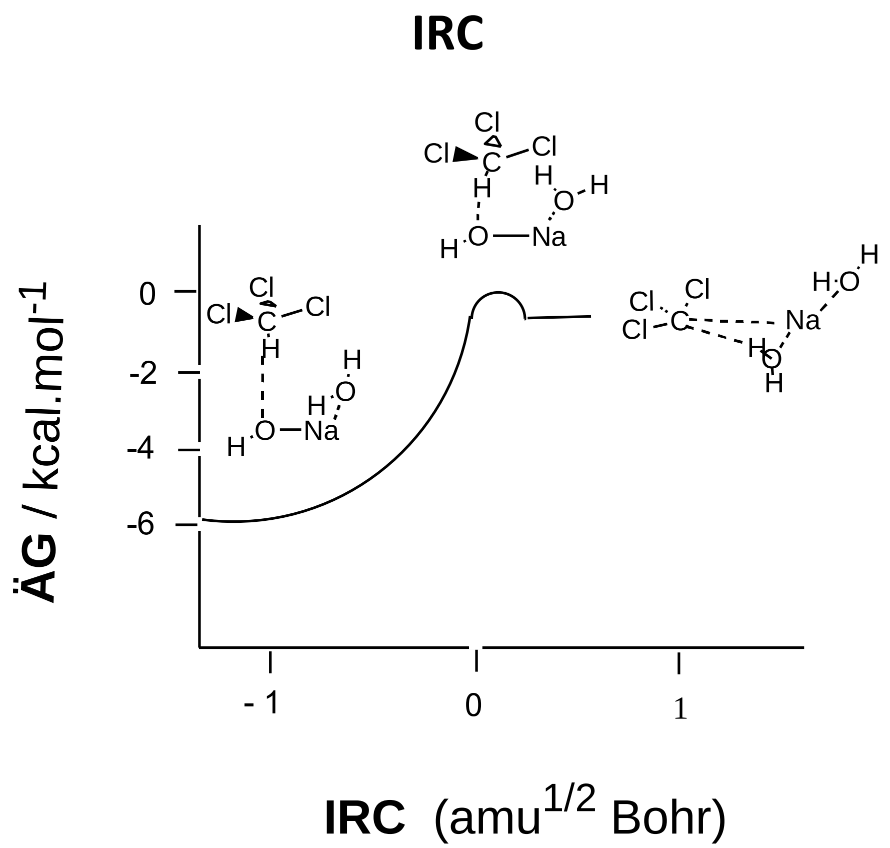

Intermediate I-8

C	-0.51169500	-0.12719800	0.78960500
H	-1.01763000	-0.78322700	-0.65582800
Cl	0.92327200	-1.19404800	1.33161700
Cl	0.28339100	1.49735900	0.36954600
Cl	-1.51671800	0.14103400	2.22175700
O	-0.95760700	-1.13394000	-1.64326500
H	-1.51600300	-1.90981100	-1.71332100
Na	1.21505300	-0.51707400	-1.29104800
O	0.51419600	0.54774400	-3.17374500
H	0.36052300	1.49167600	-3.25021100
H	-0.34317600	0.14529900	-2.95408200

Zero-point correction= 0.056438 (Hartree/Particle)
Thermal correction to Energy= 0.068357
Thermal correction to Enthalpy= 0.069301
Thermal correction to Gibbs Free Energy= 0.016976
Sum of electronic and zero-point Energies= -1733.844295
Sum of electronic and thermal Energies= -1733.832376
Sum of electronic and thermal Enthalpies= -1733.831432
Sum of electronic and thermal Free Energies= -1733.883757

Transition State 2

C	0.74613900	-0.33247600	1.05366300
H	-0.22298600	-0.96866200	-1.35038000
Cl	2.23508000	-1.10826400	0.69930700
Cl	-0.29539500	0.74933100	-2.75834600
Cl	-0.01589400	-1.10348200	2.39807700
O	-0.42998200	-1.37329000	-0.48051400
H	-1.34357100	-1.10065100	-0.31901300
Na	-0.18372200	1.47717400	-0.33704600
O	-2.39304100	0.80841800	-0.60822100
H	-3.32121800	1.04852900	-0.58252100
H	-2.11877900	0.77476400	-1.55007700

Zero-point correction= 0.057029 (Hartree/Particle)
Thermal correction to Energy= 0.068669
Thermal correction to Enthalpy= 0.069614
Thermal correction to Gibbs Free Energy= 0.017378
Sum of electronic and zero-point Energies= -1733.822511
Sum of electronic and thermal Energies= -1733.810871
Sum of electronic and thermal Enthalpies= -1733.809927
Sum of electronic and thermal Free Energies= -1733.862162

IRC

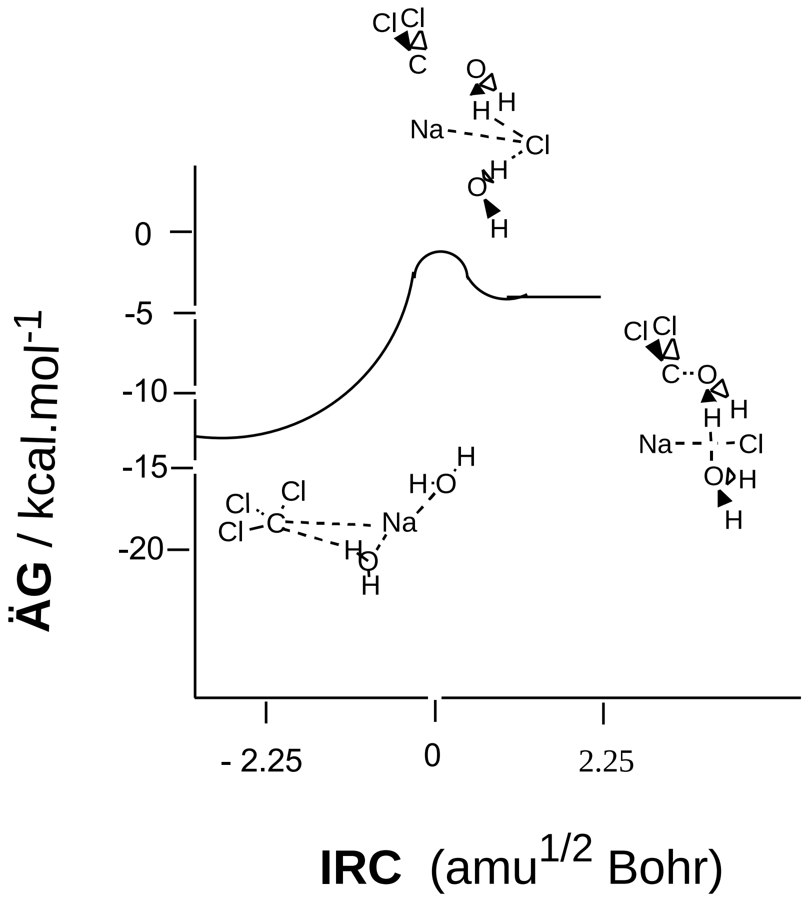

Intermediate I-9

C	0.38244400	-0.21824600	0.70410300
H	-0.23260500	-0.58231500	-1.26803800
Cl	2.01187400	-0.93459400	0.62323400
Cl	-0.29885800	0.72342700	-2.60565400
Cl	-0.41500600	-0.80899100	2.21143000
O	-0.38184900	-1.08694100	-0.37074800
H	-1.32278700	-0.91554900	-0.17501800
Na	-0.21908200	1.90941200	-0.28498500
O	-2.41248000	0.92030200	-0.48748500
H	-3.35548700	1.05406800	-0.36799100
H	-2.23143900	0.87222400	-1.44418500

Zero-point correction= 0.058337 (Hartree/Particle)
Thermal correction to Energy= 0.069627
Thermal correction to Enthalpy= 0.070571
Thermal correction to Gibbs Free Energy= 0.019615
Sum of electronic and zero-point Energies= -1733.829843
Sum of electronic and thermal Energies= -1733.818554
Sum of electronic and thermal Enthalpies= -1733.817609
Sum of electronic and thermal Free Energies= -1733.868566

Transition State 3

C	-0.23549900	-0.54096000	0.30549500
H	-0.66557200	-0.35298500	-0.93597800
Cl	0.82577100	-1.83916200	0.82125200
Cl	-1.46694700	1.80540900	-1.98309200
Cl	-0.92081100	0.29986000	1.74520500
O	-1.36237200	-1.18190500	-0.45236900
H	-2.25431900	-0.67771600	-0.32014400
Na	0.24687300	1.89837100	-0.20099200
O	-3.47273800	0.21716600	-0.36435300
H	-4.21403700	-0.12219900	-0.87290500
H	-3.06001500	0.92474400	-0.91047700

Zero-point correction= 0.054078 (Hartree/Particle)
Thermal correction to Energy= 0.064877
Thermal correction to Enthalpy= 0.065821
Thermal correction to Gibbs Free Energy= 0.015905
Sum of electronic and zero-point Energies= -1733.790410
Sum of electronic and thermal Energies= -1733.779611
Sum of electronic and thermal Enthalpies= -1733.778667
Sum of electronic and thermal Free Energies= -1733.828583

IRC

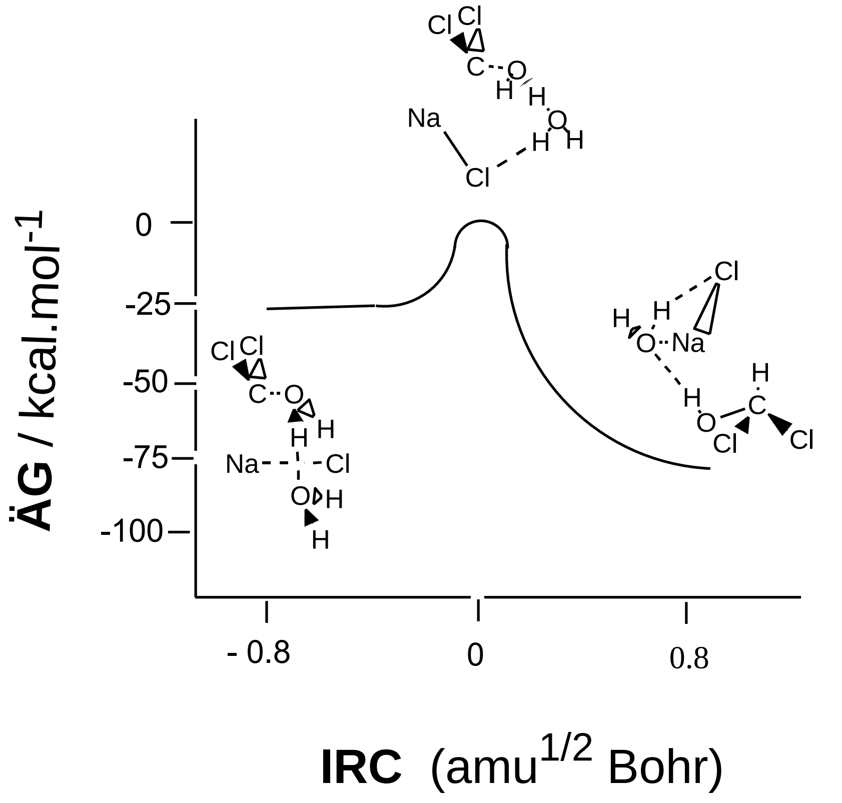

Product (1)

C	0.45872900	-1.09567200	0.15065600
H	0.61775400	-0.55553700	-0.77949600
Cl	1.71694100	-2.29462900	0.38474800
Cl	-0.31095500	1.41730100	-2.28117100
Cl	0.72847300	0.25948300	1.44598100
O	-0.74554800	-1.64951900	0.31173900
H	-1.42953500	-1.03095700	-0.02258800
Na	-0.78220700	2.03439200	0.09596800
O	-2.40031300	0.38213300	-0.53231300
H	-3.33365900	0.25200100	-0.71701300
H	-1.93955700	0.59918900	-1.38796100

Zero-point correction= 0.061115 (Hartree/Particle)
Thermal correction to Energy= 0.072011
Thermal correction to Enthalpy= 0.072955
Thermal correction to Gibbs Free Energy= 0.022358
Sum of electronic and zero-point Energies= -1733.912388
Sum of electronic and thermal Energies= -1733.901492
Sum of electronic and thermal Enthalpies= -1733.900548
Sum of electronic and thermal Free Energies= -1733.951145

Figure-5

Intermediate (I-12)

C	-0.07922200	-0.91617900	-0.76656000
Cl	0.93420800	-2.24964700	-1.12359600
Cl	-1.12196400	1.31797600	1.58783900
Cl	-1.75683900	-1.29885100	-0.79442500
O	0.03523400	-1.85514300	2.12465100
H	-0.81604100	-2.28437600	2.01402200
H	-0.17671700	-0.91843900	2.18767900
C	0.38046700	0.30822200	-0.47210200
C	1.80409700	0.59342400	-0.38621700
C	2.24424000	1.85780500	-0.36687200
C	1.31756300	2.97608400	-0.44812200
C	0.00036400	2.78567300	-0.37580900
C	-0.57416700	1.42748400	-0.18415000
H	2.49100000	-0.23816300	-0.34626800
H	3.30401400	2.06123300	-0.30612000
H	1.71554100	3.97570800	-0.55651500
H	-0.69712500	3.61104500	-0.40083400
H	-1.50573600	1.30348400	-0.72443400

Zero-point correction= 0.124734 (Hartree/Particle)

Thermal correction to Energy= 0.137870

Thermal correction to Enthalpy= 0.138814

Thermal correction to Gibbs Free Energy= 0.083396

Sum of electronic and zero-point Energies= -1726.615453

Sum of electronic and thermal Energies= -1726.602316

Sum of electronic and thermal Enthalpies= -1726.601372

Sum of electronic and thermal Free Energies= -1726.656791

Transition State

C	-1.53206900	-0.83301100	-0.80304700
Cl	-0.53663600	-2.15298400	-1.20502000
Cl	-2.90362700	1.21572700	1.87253000
Cl	-3.19085500	-1.13937300	-0.94773800
O	-1.34389500	-1.17943500	1.36684600
H	-1.90015300	-1.89535100	1.69199800
H	-1.81895100	-0.33797700	1.67059400
C	-1.03027200	0.48213600	-0.76757800
C	0.36603300	0.70682800	-0.65654400
C	0.84246400	1.98259800	-0.56371500
C	-0.05178800	3.06808700	-0.57898600
C	-1.41042800	2.86891300	-0.66021800
C	-1.92019600	1.57620700	-0.72263600
H	1.04055200	-0.13506100	-0.62525200
H	1.90415300	2.15941700	-0.47245300
H	0.33826300	4.07496900	-0.51337800
H	-2.09362100	3.70428300	-0.63706100
H	-2.98012500	1.41887100	-0.81741200

Zero-point correction= 0.124070 (Hartree/Particle)
Thermal correction to Energy= 0.135543
Thermal correction to Enthalpy= 0.136488
Thermal correction to Gibbs Free Energy= 0.085250
Sum of electronic and zero-point Energies= -1726.587337
Sum of electronic and thermal Energies= -1726.575863
Sum of electronic and thermal Enthalpies= -1726.574919
Sum of electronic and thermal Free Energies= -1726.626157

Product (3)

C	-0.35283300	-0.91199900	-0.22445700
Cl	-0.20142100	-1.82274200	-1.77906000
Cl	-0.02003700	-0.08331800	3.75403800
Cl	-2.12169500	-0.79431100	0.14269100
O	0.26714800	-1.61291400	0.77510100
H	-0.08524200	-2.51113800	0.79740900
H	-0.01510300	-0.62638500	2.58823200
C	0.25233900	0.45992700	-0.36233000
C	1.38441400	0.79747200	0.36487800
C	1.93930500	2.06334600	0.22279000
C	1.37225500	2.98082000	-0.64477000
C	0.24205000	2.63517500	-1.37762300
C	-0.31796900	1.37961900	-1.23675300
H	1.83437600	0.08147500	1.03591300
H	2.81721300	2.32621500	0.79605600
H	1.80666300	3.96504800	-0.75278000
H	-0.20382500	3.34785600	-2.05719800
H	-1.19934600	1.10655100	-1.80167000

Zero-point correction= 0.124150 (Hartree/Particle)
Thermal correction to Energy= 0.136180
Thermal correction to Enthalpy= 0.137124
Thermal correction to Gibbs Free Energy= 0.082827
Sum of electronic and zero-point Energies= -1726.665671
Sum of electronic and thermal Energies= -1726.653641
Sum of electronic and thermal Enthalpies= -1726.652697
Sum of electronic and thermal Free Energies= -1726.706994

IRC

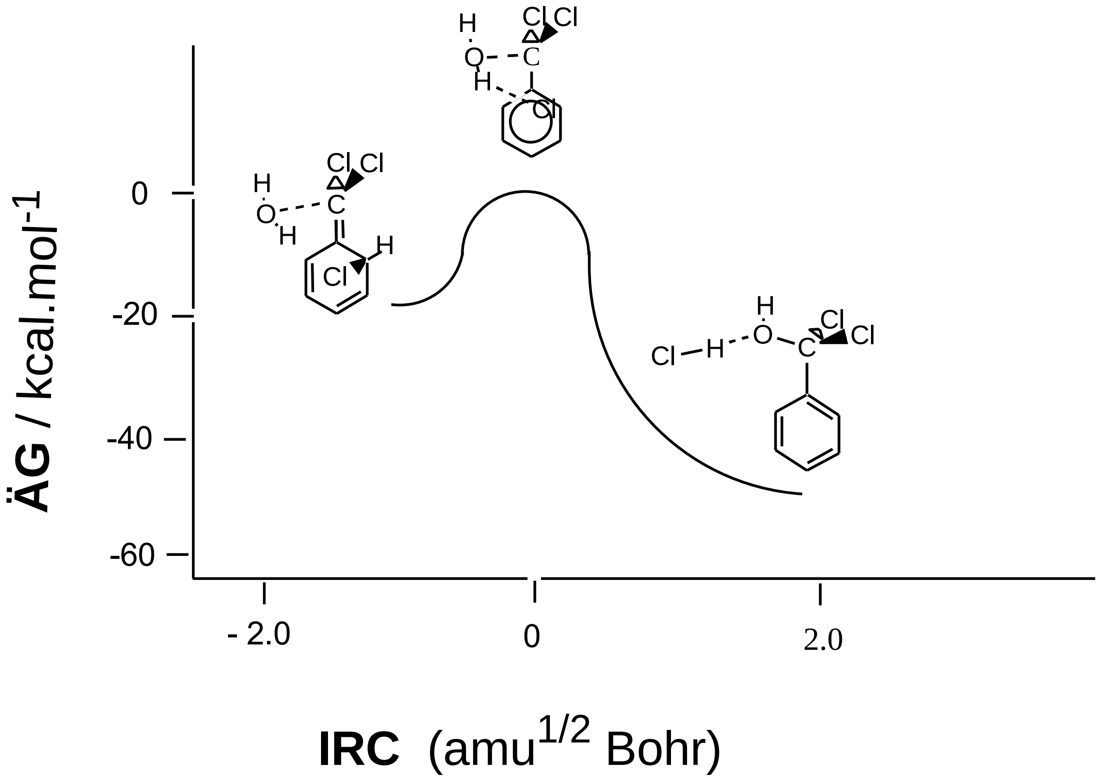

Figure-6

Intermediate (I-13)

C	0.50804000	-0.31792400	-0.74261100
Cl	0.61808400	-1.60504600	0.52769700
Cl	1.29689600	-0.96171200	-2.19691200
Cl	-1.22938800	-0.09924600	-1.12569700
O	-1.93722500	0.46288100	2.31172200
H	-2.19963600	1.06596800	3.00328800
C	1.10908300	0.96462100	-0.22071200
C	2.24795800	1.52245700	-0.78732200
C	2.77245600	2.69442600	-0.25976800
C	2.16412900	3.30035100	0.82726000
C	1.02722600	2.73224500	1.38854400
C	0.48726900	1.56473700	0.87567500
H	2.72937400	1.05708900	-1.63333000
H	3.65732300	3.12845300	-0.70410900
H	2.57486400	4.21353800	1.23672000
H	0.54697700	3.19740400	2.23863300
H	-0.41196600	1.13925000	1.34603000
Na	-2.23102900	-1.33308300	1.45339900
O	-2.18705500	-3.23703100	0.18353600

H	-2.79778400	-3.80466700	-0.29131700
H	-1.37667000	-3.21047800	-0.33640700

Zero-point correction= 0.136879 (Hartree/Particle)
Thermal correction to Energy= 0.154565
Thermal correction to Enthalpy= 0.155509
Thermal correction to Gibbs Free Energy= 0.088765
Sum of electronic and zero-point Energies= -1964.784169
Sum of electronic and thermal Energies= -1964.766483
Sum of electronic and thermal Enthalpies= -1964.765539
Sum of electronic and thermal Free Energies= -1964.832284

Transition State

C	-0.19668800	0.13187800	0.06601300
Cl	-1.12392500	0.73844900	-1.31274900
Cl	0.16887800	2.25683700	0.84339600
Cl	-1.14252600	-0.28442300	1.49215500
O	-0.50944500	-1.76643300	-0.56916000
H	0.20545700	-1.94913100	-1.18182700
C	1.26665700	-0.11519800	-0.02444900
C	1.98348700	0.34801800	-1.12132900
C	3.34173600	0.09053000	-1.21499300
C	3.99278700	-0.61998400	-0.21740400
C	3.27200000	-1.07644400	0.87726500
C	1.91245700	-0.83351900	0.97470300
H	1.48763400	0.93416500	-1.88114200
H	3.89403100	0.46056100	-2.06784800
H	5.05415700	-0.81258400	-0.28971800
H	3.76926300	-1.63088500	1.66132200
H	1.35106500	-1.20326100	1.81939500
Na	-2.60397600	-1.66268100	-0.51021600
O	-4.08992800	0.02976500	-0.21412800
H	-4.89488400	0.24487100	0.26201300
H	-3.50877600	0.79563000	-0.12199000

Zero-point correction= 0.137790 (Hartree/Particle)
Thermal correction to Energy= 0.154181
Thermal correction to Enthalpy= 0.155125
Thermal correction to Gibbs Free Energy= 0.092605
Sum of electronic and zero-point Energies= -1964.738960
Sum of electronic and thermal Energies= -1964.722569
Sum of electronic and thermal Enthalpies= -1964.721625
Sum of electronic and thermal Free Energies= -1964.784145

Product (3)

C	-0.76529200	0.59871300	1.65369500
Cl	-0.07147300	-1.05250800	2.04643800

Cl	1.10424500	-2.06344800	-1.72615600
Cl	-2.09150400	0.32430600	0.45475200
O	-1.38454800	1.09196600	2.76071100
H	-0.70648400	1.36601300	3.39222600
C	0.29795600	1.48748300	1.05925300
C	1.23554800	0.99099600	0.15986900
C	2.16576700	1.85168300	-0.39811300
C	2.16904500	3.19872800	-0.06029800
C	1.22647000	3.69219800	0.82872200
C	0.28284400	2.84055700	1.38434900
H	1.24562600	-0.05402000	-0.12790100
H	2.88599200	1.45820500	-1.10161300
H	2.90141700	3.86466100	-0.49569100
H	1.21609100	4.74210600	1.08624400
H	-0.47378300	3.23443600	2.04863600
Na	-1.00229700	-2.21531000	-0.39005300
O	-1.10325300	-4.13574900	-1.54464200
H	-1.43743900	-4.82335900	-2.12085400
H	-0.26123900	-3.80856000	-1.92347700

Zero-point correction= 0.140969 (Hartree/Particle)
Thermal correction to Energy= 0.157561
Thermal correction to Enthalpy= 0.158505
Thermal correction to Gibbs Free Energy= 0.091621
Sum of electronic and zero-point Energies= -1964.860887
Sum of electronic and thermal Energies= -1964.844296
Sum of electronic and thermal Enthalpies= -1964.843352
Sum of electronic and thermal Free Energies= -1964.910236

IRC

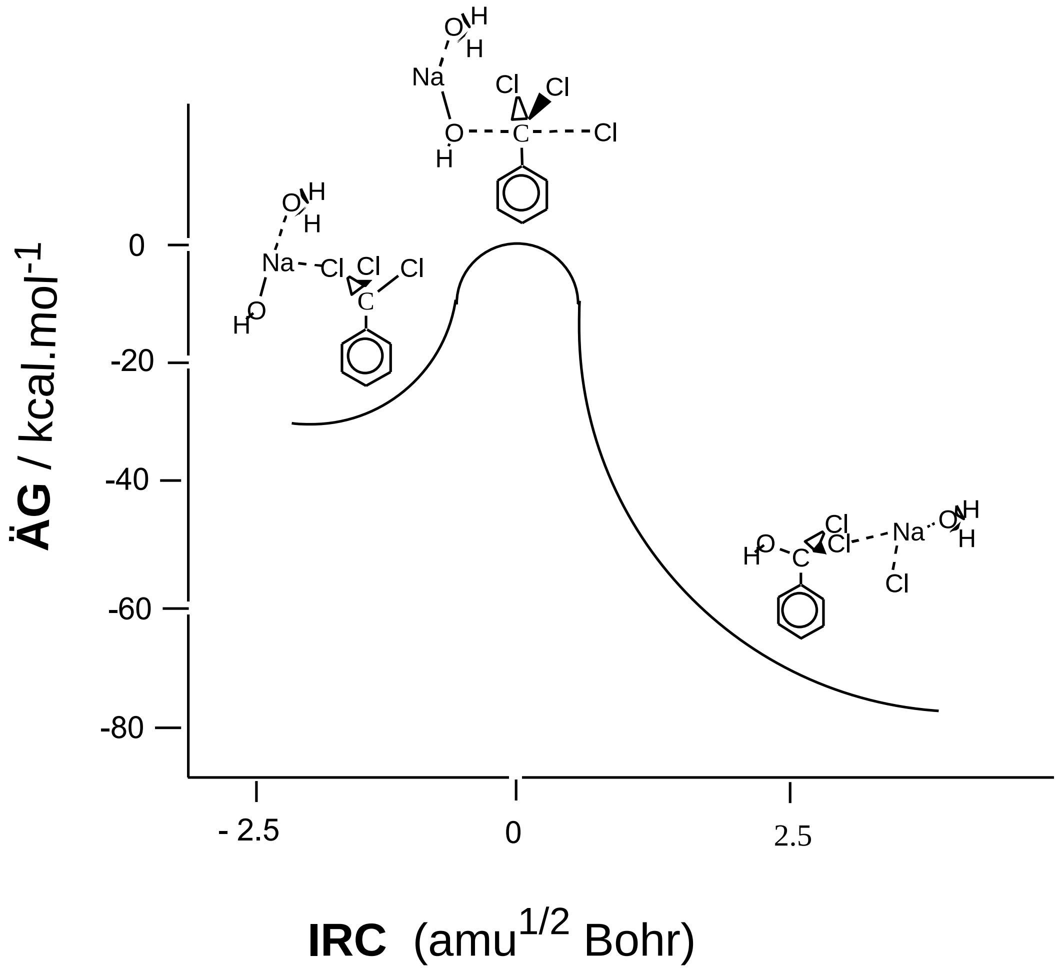

Figure-7

Intermediate (I-14)

C	-0.49311800	0.78120100	0.23226300
H	-0.72434500	1.83253900	0.12927300
Cl	0.76920400	0.66216700	1.53043000
Cl	0.28614800	0.27552200	-1.32629500
O	-0.37001700	-2.34135600	0.63333800
H	0.32327000	-2.13279200	1.26813600
H	0.06810800	-2.33651800	-0.22406400
O	-1.58872600	0.07475800	0.51316400
H	-1.34286100	-0.87406300	0.59971300

Zero-point correction= 0.059128 (Hartree/Particle)
Thermal correction to Energy= 0.066744
Thermal correction to Enthalpy= 0.067688
Thermal correction to Gibbs Free Energy= 0.026419
Sum of electronic and zero-point Energies= -1111.326367
Sum of electronic and thermal Energies= -1111.318751
Sum of electronic and thermal Enthalpies= -1111.317807
Sum of electronic and thermal Free Energies= -1111.359075

Transition State

C	0.68338200	-0.32141200	-0.70066700
H	0.73867600	0.67659400	-0.28168600
Cl	2.69575500	-0.88139000	0.35386800
Cl	1.21969000	-0.36104400	-2.37619200
O	1.16240400	-3.17067300	-0.16024000
H	0.94741600	-3.67705800	0.63269300
H	1.95284600	-2.52586400	0.07106200
O	-0.12514000	-1.16575200	-0.30696400
H	0.40551800	-2.36628900	-0.31385600

Zero-point correction= 0.054749 (Hartree/Particle)
Thermal correction to Energy= 0.060911
Thermal correction to Enthalpy= 0.061856
Thermal correction to Gibbs Free Energy= 0.023613
Sum of electronic and zero-point Energies= -1111.299747
Sum of electronic and thermal Energies= -1111.293585
Sum of electronic and thermal Enthalpies= -1111.292641
Sum of electronic and thermal Free Energies= -1111.330884

Product (2)

C	-0.99145000	0.97746600	-0.10146800
H	-0.65086400	1.95394400	0.24863100

Cl	1.68338700	-0.04596900	1.36515800
Cl	-0.32263000	0.59836100	-1.67857500
O	-0.26605400	-2.22352300	0.51206700
H	-0.49536800	-2.82367100	1.22617300
H	0.91601000	-1.05050900	0.99390500
O	-1.72232700	0.24830700	0.47502300
H	-1.05305700	-1.68595400	0.35462400

Zero-point correction= 0.052694 (Hartree/Particle)
Thermal correction to Energy= 0.061957
Thermal correction to Enthalpy= 0.062901
Thermal correction to Gibbs Free Energy= 0.015386
Sum of electronic and zero-point Energies= -1111.326329
Sum of electronic and thermal Energies= -1111.317067
Sum of electronic and thermal Enthalpies= -1111.316123
Sum of electronic and thermal Free Energies= -1111.363637

IRC

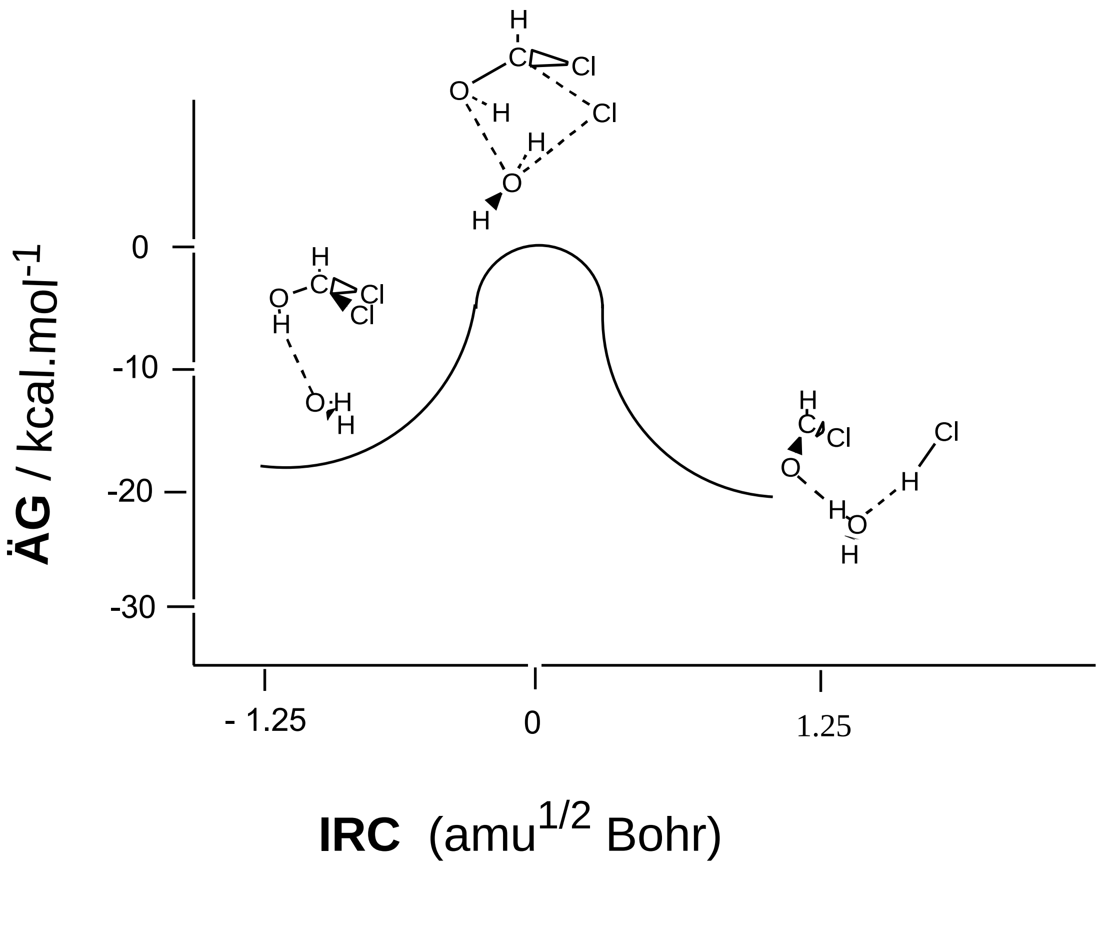

Figure-8

Intermediate (I-15)

C	-0.21974800	0.99286600	-0.86177300
H	-0.17747000	1.96328200	-1.33769500
Cl	-0.57496600	1.33261700	0.90231300
Cl	1.46961700	0.32268000	-0.94183700
O	0.81093500	-1.59244800	1.72035800
H	0.66515800	-0.64060300	1.78589300
H	1.64840300	-1.67438700	1.25523200
O	-1.12011800	0.20716400	-1.42454000
H	-1.16316100	-0.68014200	-0.96166500
O	-1.16739900	-2.05033200	-0.11659000
H	-1.98949700	-2.24664400	0.33792500
H	-0.47370300	-2.00727900	0.57295300

Zero-point correction= 0.084133 (Hartree/Particle)
Thermal correction to Energy= 0.094408
Thermal correction to Enthalpy= 0.095352
Thermal correction to Gibbs Free Energy= 0.047823
Sum of electronic and zero-point Energies= -1187.745114
Sum of electronic and thermal Energies= -1187.734839
Sum of electronic and thermal Enthalpies= -1187.733894
Sum of electronic and thermal Free Energies= -1187.781424

Transition State

C	-0.40942100	0.52137400	0.26290300
H	-0.61885700	1.57980300	0.15748700
Cl	-1.15021500	0.62450200	2.45505500
Cl	1.35639200	0.28449600	0.54753300
O	0.50007900	-1.94958000	2.79937600
H	0.27140900	-1.02068500	3.01152500
H	1.34751100	-1.90391900	2.34394700
O	-1.03571100	-0.36920200	-0.28461900
H	-1.27063200	-1.52191100	0.53022000
O	-1.51120700	-2.14915600	1.34083200
H	-2.05949400	-1.54258200	1.87973600
H	-0.62849100	-2.20302300	1.91009700

Zero-point correction= 0.082161 (Hartree/Particle)
Thermal correction to Energy= 0.090982
Thermal correction to Enthalpy= 0.091926
Thermal correction to Gibbs Free Energy= 0.047643
Sum of electronic and zero-point Energies= -1187.726784
Sum of electronic and thermal Energies= -1187.717963
Sum of electronic and thermal Enthalpies= -1187.717019
Sum of electronic and thermal Free Energies= -1187.761302

Product (2)

C	0.25666900	0.75508900	-1.22318800
H	-0.42569200	1.60393100	-1.28249600
Cl	-1.42876400	0.75432100	1.40145900
Cl	1.80025600	1.24300800	-0.51009000
O	0.94570900	-1.63654400	1.14503600
H	0.74291100	-0.80819400	1.59742600
H	1.55749200	-1.39185600	0.44418500
O	0.05125100	-0.35075300	-1.57833700
H	-1.62316800	-1.75607700	-0.68705500
O	-1.62091700	-1.85763600	0.27089300
H	-1.65028900	-0.49879600	0.85212000
H	-0.68680000	-2.04282500	0.50634800

Zero-point correction= 0.078664 (Hartree/Particle)
Thermal correction to Energy= 0.089891
Thermal correction to Enthalpy= 0.090835
Thermal correction to Gibbs Free Energy= 0.040627
Sum of electronic and zero-point Energies= -1187.744929
Sum of electronic and thermal Energies= -1187.733702
Sum of electronic and thermal Enthalpies= -1187.732758
Sum of electronic and thermal Free Energies= -1187.782966

IRC

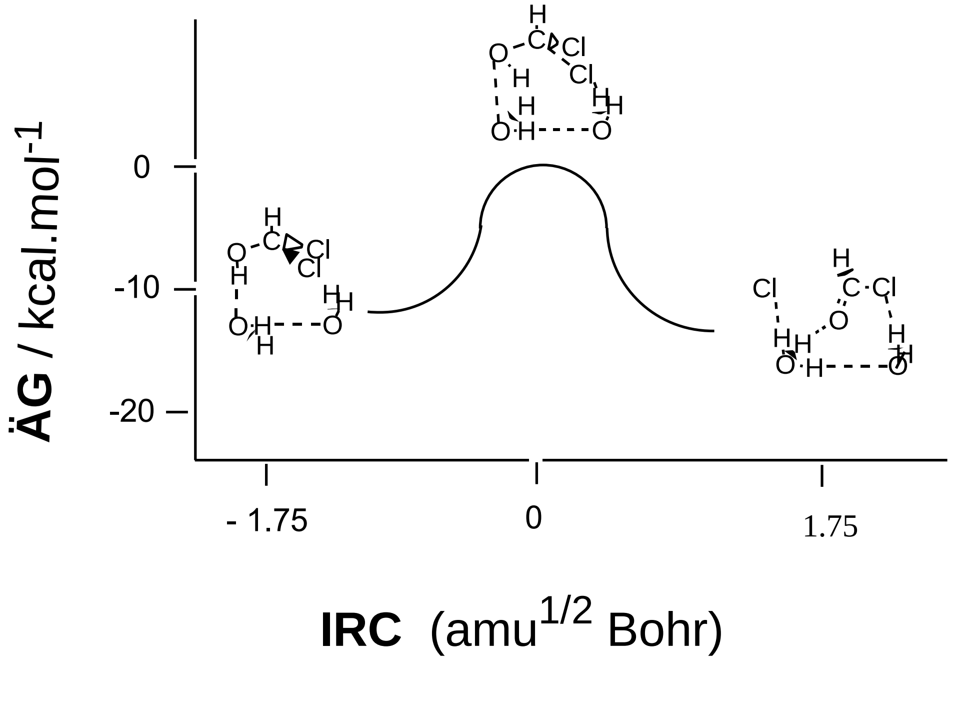

Figure-9

Intermediate (I-16)

C	0.72643400	-0.65939700	-0.52811400
H	0.31704600	0.08817300	-1.21252700
Cl	-0.04324700	-0.44851500	1.08234700
O	1.53070600	-1.48844400	-0.72822300
O	-1.16950800	1.65376100	-1.09410700
H	-1.36007300	2.56032500	-1.34192000
H	-1.39357100	1.58212400	-0.16236900

Zero-point correction= 0.042845 (Hartree/Particle)
Thermal correction to Energy= 0.050125
Thermal correction to Enthalpy= 0.051070
Thermal correction to Gibbs Free Energy= 0.010441
Sum of electronic and zero-point Energies= -650.517339
Sum of electronic and thermal Energies= -650.510059
Sum of electronic and thermal Enthalpies= -650.509114
Sum of electronic and thermal Free Energies= -650.549743

Transition State

C	0.76462200	-0.30632500	1.26821600
H	-0.08109100	0.67528300	0.90383800
Cl	-0.21847800	0.12745600	3.34923100
O	1.55068800	-1.11719800	1.26043800
O	-0.99975400	1.49275000	1.03237200
H	-0.70355400	2.40689300	0.95639200
H	-1.06512700	1.26534200	2.02030100

Zero-point correction= 0.039386 (Hartree/Particle)
Thermal correction to Energy= 0.044576
Thermal correction to Enthalpy= 0.045520
Thermal correction to Gibbs Free Energy= 0.010736
Sum of electronic and zero-point Energies= -650.484745
Sum of electronic and thermal Energies= -650.479556
Sum of electronic and thermal Enthalpies= -650.478612
Sum of electronic and thermal Free Energies= -650.513395

Product (4)

C	1.05916100	-0.78920400	-1.19980200
H	-0.48552300	0.92472000	-1.59281800
Cl	-0.68284000	0.19963800	1.61471000
O	1.71115300	-1.61554500	-0.81853000
O	-1.15905000	1.47040900	-1.17201900
H	-0.86756900	2.37926300	-1.27674200

H -1.01498500 0.80909600 0.51042600

Zero-point correction= 0.038583 (Hartree/Particle)
Thermal correction to Energy= 0.046738
Thermal correction to Enthalpy= 0.047682
Thermal correction to Gibbs Free Energy= 0.004876
Sum of electronic and zero-point Energies= -650.527313
Sum of electronic and thermal Energies= -650.519158
Sum of electronic and thermal Enthalpies= -650.518214
Sum of electronic and thermal Free Energies= -650.561020

IRC

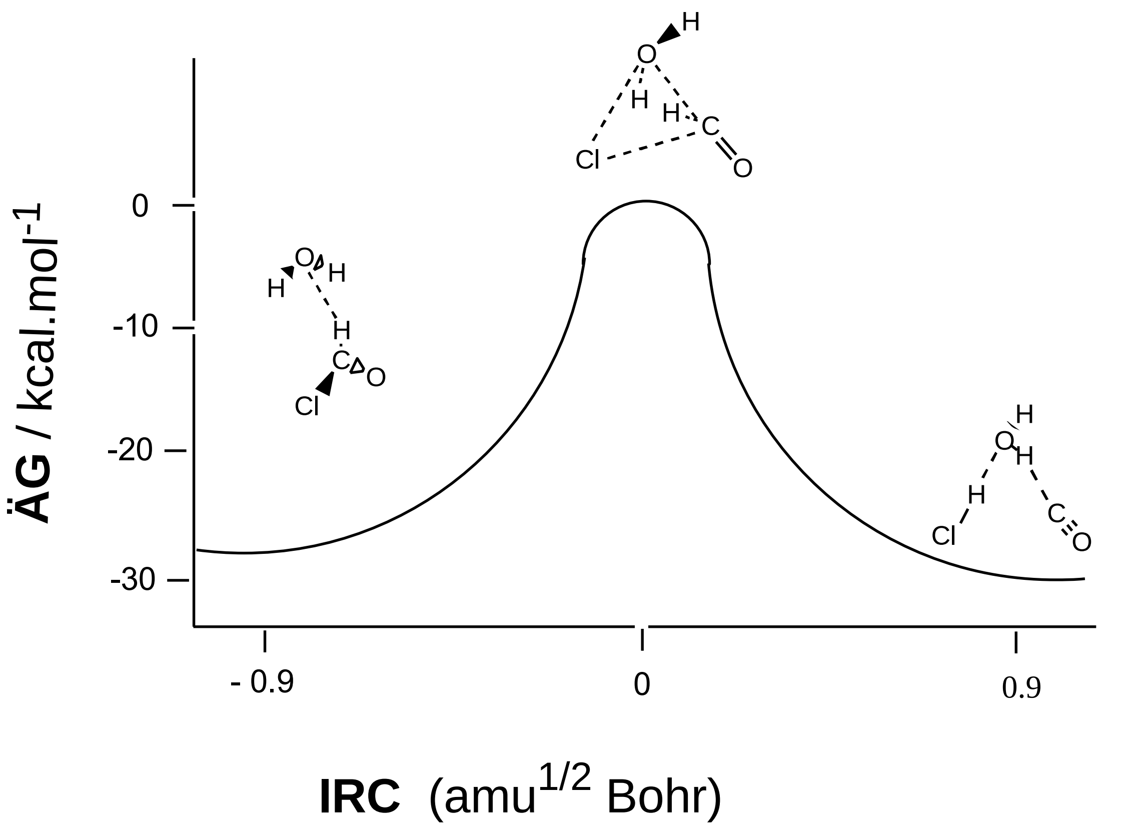

Figure-10

Intermediate (I-18)

C	-0.71733500	-0.91934500	-0.45312300
H	-1.68419000	-0.62146000	-0.85641300
Cl	-0.72251400	-0.84872000	1.31402900
O	0.22490400	-1.28042100	-1.06559300
O	-0.48943500	1.71637800	-0.72174900
H	0.47174800	1.58025900	-0.73960100
O	2.19153600	0.80898300	-0.66843200
H	2.73245000	0.91059400	-1.45459200
H	1.91011400	-0.11367200	-0.67037900
H	-0.65466000	2.21564200	0.08041900

Zero-point correction= 0.068796 (Hartree/Particle)
Thermal correction to Energy= 0.078454
Thermal correction to Enthalpy= 0.079398
Thermal correction to Gibbs Free Energy= 0.033410
Sum of electronic and zero-point Energies= -726.932725
Sum of electronic and thermal Energies= -726.923067
Sum of electronic and thermal Enthalpies= -726.922123
Sum of electronic and thermal Free Energies= -726.968111

Transition State

C	0.75335500	0.29596000	1.10550000
H	-0.23015200	0.14771400	0.66511500
Cl	0.38393600	0.18877100	2.98748100
O	1.74414000	-0.34124900	0.70616600
O	0.99292600	1.84479300	0.97883600
H	2.17773300	1.89830800	0.94620100
O	3.33015100	1.47795900	0.91113500
H	3.79364000	1.68893200	0.09518600
H	2.90950700	0.51296600	0.82771600
H	0.63190900	2.25553100	1.77555800

Zero-point correction= 0.068550 (Hartree/Particle)
Thermal correction to Energy= 0.074494
Thermal correction to Enthalpy= 0.075438
Thermal correction to Gibbs Free Energy= 0.038295
Sum of electronic and zero-point Energies= -726.902295
Sum of electronic and thermal Energies= -726.896351
Sum of electronic and thermal Enthalpies= -726.895407
Sum of electronic and thermal Free Energies= -726.932550

Product (5)

C	-0.70441500	-0.25222000	-0.46481200
H	-1.68970100	-0.64080500	-0.70402800
Cl	-0.57276700	-0.49036500	1.37219700
O	0.23142500	-0.93853800	-1.12966900
O	-0.58496600	1.11182700	-0.71360500
H	1.54249900	1.43609800	-0.57026000
O	2.28079800	0.81865500	-0.63985100
H	2.83090100	1.14308900	-1.35620600
H	1.10170900	-0.51396600	-0.97999800
H	-1.18409300	1.58555500	-0.12797400

Zero-point correction= 0.073458 (Hartree/Particle)
Thermal correction to Energy= 0.081063
Thermal correction to Enthalpy= 0.082008
Thermal correction to Gibbs Free Energy= 0.041293
Sum of electronic and zero-point Energies= -726.938307
Sum of electronic and thermal Energies= -726.930701
Sum of electronic and thermal Enthalpies= -726.929757
Sum of electronic and thermal Free Energies= -726.970472

IRC

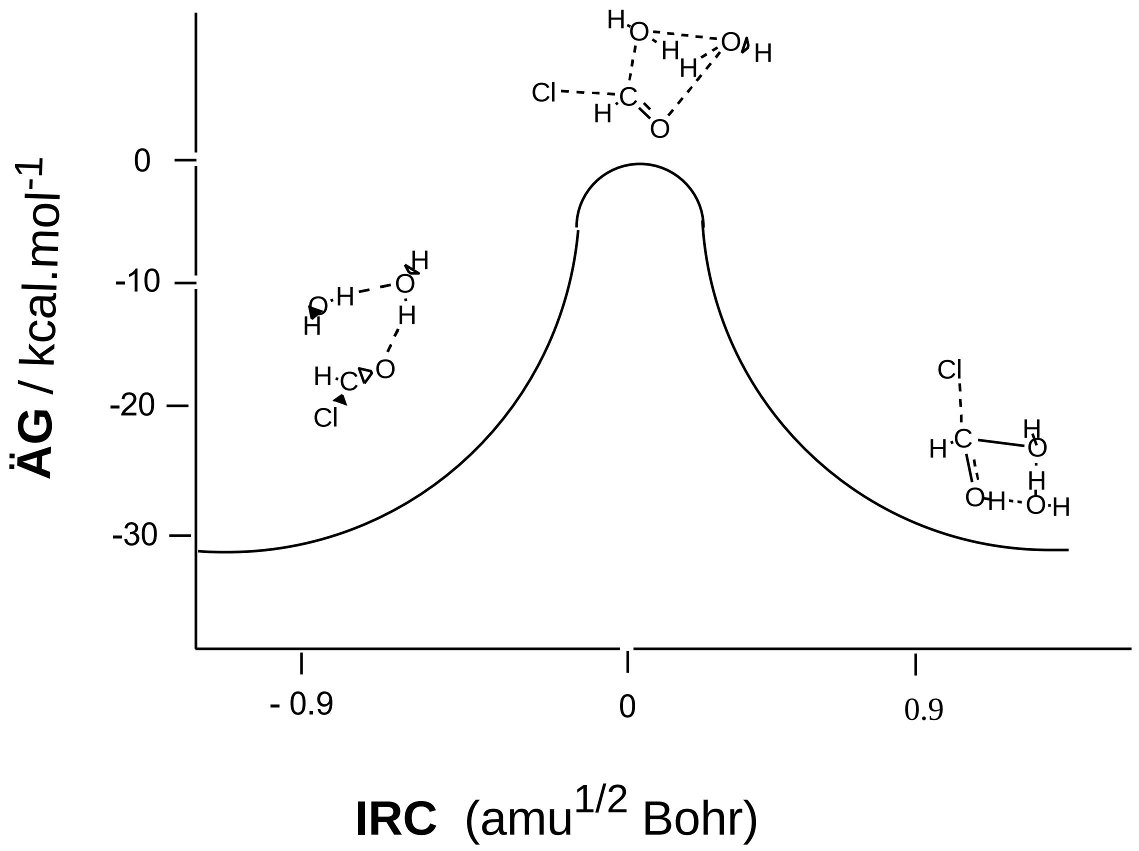
ÄG / kcal.mol-1
0
-10
-20
-30
- 0.9
0
0.9
IRC (amu1/2 Bohr)
Cl
H
C
O

Figure-11

Intermediate (i-19)

C	-0.93146200	-0.84744500	0.31382400
H	-1.38008300	-1.75370000	0.69887800
Cl	0.82890800	-0.92144500	1.09754100
O	-0.78324400	-0.94249200	-1.03218500
O	-0.52966500	2.39099200	-0.51902100
H	-1.06399400	2.76551000	-1.22235400
H	0.30726300	2.11836600	-0.92354800
O	-1.56368100	0.23188000	0.78778400
H	-0.20745100	-0.23684200	-1.36455700
H	-1.22002000	1.04920200	0.36455400
O	1.52030200	0.76966800	-1.43881900
H	1.74675400	0.26371500	-0.64203300
H	2.22970200	0.63468200	-2.07058700

Zero-point correction= 0.098688 (Hartree/Particle)
Thermal correction to Energy= 0.108982
Thermal correction to Enthalpy= 0.109926
Thermal correction to Gibbs Free Energy= 0.063277
Sum of electronic and zero-point Energies= -803.359927
Sum of electronic and thermal Energies= -803.349633
Sum of electronic and thermal Enthalpies= -803.348689
Sum of electronic and thermal Free Energies= -803.395338

Transition State

C	-0.87261500	-0.71864300	0.04733500
H	-1.21342900	-1.62018300	0.54209000
Cl	1.59986600	-0.86180800	1.18066600
O	-0.39704800	-0.84377700	-1.12710000
O	-0.14516200	2.43483800	-0.44989600
H	-0.57737000	2.94207900	-1.14063800
H	0.70044500	2.10767000	-0.80624900
O	-1.18229200	0.37431300	0.60547600
H	0.36812500	-0.17458900	-1.34902000
H	-0.80884000	1.21575800	0.15300200
O	1.63732200	0.58409500	-1.24438300
H	1.83457200	0.10938100	-0.33017300
H	2.34517000	0.34665800	-1.84973300

Zero-point correction= 0.095533 (Hartree/Particle)
Thermal correction to Energy= 0.104217
Thermal correction to Enthalpy= 0.105161
Thermal correction to Gibbs Free Energy= 0.061978
Sum of electronic and zero-point Energies= -803.351334
Sum of electronic and thermal Energies= -803.342650

Sum of electronic and thermal Enthalpies= -803.341706

Sum of electronic and thermal Free Energies= -803.384888

Product (6)

C	-2.18872000	-0.67302300	-0.34705000
H	-3.02181500	-1.37375900	-0.23117500
Cl	3.61179900	-0.27622400	0.96408800
O	-1.10690400	-1.03131000	-0.75676000
O	-0.51937500	2.21190300	-0.26703100
H	-0.61732600	3.02359400	-0.76788300
H	0.25978900	1.74386200	-0.61689700
O	-2.52337100	0.53822000	0.00287700
H	0.44370100	-0.29393800	-0.98716400
H	-1.75949100	1.17821500	-0.10671100
O	1.23819100	0.27490200	-1.08218200
H	2.59763600	-0.05728800	0.18645800
H	1.59813900	0.10059500	-1.95500500

Zero-point correction= 0.094020 (Hartree/Particle)

Thermal correction to Energy= 0.105215

Thermal correction to Enthalpy= 0.106159

Thermal correction to Gibbs Free Energy= 0.054187

Sum of electronic and zero-point Energies= -803.378555

Sum of electronic and thermal Energies= -803.367360

Sum of electronic and thermal Enthalpies= -803.366415

Sum of electronic and thermal Free Energies= -803.418387

IRC

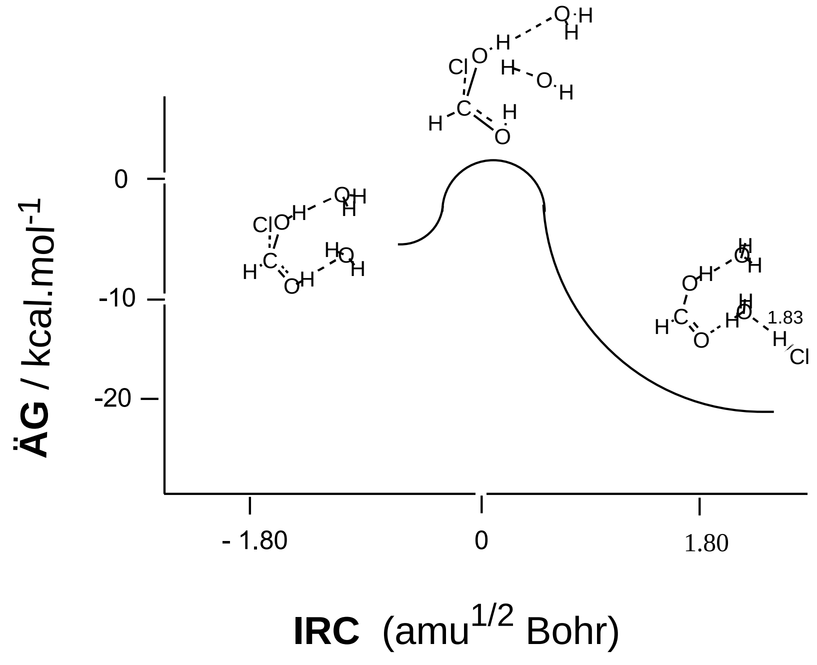

Figure-12

Intermediate (I-20)

C	-0.46536800	-0.18093700	0.16660200
O	0.20142500	0.09801500	1.11936500
Na	2.32769400	0.25923200	0.18304400
O	1.52541500	-0.19494200	-1.62051600
H	-0.02734400	-0.30019900	-0.86322200
H	1.67383900	-0.34366500	-2.55111000
Cl	-2.20818700	-0.42236500	0.34676900

Zero-point correction= 0.031986 (Hartree/Particle)
Thermal correction to Energy= 0.039317
Thermal correction to Enthalpy= 0.040261
Thermal correction to Gibbs Free Energy= -0.000115
Sum of electronic and zero-point Energies= -812.236019
Sum of electronic and thermal Energies= -812.228689
Sum of electronic and thermal Enthalpies= -812.227744
Sum of electronic and thermal Free Energies= -812.268120

Transition State

C	-1.06744500	0.55630600	1.49997600
O	-0.38860100	1.03193900	2.35825700
Na	1.54855600	1.66009000	1.27992700
O	0.63437200	0.93481300	-0.36436000
H	-0.75272200	-0.16653300	0.75005500
H	0.65307300	0.90492600	-1.31817700
Cl	-2.75271900	1.03198600	1.31495200

Zero-point correction= 0.032775 (Hartree/Particle)
Thermal correction to Energy= 0.039294
Thermal correction to Enthalpy= 0.040238
Thermal correction to Gibbs Free Energy= 0.001656
Sum of electronic and zero-point Energies= -812.231224
Sum of electronic and thermal Energies= -812.224706
Sum of electronic and thermal Enthalpies= -812.223761
Sum of electronic and thermal Free Energies= -812.262343

Product (6)

C	0.45088400	-1.39030300	-0.06981100
O	0.92416200	-0.63730100	0.76502700
Na	0.79511900	1.57069800	0.81147900
O	-0.23578100	-1.05585100	-1.11362300
H	0.58212700	-2.47448300	0.00858400
H	-0.35887900	-0.04795500	-1.19021700

Cl	-0.52773300	1.88835500	-1.23570100

Zero-point correction= 0.036332 (Hartree/Particle)
Thermal correction to Energy= 0.042674
Thermal correction to Enthalpy= 0.043618
Thermal correction to Gibbs Free Energy= 0.005102
Sum of electronic and zero-point Energies= -812.327204
Sum of electronic and thermal Energies= -812.320863
Sum of electronic and thermal Enthalpies= -812.319919
Sum of electronic and thermal Free Energies= -812.358435

IRC

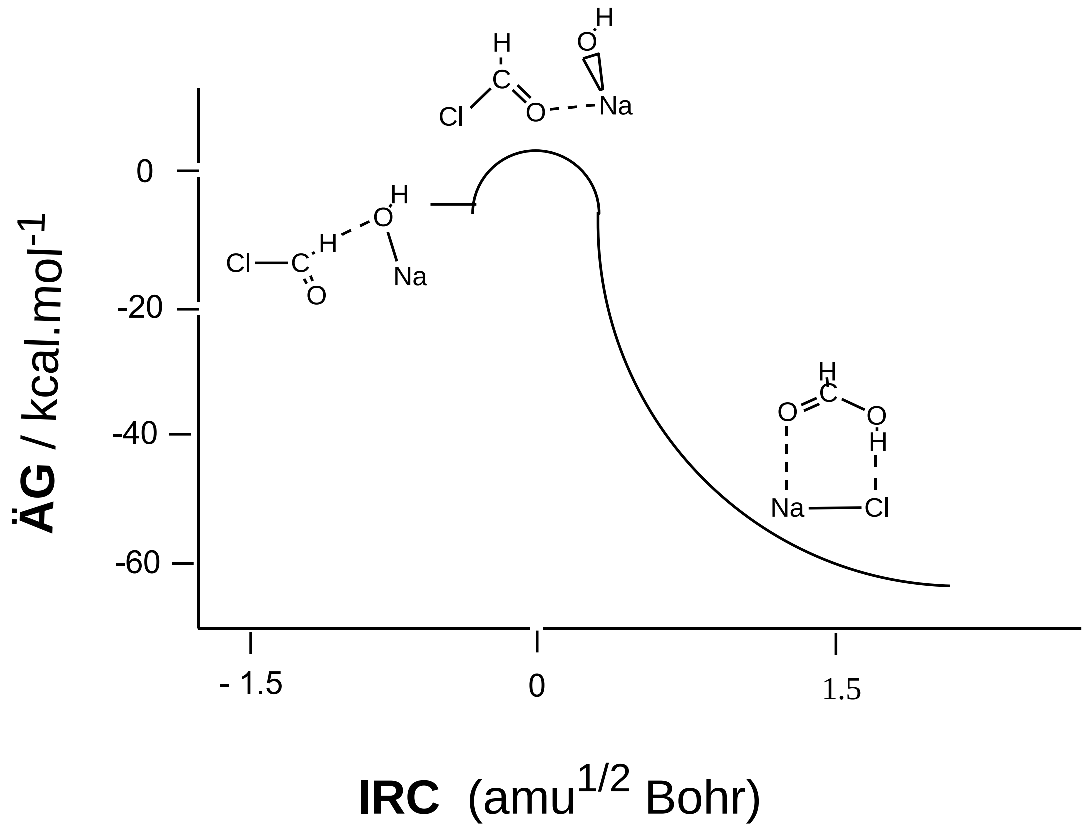

Figure-13

Intermediate (tt-7)

C	0.63246300	-1.04313800	0.46326600
O	0.20586800	0.10750400	-0.01269900
O	1.82783700	-0.82704300	0.94267800
H	-0.70357300	0.05648300	-0.40352500
Na	0.04825000	2.33381900	-0.50276600
O	1.69738700	-3.41125500	1.63733400
H	2.04754100	-4.16731500	1.16199800
H	0.86154100	-3.18287300	1.20234400
H	2.13340500	-1.68801400	1.29644700
Cl	-2.08570000	1.42339400	-1.30235000

Zero-point correction= 0.061064 (Hartree/Particle)
Thermal correction to Energy= 0.070868
Thermal correction to Enthalpy= 0.071812
Thermal correction to Gibbs Free Energy= 0.024144
Sum of electronic and zero-point Energies= -888.664423
Sum of electronic and thermal Energies= -888.654619
Sum of electronic and thermal Enthalpies= -888.653675
Sum of electronic and thermal Free Energies= -888.701343

Transition State

C	0.27941100	0.24878000	0.39910000
O	-0.24128600	1.34641600	-0.11554100
O	1.43106600	0.39549200	0.88153700
H	-1.15297000	1.22730500	-0.49328300
Na	-0.58076300	3.53560700	-0.65841200
O	0.96066300	-1.84634600	1.33080400
H	1.33829000	-2.51256600	0.75049300
H	0.18684100	-1.18336600	0.74496100
H	1.52970400	-0.78331900	1.24360600
Cl	-2.64179400	2.41353100	-1.39891300

Zero-point correction= 0.055465 (Hartree/Particle)
Thermal correction to Energy= 0.063687
Thermal correction to Enthalpy= 0.064632
Thermal correction to Gibbs Free Energy= 0.020206
Sum of electronic and zero-point Energies= -888.645386
Sum of electronic and thermal Energies= -888.637163
Sum of electronic and thermal Enthalpies= -888.636219
Sum of electronic and thermal Free Energies= -888.680645

Product (6)

C	0.90280700	-0.84743000	0.49150900
O	0.31894600	0.23465600	-0.02185800
O	2.00883300	-0.80892800	0.94779300
H	-0.61312300	0.10187800	-0.37283500
Na	-0.19417200	2.39300400	-0.57352100
O	1.54423400	-3.54462500	1.60040100
H	2.09928000	-4.22410900	1.21503600
H	0.29525700	-1.75976200	0.46229600
H	2.07037600	-2.73587000	1.57354000
Cl	-2.18962600	1.06284100	-1.17707600

Zero-point correction= 0.059866 (Hartree/Particle)
Thermal correction to Energy= 0.070264
Thermal correction to Enthalpy= 0.071208
Thermal correction to Gibbs Free Energy= 0.020816
Sum of electronic and zero-point Energies= -888.718257
Sum of electronic and thermal Energies= -888.707859
Sum of electronic and thermal Enthalpies= -888.706915
Sum of electronic and thermal Free Energies= -888.757307

IRC

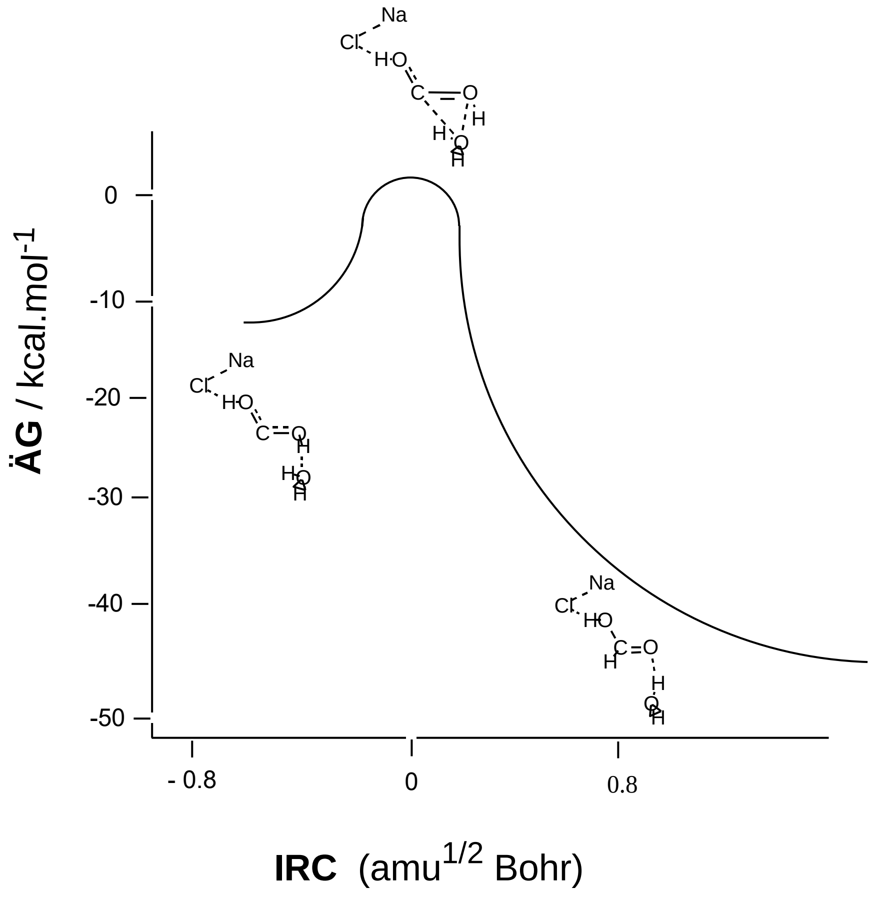

Figure-14

Intermediate (ct-7)

C	1.24785000	0.32921300	0.94901100
O	2.26389000	0.67930100	1.70650500
Na	-0.68514100	-0.14371300	-2.07425300
O	1.30248300	0.66953900	-1.83705800
H	1.37972500	0.58721300	-0.83075800
H	2.12211600	1.03929700	-2.16501800
Cl	-1.92775100	-0.91148400	-0.09047300
O	0.29825600	-0.16035900	1.66503600
H	-0.48961100	-0.43216600	1.08651000
H	2.06478200	0.48702200	2.64220100

Zero-point correction= 0.059727 (Hartree/Particle)
Thermal correction to Energy= 0.069234
Thermal correction to Enthalpy= 0.070178
Thermal correction to Gibbs Free Energy= 0.023283
Sum of electronic and zero-point Energies= -888.691491
Sum of electronic and thermal Energies= -888.681984
Sum of electronic and thermal Enthalpies= -888.681040
Sum of electronic and thermal Free Energies= -888.727934

Transition State

C	0.60166700	-0.58123100	0.39253200
O	1.14961800	-0.08313600	1.39046100
Na	-1.61458300	-0.93213300	-2.33236700
O	0.47559700	-0.34385600	-2.42080700
H	0.69211600	-0.38349300	-1.44348500
H	1.25207400	-0.02922900	-2.88413200
Cl	-2.84884600	-1.59555800	-0.28703400
O	-0.30232400	-1.46825000	1.05116800
H	-1.25741100	-1.54899100	0.65619900
H	0.24002500	-0.85349100	1.94138600

Zero-point correction= 0.052918 (Hartree/Particle)
Thermal correction to Energy= 0.062527
Thermal correction to Enthalpy= 0.063471
Thermal correction to Gibbs Free Energy= 0.015732
Sum of electronic and zero-point Energies= -888.635993
Sum of electronic and thermal Energies= -888.626385
Sum of electronic and thermal Enthalpies= -888.625441
Sum of electronic and thermal Free Energies= -888.673180

Product (4)

C	2.04026800	1.39339900	0.98158600
O	1.73580900	1.58554500	2.04371800
Na	0.03787300	-1.12139800	-0.96708900
O	1.40859300	0.34675600	-2.03080500
H	2.25968200	0.62544900	-1.68416600
H	0.77125200	0.98981800	-1.66312200
Cl	-1.06113100	1.08192200	-0.30655600
O	0.01994700	-1.27112700	1.27362300
H	-0.44170400	-0.40920900	1.22854600
H	-0.07398300	-1.60978800	2.16455800

Zero-point correction= 0.056220 (Hartree/Particle)
Thermal correction to Energy= 0.068513
Thermal correction to Enthalpy= 0.069457
Thermal correction to Gibbs Free Energy= 0.014916
Sum of electronic and zero-point Energies= -888.734068
Sum of electronic and thermal Energies= -888.721775
Sum of electronic and thermal Enthalpies= -888.720831
Sum of electronic and thermal Free Energies= -888.775372

IRC

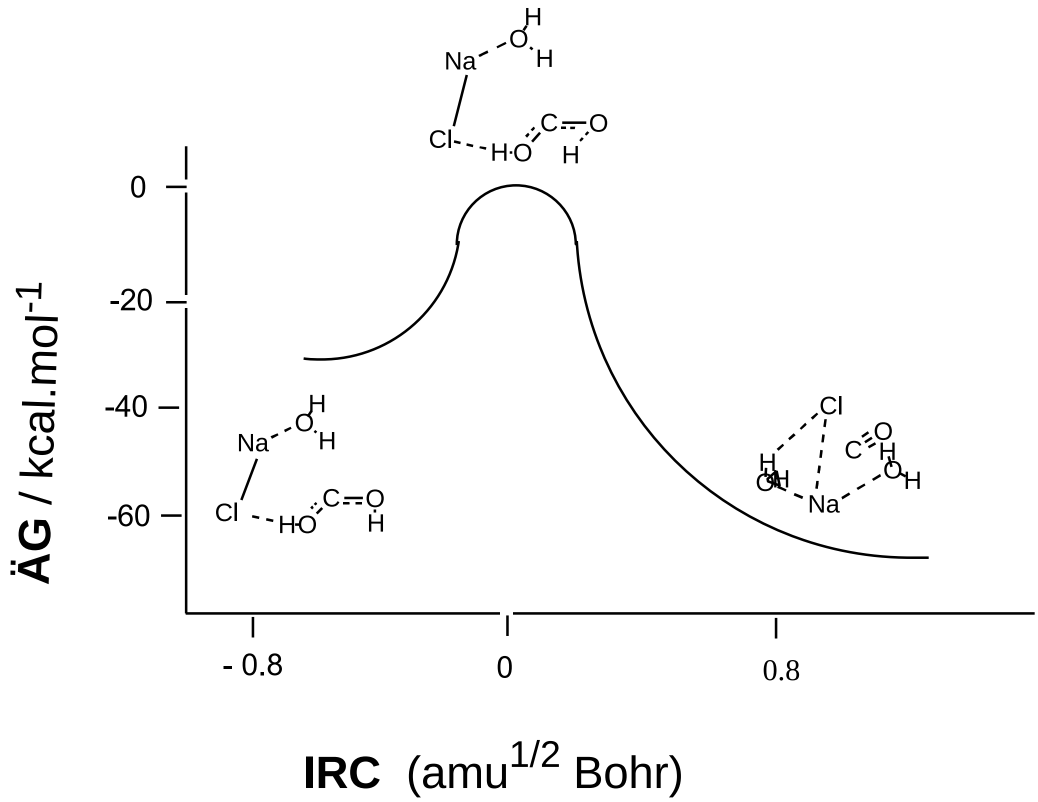

Figure-15

Intermediate (I-21)

C	-1.38338000	-1.11972900	1.33500400
O	0.52870400	-0.43958700	-0.63659400
O	-1.23785500	-2.23342100	1.31767100
H	1.11626700	-1.17338000	-0.47498000
Na	-0.12799400	1.23778100	-1.64068100
O	0.14800800	1.68782500	0.51674100
H	-0.45150900	1.64521800	1.26209400
H	0.41034200	0.69721300	0.28280600

Zero-point correction= 0.042543 (Hartree/Particle)
Thermal correction to Energy= 0.051505
Thermal correction to Enthalpy= 0.052450
Thermal correction to Gibbs Free Energy= 0.007326
Sum of electronic and zero-point Energies= -427.860159
Sum of electronic and thermal Energies= -427.851196
Sum of electronic and thermal Enthalpies= -427.850252
Sum of electronic and thermal Free Energies= -427.895375

Transition State

C	-0.42512500	0.45319700	0.30882400
O	0.24618300	0.30680300	-0.99432300
O	-0.73609900	-0.57051100	0.82878500
H	0.29979200	-0.64364800	-1.16892200
Na	0.57869500	2.41495400	-1.51265400
O	-0.30233100	3.02769900	0.26372500
H	-0.63722500	3.58807000	0.96143100
H	-0.46582300	1.83979100	0.49263100

Zero-point correction= 0.041777 (Hartree/Particle)
Thermal correction to Energy= 0.048620
Thermal correction to Enthalpy= 0.049565
Thermal correction to Gibbs Free Energy= 0.011036
Sum of electronic and zero-point Energies= -427.849187
Sum of electronic and thermal Energies= -427.842344
Sum of electronic and thermal Enthalpies= -427.841400
Sum of electronic and thermal Free Energies= -427.879929

Product (6)

C	-0.37692600	-0.97510600	0.66257800
O	0.25987400	-0.94587400	-0.57149300
O	-0.67246100	-2.01590600	1.15713100
H	0.36576600	-1.86718300	-0.84712800

Na	0.63357000	1.25423200	-1.14573000
O	-0.22165400	1.85187700	0.56812100
H	-0.47809600	2.58748300	1.11852800
H	-0.51331900	0.07244100	1.00648500

Zero-point correction= 0.047014 (Hartree/Particle)
Thermal correction to Energy= 0.054349
Thermal correction to Enthalpy= 0.055293
Thermal correction to Gibbs Free Energy= 0.015670
Sum of electronic and zero-point Energies= -427.853492
Sum of electronic and thermal Energies= -427.846158
Sum of electronic and thermal Enthalpies= -427.845214
Sum of electronic and thermal Free Energies= -427.884837

IRC

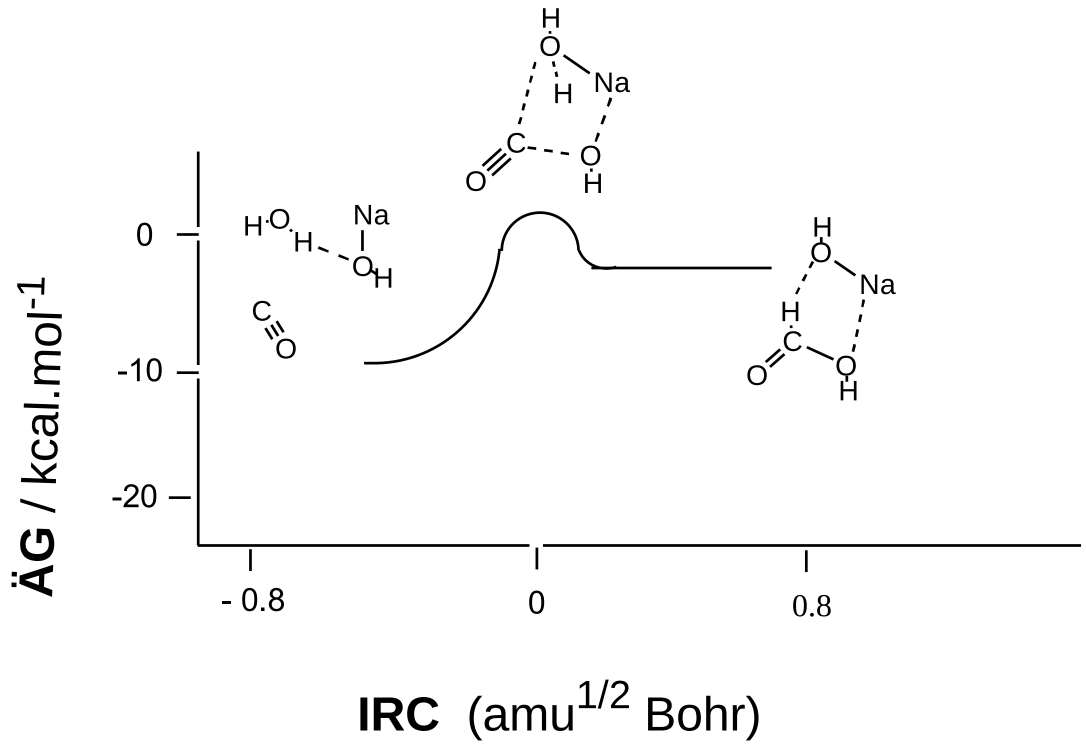

Figure-16

Intermediate (I-22)

C	-1.15775500	-0.19507900	0.45193800
O	-0.99972900	-1.30187500	0.54037400
H	0.64408100	1.58057200	-0.63023500
O	1.35884200	0.94350500	-0.69626000
H	1.88246900	1.07912700	0.09614000

Zero-point correction= 0.028104 (Hartree/Particle)
Thermal correction to Energy= 0.034475
Thermal correction to Enthalpy= 0.035419
Thermal correction to Gibbs Free Energy= -0.001494
Sum of electronic and zero-point Energies= -189.718874
Sum of electronic and thermal Energies= -189.712503
Sum of electronic and thermal Enthalpies= -189.711559
Sum of electronic and thermal Free Energies= -189.748472

Transition State

C	-0.22111200	0.94491700	0.07622900
O	-0.30987900	-0.09737900	0.52838000
H	0.09220400	2.01924000	-0.27173600
O	1.34647800	1.55255700	-0.58047300
H	2.05094800	1.55002100	0.08003400

Zero-point correction= 0.026110 (Hartree/Particle)
Thermal correction to Energy= 0.029971
Thermal correction to Enthalpy= 0.030915
Thermal correction to Gibbs Free Energy= 0.001386
Sum of electronic and zero-point Energies= -189.619114
Sum of electronic and thermal Energies= -189.615252
Sum of electronic and thermal Enthalpies= -189.614308
Sum of electronic and thermal Free Energies= -189.643838

Product (6)

C	-0.33822900	0.26108700	0.01073100
O	-0.46892800	-0.78866000	0.56128600
H	-1.15830000	0.92455700	-0.28367900
O	0.83592400	0.79997300	-0.33729000
H	1.53207600	0.18629400	-0.06157300

Zero-point correction= 0.034352 (Hartree/Particle)
Thermal correction to Energy= 0.037508
Thermal correction to Enthalpy= 0.038452

Thermal correction to Gibbs Free Energy= 0.010290
Sum of electronic and zero-point Energies= -189.731431
Sum of electronic and thermal Energies= -189.728275
Sum of electronic and thermal Enthalpies= -189.727330
Sum of electronic and thermal Free Energies= -189.755493

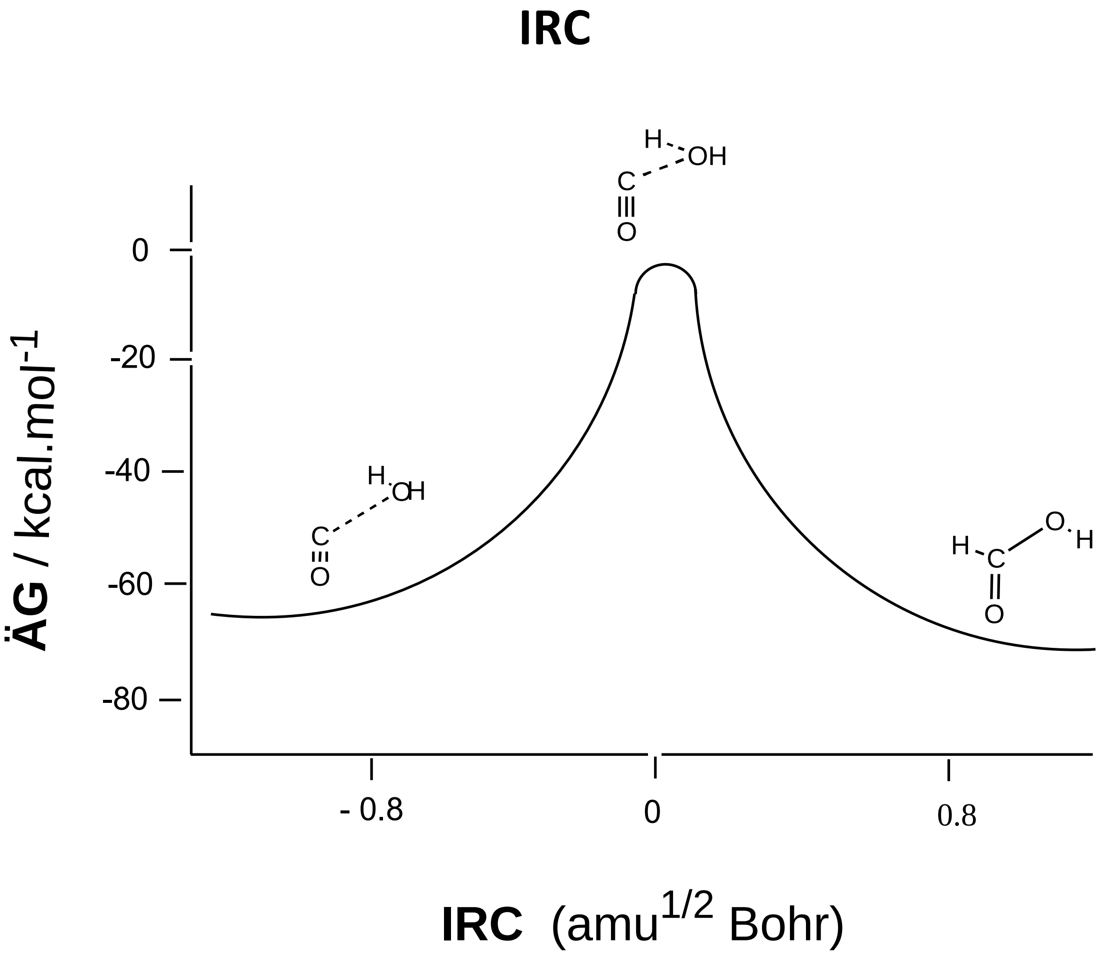

Figure-S1

Intermediate (I-2)

C	0.43824300	0.37625700	0.18988200
H	0.34979800	-0.66915900	0.46213600
Cl	1.39869400	1.22903800	1.39183800
Cl	1.20648100	0.46183800	-1.40537700
Cl	-1.20647000	1.04590100	0.11748800
O	-0.34496800	-2.56034300	0.15318700
H	-0.84534300	-2.38373300	-0.65915600
H	-0.89175800	-3.13975100	0.68567700
O	-1.51149900	-1.46477100	-2.15821900
H	-0.73431300	-1.23329000	-2.67472700
H	-1.80635900	-0.62992900	-1.77979000

Zero-point correction= 0.068704 (Hartree/Particle)
Thermal correction to Energy= 0.080230
Thermal correction to Enthalpy= 0.081174
Thermal correction to Gibbs Free Energy= 0.028904
Sum of electronic and zero-point Energies= -1572.115798
Sum of electronic and thermal Energies= -1572.104272
Sum of electronic and thermal Enthalpies= -1572.103328
Sum of electronic and thermal Free Energies= -1572.155598

Transition State

C	-0.79445300	0.43667900	-0.09762300
H	-0.69468600	0.32301600	-1.16798100
Cl	0.58576800	0.51474700	0.82670400
Cl	-0.83694300	2.01425700	-2.37794500
Cl	-2.22534100	1.00473700	0.54606200
O	-1.23898700	-1.68939600	-0.21358800
H	-1.63176900	-1.55640600	-1.10942100
H	-1.95423600	-1.97747800	0.36181600
O	-2.13662600	-0.78713400	-2.58867900
H	-1.90641700	-1.20745000	-3.42044300
H	-1.90750600	0.16298200	-2.71047600

Zero-point correction= 0.067758 (Hartree/Particle)
Thermal correction to Energy= 0.077769
Thermal correction to Enthalpy= 0.078714
Thermal correction to Gibbs Free Energy= 0.030631
Sum of electronic and zero-point Energies= -1572.035522
Sum of electronic and thermal Energies= -1572.025511
Sum of electronic and thermal Enthalpies= -1572.024567
Sum of electronic and thermal Free Energies= -1572.072649

Product (1)

C	0.13491300	-0.41862300	0.74250700
H	0.35484600	0.10933300	-0.17723300
Cl	1.63120100	-0.49517700	1.71292400
Cl	-0.56866100	1.34784300	-2.36163900
Cl	-1.09856600	0.51735100	1.62130300
O	-0.31836400	-1.67550300	0.43190300
H	-0.96658000	-1.86734400	-1.38890700
H	-0.55491200	-2.14278900	1.24278100
O	-1.10166700	-1.64555500	-2.32015900
H	-0.44734800	-2.14891300	-2.81059100
H	-0.87328000	0.07229800	-2.44808600

Zero-point correction= 0.068003 (Hartree/Particle)
Thermal correction to Energy= 0.078527
Thermal correction to Enthalpy= 0.079472
Thermal correction to Gibbs Free Energy= 0.027397
Sum of electronic and zero-point Energies= -1572.125274
Sum of electronic and thermal Energies= -1572.114749
Sum of electronic and thermal Enthalpies= -1572.113805
Sum of electronic and thermal Free Energies= -1572.165879

IRC

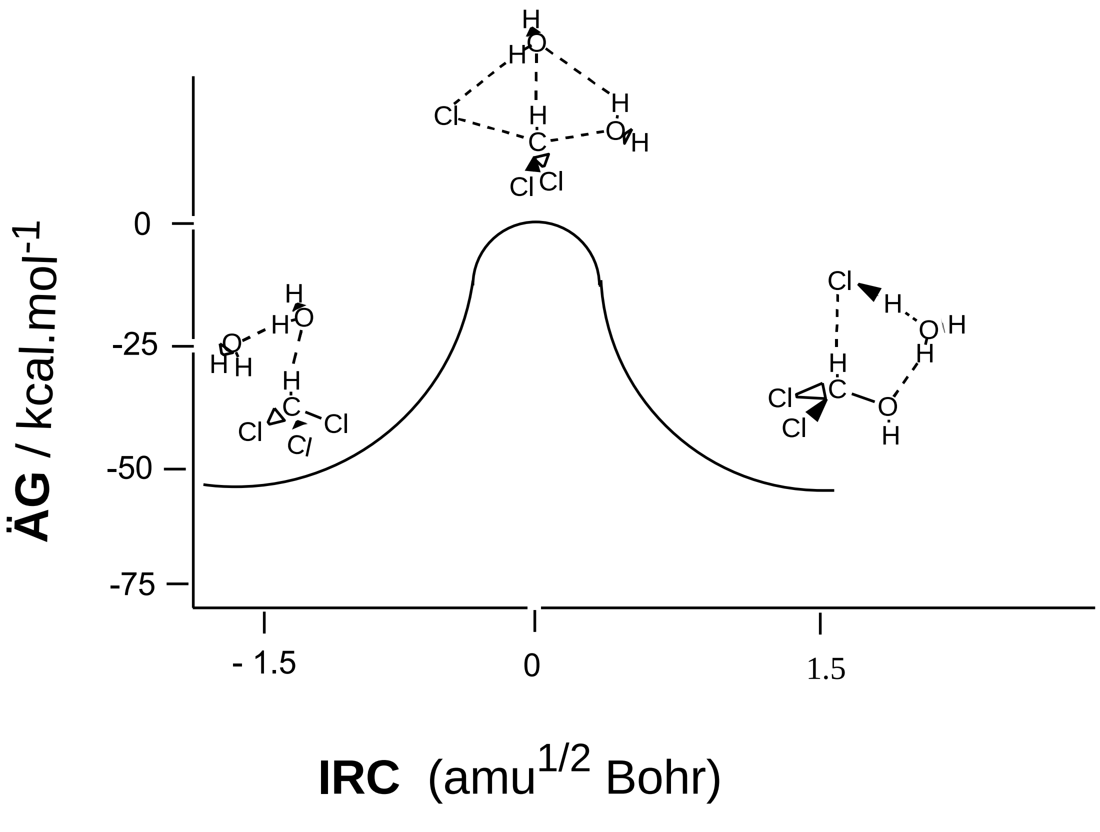

Figure-S2

Intermediate (I-4)

C	-0.53141800	0.20583300	0.11495800
H	0.29089900	0.39464800	0.86095300
Cl	0.17089500	-1.02320700	-1.03201900
Cl	-1.95571900	-0.48359200	0.88429800
Cl	-0.93173600	1.66248200	-0.78765700
O	1.75068700	0.29687100	1.62893700
H	1.83989100	0.84902700	2.40173200
Na	2.55142800	-1.05033800	0.37160300

Zero-point correction= 0.031997 (Hartree/Particle)
Thermal correction to Energy= 0.041050
Thermal correction to Enthalpy= 0.041995
Thermal correction to Gibbs Free Energy= -0.004426
Sum of electronic and zero-point Energies= -1657.414248
Sum of electronic and thermal Energies= -1657.405194
Sum of electronic and thermal Enthalpies= -1657.404250
Sum of electronic and thermal Free Energies= -1657.450671

Transition State

C	-0.23719700	0.03873300	0.50084000
H	-0.60964600	0.07583300	1.49951000
Cl	0.17711200	-1.48780400	-0.25332400
Cl	-2.42974300	-0.10706000	0.01277600
Cl	0.02239700	1.48444300	-0.45858400
O	1.51496500	0.12884100	1.32712800
H	1.45420200	0.86270600	1.94324100
Na	2.48140000	-0.03906500	-0.52061200

Zero-point correction= 0.032944 (Hartree/Particle)
Thermal correction to Energy= 0.041010
Thermal correction to Enthalpy= 0.041954
Thermal correction to Gibbs Free Energy= -0.001077
Sum of electronic and zero-point Energies= -1657.358936
Sum of electronic and thermal Energies= -1657.350870
Sum of electronic and thermal Enthalpies= -1657.349926
Sum of electronic and thermal Free Energies= -1657.392957

Product (1)

C	0.49207300	-0.12270800	0.08917600
H	0.36060000	0.79979700	-0.46647700

Cl	2.13432700	-0.19559200	0.72742300
Cl	-2.08149000	-0.51901400	-2.21703400
Cl	-0.65959400	0.09428100	1.54889800
O	0.17570600	-1.23211300	-0.58616200
H	-0.44521800	-0.97858600	-1.35328600
Na	-2.25798500	-1.52220200	0.05401600

Zero-point correction= 0.035342 (Hartree/Particle)
Thermal correction to Energy= 0.043520
Thermal correction to Enthalpy= 0.044464
Thermal correction to Gibbs Free Energy= -0.000199
Sum of electronic and zero-point Energies= -1657.490599
Sum of electronic and thermal Energies= -1657.482422
Sum of electronic and thermal Enthalpies= -1657.481478
Sum of electronic and thermal Free Energies= -1657.526141

IRC

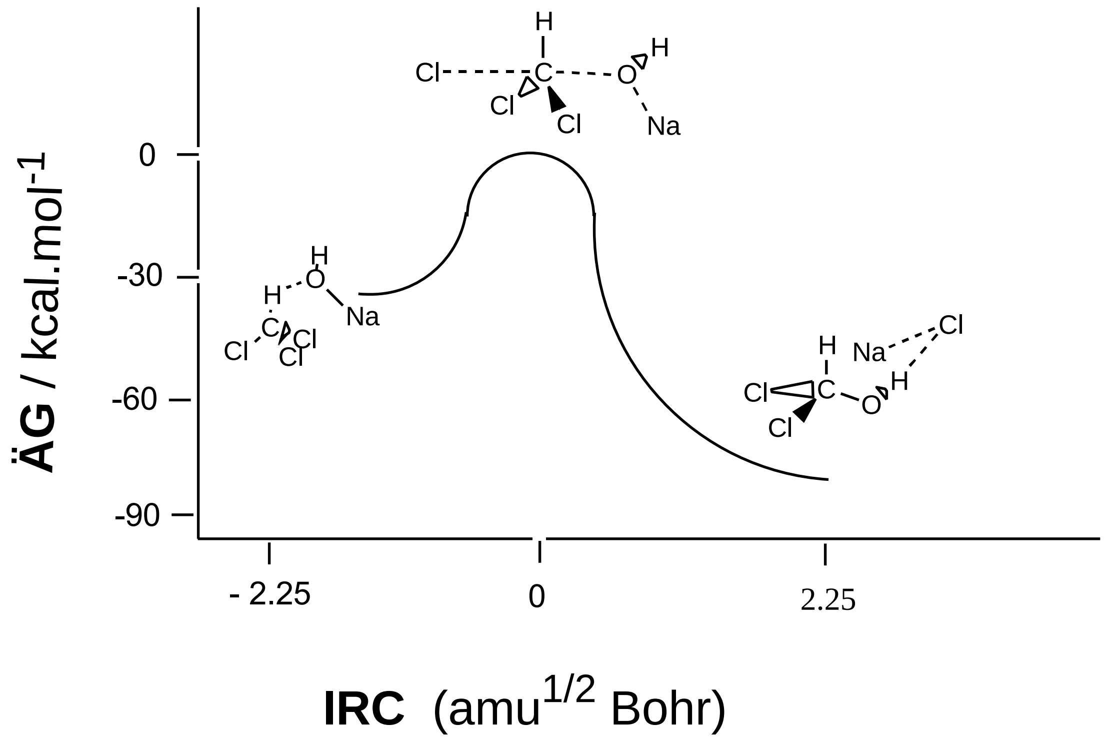

Figure-S3

Intermediate (I-6)

C	-0.47606000	0.42542100	0.55943100
H	0.41967300	0.75630300	0.00624400
Cl	-0.13110100	-1.26542100	1.07682100
Cl	-0.76646900	1.42767600	1.97368900
Cl	-1.86764900	0.43576200	-0.52743600
O	1.86174300	0.59465700	-1.04249600
H	2.35874200	1.39016100	-1.22149400
Na	1.94545100	-1.50258700	-0.84197100
O	0.67162900	-0.95278300	-2.60092100
H	-0.26980500	-0.81975400	-2.71635100
H	1.05199600	-0.10650900	-2.16913200

Zero-point correction= 0.057760 (Hartree/Particle)
Thermal correction to Energy= 0.069126
Thermal correction to Enthalpy= 0.070071
Thermal correction to Gibbs Free Energy= 0.018348
Sum of electronic and zero-point Energies= -1733.853453
Sum of electronic and thermal Energies= -1733.842087
Sum of electronic and thermal Enthalpies= -1733.841143
Sum of electronic and thermal Free Energies= -1733.892866

Transition State

C	-0.80799300	-0.02242500	1.14437400
H	-0.23579500	0.68419200	1.70162500
Cl	-0.68747700	-1.73262000	1.52194100
Cl	-2.20266600	0.39205100	2.93749000
Cl	-1.96234500	0.45851600	-0.05194600
O	0.66447000	-0.10839100	-0.08098400
H	0.86816500	0.81172000	-0.26170800
Na	1.28644100	-2.13514700	-0.31953900
O	-0.09954500	-1.79604500	-2.05393700
H	-0.98607700	-2.02964700	-2.33459900
H	-0.16448700	-0.93420900	-1.59714400

Zero-point correction= 0.058343 (Hartree/Particle)
Thermal correction to Energy= 0.069209
Thermal correction to Enthalpy= 0.070154
Thermal correction to Gibbs Free Energy= 0.020586
Sum of electronic and zero-point Energies= -1733.791746
Sum of electronic and thermal Energies= -1733.780880
Sum of electronic and thermal Enthalpies= -1733.779936
Sum of electronic and thermal Free Energies= -1733.829504

Product (2)

C	-0.99536100	0.44003500	-0.13879400
H	-1.38982100	-0.11834300	0.70712100
Cl	0.34778200	-2.11197400	-0.00326000
Cl	2.73452000	1.75269400	1.24270500
Cl	-1.97224100	0.21514900	-1.57525200
O	-0.05901500	1.17893200	-0.13192900
H	1.45385500	1.86436200	1.13333300
Na	1.94608900	-0.24714700	-0.58993900
O	1.18684200	-0.79560600	-2.65376000
H	0.66964600	-1.46906900	-2.17186300
H	0.58210400	-0.37929400	-3.27103500

Zero-point correction= 0.055131 (Hartree/Particle)
Thermal correction to Energy= 0.068120
Thermal correction to Enthalpy= 0.069064
Thermal correction to Gibbs Free Energy= 0.012417
Sum of electronic and zero-point Energies= -1733.919687
Sum of electronic and thermal Energies= -1733.906698
Sum of electronic and thermal Enthalpies= -1733.905754
Sum of electronic and thermal Free Energies= -1733.962400

IRC

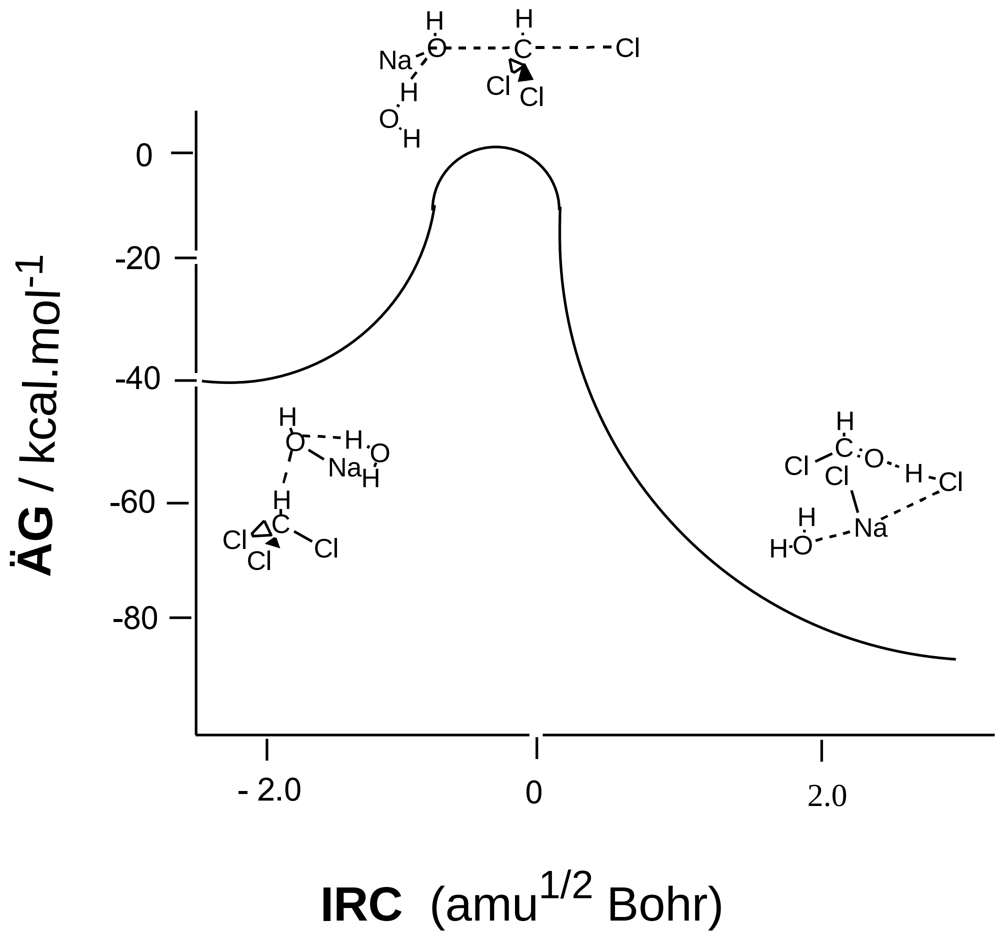

Figure-S5

Intermediate-(I-10)

C	-0.40924100	-0.05722000	0.04000100
H	-1.35287000	-0.45545200	-0.30366100
Cl	0.87518600	-0.90515500	-0.83630800
Cl	-0.33922900	1.67594500	-0.28829000
Cl	-0.29382600	-0.37569700	1.77404000
O	-1.93878500	-2.63711200	-0.12802500
H	-1.77904700	-2.82796300	0.79971700
O	2.49740800	1.75727700	-2.29945500
H	1.62269700	1.46539100	-2.56459700
H	2.51129200	1.60543600	-1.35203200
H	-1.26242600	-3.12669700	-0.60258100

Zero-point correction= 0.066723 (Hartree/Particle)
Thermal correction to Energy= 0.079589
Thermal correction to Enthalpy= 0.080533
Thermal correction to Gibbs Free Energy= 0.024109

Sum of electronic and zero-point Energies= -1572.106360
Sum of electronic and thermal Energies= -1572.093494
Sum of electronic and thermal Enthalpies= -1572.092550
Sum of electronic and thermal Free Energies= -1572.148974

Transition State-1

C	-1.14593400	0.09854700	0.46278400
H	-1.68486800	-1.20696200	-0.17944200
Cl	0.97648400	-1.60572000	-0.81265600
Cl	-0.93042800	1.48733300	-0.42328600
Cl	-0.56270500	0.19148200	2.04240500
O	-1.71416700	-2.12672100	-0.77682800
H	-1.92740200	-2.88398500	-0.21913000
O	1.31603200	1.23502900	-2.38639000
H	1.24324800	0.31619300	-2.09086400
H	2.25070000	1.43490200	-2.30971300
H	-0.61978100	-2.11211200	-0.94412000

Zero-point correction= 0.061941 (Hartree/Particle)
Thermal correction to Energy= 0.072524
Thermal correction to Enthalpy= 0.073468
Thermal correction to Gibbs Free Energy= 0.023182
Sum of electronic and zero-point Energies= -1572.022245
Sum of electronic and thermal Energies= -1572.011662
Sum of electronic and thermal Enthalpies= -1572.010718
Sum of electronic and thermal Free Energies= -1572.061004

Intermediate-(I-11)

C	-1.13857800	0.38033200	1.15483700
H	-1.43922400	-1.38330500	0.29097200
Cl	1.06924700	-1.89728700	-2.01138900
Cl	-0.04643800	1.13815300	0.12667600
Cl	-1.54666800	1.36530600	2.48107400
O	-1.34834500	-2.12238900	-0.34429000
H	-1.33484800	-2.93090700	0.17384600
O	1.75734500	1.40928400	-2.08089400
H	1.55082500	0.47883300	-2.23022000
H	2.70895900	1.47082100	-2.18150400
H	-0.03869200	-1.97602400	-1.25990500

Zero-point correction= 0.061885 (Hartree/Particle)
Thermal correction to Energy= 0.074568
Thermal correction to Enthalpy= 0.075512
Thermal correction to Gibbs Free Energy= 0.017778
Sum of electronic and zero-point Energies= -1572.028033
Sum of electronic and thermal Energies= -1572.015350
Sum of electronic and thermal Enthalpies= -1572.014406

Sum of electronic and thermal Free Energies= -1572.072140

IRC

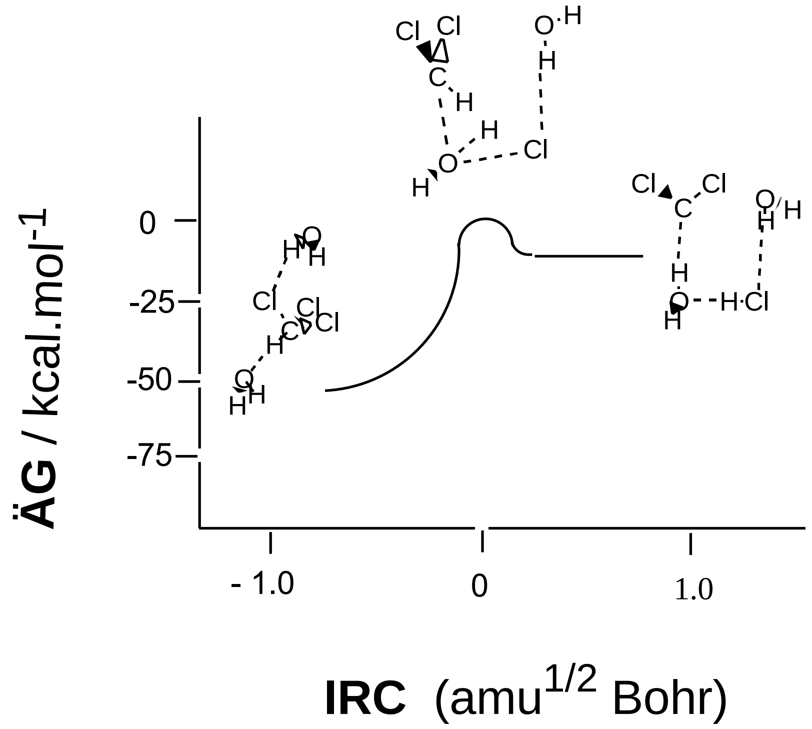

Transition State-2

C	-0.30033900	-0.17846500	0.12127100
H	-0.76212900	-0.54309100	-1.05380100
Cl	1.08390700	-1.06860900	0.68693300
Cl	-1.25237200	2.14381900	-1.93679700
Cl	-1.04718700	0.74285800	1.42171900
O	-1.32507800	-1.27559600	-0.35566300
H	-2.23058100	-0.83709400	-0.34759600
O	-3.52959000	0.13520000	-0.58845400
H	-3.20426500	0.88164000	-1.11186400
H	-3.82061500	0.51747900	0.24485000
H	-0.56039500	1.28918300	-1.18991200

Zero-point correction= 0.061424 (Hartree/Particle)
Thermal correction to Energy= 0.071477
Thermal correction to Enthalpy= 0.072421
Thermal correction to Gibbs Free Energy= 0.024309
Sum of electronic and zero-point Energies= -1572.002231
Sum of electronic and thermal Energies= -1571.992179
Sum of electronic and thermal Enthalpies= -1571.991234

Sum of electronic and thermal Free Energies= -1572.039347

Product (1)

C	0.38739000	-0.97357300	0.15746000
H	0.47304800	-0.66036500	-0.88019000
Cl	1.81388800	-1.92266300	0.56874700
Cl	-0.47831200	2.01068100	-2.04621000
Cl	0.46439700	0.62922200	1.08953900
O	-0.72054000	-1.65833600	0.46072900
H	-1.48976400	-1.18558700	0.06859100
O	-2.62649500	-0.06362700	-0.59172200
H	-2.28431600	0.50413700	-1.29206500
H	-2.93812500	0.53472900	0.09290300
H	-0.02745200	1.80596000	-0.85003100

Zero-point correction= 0.067628 (Hartree/Particle)
Thermal correction to Energy= 0.078257
Thermal correction to Enthalpy= 0.079201
Thermal correction to Gibbs Free Energy= 0.028276
Sum of electronic and zero-point Energies= -1572.126121
Sum of electronic and thermal Energies= -1572.115492
Sum of electronic and thermal Enthalpies= -1572.114548
Sum of electronic and thermal Free Energies= -1572.165473

IRC

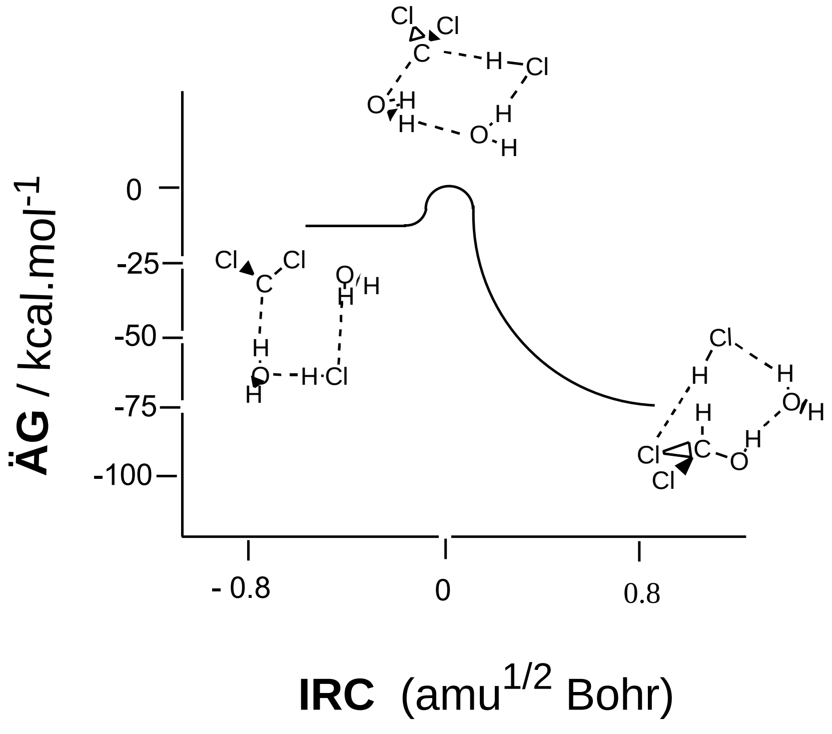

Figure-S6

Intermediate (I-17)

C	1.36422000	0.54695300	-0.96188100
H	0.30506700	0.50252600	-1.24090000
Cl	1.57572500	-0.28991600	0.64271100
O	2.27917000	1.02135900	-1.51409700
O	-1.65776500	0.05190500	-1.16038800
H	-2.38974800	0.66904100	-1.20490300
H	-1.80861500	-0.48673400	-0.36641900
O	-1.55465100	-1.38830400	1.22362100
H	-1.52183400	-2.34701700	1.19021800
H	-0.64489600	-1.10537700	1.37167300

Zero-point correction= 0.068246 (Hartree/Particle)
Thermal correction to Energy= 0.078238
Thermal correction to Enthalpy= 0.079182
Thermal correction to Gibbs Free Energy= 0.031770
Sum of electronic and zero-point Energies= -726.932047
Sum of electronic and thermal Energies= -726.922056
Sum of electronic and thermal Enthalpies= -726.921111
Sum of electronic and thermal Free Energies= -726.968523

Transition State

C	1.31586000	0.09598000	1.24404300
H	0.04391600	0.31790700	1.13576500
Cl	0.87626700	1.34760800	3.31691400
O	2.39210100	-0.18671400	1.08557200
O	-1.15739500	0.69186100	1.40080900
H	-1.43457100	1.45231200	0.87753600
H	-0.87640500	1.04365500	2.29159300
O	0.14132400	-1.62526100	2.81566400
H	0.51866700	-2.43285900	3.16946800
H	0.36474100	-0.92473900	3.44570300

Zero-point correction= 0.063780 (Hartree/Particle)
Thermal correction to Energy= 0.072473
Thermal correction to Enthalpy= 0.073417
Thermal correction to Gibbs Free Energy= 0.030635
Sum of electronic and zero-point Energies= -726.900963
Sum of electronic and thermal Energies= -726.892270
Sum of electronic and thermal Enthalpies= -726.891326
Sum of electronic and thermal Free Energies= -726.934108

Product (4)

C	0.94604900	-0.32088100	-1.86663900
H	-1.66616600	-0.48152000	-0.36067400
Cl	0.18361000	1.53508000	1.03803600
O	1.97691100	-0.66975300	-1.59888900
O	-1.91072100	0.40843400	-0.67181400
H	-1.57578300	0.45530300	-1.57271000
H	-0.83658400	1.16452500	0.23973600
O	-0.47734300	-1.60261900	0.54017400
H	0.00785700	-0.92226200	1.02579200
H	-0.63325800	-2.31833300	1.15970600

Zero-point correction= 0.063743 (Hartree/Particle)
Thermal correction to Energy= 0.074429
Thermal correction to Enthalpy= 0.075373
Thermal correction to Gibbs Free Energy= 0.026533
Sum of electronic and zero-point Energies= -726.945196
Sum of electronic and thermal Energies= -726.934510
Sum of electronic and thermal Enthalpies= -726.933566
Sum of electronic and thermal Free Energies= -726.982406

IRC

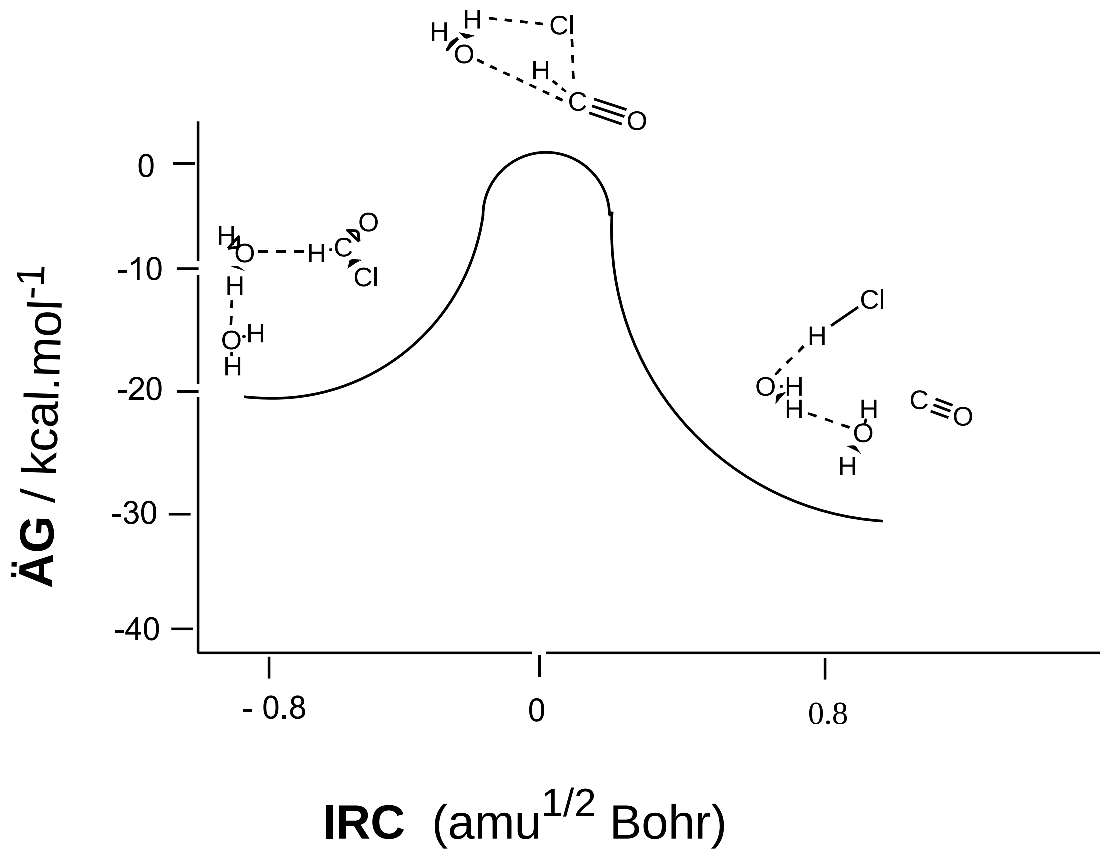

Figure-S7

Intermediate (I-23)

C	-0.53514700	-0.85326100	1.06358500
O	0.40818900	-0.13934700	-1.57091900
O	-0.70757800	-1.95465100	0.95362800
H	1.26186300	-0.37129700	-1.93971500
O	0.39740400	2.05189800	0.23177000
H	-0.30885700	2.69699900	0.15637600
H	0.05417900	1.35785400	0.80599400
H	0.52603200	0.73504300	-1.17458000

Zero-point correction= 0.053815 (Hartree/Particle)
Thermal correction to Energy= 0.062725
Thermal correction to Enthalpy= 0.063669
Thermal correction to Gibbs Free Energy= 0.020655
Sum of electronic and zero-point Energies= -266.132071
Sum of electronic and thermal Energies= -266.123161
Sum of electronic and thermal Enthalpies= -266.122217
Sum of electronic and thermal Free Energies= -266.165231

Transition State

C	-0.63344500	0.39547700	0.65705300
O	-0.10674800	0.85292700	-0.95148700
O	-0.78432600	-0.73184900	0.82180100
H	0.52381700	0.23438000	-1.33074200
O	0.02485300	2.75172400	0.37853600
H	-0.63223900	3.42562300	0.18209000
H	-0.46485100	1.76527100	0.88711800
H	0.14403700	2.03550000	-0.48940900

Zero-point correction= 0.051180 (Hartree/Particle)
Thermal correction to Energy= 0.056219
Thermal correction to Enthalpy= 0.057163
Thermal correction to Gibbs Free Energy= 0.023778
Sum of electronic and zero-point Energies= -266.063835
Sum of electronic and thermal Energies= -266.058796
Sum of electronic and thermal Enthalpies= -266.057852
Sum of electronic and thermal Free Energies= -266.091237

Product (6)

C	-0.20780300	-0.74804400	0.27771500
O	0.32412700	-0.34417500	-0.89468200
O	-0.44976200	-1.88584200	0.53629200
H	0.46607300	-1.13275000	-1.43741100

O	0.37640900	2.30471300	0.22472900
H	-0.13066600	3.04705600	-0.10840400
H	-0.37183000	0.11814000	0.92484500
H	0.58263100	1.76706000	-0.54567200

Zero-point correction= 0.058321 (Hartree/Particle)
Thermal correction to Energy= 0.065168
Thermal correction to Enthalpy= 0.066113
Thermal correction to Gibbs Free Energy= 0.027832
Sum of electronic and zero-point Energies= -266.140352
Sum of electronic and thermal Energies= -266.133504
Sum of electronic and thermal Enthalpies= -266.132560
Sum of electronic and thermal Free Energies= -266.170840

IRC

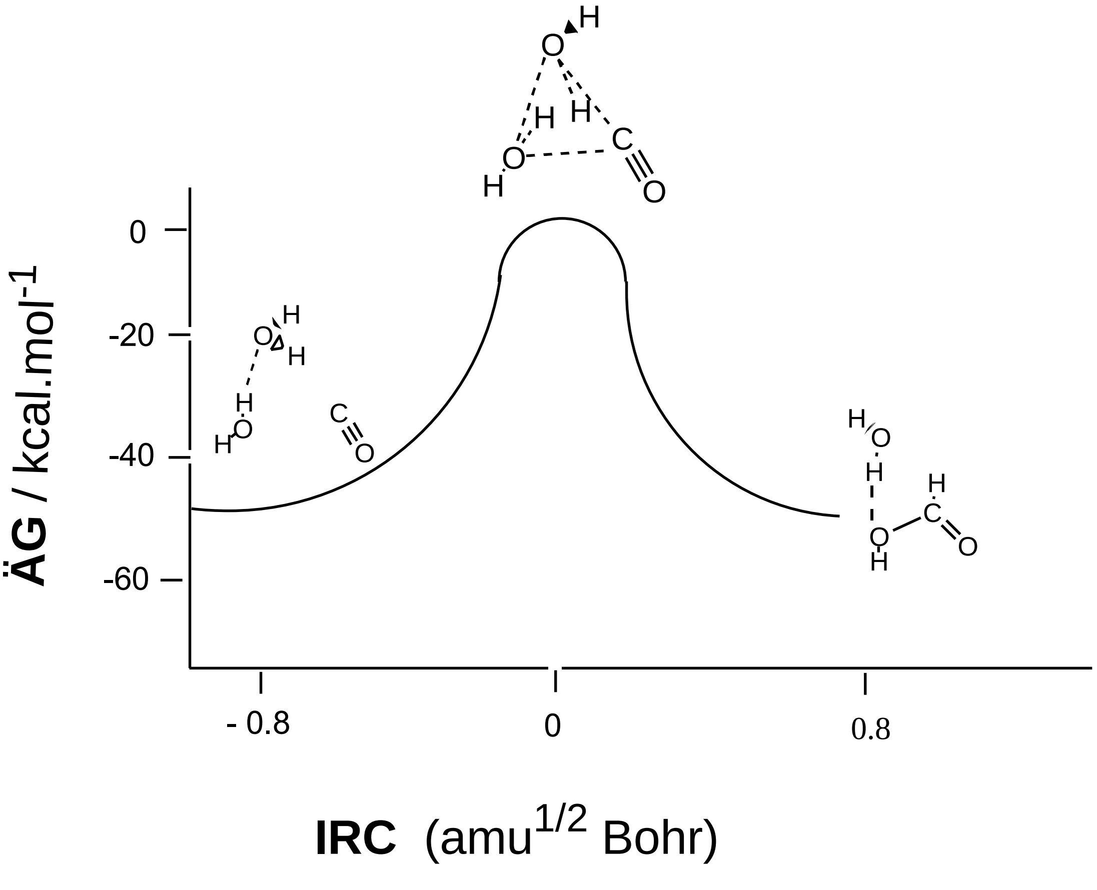

Calculations at the ωB97XD/cc-pVTZ level

Table S5. Gas-phase Cartesian coordinates of all Reactants and Products along with all corresponding calculated Energy values at the ωB97XD/cc-pVTZ level.

A-Reactants

$CHCl_3$

C	-2.59815000	-2.63735600	0.02463400
H	-2.23786200	-2.12770500	-0.85757900
Cl	-1.98339400	-1.76884100	1.43814800
Cl	-4.36672900	-2.61149300	-0.02115200
Cl	-1.98401000	-4.29606900	-0.02066800

Zero-point correction= 0.020147 (Hartree/Particle)
Thermal correction to Energy= 0.024570
Thermal correction to Enthalpy= 0.025514
Thermal correction to Gibbs Free Energy= -0.009020
Sum of electronic and zero-point Energies= -1419.327660
Sum of electronic and thermal Energies= -1419.323237
Sum of electronic and thermal Enthalpies= -1419.322293
Sum of electronic and thermal Free Energies= -1419.356826

NaOH

H	-1.97657200	-2.25039000	-0.64580700
Na	-4.72439700	-2.71334100	0.15610700
O	-2.87644200	-2.40156200	-0.38395100

Zero-point correction= 0.010909 (Hartree/Particle)
Thermal correction to Energy= 0.014481
Thermal correction to Enthalpy= 0.015425
Thermal correction to Gibbs Free Energy= -0.006003
Sum of electronic and zero-point Energies= -238.119285
Sum of electronic and thermal Energies= -238.115714
Sum of electronic and thermal Enthalpies= -238.114770
Sum of electronic and thermal Free Energies= -238.136198

H_2O

H	-2.29561500	-2.15184800	-0.81648000
O	-2.55755300	-2.61203400	-0.01941000
H	-3.51424400	-2.60142600	-0.03776200

Zero-point correction= 0.021690 (Hartree/Particle)
Thermal correction to Energy= 0.024525
Thermal correction to Enthalpy= 0.025469
Thermal correction to Gibbs Free Energy= 0.003406

Sum of electronic and zero-point Energies= -76.413146
Sum of electronic and thermal Energies= -76.410311
Sum of electronic and thermal Enthalpies= -76.409367
Sum of electronic and thermal Free Energies= -76.431430

$PhCCl_3$

C	-1.83008000	-0.51122500	-0.11868500
Cl	-0.86350300	-2.00289600	-0.23714400
Cl	-3.13078600	-0.61102400	-1.35120400
C	-1.02088800	0.75546000	-0.32185500
C	0.34204200	0.73710800	-0.56974800
C	1.03307500	1.92824300	-0.74695500
C	0.36997300	3.13881800	-0.67781900
C	-0.99613500	3.15928200	-0.42935400
C	-1.68742000	1.97767400	-0.25258400
H	0.87335600	-0.19962700	-0.62645500
H	2.09642600	1.89970300	-0.93983700
H	0.91078900	4.06478000	-0.81626800
H	-1.52513300	4.10039200	-0.37316400
H	-2.75074400	2.00124100	-0.05956900
Cl	-2.59851500	-0.49216600	1.50307200

Zero-point correction= 0.101052 (Hartree/Particle)
Thermal correction to Energy= 0.110101
Thermal correction to Enthalpy= 0.111045
Thermal correction to Gibbs Free Energy= 0.064665
Sum of electronic and zero-point Energies= -1650.286339
Sum of electronic and thermal Energies= -1650.277291
Sum of electronic and thermal Enthalpies= -1650.276347
Sum of electronic and thermal Free Energies= -1650.322726

B-Products

$CH(OH)Cl_2$ (1)

C	0.18047400	0.11813000	0.67195300
H	-0.29515000	-0.47908900	-0.09427400
Cl	1.49501900	-0.88649500	1.38564500
Cl	-1.05716600	0.42990100	1.94382200
O	0.64635400	1.25633700	0.11742400
H	1.07172600	1.79529300	0.79102900

Zero-point correction= 0.034395 (Hartree/Particle)
Thermal correction to Energy= 0.038868
Thermal correction to Enthalpy= 0.039812
Thermal correction to Gibbs Free Energy= 0.006016
Sum of electronic and zero-point Energies= -1034.938028

Sum of electronic and thermal Energies= -1034.933556
Sum of electronic and thermal Enthalpies= -1034.932611
Sum of electronic and thermal Free Energies= -1034.966407

HCOCl (2)

C	-0.85714400	-1.21150000	-0.25918500
H	-0.71504800	-0.27624000	-0.80976700
Cl	0.45817600	-1.42078100	0.91703000
O	-1.73234200	-1.98282100	-0.39645300

Zero-point correction= 0.019326 (Hartree/Particle)
Thermal correction to Energy= 0.022557
Thermal correction to Enthalpy= 0.023501
Thermal correction to Gibbs Free Energy= -0.005869
Sum of electronic and zero-point Energies= -574.125547
Sum of electronic and thermal Energies= -574.122316
Sum of electronic and thermal Enthalpies= -574.121372
Sum of electronic and thermal Free Energies= -574.150741

CO (4)

C	-0.81919400	-1.16574800	-0.26157600
O	-1.64218200	-1.92386500	-0.36788800

Zero-point correction= 0.005115 (Hartree/Particle)
Thermal correction to Energy= 0.007475
Thermal correction to Enthalpy= 0.008420
Thermal correction to Gibbs Free Energy= -0.014000
Sum of electronic and zero-point Energies= -113.311402
Sum of electronic and thermal Energies= -113.309041
Sum of electronic and thermal Enthalpies= -113.308097
Sum of electronic and thermal Free Energies= -113.330517

$HCCl(OH)_2$ (5)

C	-0.89637000	-0.83659900	0.27893300
H	-1.42715900	-1.71142400	0.63444100
Cl	0.77337600	-0.89938300	1.06290100
O	-0.76152300	-0.94457400	-1.07647700
O	-1.56063900	0.27175900	0.72314700
H	-0.13984300	-0.28720500	-1.39884200
H	-1.02205500	1.05518000	0.58626700

Zero-point correction= 0.048000 (Hartree/Particle)
Thermal correction to Energy= 0.052899

Thermal correction to Enthalpy= 0.053843
Thermal correction to Gibbs Free Energy= 0.019728
Sum of electronic and zero-point Energies= -650.543555
Sum of electronic and thermal Energies= -650.538657
Sum of electronic and thermal Enthalpies= -650.537713
Sum of electronic and thermal Free Energies= -650.571827

HCOOH (6)

C	0.26035600	0.10760500	0.74241300
H	-0.30184700	-0.55028000	0.06952900
O	0.58084800	1.23908000	0.10777000
H	1.07240500	1.78938700	0.73049500
O	0.54394300	-0.14815400	1.87353800

Zero-point correction= 0.034266 (Hartree/Particle)
Thermal correction to Energy= 0.037421
Thermal correction to Enthalpy= 0.038365
Thermal correction to Gibbs Free Energy= 0.010206
Sum of electronic and zero-point Energies= -189.741831
Sum of electronic and thermal Energies= -189.738676
Sum of electronic and thermal Enthalpies= -189.737732
Sum of electronic and thermal Free Energies= -189.765891

Table S6. Gas-phase Cartesian coordinates of all stationary points along with all corresponding calculated Energy values found, and the IRC plots in the Potential Energy Surfaces shown in the Figures.

Figure-1

Intermediate (I-1)

C	0.22417300	0.06745400	0.14516500
H	0.23670400	-1.01463800	0.14933100
Cl	1.67493700	0.67822300	0.94496600
Cl	0.16441000	0.58468800	-1.55136000
Cl	-1.24202800	0.57797000	1.00524100
O	-1.02478500	-2.69092600	-0.54230500
H	-1.13556600	-2.47115100	-1.46828200
H	-1.86378100	-2.46877100	-0.13640000

Zero-point correction= 0.043684 (Hartree/Particle)

Thermal correction to Energy= 0.052232

Thermal correction to Enthalpy= 0.053176

Thermal correction to Gibbs Free Energy= 0.007519

Sum of electronic and zero-point Energies= -1495.747617

Sum of electronic and thermal Energies= -1495.739069

Sum of electronic and thermal Enthalpies= -1495.738125

Sum of electronic and thermal Free Energies= -1495.783782

Transition State

C	-0.67195100	0.93578800	0.44021500
H	-1.06511000	1.05103300	-1.06415900
Cl	0.87692800	0.48378700	0.92952000
Cl	-1.43935900	0.92855900	-2.43449500
Cl	-1.71371600	1.26466200	1.73818400
O	-1.46070400	-1.35634400	-0.18499300
H	-1.44770200	-1.08545900	-1.11251200
H	-2.39279300	-1.44649200	0.02063900

Zero-point correction= 0.037405 (Hartree/Particle)

Thermal correction to Energy= 0.045341

Thermal correction to Enthalpy= 0.046285

Thermal correction to Gibbs Free Energy= 0.002779

Sum of electronic and zero-point Energies= -1495.667862

Sum of electronic and thermal Energies= -1495.659926

Sum of electronic and thermal Enthalpies= -1495.658982

Sum of electronic and thermal Free Energies= -1495.702488

Product (1)

C	0.15858600	-0.05750900	0.48155900
H	0.52465800	0.64537100	-0.25548900
Cl	1.51338200	-0.43457100	1.59179400
Cl	-1.26605200	-0.05976700	-3.07546200
Cl	-1.15063600	0.75496800	1.39707700
O	-0.28808200	-1.16827800	-0.16733500
H	-1.01463600	-0.69816500	-1.98368600
H	-0.64646500	-1.79887000	0.46483600

Zero-point correction= 0.043090 (Hartree/Particle)
Thermal correction to Energy= 0.050684
Thermal correction to Enthalpy= 0.051628
Thermal correction to Gibbs Free Energy= 0.007398
Sum of electronic and zero-point Energies= -1495.759790
Sum of electronic and thermal Energies= -1495.752196
Sum of electronic and thermal Enthalpies= -1495.751252
Sum of electronic and thermal Free Energies= -1495.795482

IRC

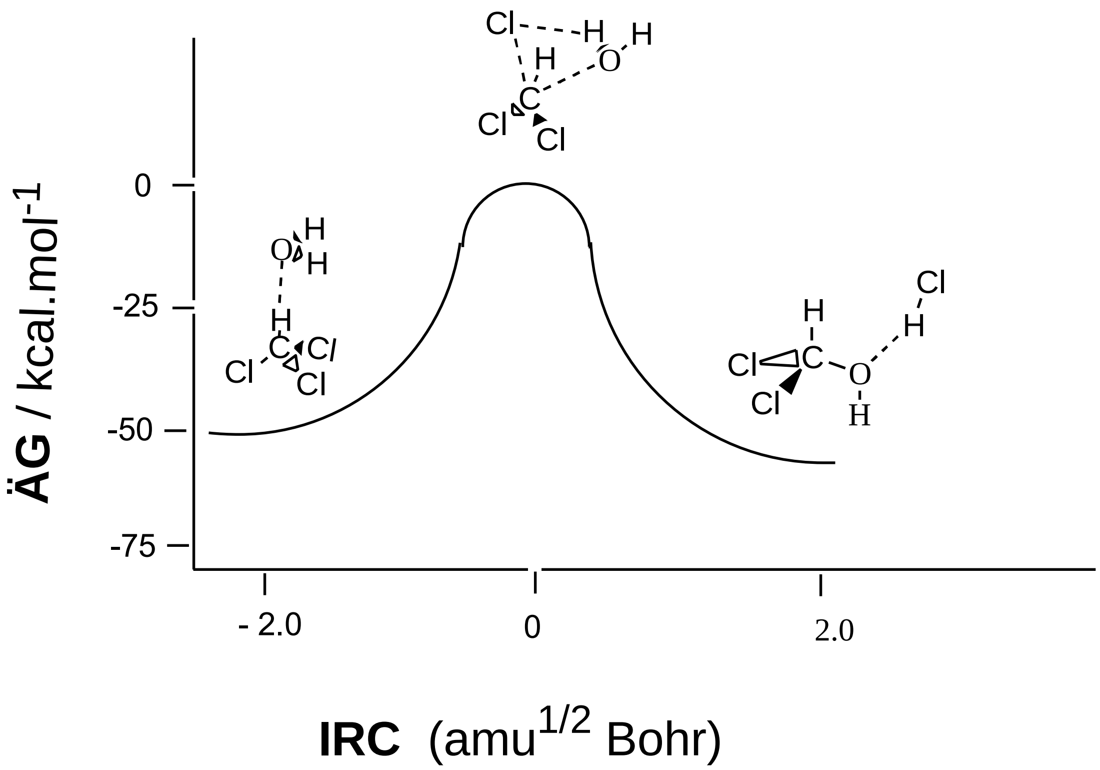

Figure-2

Intermediate (I-3)

C	0.57806600	0.52061500	-0.03261200
H	0.33728500	-0.39411100	-0.61469600
Cl	1.43199800	0.06256700	1.44945400
Cl	1.53103000	1.64537300	-0.99775700
Cl	-1.00731300	1.27438000	0.39464300
O	-1.05832000	-2.70416400	1.07473100
H	-0.57396300	-2.56080900	0.20168100
H	-0.43759500	-2.49318800	1.77002000
O	-0.47496100	-1.83530800	-1.23828000
H	-0.04571800	-2.25370400	-1.97848000
Na	-2.30440500	-1.31855300	-0.28959500

Zero-point correction= 0.058172 (Hartree/Particle)
Thermal correction to Energy= 0.069482
Thermal correction to Enthalpy= 0.070426
Thermal correction to Gibbs Free Energy= 0.018987
Sum of electronic and zero-point Energies= -1733.921391
Sum of electronic and thermal Energies= -1733.910081
Sum of electronic and thermal Enthalpies= -1733.909137
Sum of electronic and thermal Free Energies= -1733.960576

Transition State

C	-1.00774400	0.20624300	-0.49574200
H	-1.22441100	-0.33395700	-1.40979400
Cl	0.57335600	0.39335400	0.01178200
Cl	-1.13504600	1.93953200	-2.48768400
Cl	-2.23442600	0.91240300	0.42333600
O	-1.34882100	-1.90301200	0.05157000
H	-1.72700600	-1.99632400	-0.91634000
H	-2.09375600	-1.98478000	0.64920600
O	-2.07472800	-1.74459300	-2.33170400
H	-1.66299500	-2.47959300	-2.78413400
Na	-2.48439600	0.01428700	-3.53630700

Zero-point correction= 0.056594 (Hartree/Particle)
Thermal correction to Energy= 0.067496
Thermal correction to Enthalpy= 0.068441
Thermal correction to Gibbs Free Energy= 0.018254
Sum of electronic and zero-point Energies= -1733.865715
Sum of electronic and thermal Energies= -1733.854813
Sum of electronic and thermal Enthalpies= -1733.853869
Sum of electronic and thermal Free Energies= -1733.904056

Product (1)

C	0.40572800	-0.37673500	0.97224900
H	0.67379800	-0.11750600	-0.05307300
Cl	1.56921600	0.34028600	2.10766800
Cl	0.47221400	0.23778700	-2.41315600
Cl	-1.23117400	0.42251600	1.26299300
O	0.31014200	-1.72032300	1.10587100
H	-0.86340900	-2.50532000	-0.63689900
H	0.19233300	-1.95360200	2.03192300
O	-1.20889800	-2.21326400	-1.48378100
H	-0.43436700	-1.82720400	-1.93308400
Na	-1.90459500	0.02328600	-1.58488000

Zero-point correction= 0.061439 (Hartree/Particle)
Thermal correction to Energy= 0.072795
Thermal correction to Enthalpy= 0.073739
Thermal correction to Gibbs Free Energy= 0.021941
Sum of electronic and zero-point Energies= -1733.976319
Sum of electronic and thermal Energies= -1733.964963
Sum of electronic and thermal Enthalpies= -1733.964019
Sum of electronic and thermal Free Energies= -1734.015817

IRC

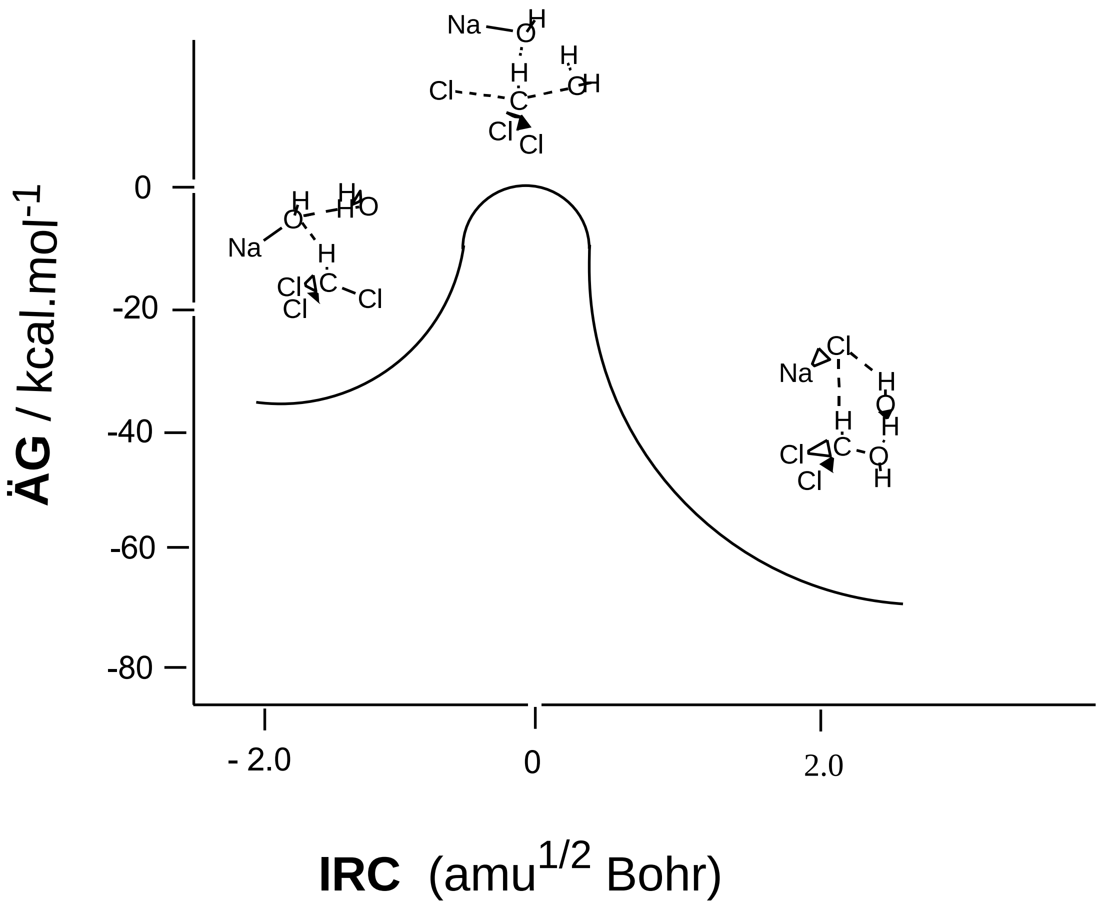
ÄG / kcal.mol-1
0
-20
-40
-60
-80
- 2.0
0
2.0
IRC (amu1/2 Bohr)
Na
Cl

Figure-3

Intermediate (I-5)

C	-0.37192800	0.67195700	-0.17081700
H	0.29777200	0.72834800	-1.07871900
Cl	-0.00136700	-0.91897500	0.61416500
Cl	-0.00263900	1.98202000	0.95969000
Cl	-2.07528500	0.70576400	-0.64832500
O	1.41868400	0.37792900	-2.24557800
H	1.52974900	1.04333400	-2.91683900
Na	1.93442600	-1.42930900	-1.48173800
O	-2.49556200	0.30123700	2.86688300
H	-2.85257600	0.28284800	1.97776900
H	-1.77601800	0.93150700	2.80899400

Zero-point correction= 0.055690 (Hartree/Particle)
Thermal correction to Energy= 0.068757
Thermal correction to Enthalpy= 0.069701
Thermal correction to Gibbs Free Energy= 0.013561
Sum of electronic and zero-point Energies= -1733.892431
Sum of electronic and thermal Energies= -1733.879364
Sum of electronic and thermal Enthalpies= -1733.878420
Sum of electronic and thermal Free Energies= -1733.934560

Transition State

C	-0.61568400	0.36014600	1.82795100
H	0.29072200	0.92150900	1.84963600
Cl	-0.68261400	-1.20522000	2.59851900
Cl	-1.15041100	1.52395300	3.68442500
Cl	-1.99495500	0.90860400	0.90502300
O	0.23619800	-0.49726000	0.21525800
H	0.52058000	0.23228100	-0.33640700
Na	-1.39634500	-1.83519300	-0.05993800
O	-3.88959800	-0.38439500	3.38623800
H	-3.78491300	-0.88409400	4.19581200
H	-3.24183000	0.32680600	3.48054900

Zero-point correction= 0.056785 (Hartree/Particle)
Thermal correction to Energy= 0.068593
Thermal correction to Enthalpy= 0.069537
Thermal correction to Gibbs Free Energy= 0.017428
Sum of electronic and zero-point Energies= -1733.841185
Sum of electronic and thermal Energies= -1733.829377
Sum of electronic and thermal Enthalpies= -1733.828433
Sum of electronic and thermal Free Energies= -1733.880542

Product (2)

C	-0.71293000	-1.45889700	-2.27627900
H	-1.08878200	-1.74903800	-1.28961900
Cl	-0.79447900	-0.50904400	0.99224600
Cl	0.66812100	3.34285300	0.60561300
Cl	-1.05906800	-2.64959900	-3.50882300
O	-0.12882600	-0.45109700	-2.51337200
H	-0.15658200	2.51045000	1.84291500
Na	0.31577500	1.14212000	-0.75598500
O	-0.66356700	1.86619600	2.56453300
H	-0.78547900	0.96293800	2.06117300
H	-0.06022400	1.70915700	3.29775700

Zero-point correction= 0.055979 (Hartree/Particle)
Thermal correction to Energy= 0.067841
Thermal correction to Enthalpy= 0.068785
Thermal correction to Gibbs Free Energy= 0.013271
Sum of electronic and zero-point Energies= -1734.005492
Sum of electronic and thermal Energies= -1733.993631
Sum of electronic and thermal Enthalpies= -1733.992686
Sum of electronic and thermal Free Energies= -1734.048201

IRC

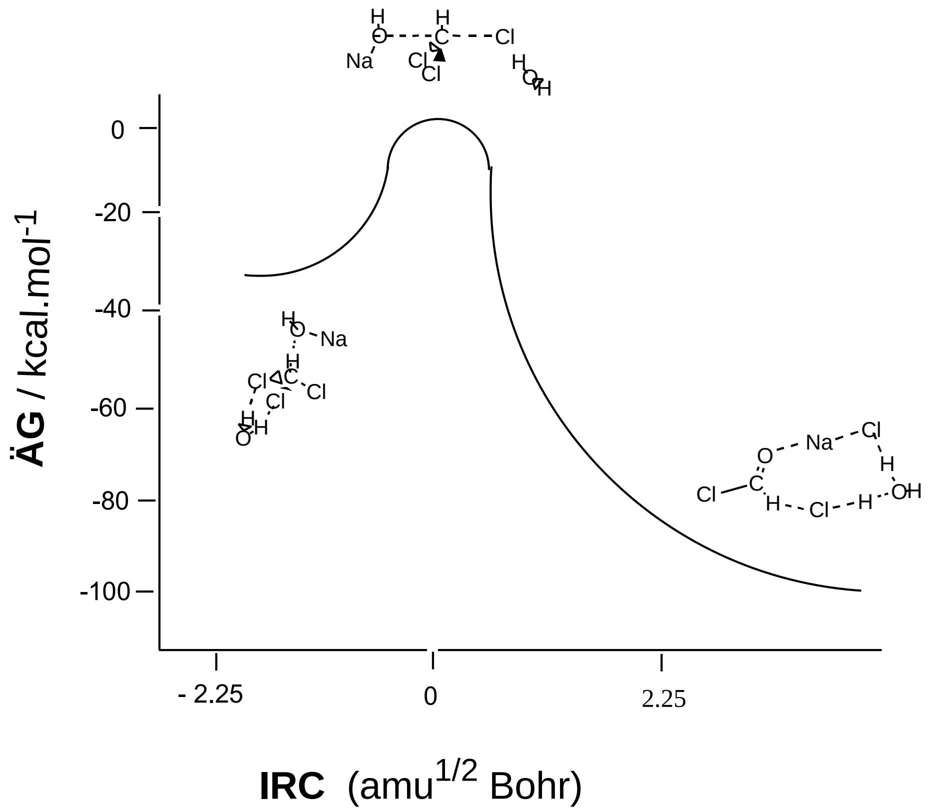

Figure-4

Intermediate I-7

C	-0.43741900	0.16036700	0.70611900
H	-0.82336900	-0.36873100	-0.19104700
Cl	1.23625500	-0.46399400	0.97545600
Cl	-0.37253500	1.89888400	0.37634900
Cl	-1.43559700	-0.18568600	2.11657400
O	-0.92237900	-1.21064900	-1.74227600
H	-1.78882500	-1.53107900	-1.97427700
Na	1.17743100	-1.52866800	-1.80291800
O	0.63204800	0.42422100	-2.91064000
H	0.60415600	1.32535400	-2.59358300
H	-0.20764500	-0.02410500	-2.57839700

Zero-point correction= 0.058018 (Hartree/Particle)
Thermal correction to Energy= 0.069490
Thermal correction to Enthalpy= 0.070435
Thermal correction to Gibbs Free Energy= 0.018165
Sum of electronic and zero-point Energies= -1733.921539
Sum of electronic and thermal Energies= -1733.910067
Sum of electronic and thermal Enthalpies= -1733.909122
Sum of electronic and thermal Free Energies= -1733.961392

Transition State 1

C	-0.21525900	0.10551700	0.57910600
H	-0.69853600	-0.69573000	-0.64699000
Cl	1.47613300	-0.53653100	1.08148000
Cl	0.02162200	1.87452400	0.24534700
Cl	-1.25453600	-0.04659000	2.02040300
O	-0.81367300	-1.26423400	-1.58208700
H	-1.64584800	-1.73153000	-1.55434300
Na	1.42944600	-1.31544300	-1.54936900
O	0.83296400	0.58237500	-2.76154300
H	0.82739900	1.36140600	-2.19765800
H	-0.06098800	0.21851000	-2.66104400

Zero-point correction= 0.055213 (Hartree/Particle)
Thermal correction to Energy= 0.066251
Thermal correction to Enthalpy= 0.067195
Thermal correction to Gibbs Free Energy= 0.016586
Sum of electronic and zero-point Energies= -1733.910940
Sum of electronic and thermal Energies= -1733.899902
Sum of electronic and thermal Enthalpies= -1733.898958
Sum of electronic and thermal Free Energies= -1733.949567

IRC

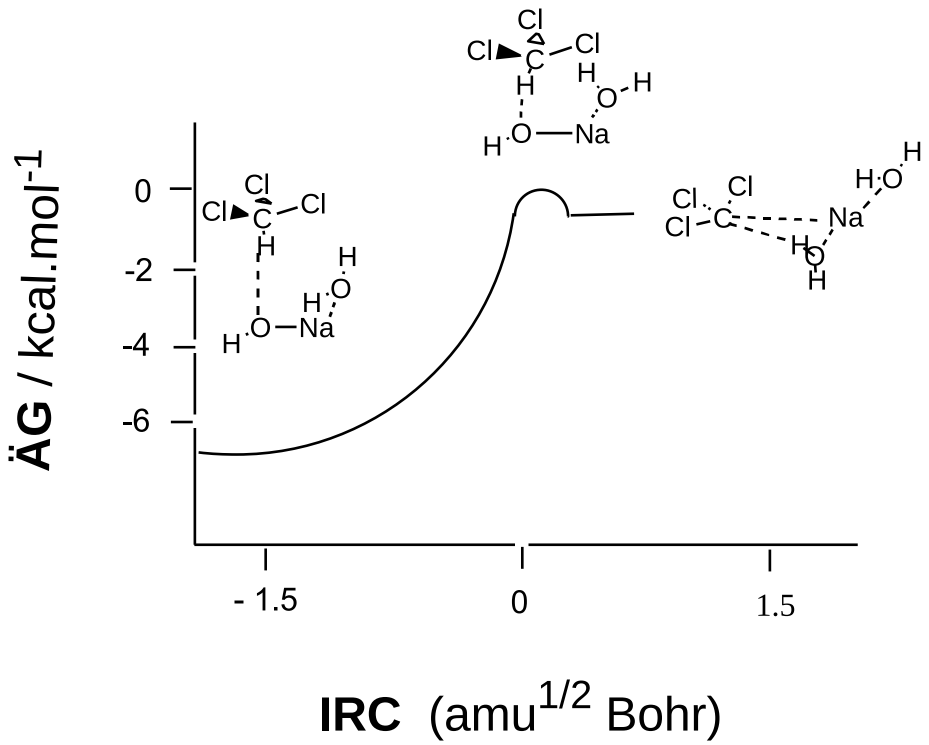

Intermediate I-8

C	-0.47457500	-0.10886800	0.77465900
H	-0.97787100	-0.76585400	-0.68325500
Cl	0.93670100	-1.17785900	1.39192300
Cl	0.32309600	1.53528200	0.43831900
Cl	-1.58413200	0.11572300	2.14453500
O	-0.95982500	-1.13082100	-1.66148400
H	-1.56600800	-1.86765400	-1.71139800
Na	1.31540300	-0.56757900	-1.33441800
O	0.47660200	0.54223200	-3.20550800
H	0.29116000	1.48092500	-3.19117400
H	-0.34694400	0.10228800	-2.93117600

Zero-point correction= 0.056395 (Hartree/Particle)
Thermal correction to Energy= 0.068521
Thermal correction to Enthalpy= 0.069465
Thermal correction to Gibbs Free Energy= 0.016298
Sum of electronic and zero-point Energies= -1733.910279
Sum of electronic and thermal Energies= -1733.898153
Sum of electronic and thermal Enthalpies= -1733.897209
Sum of electronic and thermal Free Energies= -1733.950376

Transition State 2

C	0.75295300	-0.35983300	1.04068000
H	-0.23331100	-0.94063000	-1.32859300
Cl	2.24284700	-1.14945400	0.68206400
Cl	-0.31496100	0.74699600	-2.75993900
Cl	0.00115500	-1.10807100	2.41292500
O	-0.44735000	-1.37413100	-0.47474000
H	-1.34214400	-1.05774900	-0.29526700
Na	-0.14134300	1.55435400	-0.31026000
O	-2.41730300	0.79302100	-0.61536400
H	-3.34961800	1.00338500	-0.64452400
H	-2.09429400	0.76350100	-1.54205300

Zero-point correction= 0.057422 (Hartree/Particle)
Thermal correction to Energy= 0.068991
Thermal correction to Enthalpy= 0.069935
Thermal correction to Gibbs Free Energy= 0.018007
Sum of electronic and zero-point Energies= -1733.891848
Sum of electronic and thermal Energies= -1733.880279
Sum of electronic and thermal Enthalpies= -1733.879335
Sum of electronic and thermal Free Energies= -1733.931263

IRC

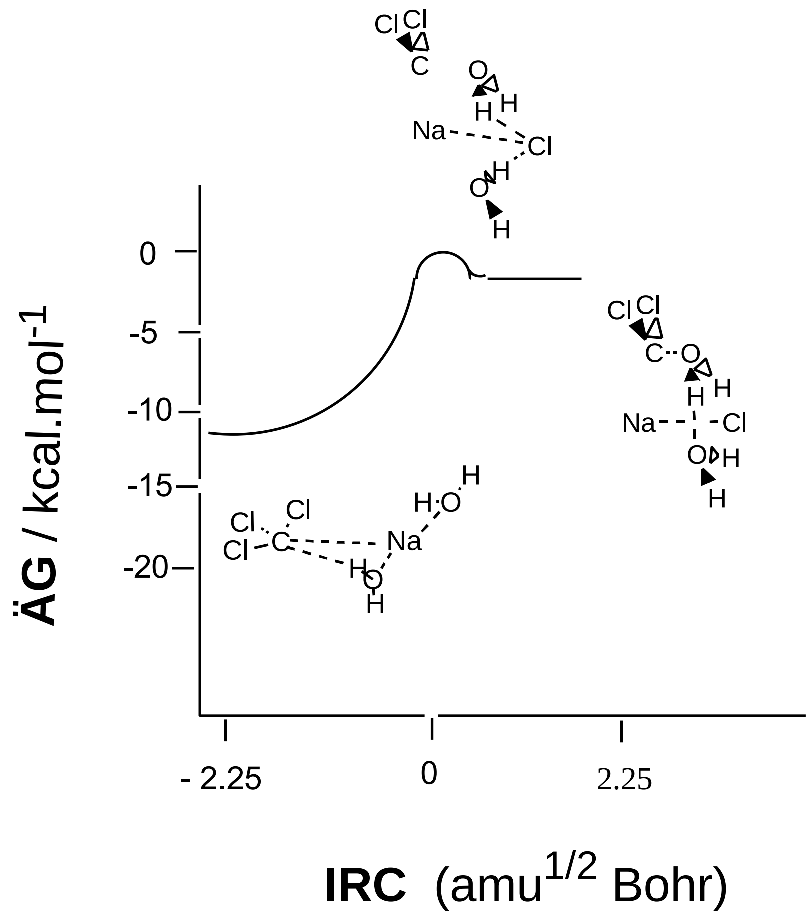

Intermediate I-9

C	0.40543400	-0.22585200	0.74068900
H	-0.24455600	-0.60300000	-1.21954900
Cl	1.99269200	-1.02389400	0.56213400
Cl	-0.34426900	0.80469400	-2.62758900
Cl	-0.35006500	-0.80353300	2.27014100
O	-0.46840600	-1.04110500	-0.33264500
H	-1.37500600	-0.69628500	-0.17571500
Na	-0.07049800	1.96353100	-0.26889100
O	-2.45023700	0.82037400	-0.49987700
H	-3.40542000	0.87492600	-0.47905800
H	-2.16494600	0.86294200	-1.43497700

Zero-point correction= 0.059213 (Hartree/Particle)

Thermal correction to Energy= 0.070477

Thermal correction to Enthalpy= 0.071421

Thermal correction to Gibbs Free Energy= 0.020435

Sum of electronic and zero-point Energies= -1733.895110

Sum of electronic and thermal Energies= -1733.883846

Sum of electronic and thermal Enthalpies= -1733.882902

Sum of electronic and thermal Free Energies= -1733.933888

Transition State 3

C	-0.24165600	-0.53101500	0.35134900
H	-0.71158000	-0.32628600	-0.88147500
Cl	0.89484300	-1.81776700	0.73913100
Cl	-1.45388300	1.77444600	-2.06260500
Cl	-0.90968600	0.18149700	1.86535500
O	-1.37051800	-1.18841800	-0.41819200
H	-2.24788800	-0.69486400	-0.27803500
Na	0.25645600	1.94349100	-0.23031100
O	-3.50504100	0.25871100	-0.38725000
H	-4.23053600	-0.09000600	-0.90679100
H	-3.06017800	0.92083300	-0.95953300

Zero-point correction= 0.054467 (Hartree/Particle)
Thermal correction to Energy= 0.065431
Thermal correction to Enthalpy= 0.066375
Thermal correction to Gibbs Free Energy= 0.015667
Sum of electronic and zero-point Energies= -1733.856679
Sum of electronic and thermal Energies= -1733.845715
Sum of electronic and thermal Enthalpies= -1733.844771
Sum of electronic and thermal Free Energies= -1733.895479

IRC

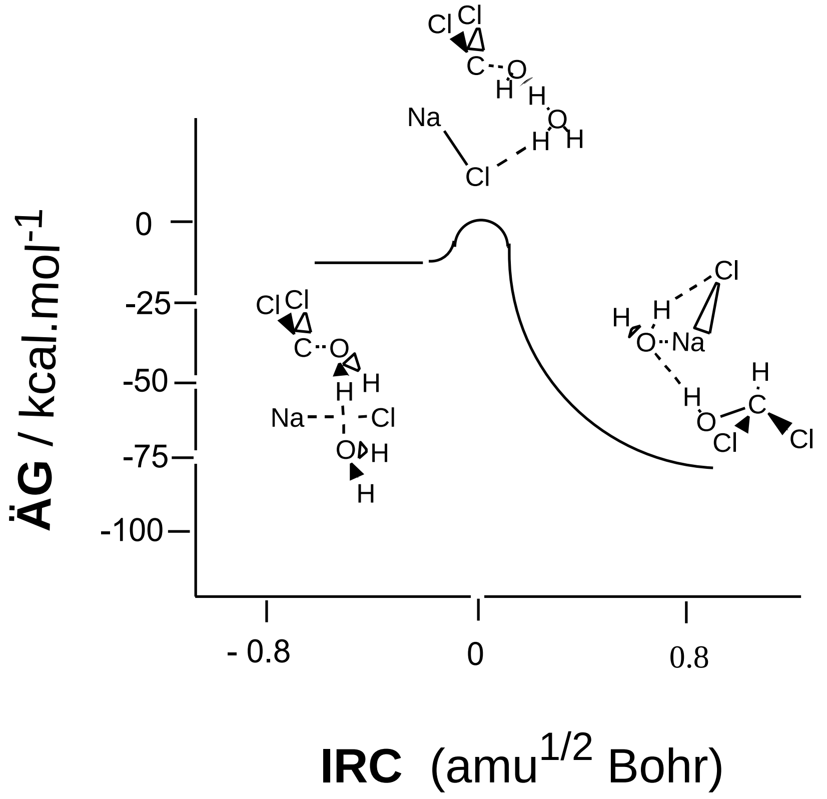

Product (1)

C	0.44221000	-1.09040000	0.17508600
H	0.58269300	-0.51888700	-0.74084000
Cl	1.69514100	-2.31517300	0.32351600
Cl	-0.35879500	1.45890100	-2.32795600
Cl	0.79348200	0.23091800	1.51066900
O	-0.76029700	-1.62152400	0.35741600
H	-1.44032300	-1.00865600	0.00047000
Na	-0.71966400	2.07400700	0.11149200
O	-2.40243900	0.33225400	-0.56252100
H	-3.32319900	0.18737800	-0.78053900
H	-1.92868500	0.58936600	-1.39824300

Zero-point correction= 0.061559 (Hartree/Particle)
Thermal correction to Energy= 0.072429
Thermal correction to Enthalpy= 0.073373
Thermal correction to Gibbs Free Energy= 0.022789
Sum of electronic and zero-point Energies= -1733.981494
Sum of electronic and thermal Energies= -1733.970624
Sum of electronic and thermal Enthalpies= -1733.969680
Sum of electronic and thermal Free Energies= -1734.020264

Figure-5

Intermediate (I-12)

C	-0.30198900	-0.90618500	-1.01040200
Cl	0.66341100	-2.30189600	-1.30290300
Cl	-1.43608100	1.26649000	1.39645800
Cl	-1.97653400	-1.15547200	-1.33910100
O	0.72581000	-1.35231100	2.29701900
H	0.07738300	-2.00515100	2.03280500
H	0.24475200	-0.52147000	2.27032200
C	0.19697600	0.25028400	-0.55353900
C	1.61153700	0.42054100	-0.25814300
C	2.13226800	1.64622500	-0.13292100
C	1.30917700	2.83248800	-0.28704100
C	-0.01703200	2.74099500	-0.37619000
C	-0.70197700	1.42463600	-0.30890800
H	2.22136600	-0.46046300	-0.13852600
H	3.18527100	1.76560500	0.07927500
H	1.78907400	3.80122900	-0.31012000
H	-0.64511700	3.61699100	-0.45176100
H	-1.57937700	1.39480500	-0.94415700

Zero-point correction= 0.124512 (Hartree/Particle)

Thermal correction to Energy= 0.137819

Thermal correction to Enthalpy= 0.138763

Thermal correction to Gibbs Free Energy= 0.082613

Sum of electronic and zero-point Energies= -1726.671354

Sum of electronic and thermal Energies= -1726.658047

Sum of electronic and thermal Enthalpies= -1726.657103

Sum of electronic and thermal Free Energies= -1726.713252

Transition State

C	-1.51072600	-0.83958600	-0.79809400
Cl	-0.49723800	-2.15793500	-1.18043000
Cl	-2.88105300	1.23342000	1.95422800
Cl	-3.16583700	-1.15744700	-1.02098500
O	-1.39893300	-1.19711700	1.38380100
H	-1.99974300	-1.89214700	1.66440100
H	-1.85035900	-0.34747100	1.69408700
C	-1.01582800	0.47896000	-0.75724600
C	0.37831000	0.71667800	-0.66701100
C	0.84775500	1.99553100	-0.58948100
C	-0.05058400	3.07337000	-0.59544000
C	-1.40798800	2.86332700	-0.65320400
C	-1.90887300	1.56912900	-0.70160300
H	1.06323100	-0.11658500	-0.64855400
H	1.91005400	2.17821700	-0.51948400
H	0.33319900	4.08318600	-0.54190600
H	-2.09664900	3.69358300	-0.62243400
H	-2.96989000	1.40773000	-0.76971300

Zero-point correction= 0.124749 (Hartree/Particle)
Thermal correction to Energy= 0.136093
Thermal correction to Enthalpy= 0.137037
Thermal correction to Gibbs Free Energy= 0.086078
Sum of electronic and zero-point Energies= -1726.645581
Sum of electronic and thermal Energies= -1726.634238
Sum of electronic and thermal Enthalpies= -1726.633294
Sum of electronic and thermal Free Energies= -1726.684253

Product (3)

C	-0.35908400	-0.90054700	-0.22358300
Cl	-0.11683800	-1.85573700	-1.75102000
Cl	0.01065200	-0.22060500	3.84340900
Cl	-2.15335300	-0.80214700	0.04761000
O	0.21605100	-1.56897600	0.81967700
H	-0.12730400	-2.46669900	0.84975400
H	0.01514500	-0.67896700	2.63796400
C	0.24194700	0.47395500	-0.36915400
C	1.37944400	0.81126900	0.34960200
C	1.94038300	2.07131600	0.20183400
C	1.37577600	2.98880500	-0.66578700
C	0.24274900	2.64596500	-1.39137300
C	-0.32348200	1.39472600	-1.24410300
H	1.83030200	0.09816700	1.02227300
H	2.82193300	2.33045800	0.77116900
H	1.81470900	3.97050000	-0.77868300
H	-0.20286100	3.35791300	-2.07189700
H	-1.20787700	1.13129900	-1.80722300

Zero-point correction= 0.124050 (Hartree/Particle)
Thermal correction to Energy= 0.136169
Thermal correction to Enthalpy= 0.137113
Thermal correction to Gibbs Free Energy= 0.082325
Sum of electronic and zero-point Energies= -1726.723741
Sum of electronic and thermal Energies= -1726.711621
Sum of electronic and thermal Enthalpies= -1726.710677
Sum of electronic and thermal Free Energies= -1726.765465

IRC

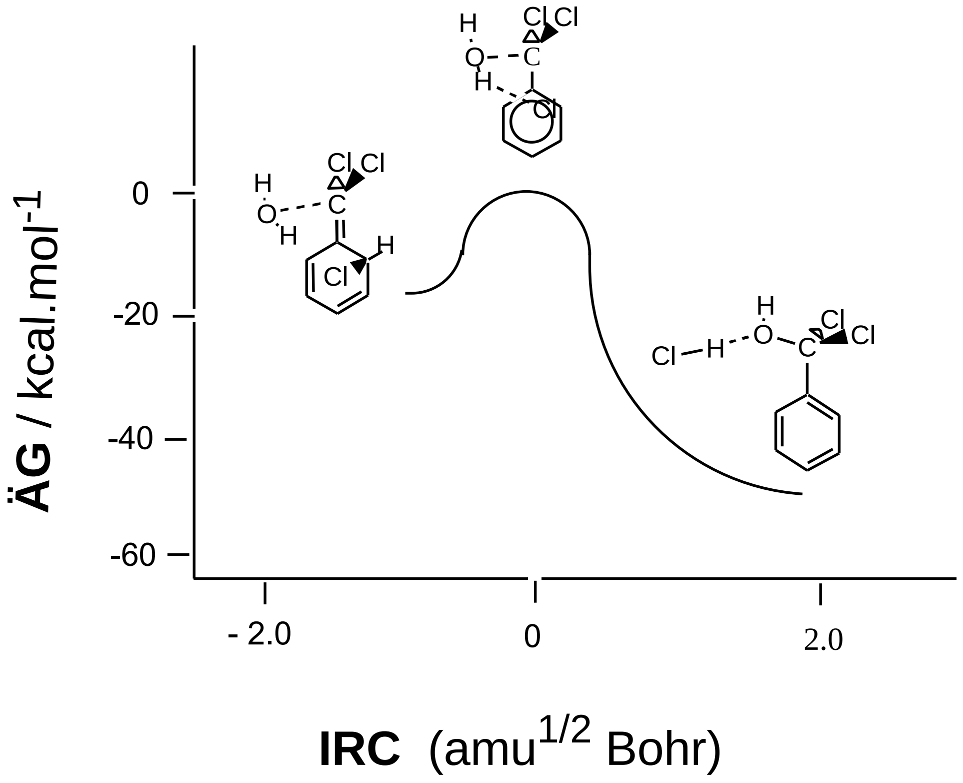

Figure-6

Intermediate (I-13)

C	0.53369600	-0.29429400	-0.90082600
Cl	0.77599000	-1.68221800	0.25907300
Cl	1.29121400	-0.78165800	-2.43597600
Cl	-1.22730600	-0.15011300	-1.19865400
O	-1.97291700	0.56576000	2.32047200
H	-2.30392100	1.26350400	2.87665600
C	1.10009200	0.96912000	-0.29757600
C	2.27643000	1.53177100	-0.77854200
C	2.79345200	2.66872600	-0.17925000
C	2.14383300	3.24012800	0.90191600
C	0.97510900	2.66877100	1.38176100
C	0.44084000	1.53564900	0.79298000
H	2.79330400	1.09387700	-1.61820600
H	3.70584500	3.10404100	-0.56270300
H	2.54852900	4.12786000	1.36941500
H	0.46029600	3.09992500	2.22942300
H	-0.48566100	1.12690300	1.22440100
Na	-2.06222600	-1.35193600	1.67971900
O	-2.15138700	-3.30870700	0.37794200

H	-2.86430200	-3.69931300	-0.12710300
H	-1.40198800	-3.25355900	-0.22060400

Zero-point correction= 0.137297 (Hartree/Particle)
Thermal correction to Energy= 0.154929
Thermal correction to Enthalpy= 0.155873
Thermal correction to Gibbs Free Energy= 0.088937
Sum of electronic and zero-point Energies= -1964.860101
Sum of electronic and thermal Energies= -1964.842469
Sum of electronic and thermal Enthalpies= -1964.841525
Sum of electronic and thermal Free Energies= -1964.908461

Transition State

C	-0.18624900	0.16891000	0.10928300
Cl	-1.10067300	0.90543700	-1.20572200
Cl	0.26715700	2.27484300	1.04927000
Cl	-1.13017800	-0.30177100	1.51289500
O	-0.53606500	-1.69037400	-0.66328800
H	0.21778000	-1.86724200	-1.22362100
C	1.27017500	-0.09392600	0.00010400
C	1.96914200	0.30261900	-1.13382800
C	3.31977100	0.02187800	-1.24712000
C	3.98464300	-0.65153300	-0.23555100
C	3.28453700	-1.04357600	0.89534400
C	1.93450700	-0.77223400	1.01512000
H	1.46580000	0.85040600	-1.91605100
H	3.85493000	0.34228200	-2.13051400
H	5.04095300	-0.86440100	-0.32437000
H	3.79267800	-1.56570400	1.69448000
H	1.39734600	-1.07935200	1.89907400
Na	-2.64364500	-1.75505600	-0.59797500
O	-4.15813900	-0.01871700	-0.29478300
H	-4.86091000	0.18305200	0.32269600
H	-3.55409900	0.73062100	-0.25613100

Zero-point correction= 0.137680 (Hartree/Particle)
Thermal correction to Energy= 0.154355
Thermal correction to Enthalpy= 0.155299
Thermal correction to Gibbs Free Energy= 0.091484
Sum of electronic and zero-point Energies= -1964.811228
Sum of electronic and thermal Energies= -1964.794553
Sum of electronic and thermal Enthalpies= -1964.793609
Sum of electronic and thermal Free Energies= -1964.857424

Product (3)

C	-0.77334200	0.61571100	1.87085500
Cl	-0.10917500	-1.03788800	2.32467200

Cl	1.44334000	-2.20648700	-1.53389700
Cl	-2.18606700	0.30426600	0.76740100
O	-1.31093000	1.19483600	2.97356800
H	-0.59451200	1.53814900	3.51735700
C	0.26885600	1.43977200	1.15315700
C	1.22005300	0.86677800	0.31859000
C	2.11422800	1.67506300	-0.36178900
C	2.07221400	3.05178500	-0.20731700
C	1.12027900	3.62486300	0.62145700
C	0.21300000	2.82421900	1.29438200
H	1.28416500	-0.20351100	0.16915500
H	2.83929300	1.21153200	-1.01557800
H	2.77615900	3.67872500	-0.73713500
H	1.07450100	4.69860100	0.73883800
H	-0.54991200	3.27918600	1.91048600
Na	-0.82538400	-2.11559200	-0.38637400
O	-1.05411000	-3.93681300	-1.79984200
H	-1.45269600	-4.24096900	-2.61320700
H	-0.13627200	-3.67312400	-2.01868300

Zero-point correction= 0.141090 (Hartree/Particle)
Thermal correction to Energy= 0.157694
Thermal correction to Enthalpy= 0.158639
Thermal correction to Gibbs Free Energy= 0.091120
Sum of electronic and zero-point Energies= -1964.937453
Sum of electronic and thermal Energies= -1964.920848
Sum of electronic and thermal Enthalpies= -1964.919904
Sum of electronic and thermal Free Energies= -1964.987423

IRC

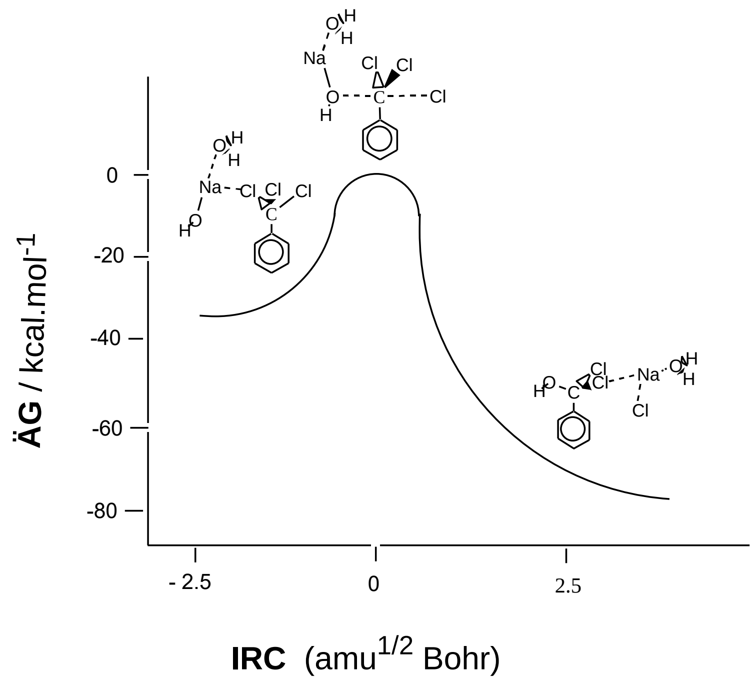

Figure-7

Intermediate (I-14)

C	-0.49546000	0.81588900	0.22785500
H	-0.76940500	1.85801600	0.13354700
Cl	0.78569100	0.74425900	1.52198200
Cl	0.30094500	0.35735600	-1.34595300
O	-0.39869400	-2.38862700	0.64497600
H	0.30016800	-2.21926600	1.28119300
H	0.04536800	-2.42086600	-0.20567300
O	-1.55692800	0.06751200	0.50833700
H	-1.28402300	-0.87281600	0.58969300

Zero-point correction= 0.059573 (Hartree/Particle)
Thermal correction to Energy= 0.067094
Thermal correction to Enthalpy= 0.068039
Thermal correction to Gibbs Free Energy= 0.026894
Sum of electronic and zero-point Energies= -1111.366393
Sum of electronic and thermal Energies= -1111.358871
Sum of electronic and thermal Enthalpies= -1111.357927
Sum of electronic and thermal Free Energies= -1111.399072

Transition State

C	0.68816900	-0.32078300	-0.70809800
H	0.72204300	0.66897900	-0.26840700
Cl	2.70758700	-0.87507400	0.33882500
Cl	1.21193000	-0.29768100	-2.39413300
O	1.16056900	-3.19210500	-0.14179300
H	0.93042900	-3.65555400	0.66944300
H	1.95306000	-2.57053400	0.07461200
O	-0.10948800	-1.18517400	-0.33183000
H	0.41624800	-2.36496200	-0.32060400

Zero-point correction= 0.054643 (Hartree/Particle)
Thermal correction to Energy= 0.060894
Thermal correction to Enthalpy= 0.061838
Thermal correction to Gibbs Free Energy= 0.023460
Sum of electronic and zero-point Energies= -1111.339843
Sum of electronic and thermal Energies= -1111.333592
Sum of electronic and thermal Enthalpies= -1111.332647
Sum of electronic and thermal Free Energies= -1111.371025

Product (2)

C	-0.87027500	1.03772500	-0.04992600
H	-0.57922600	2.00111900	0.37568400

Cl	1.93235100	-0.43009000	1.40376600
Cl	-0.19859600	0.83436900	-1.66155400
O	-0.29484400	-2.37865900	0.59238400
H	-0.63294300	-2.83986300	1.36119500
H	1.04422900	-1.33891500	1.05509500
O	-1.55101600	0.22626000	0.47519900
H	-0.96703200	-1.72349400	0.37225800

Zero-point correction= 0.053359 (Hartree/Particle)
Thermal correction to Energy= 0.062447
Thermal correction to Enthalpy= 0.063391
Thermal correction to Gibbs Free Energy= 0.016965
Sum of electronic and zero-point Energies= -1111.371646
Sum of electronic and thermal Energies= -1111.362558
Sum of electronic and thermal Enthalpies= -1111.361614
Sum of electronic and thermal Free Energies= -1111.408040

IRC

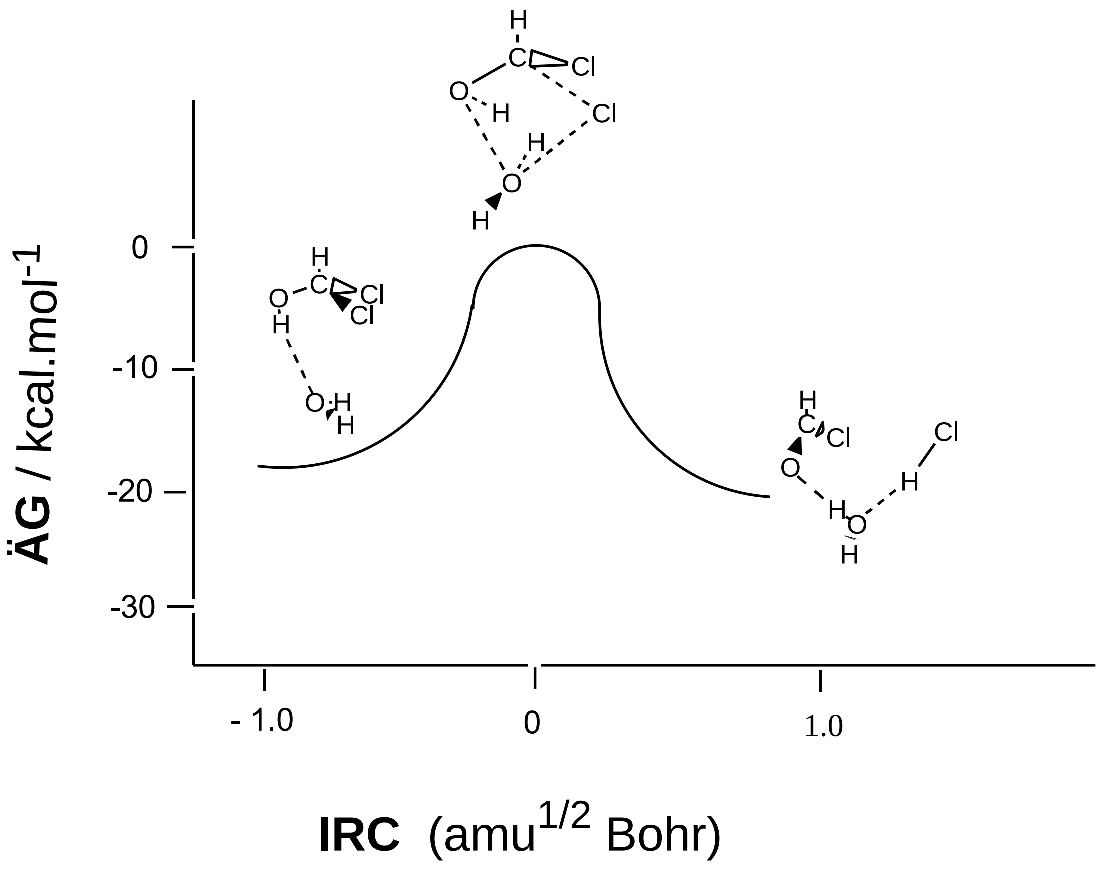

Figure-8

Intermediate (I-15)

C	-0.20691700	1.01423600	-0.87066400
H	-0.20083400	1.97608800	-1.36615600
Cl	-0.48031800	1.39903900	0.90905500
Cl	1.49326700	0.36805800	-1.01769000
O	0.78758900	-1.60441700	1.73478000
H	0.59675400	-0.66280300	1.80605500
H	1.62120100	-1.63900800	1.26159300
O	-1.12277100	0.21376900	-1.37513900
H	-1.14231500	-0.67214600	-0.92013700
O	-1.16607600	-2.10576300	-0.12558400
H	-1.98915000	-2.29832000	0.32335300
H	-0.48237800	-2.06196000	0.57110900

Zero-point correction= 0.084780 (Hartree/Particle)
Thermal correction to Energy= 0.094972
Thermal correction to Enthalpy= 0.095916
Thermal correction to Gibbs Free Energy= 0.048431
Sum of electronic and zero-point Energies= -1187.794537
Sum of electronic and thermal Energies= -1187.784345
Sum of electronic and thermal Enthalpies= -1187.783401
Sum of electronic and thermal Free Energies= -1187.830886

Transition State

C	-0.41683900	0.53310700	0.28276000
H	-0.62608600	1.58551200	0.12838300
Cl	-1.12361200	0.66198400	2.43077200
Cl	1.37398900	0.32939600	0.52456900
O	0.51335500	-1.96559900	2.82384700
H	0.27485600	-1.03992500	3.02043800
H	1.35189100	-1.90580300	2.35933000
O	-1.03448500	-0.37392900	-0.25843500
H	-1.26842500	-1.50474800	0.53681800
O	-1.52207400	-2.16199400	1.33227500
H	-2.07175200	-1.57250900	1.88092800
H	-0.65945400	-2.23537400	1.89240700

Zero-point correction= 0.082722 (Hartree/Particle)
Thermal correction to Energy= 0.091516
Thermal correction to Enthalpy= 0.092460
Thermal correction to Gibbs Free Energy= 0.048108
Sum of electronic and zero-point Energies= -1187.774518
Sum of electronic and thermal Energies= -1187.765724
Sum of electronic and thermal Enthalpies= -1187.764780
Sum of electronic and thermal Free Energies= -1187.809132

Product (2)

C	0.31596900	0.86862000	-1.26827300
H	-0.42829800	1.64020500	-1.47348100
Cl	-1.82909700	0.92028300	1.20371700
Cl	1.70778200	1.56007700	-0.41431900
O	0.92042400	-1.62334200	1.19802500
H	0.80579800	-0.82329800	1.71647500
H	1.42878300	-1.34653000	0.43226500
O	0.25481800	-0.27314800	-1.55686400
H	-1.61270200	-1.85517300	-0.67809700
O	-1.67207700	-1.86868200	0.27929900
H	-1.87140700	-0.35828200	0.77595600
H	-0.75133300	-1.97706300	0.58659600

Zero-point correction= 0.079094 (Hartree/Particle)
Thermal correction to Energy= 0.090665
Thermal correction to Enthalpy= 0.091609
Thermal correction to Gibbs Free Energy= 0.039578
Sum of electronic and zero-point Energies= -1187.798094
Sum of electronic and thermal Energies= -1187.786523
Sum of electronic and thermal Enthalpies= -1187.785579
Sum of electronic and thermal Free Energies= -1187.837610

IRC

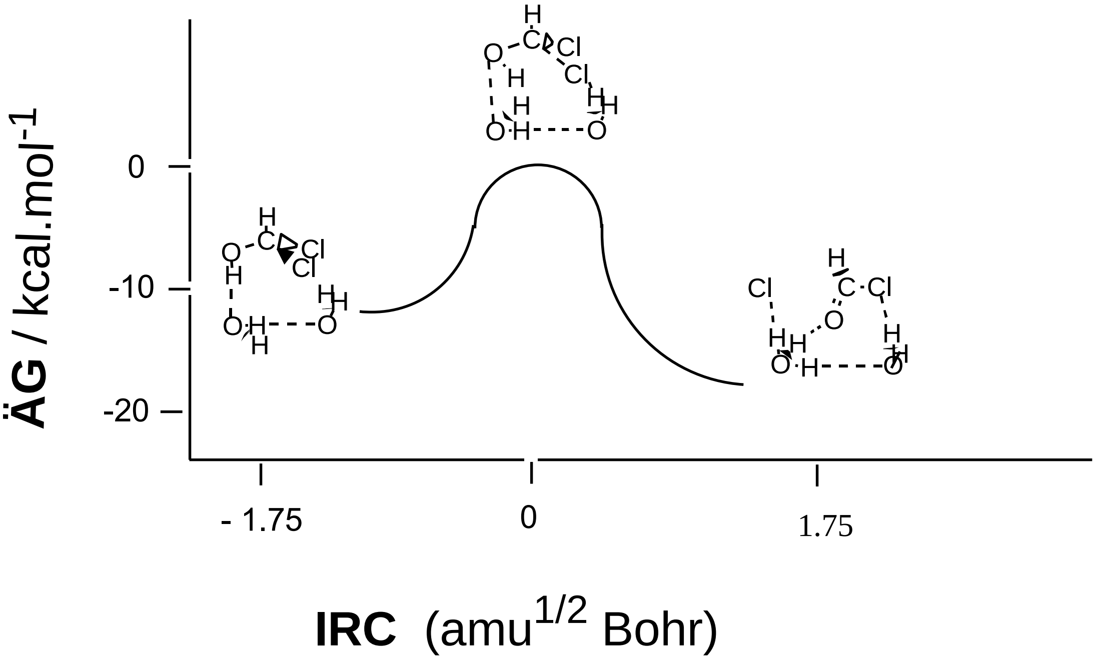

Figure-9

Intermediate (I-16)

C	0.72207700	-0.64547000	-0.51023200
H	0.19292000	0.06669100	-1.15255900
Cl	0.09697500	-0.45849900	1.17215900
O	1.55793900	-1.41946500	-0.78666300
O	-1.31767500	1.64509000	-1.18213300
H	-1.04692900	2.56051400	-1.25423800
H	-1.59752200	1.53916600	-0.27124600

Zero-point correction= 0.043134 (Hartree/Particle)

Thermal correction to Energy= 0.050228

Thermal correction to Enthalpy= 0.051172

Thermal correction to Gibbs Free Energy= 0.011247

Sum of electronic and zero-point Energies= -650.544755

Sum of electronic and thermal Energies= -650.537661

Sum of electronic and thermal Enthalpies= -650.536717

Sum of electronic and thermal Free Energies= -650.576642

Transition State

C	0.77170500	-0.30737300	1.24515000
H	-0.09297000	0.67980600	0.90516800
Cl	-0.24424400	0.15592800	3.37354500
O	1.55989500	-1.11623000	1.24660400
O	-1.00076100	1.48603000	1.03162900
H	-0.68457500	2.39169300	0.96805100
H	-1.06174600	1.25434800	2.02064000

Zero-point correction= 0.039605 (Hartree/Particle)

Thermal correction to Energy= 0.044770

Thermal correction to Enthalpy= 0.045714

Thermal correction to Gibbs Free Energy= 0.010930

Sum of electronic and zero-point Energies= -650.510377

Sum of electronic and thermal Energies= -650.505212

Sum of electronic and thermal Enthalpies= -650.504268

Sum of electronic and thermal Free Energies= -650.539052

Product (4)

C	1.12084500	-0.81157000	-1.31233100
H	-0.46971000	0.83996300	-1.52326700
Cl	-0.88573100	0.37817400	1.75138900
O	1.82223200	-1.63516000	-1.01698900
O	-1.14078400	1.42307100	-1.15450400
H	-0.78625300	2.30931500	-1.23707000

H -1.10025200 0.87458500 0.55799700

Zero-point correction= 0.038693 (Hartree/Particle)
Thermal correction to Energy= 0.046765
Thermal correction to Enthalpy= 0.047709
Thermal correction to Gibbs Free Energy= 0.004392
Sum of electronic and zero-point Energies= -650.553589
Sum of electronic and thermal Energies= -650.545518
Sum of electronic and thermal Enthalpies= -650.544574
Sum of electronic and thermal Free Energies= -650.587890

IRC

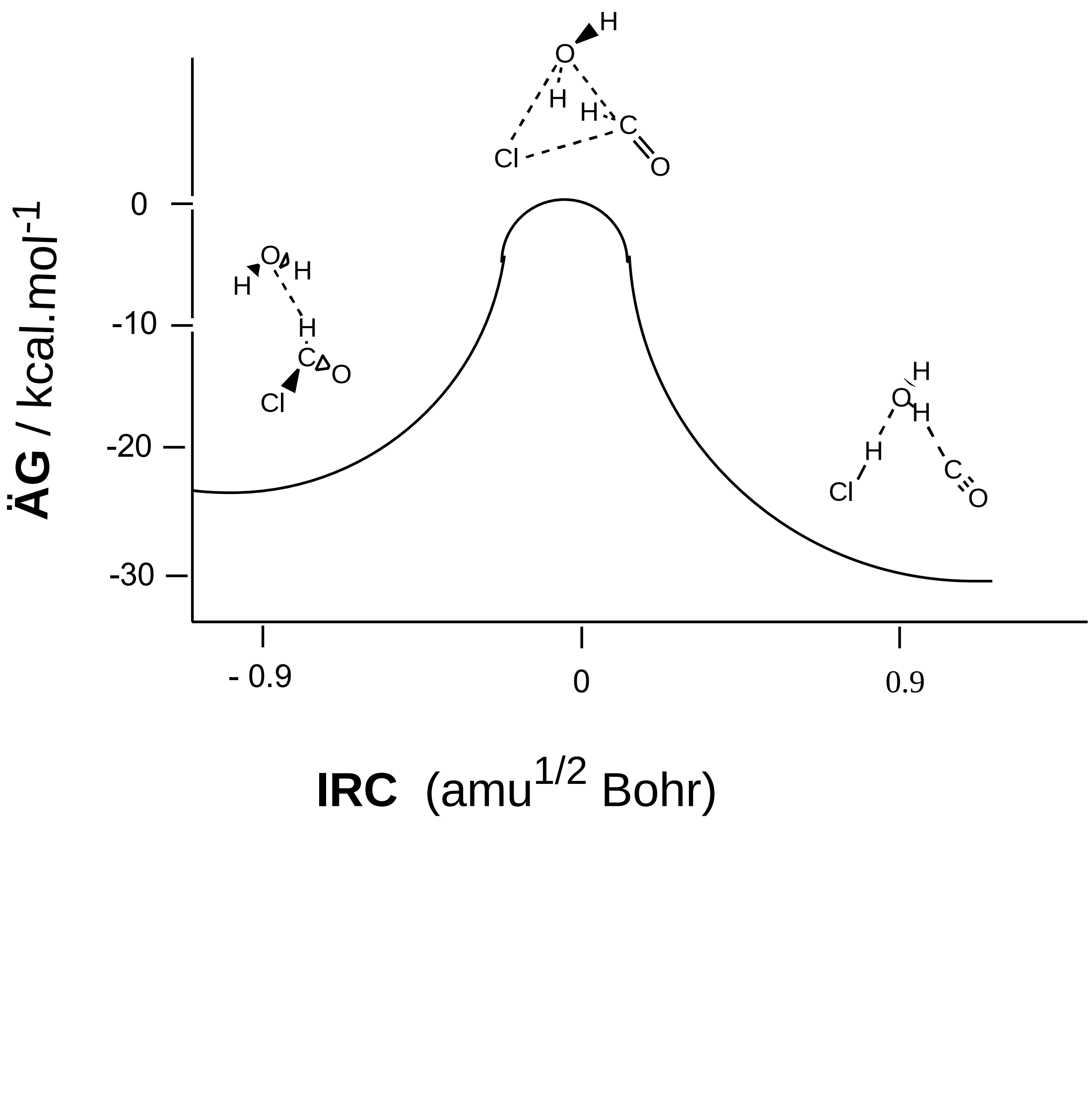

Figure-10

Intermediate (I-18)

C	-0.55186300	-1.03019800	-0.01879200
H	-0.87141500	0.01501000	-0.12278600
Cl	-1.87540300	-2.08415300	0.49341700
O	0.54031000	-1.44505200	-0.21545300
O	-0.41259200	1.98586200	-0.65644800
H	0.51193500	1.76782100	-0.85958700
O	2.10023000	0.84592300	-1.07704200
H	2.39407700	0.72222600	-1.97940900
H	1.79970100	-0.02386500	-0.78360300
H	-0.37236300	2.69466200	-0.01573000

Zero-point correction= 0.069442 (Hartree/Particle)
Thermal correction to Energy= 0.078832
Thermal correction to Enthalpy= 0.079776
Thermal correction to Gibbs Free Energy= 0.034320
Sum of electronic and zero-point Energies= -726.971043
Sum of electronic and thermal Energies= -726.961653
Sum of electronic and thermal Enthalpies= -726.960709
Sum of electronic and thermal Free Energies= -727.006165

Transition State

C	0.75683800	0.30548300	1.09814100
H	-0.22487900	0.14587800	0.65648800
Cl	0.35020900	0.19538800	2.99684400
O	1.74777000	-0.34028700	0.71571400
O	0.99480400	1.84365500	0.98103300
H	2.22003300	1.89560100	0.95207900
O	3.33430000	1.48204400	0.90743600
H	3.75965600	1.69297100	0.07319200
H	2.91534300	0.51686000	0.83266800
H	0.63307000	2.23209300	1.78530000

Zero-point correction= 0.068651 (Hartree/Particle)
Thermal correction to Energy= 0.074575
Thermal correction to Enthalpy= 0.075519
Thermal correction to Gibbs Free Energy= 0.038510
Sum of electronic and zero-point Energies= -726.932981
Sum of electronic and thermal Energies= -726.927056
Sum of electronic and thermal Enthalpies= -726.926112
Sum of electronic and thermal Free Energies= -726.963122

Product (5)

C	-0.70490000	-0.25238500	-0.46860000
H	-1.67712800	-0.64278200	-0.75717400
Cl	-0.68360400	-0.52198100	1.38811700
O	0.26174000	-0.92827000	-1.08887200
O	-0.57595200	1.11173900	-0.68618600
H	1.60957800	1.44886400	-0.52259500
O	2.33585400	0.83257300	-0.66245700
H	2.75879900	1.11947100	-1.47255400
H	1.11807500	-0.48062100	-0.93186900
H	-1.19106900	1.57272300	-0.11201400

Zero-point correction= 0.073674 (Hartree/Particle)
Thermal correction to Energy= 0.081275
Thermal correction to Enthalpy= 0.082219
Thermal correction to Gibbs Free Energy= 0.041496
Sum of electronic and zero-point Energies= -726.970397
Sum of electronic and thermal Energies= -726.962796
Sum of electronic and thermal Enthalpies= -726.961852
Sum of electronic and thermal Free Energies= -727.002575

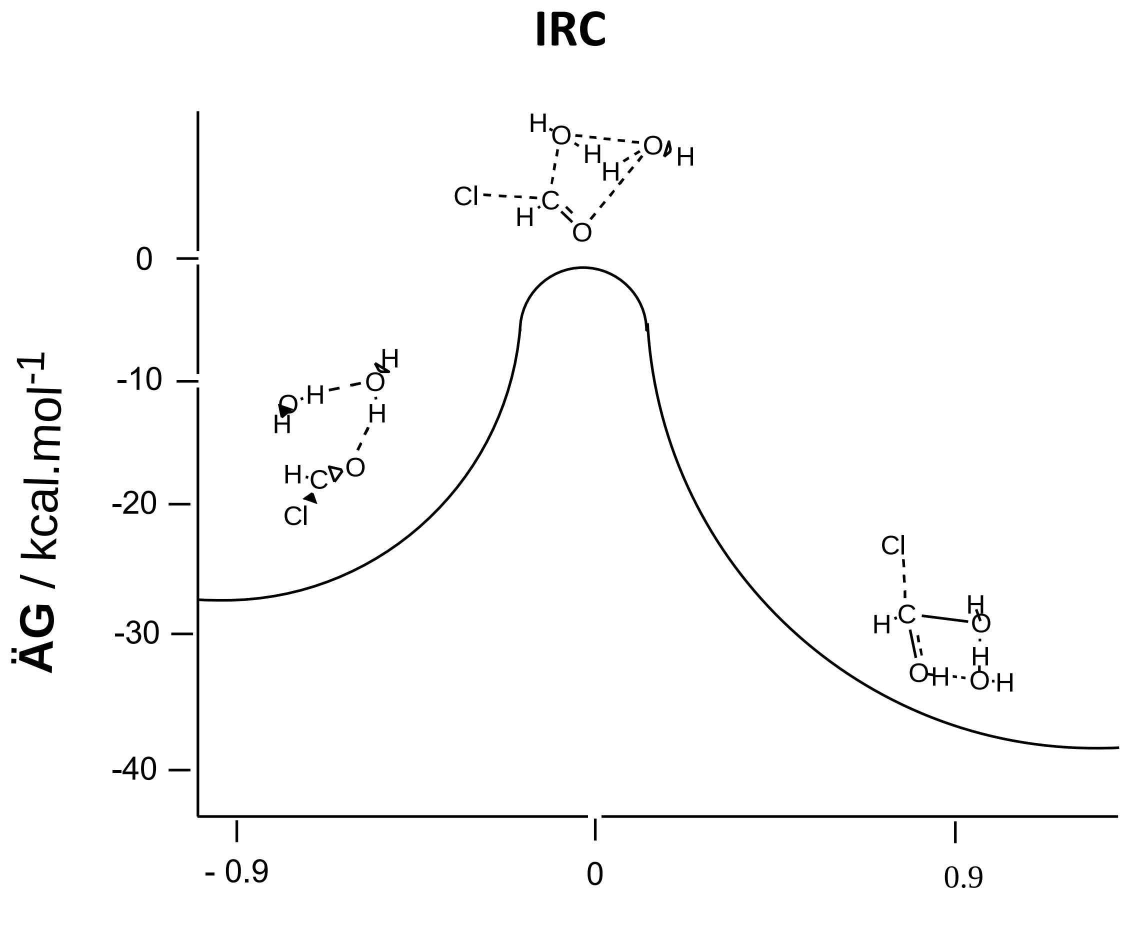

Figure-11

Intermediate (i-19)

C	-0.92193600	-0.84453900	0.30000200
H	-1.39661700	-1.73219100	0.69802000
Cl	0.86390700	-1.00464200	1.07894000
O	-0.78593500	-0.95293600	-1.04175800
O	-0.53519100	2.45039900	-0.52262800
H	-1.09230800	2.75594200	-1.23843500
H	0.28214500	2.13530300	-0.93423300
O	-1.49732300	0.25590500	0.78339000
H	-0.17466900	-0.28103000	-1.37793200
H	-1.16514300	1.06405700	0.33518300
O	1.48088300	0.77209100	-1.40499800
H	1.70328400	0.27979400	-0.59854500
H	2.19223200	0.62394000	-2.02753000

Zero-point correction= 0.099614 (Hartree/Particle)
Thermal correction to Energy= 0.109570
Thermal correction to Enthalpy= 0.110515
Thermal correction to Gibbs Free Energy= 0.064812
Sum of electronic and zero-point Energies= -803.400835
Sum of electronic and thermal Energies= -803.390879
Sum of electronic and thermal Enthalpies= -803.389935
Sum of electronic and thermal Free Energies= -803.435637

Transition State

C	-0.93701700	-0.73561700	0.02219300
H	-1.34566500	-1.61872100	0.50113100
Cl	1.71656600	-0.73607100	1.21440500
O	-0.38497300	-0.90818100	-1.10514500
O	-0.10467600	2.43442100	-0.41187400
H	-0.53763000	2.94135800	-1.09883200
H	0.71661100	2.07338200	-0.79338800
O	-1.20878900	0.36399100	0.57779500
H	0.35783100	-0.22890500	-1.33703700
H	-0.78974700	1.19722400	0.15704200
O	1.62054000	0.58698600	-1.27224600
H	1.85227700	0.15010900	-0.34633200
H	2.33341700	0.37581600	-1.87633700

Zero-point correction= 0.096518 (Hartree/Particle)
Thermal correction to Energy= 0.105039
Thermal correction to Enthalpy= 0.105983
Thermal correction to Gibbs Free Energy= 0.063220
Sum of electronic and zero-point Energies= -803.393858
Sum of electronic and thermal Energies= -803.385336

Sum of electronic and thermal Enthalpies= -803.384392
Sum of electronic and thermal Free Energies= -803.427155

Product (6)

C	-2.13739200	-0.69403600	-0.33238600
H	-2.97306300	-1.38856500	-0.19383400
Cl	3.44496700	-0.30894500	1.02751900
O	-1.07759400	-1.05826800	-0.79330600
O	-0.48504500	2.26060700	-0.25423300
H	-0.63641200	3.00968600	-0.82955700
H	0.27953200	1.77872800	-0.61417800
O	-2.45105800	0.51112100	0.05353700
H	0.43532000	-0.27463000	-1.02491400
H	-1.69960600	1.15526300	-0.07275900
O	1.22610100	0.30115800	-1.10970900
H	2.49939300	-0.07059000	0.16184400
H	1.58710800	0.14422000	-1.98245900

Zero-point correction= 0.095041 (Hartree/Particle)
Thermal correction to Energy= 0.105852
Thermal correction to Enthalpy= 0.106796
Thermal correction to Gibbs Free Energy= 0.056109
Sum of electronic and zero-point Energies= -803.426286
Sum of electronic and thermal Energies= -803.415476
Sum of electronic and thermal Enthalpies= -803.414532
Sum of electronic and thermal Free Energies= -803.465219

IRC

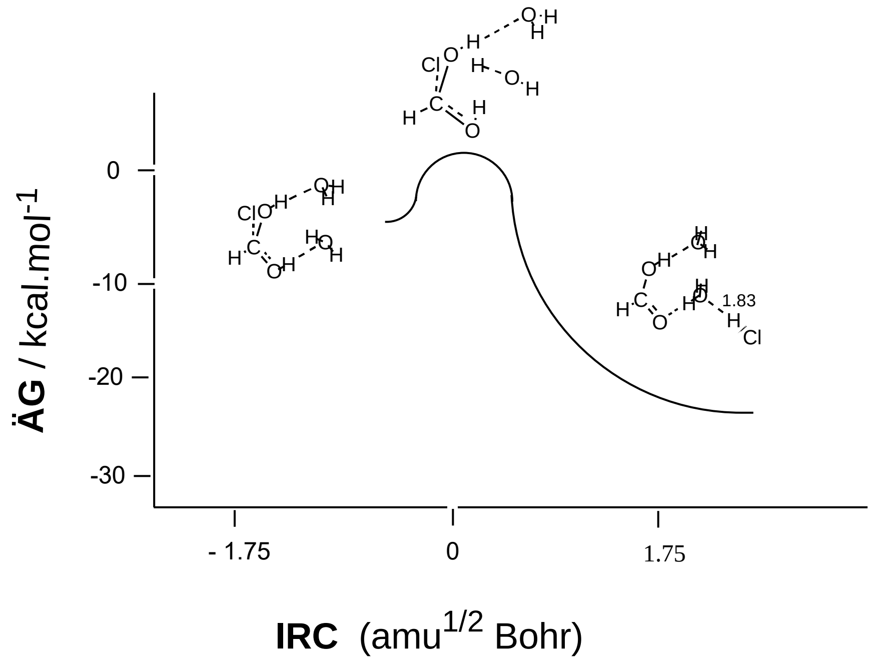

Figure-12

Intermediate (I-20)

C	-0.46505900	-0.17657200	0.18753600
O	0.16559000	0.10142100	1.16335100
Na	2.37718700	0.26404100	0.16747700
O	1.53077200	-0.19864000	-1.63417400
H	0.00432900	-0.29079200	-0.83418500
H	1.63677800	-0.35296700	-2.56736400
Cl	-2.22212200	-0.43135100	0.29829000

Zero-point correction= 0.032026 (Hartree/Particle)
Thermal correction to Energy= 0.039383
Thermal correction to Enthalpy= 0.040327
Thermal correction to Gibbs Free Energy= -0.000174
Sum of electronic and zero-point Energies= -812.275716
Sum of electronic and thermal Energies= -812.268359
Sum of electronic and thermal Enthalpies= -812.267415
Sum of electronic and thermal Free Energies= -812.307915

Transition State

C	-1.05607400	0.54991000	1.50522900
O	-0.41256600	1.01519400	2.39547300
Na	1.59793400	1.67282100	1.27635500
O	0.64417800	0.93023800	-0.35634300
H	-0.72247000	-0.17844900	0.76821800
H	0.55796500	0.91460500	-1.30454100
Cl	-2.73445400	1.04920700	1.23623900

Zero-point correction= 0.032341 (Hartree/Particle)
Thermal correction to Energy= 0.039026
Thermal correction to Enthalpy= 0.039970
Thermal correction to Gibbs Free Energy= 0.000917
Sum of electronic and zero-point Energies= -812.270419
Sum of electronic and thermal Energies= -812.263734
Sum of electronic and thermal Enthalpies= -812.262790
Sum of electronic and thermal Free Energies= -812.301843

Product (6)

C	0.45389400	-1.39947900	-0.06649600
O	0.93451400	-0.66155500	0.77703600
Na	0.79524000	1.59013100	0.81495100
O	-0.23128900	-1.05055800	-1.10690000

H	0.57405000	-2.48673200	-0.00325500
H	-0.34984300	-0.04761200	-1.17791400
Cl	-0.54666700	1.90896400	-1.26168300
Cl	-0.54054000	1.87849300	-1.25479100

Zero-point correction= 0.036304 (Hartree/Particle)
Thermal correction to Energy= 0.042721
Thermal correction to Enthalpy= 0.043666
Thermal correction to Gibbs Free Energy= 0.004947
Sum of electronic and zero-point Energies= -812.365752
Sum of electronic and thermal Energies= -812.359334
Sum of electronic and thermal Enthalpies= -812.358390
Sum of electronic and thermal Free Energies= -812.397109

IRC

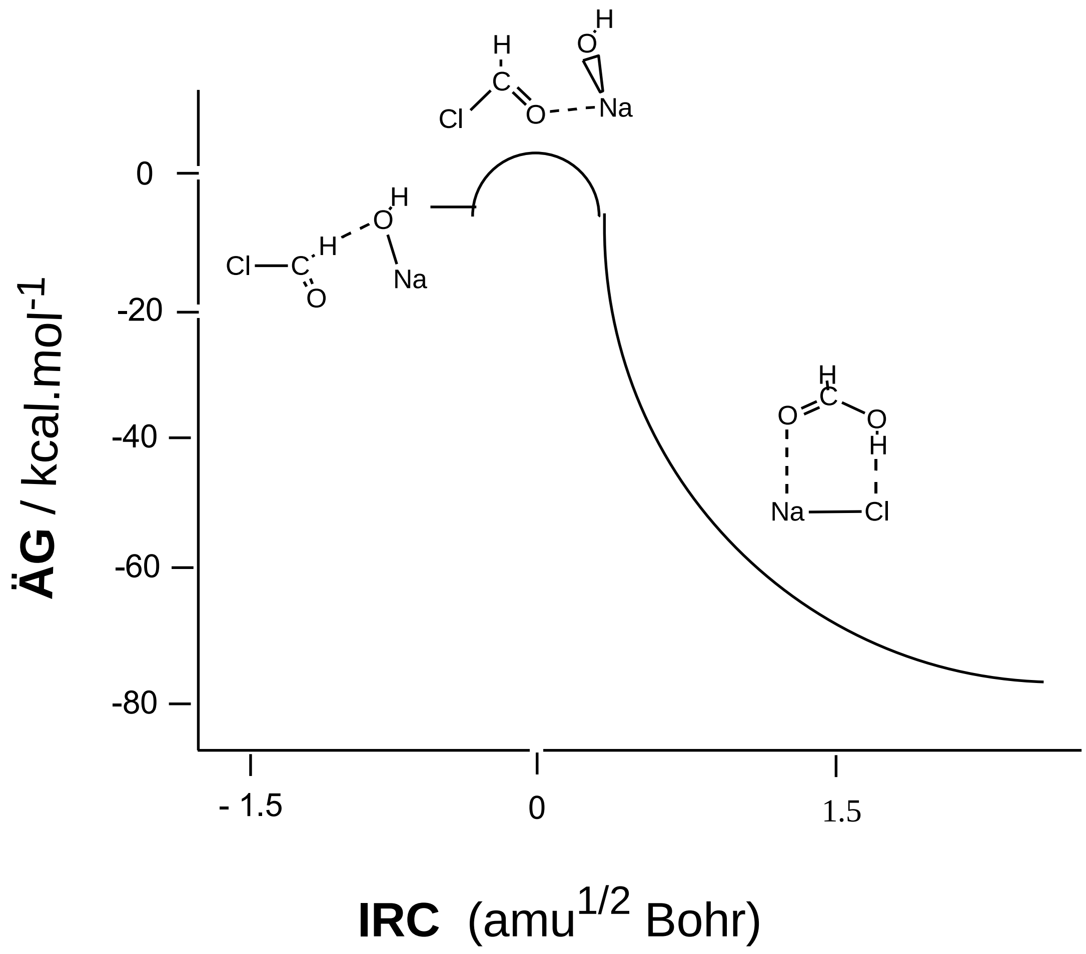

Figure-13

Intermediate (tt-7)

C	0.66286200	-1.04541500	0.43573400
O	0.27027800	0.09691800	-0.07946900
O	1.86025900	-0.84800800	0.92145800
H	-0.64436700	0.05553600	-0.45999900
Na	-0.00543900	2.40262900	-0.43721900
O	1.65782300	-3.42344700	1.67886200
H	2.01390200	-4.12485000	1.13311000
H	0.83389100	-3.15383800	1.24620000
H	2.12913100	-1.70925900	1.30097000
Cl	-2.11332000	1.35129400	-1.25692000

Zero-point correction= 0.061234 (Hartree/Particle)

Thermal correction to Energy= 0.071077

Thermal correction to Enthalpy= 0.072021

Thermal correction to Gibbs Free Energy= 0.023714

Sum of electronic and zero-point Energies= -888.709226

Sum of electronic and thermal Energies= -888.699383

Sum of electronic and thermal Enthalpies= -888.698439

Sum of electronic and thermal Free Energies= -888.746746

Transition State

C	0.31480400	0.24587500	0.37166800
O	-0.16972200	1.33664000	-0.16983200
O	1.46773200	0.37353500	0.86980100
H	-1.08953900	1.23187700	-0.54095000
Na	-0.64486400	3.60891000	-0.59653900
O	0.90958600	-1.84896600	1.34994900
H	1.26458600	-2.52804500	0.77507000
H	0.17320300	-1.13686600	0.72755900
H	1.53261900	-0.74867200	1.23862900
Cl	-2.64924300	2.30724700	-1.34100300

Zero-point correction= 0.055172 (Hartree/Particle)

Thermal correction to Energy= 0.063525

Thermal correction to Enthalpy= 0.064469

Thermal correction to Gibbs Free Energy= 0.019377

Sum of electronic and zero-point Energies= -888.690362

Sum of electronic and thermal Energies= -888.682009

Sum of electronic and thermal Enthalpies= -888.681065

Sum of electronic and thermal Free Energies= -888.726156

Product (6)

C	0.88572500	-0.86649300	0.44775400
O	0.37033700	0.21422500	-0.12566600
O	1.98452500	-0.86758500	0.92637800
H	-0.57230300	0.11337200	-0.45497800
Na	-0.21788600	2.47677700	-0.50623500
O	1.62198300	-3.60806500	1.74173400
H	2.12116600	-4.16877600	1.14905100
H	0.22897900	-1.74638400	0.44566600
H	2.02327400	-2.73614100	1.64764000
Cl	-2.20299000	1.06072600	-1.12606000

Zero-point correction= 0.060020 (Hartree/Particle)
Thermal correction to Energy= 0.070430
Thermal correction to Enthalpy= 0.071374
Thermal correction to Gibbs Free Energy= 0.020789
Sum of electronic and zero-point Energies= -888.765839
Sum of electronic and thermal Energies= -888.755429
Sum of electronic and thermal Enthalpies= -888.754485
Sum of electronic and thermal Free Energies= -888.805069

IRC

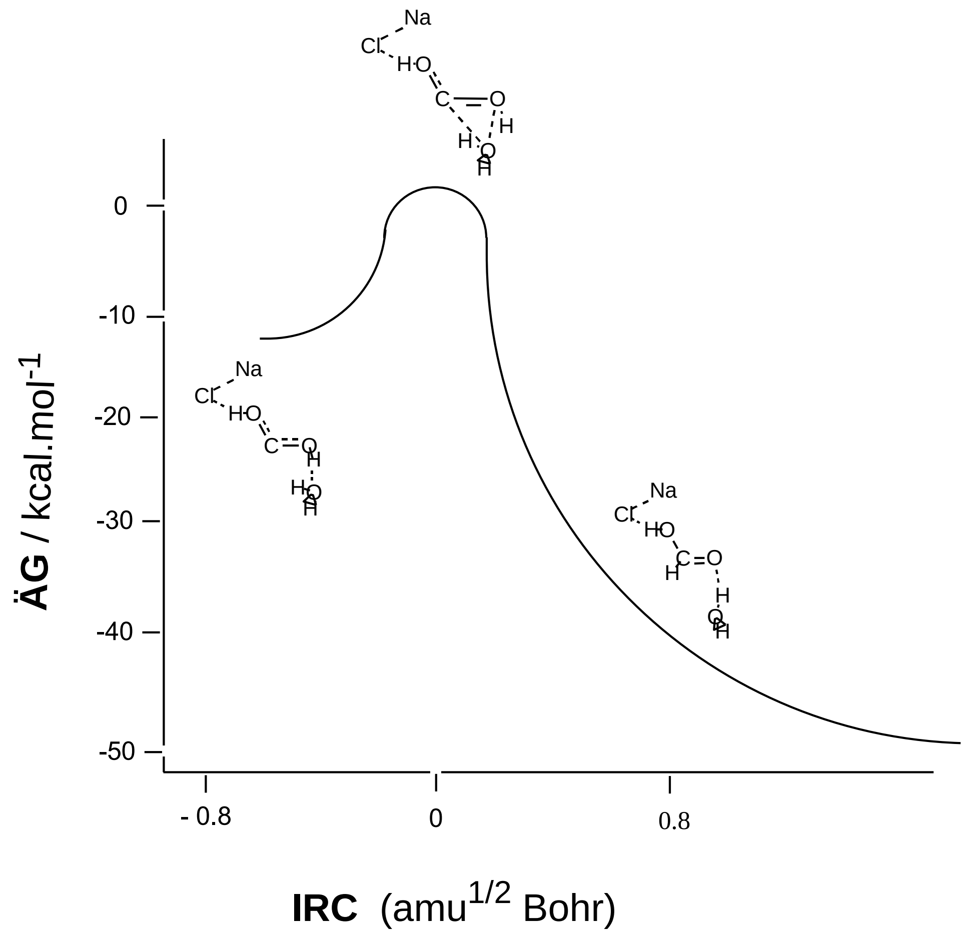

Figure-14

Intermediate (ct-7)

C	1.24449700	0.32650900	0.94145600
O	2.25823600	0.67072400	1.70298600
Na	-0.68404000	-0.16558400	-2.03568700
O	1.34106400	0.68006900	-1.85414000
H	1.41382900	0.59987500	-0.85363200
H	2.14935300	1.07217800	-2.17553500
Cl	-1.98932800	-0.93385100	-0.04563700
O	0.28880700	-0.15793700	1.65468300
H	-0.49816000	-0.42742100	1.08270800
H	2.05234000	0.47930200	2.63450200

Zero-point correction= 0.060025 (Hartree/Particle)
Thermal correction to Energy= 0.069665
Thermal correction to Enthalpy= 0.070609
Thermal correction to Gibbs Free Energy= 0.023247
Sum of electronic and zero-point Energies= -888.737343
Sum of electronic and thermal Energies= -888.727703
Sum of electronic and thermal Enthalpies= -888.726759
Sum of electronic and thermal Free Energies= -888.774120

Transition State

C	0.59592600	-0.59297200	0.37830400
O	1.14600700	-0.09091700	1.37404800
Na	-1.60378900	-0.95799600	-2.29094000
O	0.52720500	-0.36556900	-2.44560300
H	0.73082900	-0.39772100	-1.47000000
H	1.26695700	0.04528100	-2.88713900
Cl	-2.90645300	-1.60185000	-0.22919700
O	-0.31636400	-1.46722200	1.04105600
H	-1.27450300	-1.53920900	0.66357200
H	0.22211600	-0.85119400	1.92981900

Zero-point correction= 0.053236 (Hartree/Particle)
Thermal correction to Energy= 0.062846
Thermal correction to Enthalpy= 0.063790
Thermal correction to Gibbs Free Energy= 0.016300
Sum of electronic and zero-point Energies= -888.680879
Sum of electronic and thermal Energies= -888.671269
Sum of electronic and thermal Enthalpies= -888.670325
Sum of electronic and thermal Free Energies= -888.717815

Product (4)

C	2.34563900	1.28222800	1.66979600
O	2.41755400	2.20053400	2.30898100
Na	1.29462600	-0.59893300	-0.03254200
O	1.19549900	0.15748200	-2.21491900
H	1.62930700	0.66294100	-2.89984100
H	0.49811100	0.73312600	-1.83951800
Cl	-0.58887000	1.27737100	0.00193000
O	-0.32150500	-1.42562400	1.40835100
H	-0.76710000	-0.58928000	1.16241500
H	-1.00665500	-2.08847800	1.47563900

Zero-point correction= 0.056394 (Hartree/Particle)
Thermal correction to Energy= 0.068555
Thermal correction to Enthalpy= 0.069499
Thermal correction to Gibbs Free Energy= 0.015239
Sum of electronic and zero-point Energies= -888.779864
Sum of electronic and thermal Energies= -888.767703
Sum of electronic and thermal Enthalpies= -888.766759
Sum of electronic and thermal Free Energies= -888.821019

IRC

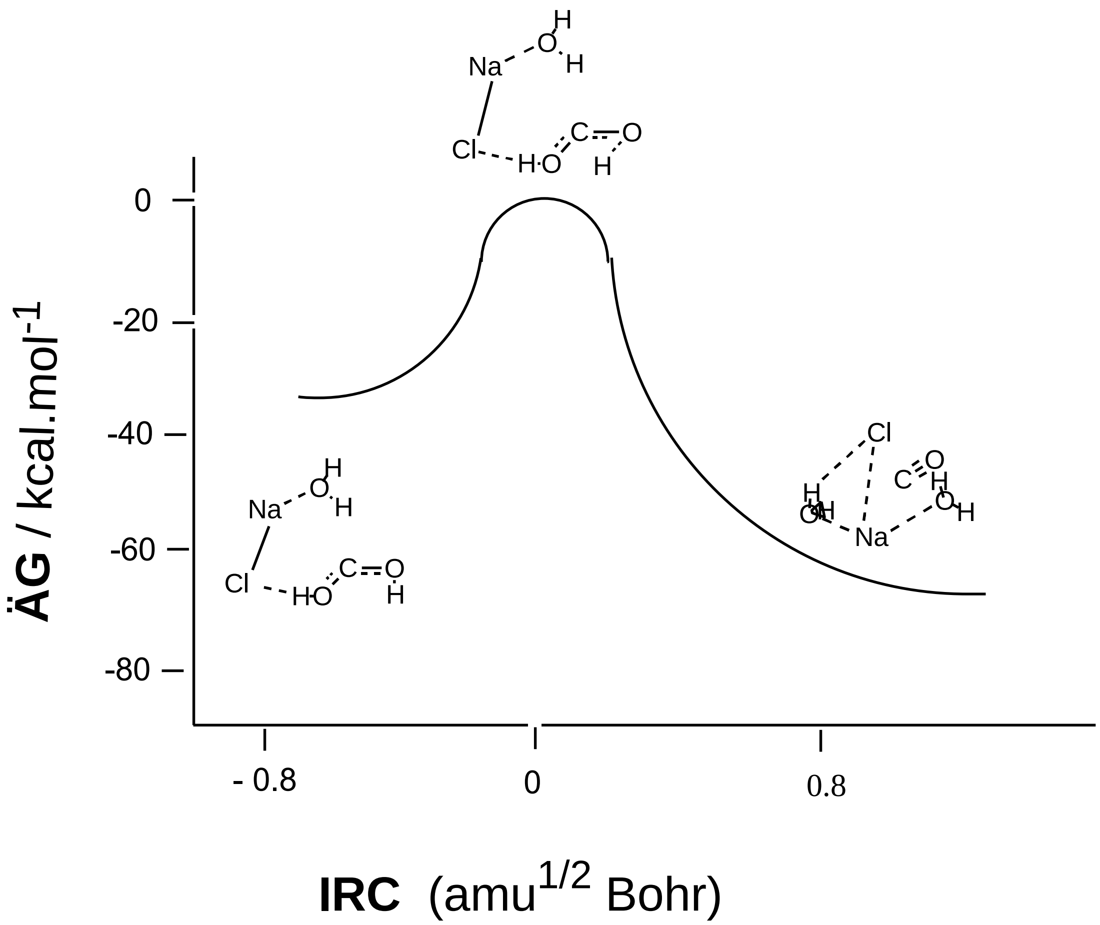

Figure-15

Intermediate (I-21)

C	-1.71177400	-1.50424500	0.45013300
O	0.61950400	-0.46713600	-0.24370500
O	-1.52723600	-2.41182700	1.08818100
H	1.40968800	-0.99799500	-0.25846700
Na	-0.99362400	0.61668000	-1.01978400
O	0.27686100	1.92738200	0.36415900
H	0.27295000	2.16490800	1.28849700
H	0.65621400	0.97415600	0.29304700

Zero-point correction= 0.043047 (Hartree/Particle)
Thermal correction to Energy= 0.051922
Thermal correction to Enthalpy= 0.052866
Thermal correction to Gibbs Free Energy= 0.008810
Sum of electronic and zero-point Energies= -427.889777
Sum of electronic and thermal Energies= -427.880902
Sum of electronic and thermal Enthalpies= -427.879958
Sum of electronic and thermal Free Energies= -427.924014

Transition State

C	-0.42273600	0.44982400	0.30715100
O	0.24683100	0.27943500	-1.01341800
O	-0.73051500	-0.56987600	0.83837300
H	0.29332600	-0.67106800	-1.17238400
Na	0.60201400	2.41767600	-1.53035900
O	-0.31439800	3.04034600	0.27566100
H	-0.65469100	3.57039500	0.99105300
H	-0.46176300	1.89962300	0.48342100

Zero-point correction= 0.041561 (Hartree/Particle)
Thermal correction to Energy= 0.048547
Thermal correction to Enthalpy= 0.049491
Thermal correction to Gibbs Free Energy= 0.010518
Sum of electronic and zero-point Energies= -427.877836
Sum of electronic and thermal Energies= -427.870850
Sum of electronic and thermal Enthalpies= -427.869906
Sum of electronic and thermal Free Energies= -427.908878

Product (6)

C	-0.36902400	-0.97750200	0.64678200
O	0.26285100	-0.99430800	-0.58650500
O	-0.68025300	-1.99549200	1.18106500
H	0.35654500	-1.92120300	-0.83674800

Na	0.64316300	1.30061100	-1.15259600
O	-0.22818000	1.87798000	0.58003100
H	-0.49897000	2.58445500	1.15722000
H	-0.48937800	0.08742300	0.95924400

Zero-point correction= 0.046936 (Hartree/Particle)
Thermal correction to Energy= 0.054282
Thermal correction to Enthalpy= 0.055226
Thermal correction to Gibbs Free Energy= 0.015500
Sum of electronic and zero-point Energies= -427.884376
Sum of electronic and thermal Energies= -427.877030
Sum of electronic and thermal Enthalpies= -427.876086
Sum of electronic and thermal Free Energies= -427.915812

IRC

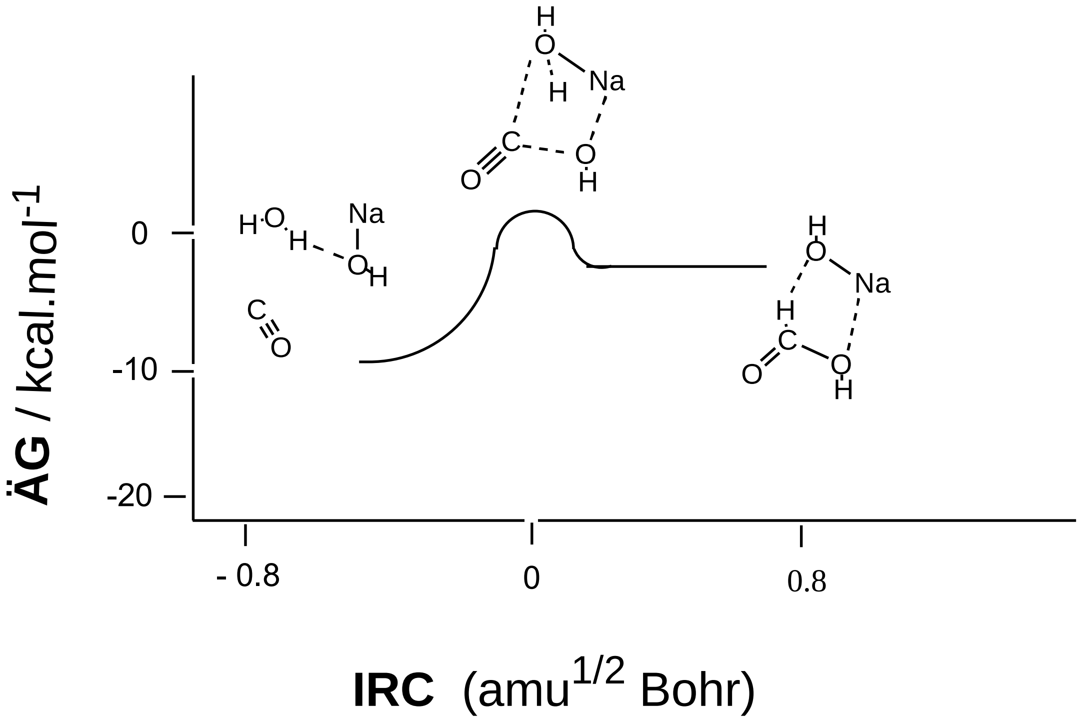

Figure-16

Intermediate (I-22)

C	-1.13293500	-0.22577800	0.49650400
O	-1.10406500	-1.34790900	0.54372900
H	0.64420400	1.54616200	-0.64935300
O	1.44505700	1.02901600	-0.74026400
H	1.87564800	1.10475900	0.11134200

Zero-point correction= 0.028618 (Hartree/Particle)
Thermal correction to Energy= 0.034675
Thermal correction to Enthalpy= 0.035619
Thermal correction to Gibbs Free Energy= -0.000234
Sum of electronic and zero-point Energies= -189.725458
Sum of electronic and thermal Energies= -189.719400
Sum of electronic and thermal Enthalpies= -189.718456
Sum of electronic and thermal Free Energies= -189.754309

Transition State

C	-0.22654900	0.94330000	0.07283000
O	-0.31436200	-0.09620200	0.53479800
H	0.09066400	2.00949900	-0.26432800
O	1.36199800	1.56610200	-0.59401800
H	2.04688900	1.54665800	0.08315300

Zero-point correction= 0.026461 (Hartree/Particle)
Thermal correction to Energy= 0.030278
Thermal correction to Enthalpy= 0.031223
Thermal correction to Gibbs Free Energy= 0.001746
Sum of electronic and zero-point Energies= -189.631382
Sum of electronic and thermal Energies= -189.627565
Sum of electronic and thermal Enthalpies= -189.626621
Sum of electronic and thermal Free Energies= -189.656098

Product (6)

C	-0.33643800	0.26007100	0.01110800
O	-0.46964000	-0.79034800	0.56218200
H	-1.15592500	0.92557900	-0.28427600
O	0.83541800	0.80019600	-0.33748600
H	1.52912900	0.18775500	-0.06205300

Zero-point correction= 0.034264 (Hartree/Particle)
Thermal correction to Energy= 0.037419
Thermal correction to Enthalpy= 0.038363

Thermal correction to Gibbs Free Energy= 0.010204
Sum of electronic and zero-point Energies= -189.741834
Sum of electronic and thermal Energies= -189.738678
Sum of electronic and thermal Enthalpies= -189.737734
Sum of electronic and thermal Free Energies= -189.765894

IRC

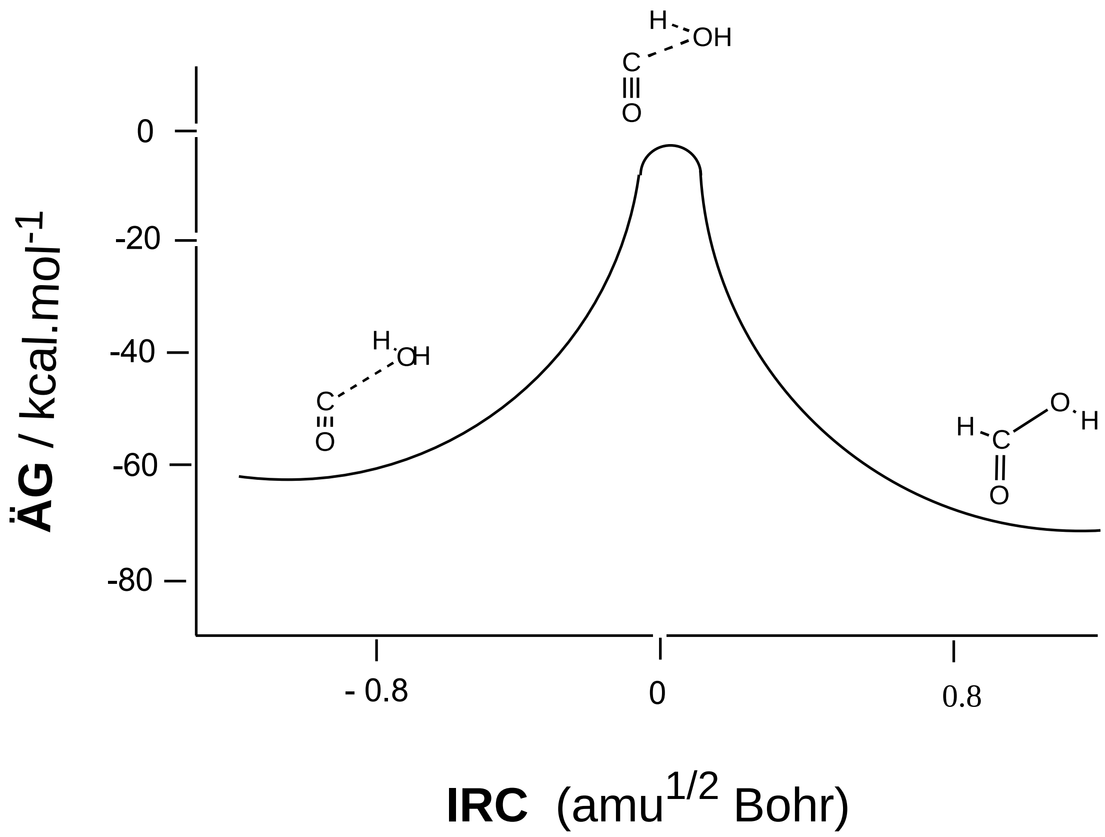

Figure-S1

Intermediate (I-2)

C	0.43787600	0.38714800	0.24138600
H	0.34632200	-0.69078300	0.34675400
Cl	1.50457500	1.01336200	1.49767100
Cl	1.08896600	0.72667000	-1.37508700
Cl	-1.19831300	1.07690100	0.40487700
O	-0.24930700	-2.59978400	-0.00589200
H	-0.84786600	-2.34882600	-0.72643300
H	-0.78336000	-3.09440600	0.61429900
O	-1.76690600	-1.33553000	-2.01322800
H	-1.10051700	-1.03903400	-2.63581300
H	-2.00821500	-0.54365900	-1.52559200

Zero-point correction= 0.069424 (Hartree/Particle)
Thermal correction to Energy= 0.080562
Thermal correction to Enthalpy= 0.081506
Thermal correction to Gibbs Free Energy= 0.030815
Sum of electronic and zero-point Energies= -1572.172415
Sum of electronic and thermal Energies= -1572.161277
Sum of electronic and thermal Enthalpies= -1572.160333
Sum of electronic and thermal Free Energies= -1572.211024

Transition State

C	-0.85942700	0.56810900	0.01116900
H	-1.40403100	0.59142800	-0.92887700
Cl	0.76863500	0.26406300	0.00982000
Cl	-1.37139000	2.05503000	-2.31184100
Cl	-1.56749500	1.20772400	1.37329500
O	-1.60169400	-1.67140800	-0.13991200
H	-1.67557200	-1.58286200	-1.11553800
H	-2.49591900	-1.82589100	0.16905700
O	-1.74345700	-1.01467400	-2.78220700
H	-1.05497600	-1.31431500	-3.37578700
H	-1.73587100	-0.03864900	-2.86075300

Zero-point correction= 0.067575 (Hartree/Particle)
Thermal correction to Energy= 0.077796
Thermal correction to Enthalpy= 0.078740
Thermal correction to Gibbs Free Energy= 0.029659
Sum of electronic and zero-point Energies= -1572.096785
Sum of electronic and thermal Energies= -1572.086564
Sum of electronic and thermal Enthalpies= -1572.085620
Sum of electronic and thermal Free Energies= -1572.134701

Product (1)

C	-0.14537900	-0.37131400	0.73017600
H	-0.31628900	0.40345900	-0.00780700
Cl	1.54282800	-0.96057600	0.49192400
Cl	-0.75020400	1.28242700	-2.52005900
Cl	-0.29091900	0.35278200	2.35411100
O	-1.05292600	-1.37134500	0.53708300
H	-0.89584900	-1.87565300	-1.41511000
H	-0.96499200	-2.02830700	1.23482000
O	-0.58113100	-1.76179700	-2.31968400
H	0.35165800	-1.98239300	-2.28367600
H	-0.70521400	-0.03436400	-2.55697400

Zero-point correction= 0.068639 (Hartree/Particle)
Thermal correction to Energy= 0.078877
Thermal correction to Enthalpy= 0.079821
Thermal correction to Gibbs Free Energy= 0.030115
Sum of electronic and zero-point Energies= -1572.185587
Sum of electronic and thermal Energies= -1572.175349
Sum of electronic and thermal Enthalpies= -1572.17440
Sum of electronic and thermal Free Energies= -1572.224111

IRC

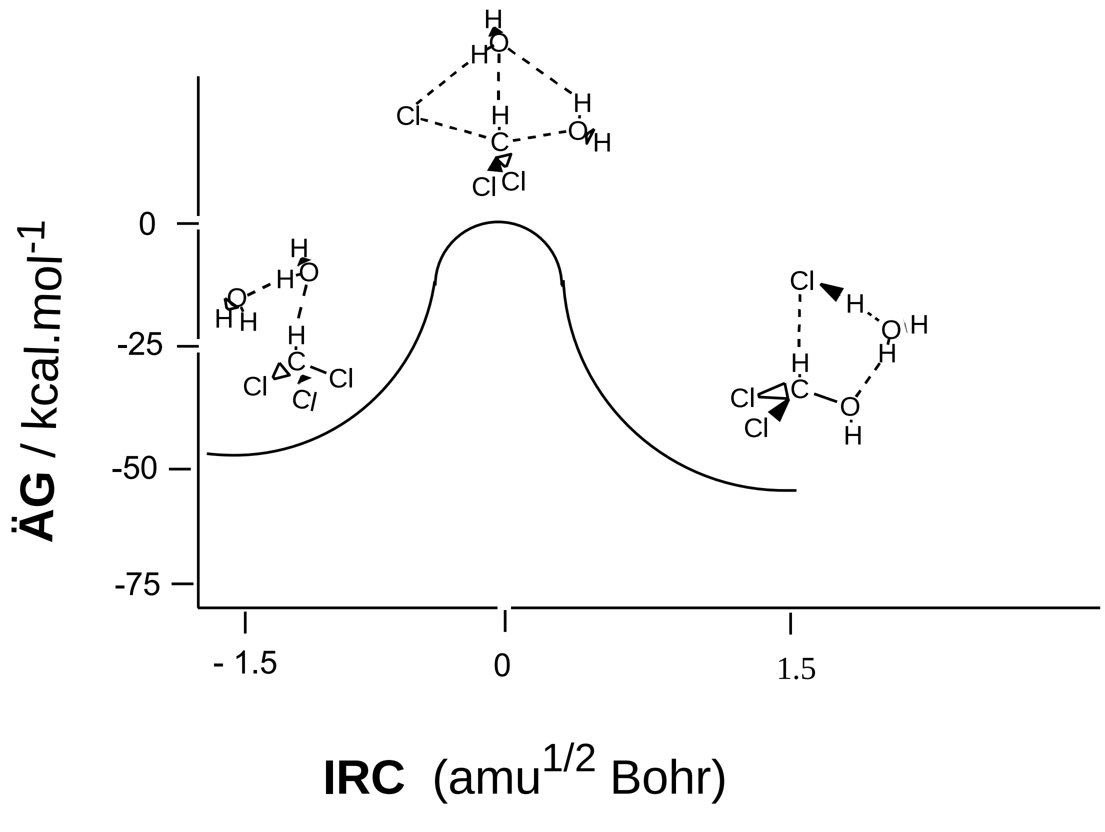

Figure-S2

Intermediate (I-4)

C	-0.53455800	0.19393100	0.09697600
H	0.29979700	0.36716800	0.83117400
Cl	0.11554700	-1.03024800	-1.08738600
Cl	-1.95281700	-0.47964100	0.90149400
Cl	-0.93086300	1.67913700	-0.76874700
O	1.77009000	0.30609500	1.65197900
H	1.82741700	0.87490500	2.41263900
Na	2.59031300	-1.05962300	0.40467600

Zero-point correction= 0.032124 (Hartree/Particle)

Thermal correction to Energy= 0.041257

Thermal correction to Enthalpy= 0.042201

Thermal correction to Gibbs Free Energy= -0.004711

Sum of electronic and zero-point Energies= -1657.475093

Sum of electronic and thermal Energies= -1657.465960

Sum of electronic and thermal Enthalpies= -1657.465016

Sum of electronic and thermal Free Energies= -1657.511928

Transition State

C	-0.24569900	0.04995200	0.48343800
H	-0.61441500	0.06568400	1.48303000
Cl	0.15696100	-1.46442900	-0.29632200
Cl	-2.49204000	-0.12390900	0.06934600
Cl	-0.03376600	1.50685100	-0.45688000
O	1.52380200	0.14659700	1.30995400
H	1.45265600	0.87217600	1.93048600
Na	2.62599100	-0.09629200	-0.47207700

Zero-point correction= 0.032613 (Hartree/Particle)

Thermal correction to Energy= 0.040900

Thermal correction to Enthalpy= 0.041844

Thermal correction to Gibbs Free Energy= -0.002166

Sum of electronic and zero-point Energies= -1657.416173

Sum of electronic and thermal Energies= -1657.407887

Sum of electronic and thermal Enthalpies= -1657.406943

Sum of electronic and thermal Free Energies= -1657.450953

Product (1)

C	0.52276500	-0.12433600	0.08025200
H	0.32609700	0.81394700	-0.42804100
Cl	2.17732400	-0.12262900	0.70060800
Cl	-2.06634400	-0.58941900	-2.25679400

Cl	-0.62312800	-0.00427000	1.59024400
O	0.26208300	-1.21820700	-0.61991600
H	-0.40735000	-0.99799600	-1.34279700
Na	-2.47302600	-1.43322700	0.07299800

Zero-point correction= 0.035400 (Hartree/Particle)
Thermal correction to Energy= 0.043767
Thermal correction to Enthalpy= 0.044711
Thermal correction to Gibbs Free Energy= -0.000912
Sum of electronic and zero-point Energies= -1657.550989
Sum of electronic and thermal Energies= -1657.542622
Sum of electronic and thermal Enthalpies= -1657.541678
Sum of electronic and thermal Free Energies= -1657.587301

IRC

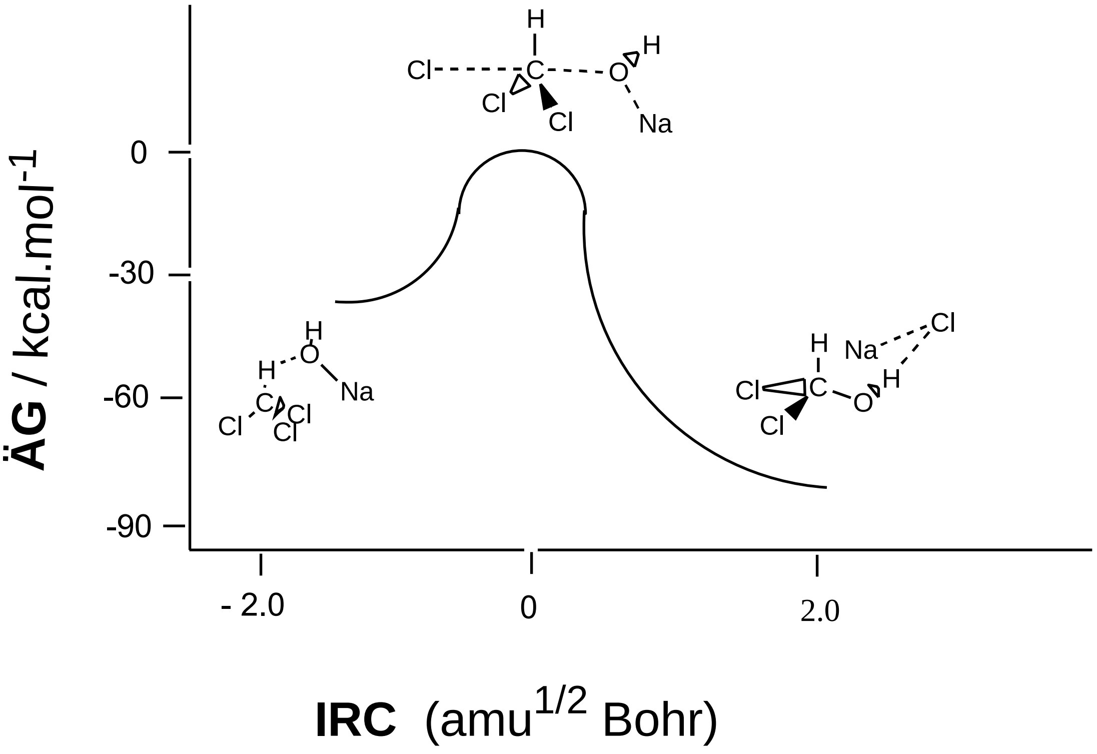

Figure-S3

Intermediate (I-6)

C	-0.48723500	0.41532800	0.59948900
H	0.40805400	0.68642300	0.00076800
Cl	-0.19667500	-1.26171700	1.20492500
Cl	-0.68590300	1.51419300	1.96260400
Cl	-1.91551100	0.42547800	-0.44716900
O	1.83890700	0.59659600	-1.03420300
H	2.30855400	1.41727200	-1.14881000
Na	1.95350900	-1.51587700	-0.84231500
O	0.72549400	-0.94765600	-2.71410200
H	-0.21726500	-0.83454300	-2.82233600
H	1.06622200	-0.11256900	-2.26246600

Zero-point correction= 0.058242 (Hartree/Particle)
Thermal correction to Energy= 0.069600
Thermal correction to Enthalpy= 0.070544
Thermal correction to Gibbs Free Energy= 0.019038
Sum of electronic and zero-point Energies= -1733.921320
Sum of electronic and thermal Energies= -1733.909962
Sum of electronic and thermal Enthalpies= -1733.909018
Sum of electronic and thermal Free Energies= -1733.960525

Transition State

C	-0.86573900	-0.01540000	1.16835000
H	-0.23803500	0.66090500	1.70109500
Cl	-0.80631000	-1.72464900	1.53816400
Cl	-2.09100000	0.49797300	3.11766500
Cl	-2.07155700	0.52243800	0.06385900
O	0.56416000	-0.13900900	-0.14991500
H	0.78136000	0.77589400	-0.32818200
Na	1.31015900	-2.18090900	-0.27900900
O	0.01398400	-1.80962600	-2.14571600
H	-0.86726200	-2.02539000	-2.44657800
H	-0.05706600	-0.97423200	-1.63416100

Zero-point correction= 0.058020 (Hartree/Particle)
Thermal correction to Energy= 0.069103
Thermal correction to Enthalpy= 0.070048
Thermal correction to Gibbs Free Energy= 0.019197
Sum of electronic and zero-point Energies= -1733.856942
Sum of electronic and thermal Energies= -1733.845859
Sum of electronic and thermal Enthalpies= -1733.844914
Sum of electronic and thermal Free Energies= -1733.895765

Product (2)

C	-1.01210100	0.53556500	-0.09047500
H	-1.27209600	-0.07862000	0.76902200
Cl	0.38158000	-2.08909500	-0.02242500
Cl	2.56421500	1.73082900	1.31926400
Cl	-2.13304600	0.30225900	-1.41870500
O	-0.12002800	1.32357500	-0.16382800
H	1.31335000	1.70556100	0.95031300
Na	2.19281800	-0.38842200	-0.64031700
O	1.24807200	-0.85988000	-2.70885500
H	0.70232900	-1.46547700	-2.17177900
H	0.63930700	-0.38655600	-3.27488800

Zero-point correction= 0.055088 (Hartree/Particle)
Thermal correction to Energy= 0.068099
Thermal correction to Enthalpy= 0.069043
Thermal correction to Gibbs Free Energy= 0.011109
Sum of electronic and zero-point Energies= -1733.991720
Sum of electronic and thermal Energies= -1733.978710
Sum of electronic and thermal Enthalpies= -1733.977766
Sum of electronic and thermal Free Energies= -1734.035700

IRC

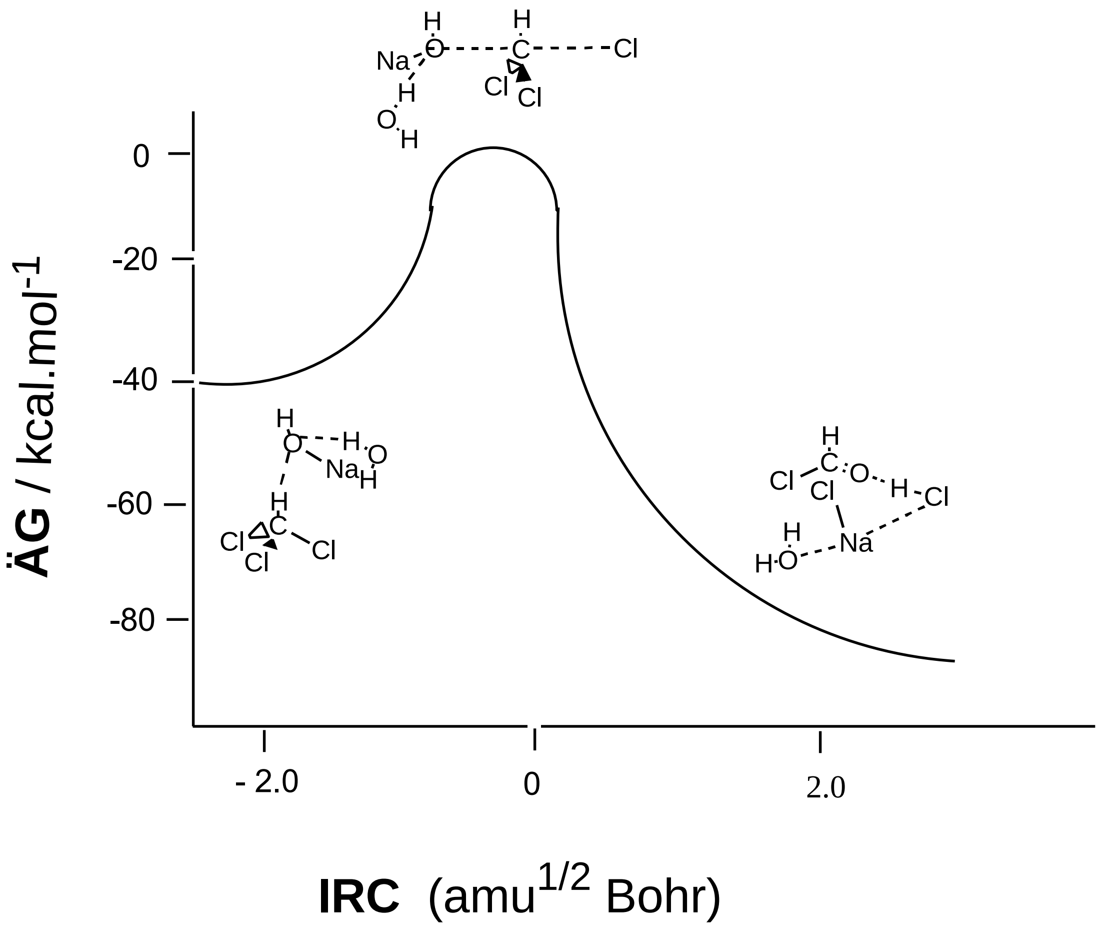

Figure-S5

Intermediate-(I-10)

C	-0.27754900	-0.13291500	0.11099600
H	-1.14059700	-0.72055600	-0.17596300
Cl	1.14309000	-0.87508800	-0.65294100
Cl	-0.48939300	1.53336100	-0.44725500
Cl	-0.14801700	-0.20085700	1.87568000
O	-2.09730200	-2.63711900	-0.18049200
H	-2.17637900	-2.91922100	0.73145600
O	2.53590500	1.94335800	-2.38547100
H	1.70999700	1.52226900	-2.62411700
H	2.56234400	1.86636200	-1.43158900
H	-1.49093800	-3.26084200	-0.58149500

Zero-point correction= 0.062662 (Hartree/Particle)
Thermal correction to Energy= 0.075254
Thermal correction to Enthalpy= 0.076198
Thermal correction to Gibbs Free Energy= 0.019213
Sum of electronic and zero-point Energies= -1572.088536
Sum of electronic and thermal Energies= -1572.075944
Sum of electronic and thermal Enthalpies= -1572.075000
Sum of electronic and thermal Free Energies= -1572.131985

Transition State-1

C	-1.15076700	0.11285800	0.46003100
H	-1.65768500	-1.18471400	-0.17437900
Cl	1.00056900	-1.65998600	-0.88822500
Cl	-0.98836700	1.51810800	-0.41507300
Cl	-0.56275200	0.20289300	2.04007500
O	-1.70561900	-2.11445200	-0.76795600
H	-1.89870100	-2.86025000	-0.19285200
O	1.32079900	1.20936200	-2.40856800
H	1.23909000	0.28712600	-2.12922700
H	2.22773600	1.43193500	-2.20039200
H	-0.62312300	-2.11489300	-0.96067500

Zero-point correction= 0.061748 (Hartree/Particle)
Thermal correction to Energy= 0.072324
Thermal correction to Enthalpy= 0.073268
Thermal correction to Gibbs Free Energy= 0.022765
Sum of electronic and zero-point Energies= -1572.080260
Sum of electronic and thermal Energies= -1572.069684
Sum of electronic and thermal Enthalpies= -1572.068740
Sum of electronic and thermal Free Energies= -1572.119243

Intermediate-(I-11)

C	-1.14138200	0.41462200	1.12936200
H	-1.40668100	-1.37393800	0.28300400
Cl	0.99327600	-1.90290400	-2.13782200
Cl	-0.11417800	1.32793200	0.15332300
Cl	-1.61612400	1.26752000	2.52900400
O	-1.34849200	-2.15706900	-0.29807300
H	-1.24107000	-2.91131600	0.28245400
O	1.83876400	1.42615600	-2.16799500
H	1.51435900	0.53257800	-2.31788400
H	2.77596100	1.31221400	-2.01145900
H	-0.06084900	-2.00298000	-1.32470900

Zero-point correction= 0.066847 (Hartree/Particle)
Thermal correction to Energy= 0.079834
Thermal correction to Enthalpy= 0.080778
Thermal correction to Gibbs Free Energy= 0.022345
Sum of electronic and zero-point Energies= -1572.162989
Sum of electronic and thermal Energies= -1572.150002
Sum of electronic and thermal Enthalpies= -1572.149058
Sum of electronic and thermal Free Energies= -1572.207490

IRC

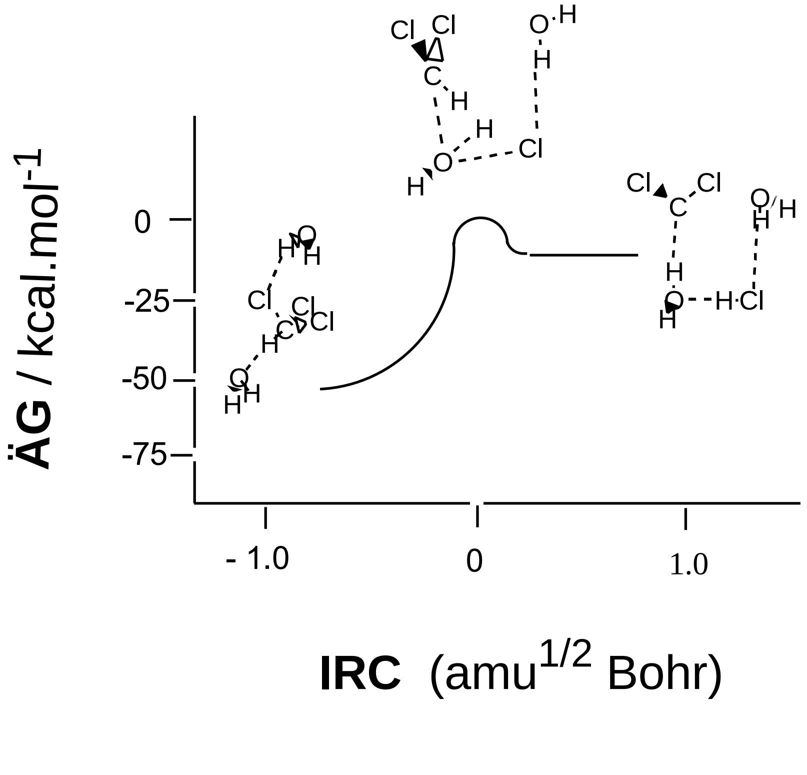

Transition State-2

C	-0.28549100	-0.17473200	0.14786600
H	-0.78641000	-0.53840000	-1.00762600
Cl	1.12689800	-1.06505700	0.64452800
Cl	-1.23260200	2.17833800	-1.97834100
Cl	-1.01464100	0.68926700	1.49671500
O	-1.32707500	-1.30740700	-0.34301200
H	-2.22378200	-0.87448700	-0.31601000
O	-3.56252100	0.14387700	-0.60577800
H	-3.22099100	0.86495500	-1.14855800
H	-3.84952800	0.56163200	0.20829700
H	-0.57250300	1.32933700	-1.20739300

Zero-point correction= 0.061899 (Hartree/Particle)
Thermal correction to Energy= 0.072033
Thermal correction to Enthalpy= 0.072977
Thermal correction to Gibbs Free Energy= 0.024762
Sum of electronic and zero-point Energies= -1572.059755
Sum of electronic and thermal Energies= -1572.049621
Sum of electronic and thermal Enthalpies= -1572.048677
Sum of electronic and thermal Free Energies= -1572.096893

Product (1)

C	0.44968800	-1.01276000	0.18340200
H	0.51140800	-0.83703900	-0.88742700
Cl	1.74629500	-2.12610400	0.63655100
Cl	-0.64633300	2.22782500	-1.89358100
Cl	0.86132900	0.66566200	0.89924600
O	-0.72017500	-1.46079400	0.62844000
H	-1.44841600	-1.00921000	0.14367800
O	-2.63445600	-0.13009100	-0.72068400
H	-2.25571200	0.58952800	-1.23877200
H	-3.27943400	0.28158700	-0.14410300
H	-0.01047400	1.83197100	-0.82899800

Zero-point correction= 0.067890 (Hartree/Particle)
Thermal correction to Energy= 0.078285
Thermal correction to Enthalpy= 0.079229
Thermal correction to Gibbs Free Energy= 0.029033
Sum of electronic and zero-point Energies= -1572.185751
Sum of electronic and thermal Energies= -1572.175356
Sum of electronic and thermal Enthalpies= -1572.174412
Sum of electronic and thermal Free Energies= -1572.224608

IRC

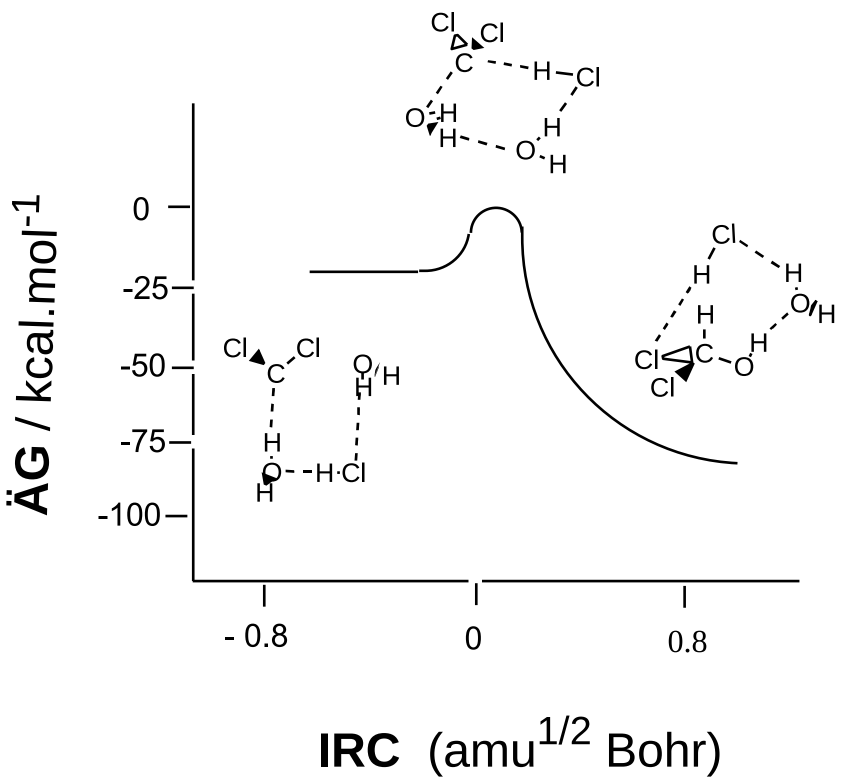

Figure-S6

Intermediate (I-17)

C	1.33143700	0.52385800	-0.92339200
H	0.26054000	0.43880600	-1.15990000
Cl	1.64222900	-0.31802100	0.67475200
O	2.20175400	1.03766100	-1.51071900
O	-1.69375500	0.00694000	-1.21908800
H	-2.34945800	0.70273500	-1.19402800
H	-1.80698200	-0.48996500	-0.39242500
O	-1.52213100	-1.34051800	1.21282500
H	-1.51630500	-2.29626800	1.15234200
H	-0.60065500	-1.09079200	1.33927000

Zero-point correction= 0.068840 (Hartree/Particle)
Thermal correction to Energy= 0.078512
Thermal correction to Enthalpy= 0.079457
Thermal correction to Gibbs Free Energy= 0.032914
Sum of electronic and zero-point Energies= -726.969526
Sum of electronic and thermal Energies= -726.959853
Sum of electronic and thermal Enthalpies= -726.958909
Sum of electronic and thermal Free Energies= -727.005452

Transition State

C	1.25359300	0.00680900	1.24951200
H	0.01033100	0.32117900	1.14040000
Cl	0.79043200	1.30440600	3.39140600
O	2.32703800	-0.28769900	1.09027600
O	-1.17422700	0.77955800	1.37791700
H	-1.38945800	1.54165400	0.83390900
H	-0.87142600	1.13497100	2.26244800
O	0.16892600	-1.71605700	2.84345200
H	0.79248400	-2.36192500	3.17635600
H	0.27681200	-0.94314500	3.41739000

Zero-point correction= 0.064585 (Hartree/Particle)
Thermal correction to Energy= 0.072996
Thermal correction to Enthalpy= 0.073941
Thermal correction to Gibbs Free Energy= 0.031745
Sum of electronic and zero-point Energies= -726.934274
Sum of electronic and thermal Energies= -726.925863
Sum of electronic and thermal Enthalpies= -726.924919
Sum of electronic and thermal Free Energies= -726.967115

Product (4)

C	1.01425300	-0.28486100	-2.12633700
H	-1.57460900	-0.51982700	-0.35930100
Cl	0.04359900	1.58434000	1.23753800
O	2.07851200	-0.61399000	-1.98887800
O	-1.83752300	0.34771600	-0.71297600
H	-1.41776000	0.39320600	-1.57512700
H	-0.86194000	1.18906000	0.33565000
O	-0.50040100	-1.65307900	0.65619800
H	-0.05070900	-0.95898300	1.15106200
H	-0.87885000	-2.23560800	1.31488800

Zero-point correction= 0.064206 (Hartree/Particle)
Thermal correction to Energy= 0.075011
Thermal correction to Enthalpy= 0.075955
Thermal correction to Gibbs Free Energy= 0.025333
Sum of electronic and zero-point Energies= -726.978933
Sum of electronic and thermal Energies= -726.968128
Sum of electronic and thermal Enthalpies= -726.967184
Sum of electronic and thermal Free Energies= -727.017806

IRC

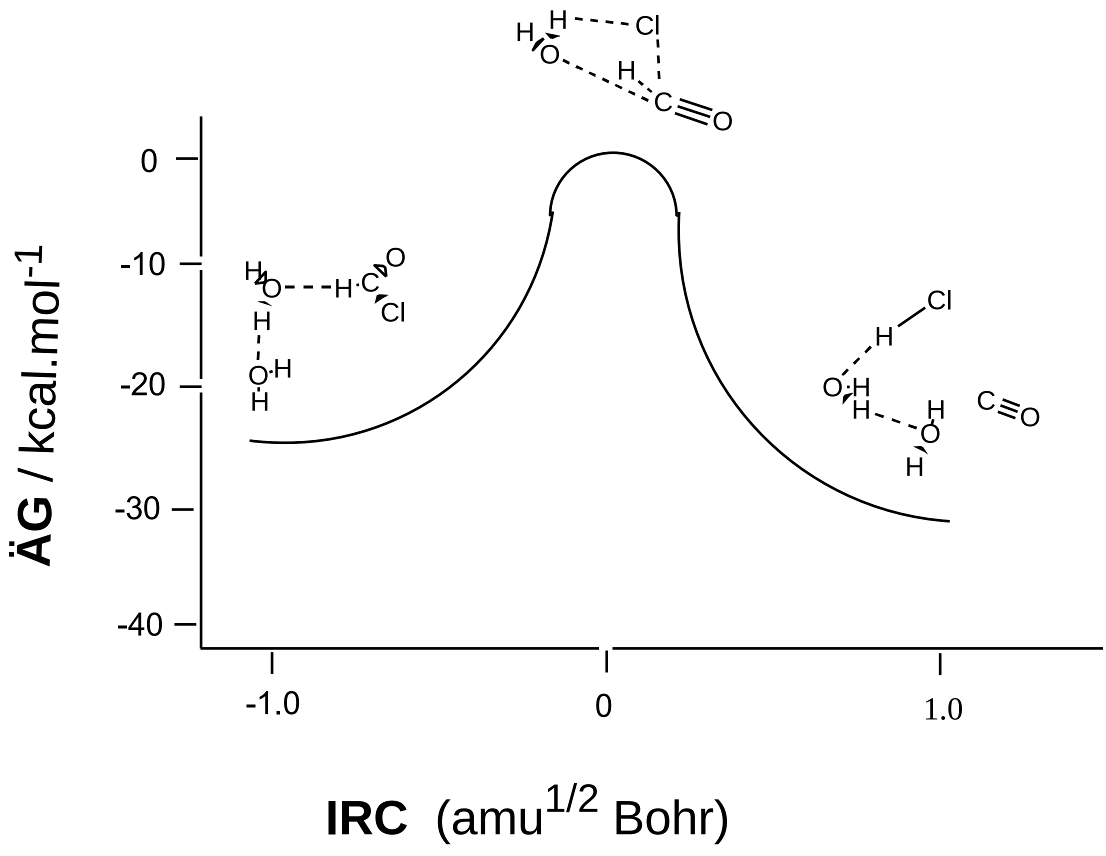

Figure-S7

Intermediate (I-23)

C	-0.53774900	-0.86690100	1.10306900
O	0.40082100	-0.07546400	-1.67564100
O	-0.74237600	-1.96518500	0.99685500
H	1.29060000	-0.35284700	-1.89029800
O	0.41491800	2.04024700	0.24165700
H	-0.32062500	2.64041000	0.11669500
H	0.08152500	1.34997100	0.82320000
H	0.50897000	0.75300700	-1.18939800

Zero-point correction= 0.054054 (Hartree/Particle)
Thermal correction to Energy= 0.062883
Thermal correction to Enthalpy= 0.063827
Thermal correction to Gibbs Free Energy= 0.020872
Sum of electronic and zero-point Energies= -266.147923
Sum of electronic and thermal Energies= -266.139095
Sum of electronic and thermal Enthalpies= -266.138150
Sum of electronic and thermal Free Energies= -266.181106

Transition State

C	-0.63772700	0.39785600	0.67503100
O	-0.12745700	0.85874600	-0.98609900
O	-0.80952000	-0.72262200	0.85199900
H	0.56768100	0.28885000	-1.31996100
O	0.03719800	2.74522900	0.37085700
H	-0.62742600	3.41236000	0.19135600
H	-0.45242600	1.73913700	0.88209400
H	0.12077500	2.00949800	-0.51031700

Zero-point correction= 0.050928 (Hartree/Particle)
Thermal correction to Energy= 0.055957
Thermal correction to Enthalpy= 0.056901
Thermal correction to Gibbs Free Energy= 0.023510
Sum of electronic and zero-point Energies= -266.080322
Sum of electronic and thermal Energies= -266.075294
Sum of electronic and thermal Enthalpies= -266.074350
Sum of electronic and thermal Free Energies= -266.107741

Product (6)

C	-0.21030300	-0.75701000	0.27778900
O	0.33131100	-0.34814800	-0.88519900
O	-0.50922500	-1.88660400	0.51559300
H	0.43068000	-1.12489200	-1.44929000

O	0.45294600	2.40994100	0.21066100
H	-0.26803200	2.92896800	-0.14520700
H	-0.32514000	0.10208100	0.94703100
H	0.68694300	1.80182300	-0.49396600

Zero-point correction= 0.058121 (Hartree/Particle)
Thermal correction to Energy= 0.065095
Thermal correction to Enthalpy= 0.066039
Thermal correction to Gibbs Free Energy= 0.027141
Sum of electronic and zero-point Energies= -266.159286
Sum of electronic and thermal Energies= -266.152312
Sum of electronic and thermal Enthalpies= -266.151368
Sum of electronic and thermal Free Energies= -266.190266

IRC

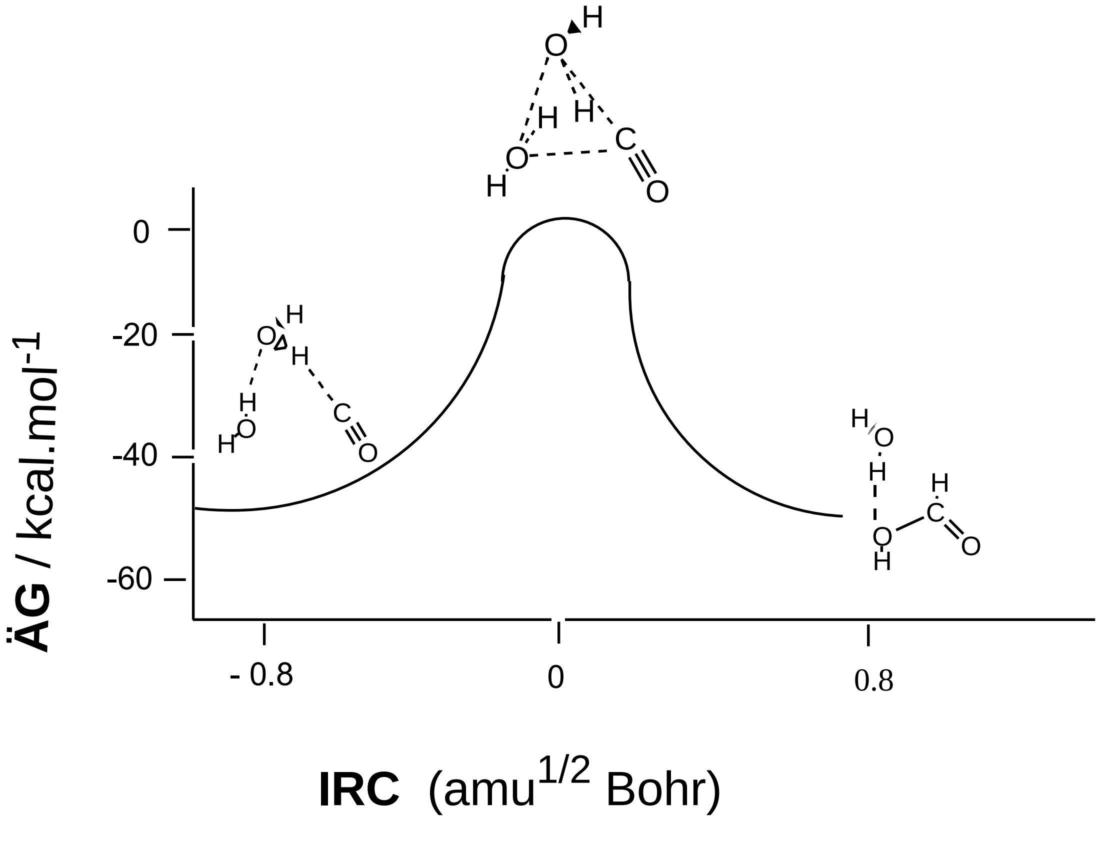

Supplement: Supplementary file 1 — jo4c00942_si_001.pdf [file jo4c00942_si_001.pdf]
